# Beyond Word Frequency: Bursts, Lulls, and Scaling in the Temporal Distributions of Words

**DOI:** 10.1371/journal.pone.0007678

**Published:** 2009-11-11

**Authors:** Eduardo G. Altmann, Janet B. Pierrehumbert, Adilson E. Motter

**Affiliations:** 1 Northwestern Institute on Complex Systems, Northwestern University, Evanston, Illinois, United States of America; 2 Department of Linguistics, Northwestern University, Evanston, Illinois, United States of America; 3 Department of Physics and Astronomy, Northwestern University, Evanston, Illinois, United States of America; University of East Piedmont, Italy

## Abstract

**Background:**

Zipf's discovery that word frequency distributions obey a power law established parallels between biological and physical processes, and language, laying the groundwork for a complex systems perspective on human communication. More recent research has also identified scaling regularities in the dynamics underlying the successive occurrences of events, suggesting the possibility of similar findings for language as well.

**Methodology/Principal Findings:**

By considering frequent words in USENET discussion groups and in disparate databases where the language has different levels of formality, here we show that the distributions of distances between successive occurrences of the same word display bursty deviations from a Poisson process and are well characterized by a stretched exponential (Weibull) scaling. The extent of this deviation depends strongly on semantic type – a measure of the logicality of each word – and less strongly on frequency. We develop a generative model of this behavior that fully determines the dynamics of word usage.

**Conclusions/Significance:**

Recurrence patterns of words are well described by a stretched exponential distribution of recurrence times, an empirical scaling that cannot be anticipated from Zipf's law. Because the use of words provides a uniquely precise and powerful lens on human thought and activity, our findings also have implications for other overt manifestations of collective human dynamics.

## Introduction

Research on the distribution of time intervals between successive occurrences of events has revealed correspondences between natural phenomena on the one hand [1], [2] and social activities on the other hand [3]–[5]. These studies consistently report bursty deviations both from random and from regular temporal distributions of events [6]. Taken together, they suggest the existence of a dynamic counterpart to the universal scaling laws in magnitude and frequency distributions [7]–[11]. Language, understood as an embodied system of representation and communication [12], is a particularly interesting and promising domain for further exploration, because it both epitomizes social activity, and provides a medium for conceptualizing natural and biological reality.

The fields of statistical natural language processing and psycholinguistics study language from a dynamical point of view. Both treat language processing as encoding and decoding of information. In psycholinguistics, the local likelihood (or predictability) of words is a central focus of current research [13]. Many widely used practical applications of statistical natural language processing, such as document retrieval based on keywords, also exploit dynamic patterns in word statistics [10], [14], [15]. Particularly important for these applications, and also noticed in different contexts [16]–[21], is the non-uniform distribution of content words through a text, suggesting that connections to the previous discoveries about inter-event distributions may be revealed through a systematic investigation of the recurrence times of different words.

With the rise of the Internet, large records of spontaneous and collective language are now available for scientific inquiry [22]–[24], allowing statistical questions about language to be investigated with an unprecedented precision. At the same time, large-scale text mining and document classification is of ever-increasing importance [25]. The primary datasets used in our study are USENET discussion groups available through Google (http://groups.google.com). These exemplify spontaneous linguistic interactions in large communities over a long period of time. We first focus on the 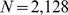 words that occurred more than 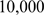 times between Sept. 1986 and Mar. 2008 in a (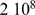-word) discussion group, talk.origins. The data were collated chronologically, maintaining the thread structure (see Text S1, *Databases*).

Here, we show that long-time word recurrence patterns follow a stretched exponential distribution, owing to bursts and lulls in word usage. We focus on time scales that exceed the scale of *syntactic* relations, and the burstiness of the words is driven by their semantics (that is, by what they mean). The burstiness of physical events and socially contextualized choices makes words more bursty than an exponential distribution. However, we show that words are typically less bursty than other human activities [26] due to their *logicality* or *permutability* [27], [28], technical constructs of formal semantics that index the extent to which the meanings and usage of words are stable over changes in the discourse context. Our quantitative analysis of the empirical data confirms the inverse relationship between burstiness and permutability. The model we develop to explain these observations shares the generative spirit of local (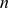-gram) and weakly non-local models of text classification and generation [29]–[31]. However it focuses on long time-scales, picking up at temporal scales where studies of local predictability and coherence leave off [13]. We verify the generality of our main findings using different databases, including books of different genres and a series of political debates.

## Methods

We are interested in the temporal distribution of each word 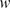. All words are enumerated in order of appearance, 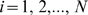, where 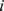 plays the role of the time along the text. The recurrence time 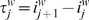 is defined by the number of words between two successive uses (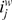 and 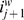) of word 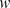 (plus one). For instance, the first appearances of the word *the* in the abstract above are at 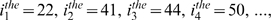 leading to a sequence of recurrence times 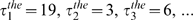. We are interested in the distribution 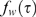 of 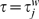, 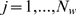. The mean recurrence time, called by Zipf the wavelength of the word [7], is given by 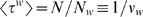 [2] (hereafter we drop 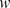 from our notation). It is mathematically convenient to consider 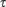 to be a continuous time variable (an assumption that is justified by our interested in 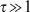) and to use the cumulative probability density function defined by 
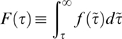
, which satisfies 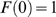 and 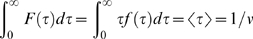.

The first point of interest is how the distribution 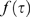 [or 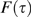] deviates from the exponential distribution
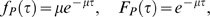
(1)where 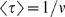 leads to 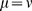. The exponential distribution is predicted by a simple *bag-of-words* model in which the probability 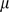 of using the word is time independent and equals 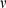 (a Poisson process with rate 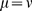) [14], [15], [19], [25], [29], as observed if the words in the text are randomly permuted. Deviations are caused by the way that people choose their words in context. Numerous studies, as reviewed in Ref. [32], already demonstrate that the language users dynamically modify their use of nouns and noun phrases as a function of the linguistic and external context. We analyze such modifications for all types of words.

## Results and Discussion

**Figure 1:**
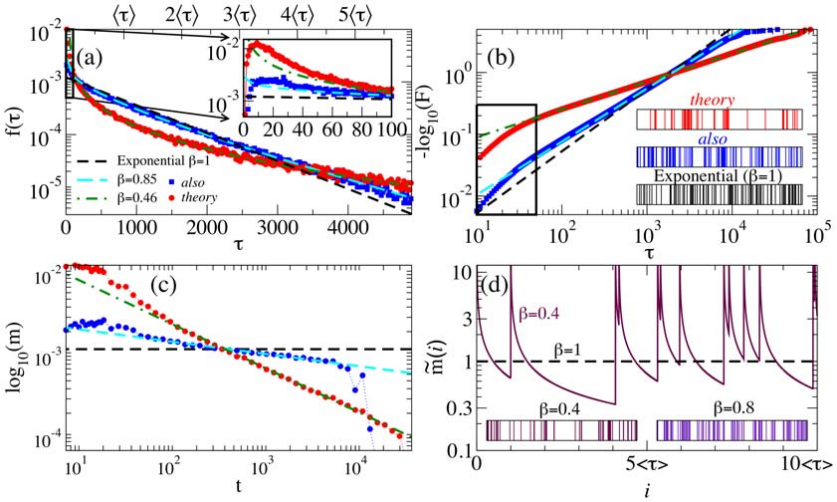
Recurrence time distributions for the words *theory* (red) and *also* (blue) in the USENET group talk.origins, a discussion group about evolution and creationism. Both words have a mean recurrence time of 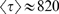. (a) Linear-logarithmic representation of 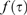, showing that the decay is slower than the exponential 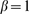 prediction (1) (black dashed line) and follows closely the stretched exponential distribution (2) with 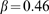 (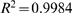) for *theory* and 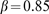 (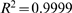) for *also*. For comparison, 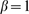 yields 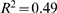 for the word *theory* and 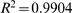 for the word *also* (see Text S1, *Fitting Procedures*). The inset in (a) shows a magnification for short times. A word-dependent peak at 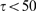 reflects the domination of syntactic effects and local discourse structure at this scale. (b) Cumulative distribution function 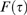 in a scale in which the stretched exponential (2) appears as a straight line. The panels in the inset show 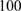 occurrences (top to bottom): of the word *theory*, of the word *also*, and of a randomly distributed word (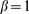). (c) The probability of word usage 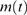 for the words *theory* and *also*. The data are binned logarithmically and the straight lines correspond to Eq. (4). (d) Illustration of the generative model for the usage of individual words when 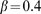, where the spikes indicate the times at which the word is used. The probability 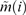 of using a word decays as a piece-wise power-law function since its last use, as determined by Eq. (4). The Poisson case corresponds to constant 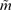. The panels at the bottom show 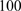 occurrences of words generated by the model for 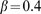 and 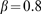.

Figure 1 shows the empirical results obtained for the example words *theory* and *also* in the talk.origins group of the USENET database. Both words have 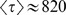 but are linguistically quite different: while *theory* is a common noun, *also* is an adverb that functions semantically as an operator. The deviation from the Poisson prediction (1) is apparent in Fig. 1(a–c): 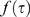 is larger than the exponential distribution for distances 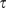 both much shorter and much longer than 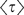, while it is smaller for 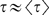. Both words exhibit a most probable recurrence time 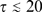 and a monotonically decaying distribution 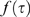 for larger times [Fig. 1(a)]. Comparing the insets in Fig. 1(b), one sees that the occurrences of *theory* are clustered close to each other in a phenomenon known as burstiness [6], [14], [15], [19], [21]. Due to burstiness, the frequency of the word *theory* estimated from a small sample would differ a great deal as a function of exactly where the sample was drawn. Similar but lesser deviations are observed for the word *also*.

Central to our discussion, Fig. 1 shows that the distributions of both words can be well described by the single free parameter 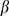 of the stretched exponential distribution
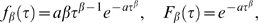
(2)where 
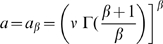
 is obtained by imposing 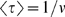, 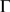 is the Gamma function, and 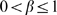. Distribution (2), also known as Weibull distribution, and similar stretched exponential distributions describe a variety of phenomena [6], [23], [33]–[35], including the recurrence time between extreme events in time series with long-term correlations [2], [36]. The stretched exponential (2) is more skewed than the simple exponential distribution (1), which corresponds to the limiting case 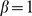, but less skewed than a power law, which is approached for 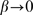.

A crucial test for the claim that an empirical distribution 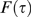 follows a stretched exponential 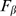 is to represent 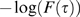 as a function of 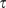 in a double logarithmic plot [2]. The straight line behavior for almost three decades shown in Fig. 1(b), which is illustrative of the words in our datasets, provides strong evidence for the stretched exponential scaling (spam-related deviations for long 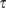 are discussed in Text S1, *Databases*). This is a clear advance over the closest precedents to our results: (i) In Ref. [8] Zipf proposed a power-law decay, which would appear as an horizontal line in Fig. 1b. (ii) Refs. [14], [15] compare two non-stationary Poisson processes for predicting the counts of words in documents (see Text S1, *Counting Distribution*); (iii) Ref. [19] proposes a non-homogeneous Poisson process for recurrence times, using a mixture of two exponentials with a total of four free parameters; (iv) Ref. [37] uses the Zipf-Alekseev distribution 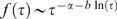, which we found to underestimate the decay rate for large 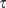 and to leave larger residuals than our fittings (see Text S1, *Zipf-Alekseev Distribution*). The stretched exponential distribution was found to describe the time between usages of words in Blogs and RSS feeds in Ref. [24]. However, time was measured as actual time and the same distribution was found for different types of words, suggesting that their observations are driven by the bursty update of webpages, a related but different effect. More strongly related to our study is Ref. [5]'s analysis of email activity, in which a non-homogeneous Poisson process captures the way one email can trigger the next.

### Generative Model

Motivated by the successful description of the stretched exponential distribution (2), we search for a generative stochastic process that can model word usage. We consider the inverse frequency 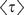 as given and focus on describing how the words are distributed throughout the text. We assume that our text (abstractly regarded as arbitrarily long) is generated by a well-defined stationary stochastic process with finite 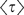 for the words of interest. We further assume that the probability 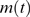 of using the word 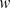 depends only on the distance 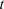 since the last occurrence of the word. The latter means that we are modeling the word usage as a *renewal process* [34], [36]. The distribution of recurrence times is then given by the (joint) probability of having the word at distance 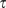 and not having this word for 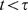:
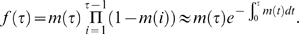
The cumulative distribution function is written as
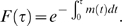
(3)The time dependent probability 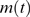, also known as *hazard function*, can be obtained empirically as 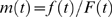 (see Text S1, *Hazard Function*). Equation (3) reduces to the exponential distribution (1) for a time independent probability 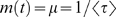. The stretched exponential distribution (2) is obtained from (3) by asserting that [34], [36], [38]

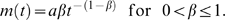
(4)This assertion means that in our model, the probability of using a word decays as a power law since the last use of that word. This is further justified by the power-law behavior of 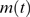 determined directly from the empirical data, as shown in Fig. 1(c) and Text S1, Fig. 9, and is in agreement with results from mathematical psychology [39], [40] and information retrieval [40]. The Weibull renewal process we propose can be analyzed formally as a particular instance of a doubly stochastic Poisson process [41].

Our model is illustrated in Fig. 1(d) and can be interpreted as a bag-of-words with memory that accounts for the burstiness of word usage. This model does not reproduce the positive correlations between 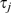 and 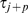 [2], [6], [20], which are usually small (less than 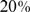
% for 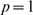) but decay slowly with 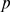 (see Text S1, *Correlation in* 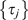). These correlations quantify the extent to which the renewal model is a good approximation of the actual generative process, and show that the burstiness of words exists not only as a departure of 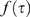 from the exponential distribution, but also as a clustering of small (large) 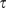 [6] (see Text S1, *Independence of* 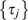). The advantage of the renewal description is that the model (i) can be substantiated to a vast literature describing power-law decay of memory in agreement with Eq. (4), see Refs. [39], [40] and references therein, and (ii) fully determines the dynamics (allowing, e.g., the precise derivation of counting distributions [38], which are used in applications to document classification [14], [15] and information retrieval [40]).

### Word Dependence

We have seen in Fig. 1 that the word-dependent deviation from the exponential distribution is encapsulated in the parameter 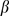: the smaller the 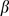 for any given word, the larger the deviation (see Text S1, *Deviation from the Exponential Distribution*). Next we investigate the dominant effects that determine the value of the parameter 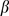 of a word. Previous research has observed that frequent *function* words (such as conjunctions and determiners) usually are closer to the random (Poisson) prediction while less frequent *content* words (particularly names and common nouns) are more bursty. These observations were quantified using: (i) an entropic analysis of texts [16]; (ii) the variance of the sequence of recurrence times [17]; (iii) the recurrence time distribution [19], [42]; and (iv) the related distribution of the number of occurrences of words per document [14], [15]. Because we have a large database and do not bin the datastream into documents, we are able to go beyond these insightful works and systematically examine frequency and linguistic status as factors in word burstiness.

Our large database allows a detailed analysis of words that, despite being in the same frequency range, have very different statistical behavior. For instance, in the range 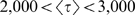, words with high 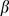 (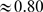) include *once, certainly, instead, yet, give, try, makes*, and *seem*; the few words with 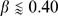 include *design, selection, intelligent*, and *Wilkins*. Corroborating Ref. [14], it is evident that words with low 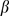 better characterize the discourse topic. However, these examples also show that the distinction between function words and content words cannot be explanatory. For instance, many content words, such as the adverbs and verbs of mental representation in the list just above, have 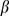 values as high as many function words. Here we obtain a deeper level of explanation by drawing on tools from formal semantics, specifically on type theory [27], [43], [44], and on dynamic theories of semantics [45], [46], which model how words and sentences update the discourse context over time. We use semantics rather than syntax because syntax governs how words are combined into sentences, and we are interested in much longer time scales over which syntactic relations are not defined. Type theory establishes a scale from simple entities (e.g., proper nouns) to high type words (e.g., words that cannot be described using first-order logic, including intensional expressions and operators). Simplifying the technical literature in the interests of good sample sizes and coding reliability, we define a ladder of four semantic classes, as listed in Table 1.

**Table 1:** Examples of the classification of words by semantic types.

Class	Name	Examples of words
1	Entities	Africa, Bible, Darwin
2	Predicates and Relations	blue, die, in, religion
3	Modifiers and Operators	believe, everyone, forty
4	Higher Level Operators	hence, let, supposedly, the

The primitive types are entities *e*, exemplified by proper nouns such as *Darwin* (Class 1), and truth values, *t* (which are the values of sentences). Predicates or relations, such as the simple verb *die*, and the adjective/noun blue, take entities as arguments and map them to sentences (e.g., *Darwin dies*, *Tahoe is blue*). They are classified as 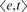 (Class 2). The notation 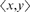 denotes a mapping from an element 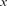 in the domain to the image 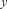 [43], [44]. The semantic types of higher Classes are established by assessing what mappings they perform when they are instantiated. For example, *everyone* is of type 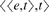 (Class 3), because it is a mapping from sets of properties of entities to truth values [44]; the verb *believe* shares this classification as a verb involving mental representation. The adverb *supposedly* is a higher order operator (Class 4), because it modifies other modifiers. Following Ref. [44] (contra Ref. [43]) words are coded by the lowest type in which they commonly occur (see Text S1, *Coding of Semantic Types*).

**Figure 2:**
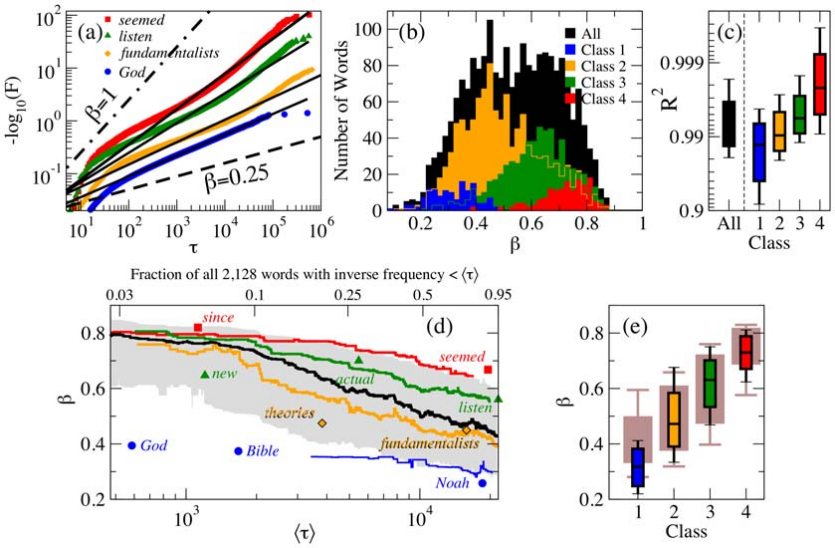
Dependence of 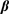 on semantic Class and frequency for the 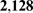 most frequent words of the USENET group talk.origins. Different classes of words (see Table 1) are marked in different colors. (a) Fitting of 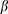 exemplified for four words with 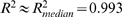 (bottom to top): *God*, Class 1, 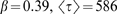; *fundamentalists*, Class 2, 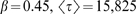; *listen*, Class 3, 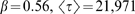; *seemed*, Class 4, 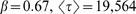. (b) Histogram of the fitted 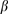, providing evidence that the Class is determinant to the value of 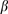. (c) Quality of fit quantified in terms of the coefficient of determination 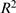 between the fitted stretched exponential and the empirical 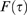 (see Text S1, *Quality of Fit*). The box-plots are centered at the median and indicate the 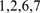 octiles. For comparison, an exponential fit with two free parameters yields 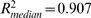 (see Text S1, *Deviation from the Exponential Distribution*). (d) Relative dependence of 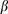 on Class and 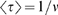 (inverse frequency), indicating: running median on words ordered according to 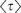 (center black line) and 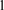-st and 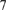-th octiles (boundaries of the gray region); and running medians on words by Class (colored lines, Class 1–4, from bottom to top) with illustrative words for each Class. At each 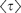, large variability in 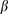 and a systematic ordering by Class is observed. (e) Box-plots of the variation of 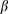 for words in a given Class. The box-plots in the background are obtained using frequency to divide all words in four groups with the same number of words of the semantic Classes (first box-plot has words with lowest frequency and last box-plot has words with highest frequency). The classification based on Classes leads to a narrower distribution of 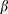
's inside Class and to a better discrimination between Classes.

In Fig. 2, we report our systematical analysis of the recurrence time distribution of all 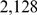 words that appeared more than ten thousand times in our database (for word-specific results see Table S1). We find a wide range of values for the burstiness parameter 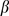 [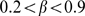, Fig. 2(a,b)] and the stretched exponential distribution describes well most of the words [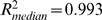, Fig. 2(c)]. The Class-specific results displayed in Fig. 2(a–c) show that words of all classes are accurately described by the same statistical model over a wide range of scales, a strong indication of a universal process governing word usage at these scales. Figure 2(b) also reveals a systematic dependence of 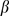 on the semantic Classes: burstiness increases (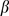 decreases) with decreasing semantic Class. This relation implies that words functioning unambiguously as Class 3 verbs should be less bursty than words of the same frequency functioning unambiguously as common nouns (Class 2). This prediction is confirmed by a paired comparison in our database: such verbs have a higher 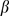 in 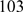 out of 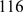 pairs of verbs and frequency-matched nouns (sign test, 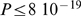). The relation applies even to morphologically related forms of the same word stem (see Text S1, *Lemmatization*): for 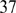 out of the 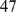 pairs of Class 3 adjectives and Class 4 adverbs in the database that are derived with *-ly*, such as *perfect, perfectly*, the adverbial form has a higher 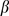 than the adjective form (sign test, 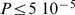). Figure 2(d) shows the dependence of 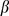 on inverse frequency 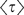. This figure may be compared to the TF-IDF (term frequency-inverse document frequency) method used for keyword identification [14], but it is computed from a single document (see also Refs. [16]–[18]). Figure 2(d) reveals that 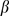 is correlated with 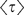 and that the Class ordering observed in Fig. 2(b) is valid at all 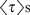
s. The detailed analysis in Fig. 2(e) demonstrates that semantic Class is more important than frequency as a predictor of burstiness (Class accounts for 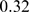 and log-frequency for 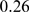 of the variance of 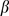, by the test proposed in Ref. [47]).

We are now in a position to discuss why burstiness depends on semantic Class. A straw man theory would seek to derive the burstiness of referring expressions directly from the burstiness of their referents. The limitations of such a theory are obvious: *Oxygen* is a very bursty word in our database (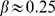) though oxygen is ubiquitous. A more careful observer would connect the burstiness of words to the human decisions to perform activities related to the words. For instance, the recurrence time between sending emails is known to approximately follow a power law [3], [5]. However, in our database the word *email* is significantly closer to the exponential (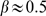) than a power law would predict (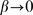). Indeed, a defining characteristic of human language is the ability to refer to entities and events that are not present in the immediate reality [48]. These nontrivial connections between language and the world are investigated in semantics. An insight on the problem of word usage can be obtained from Ref. [27], which establishes that the meaning and applicability of words with great *logicality* remains invariant under *permutations* of alternatives for the entities and relations specified in the constructions in which they appear. Here we consider permutability to be proportional to the semantic Classes of Table 1. As a long discourse unfolds exploring different constructions, we expect words with higher permutability (higher semantic Class) to be more homogeneously distributed throughout the discourse and therefore have higher 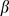 (be less bursty). Critical to this explanation is the fact that human language manipulates representations of abstract operators and mental states [49]. However, the overt statistics of recurrence times do not need to be learned word by word. It seems more likely that they are an epiphenomenal result of the differential contextualization of word meanings. The fact that the behavior of almost all words deviate from a Poisson process to at least some extent, indicates that the permutability and usage of almost all words are contextually restricted to some degree, whether by their intrinsic meaning or by their social connotations.

### Different Databases

**Figure 3:**
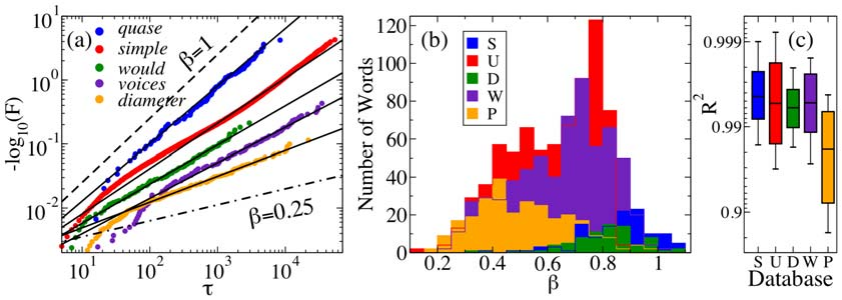
Stretched exponential recurrence time distributions observed in different databases. The databases consist of the documentary novel *Os Sertões* by Euclides da Cunha (S), in Portuguese (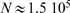); the USENET group comp.os.linux.misc (U) between Aug. 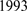 and Mar. 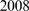 (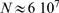); the three Obama-McCain debates of the 2008 United States presidential election (D) arranged in chronological order (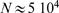); an English edition of the novel *War and Peace* by Leon Tolstoy (W) (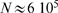); and the first English edition of Isaac Newton's *Principia* (P) (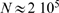). All words appearing more than 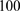 times were considered in S (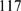 words), D (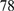 words), P (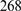 words), and W (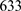 words), whereas in U all 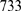 words appearing more than 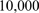 times were used (see Text S1, *Databases*). (a) Recurrence time distributions for the words *quase* in S (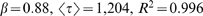), *simple* in U (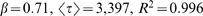), *would* in D (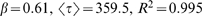), *voices* in W (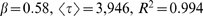), and *diameter* in P (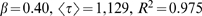). (b) Histograms of the fitted 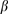 for all datasets. Due to sample size limits, the analysis into semantic Classes is not feasible for the smaller datasets. (c) Box-plots of the coefficient of determination 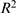 of the corresponding stretched exponential fit.

In Fig. 3 we verify our main results using databases of different sizes and characterized by different levels of formality. We analyzed a second example of a USENET group (U), a series of political debates (D), two novels (S,W), and a technical book (P) (for word-specific results see Table S1). The stretched exponential provides a close fit for frequent words in these datasets [Fig. 3(a,c)], and a wide and smoothly varying range of 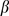
s is observed in each case [Fig. 3(b)]. The technical book exhibits lower 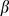 values, which can be attributed to the predominance of specific scientific terms. These datasets include examples of texts differing by almost four orders of magnitudes in size, generated by a single author (books), a few authors (debates) or a large number of authors (USENET), in writing and speech (e.g., books vs. debates), and in different languages (e.g., novels), indicating that the stretched exponential scaling is robust with regard to sample size, number of authors, language mode, and language.

### Conclusions

The quest for statistical laws in language has been driven both by applications in text mining and document retrieval, and by the desire for foundational understanding of humans as agents and participants in the world. Taking texts as examples of extended discourse, we combined these research agendas by showing that word meanings are directly related to their recurrence distributions via the permutability of concepts across discourse contexts. Our model for generating long-term recurrence patterns of words, a bag-of-words model with memory, is stationary and uniformly applicable to words of all parts of speech and semantic types. A word's position along the range in the memory parameter in the model, 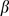, effectively captures its position in between a power-law and an exponential distribution, thus capturing its degree of contextual anchoring. Our results agree with Ref. [49] in emphasizing both the specific ability to learn abstract operators and the broader conceptual-intentional system as components in the human capability for language and in its use in the flow of discourse.

Analogies between communicative dynamics and social dynamics more generally are suggested by the recent documentation of heavy-tailed distributions in many other human driven activities [3], [5], [26]. They indicate that tracing linguistic activities in the ever larger digital databases of human communications can be a most promising tool for tracing human and social dynamics [22]. The stretched exponential form for recurrence distributions that derives from our model and the empirical finding it embodies are thus expected to also find applicability in other areas of human endeavor.

## Supporting Information

Text S1 Supplementary information on language analysis, statistical analysis, and counting models.
(0.55 MB PDF)

Table S1 Detailed information on the statistical analysis of all words that were studied (six databases).
(31.88 MB TAR)
