# Supplementary material for: Beyond Word Frequency: Bursts, Lulls, and Scaling in the Temporal Distributions of Words
Source: PLoS One. 2009 Nov 11;4(11):e7678. doi: 10.1371/journal.pone.0007678 (PMC2770836; doi:10.1371/journal.pone.0007678)
Supplement: Text S1 — Supplementary information on language analysis, statistical analysis, and counting models. (0.55 MB PDF) [file pone.0007678.s001.pdf]

Text S1: Supplementary Information

Eduardo G. Altmann,[1] Janet B. Pierrehumbert,[1,2] and Adilson E. Motter[1,3]
[1]*Northwestern Institute on Complex Systems, Northwestern University, Evanston, IL 60208, USA*
[2]*Department of Linguistics, Northwestern University, Evanston, IL 60208, USA*
[3]*Department of Physics and Astronomy, Northwestern University, Evanston, IL 60208, USA*

Contents

I. Language Analysis 1
A. Databases 1
B. Lemmatization 3
C. Coding of Semantic Types 4

II. Statistical Analysis 7
A. Fitting Procedures 7
B. Quality of Fit 8
C. Deviation from the Exponential Distribution 11
D. Correlation in $\{\tau_j\}$ 12
E. Independence of $\{\tau_j\}$ 13
F. Zipf-Alekseev Distribution 13

III. Counting Models 14
A. Counting Distribution 14
B. Hazard Function 15

References 17

I. LANGUAGE ANALYSIS

A. Databases

The primary database for this study is the USENET group talk.origins, a discussion group about evolution and creationism that started in 1986 and is still active. We have obtained all messages between 1986 and March 2008 through the Google Groups website. This database contains $N \approx 2\ 10^8$ words produced by fifty thousand users, and exemplifies the challenges and opportunities associated with very large data sets of spontaneous communication on the Internet. Our central findings were also validated with five additional text datasets illustrating other registers: the first English translation of Tolstoy's historical novel *War and Peace* (W) and the documentary novel *Os Sertões* by Euclides da Cunha (S) as examples of historical novels in two different languages (English and Portuguese); the transcripts of the three debates between Barack Obama and John McCain in the 2008 United States presidential election (D), available through http://www.debates.org, as a dialogue in a deliberate speech style; the first English translation of Isaac Newton's *Principia* (P), which provides an example of technical prose by a single author; and a second USENET group, comp.os.linux.misc (U), which is comprised of technical discussion in a community of experts.

The datasets W, S, and P are used in their full form, except for introductory and editorial notes. For dataset D, labels indicating the speakers were removed and the three debates were arranged in chronological order. The groups talk.origins and comp.os.linux.misc are more varied and noisy databases in which unequivocal examples of spam and long quotations in foreign languages were removed (see below).

As USENET discussion groups, talk.origins and comp.os.linux.misc consist of threads that in turn consist of posts, where a post is an individual communication by a participant. A post begins a new thread if the participant is not replying to any previous post. If a post replies to a previous post, it is appended to the corresponding thread. The thread lengths in talk.origins, for example, span from 1 to $5,248$ posts. The number of words per post and per thread is distributed approximately log-normally, as shown in Figure 1.

Each discussion group was collated into a long data stream by referring to the time stamps on the posts. Threads consisting of only one post were eliminated in order to eliminate spams and other messages extraneous to the topic

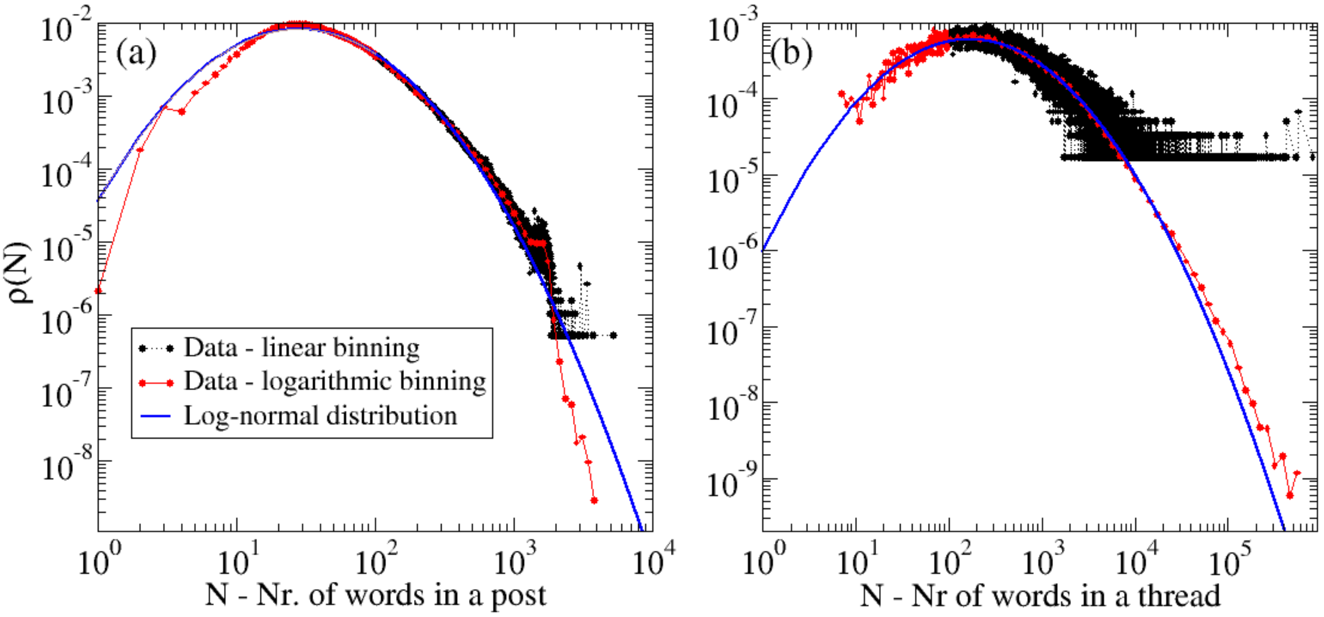

FIG. 1: Distribution $\rho(N)$ of the number N of words per (a) post and (b) threads in the USENET group talk.origins. The solid blue line corresponds to the log-normal distribution with mean and standard deviation of $\rho(N)$.

of discussion of the group. In the case of talk.origins, extremely large outliers in word recurrence patterns were used to identify and eliminate about 100 long threads written exclusively in languages different from English. (Quotes and citations in foreign languages within posts were kept.) Threads consisting of more than one post were collated one after the other by the order of the initial post. Posts within a thread were collated in temporal order. This collation scheme in which threads are organized chronologically (*chronological threads* in Fig. 2) is not the only logical possibility. The robustness of the results presented in the main text was also evaluated against alternative methods, and essentially similar results are found if: (i) only recurrence between words inside a thread is considered (*intra-threads* in Fig. 2); and (ii) threads are randomly permuted with each other (*random threads* in Fig. 2). Random permutation of the posts (*random posts* in Fig. 2) substantially changes the results, introducing an exponential tail to $F(\tau)$, which substantially reduces burstiness. These results are illustrated in Figure 2 for the word *evolution*. They show that the recurrence distributions for individual frequent words are dominated by the within-thread statistics and that the randomization of posts averages out the bursts of interests in a topic within a thread.

The main properties of the databases used are summarized in Table I.

**Dataset**	**Authors**	**Translator**	**Language**	**Year**	**Size in words**	**Nr. words**
War and Peace	L. Tolstoy	L. S. Maude	English	1865 – 1869	564, 295	633
Os Sertões	E. da Cunha		Portuguese	1902	153, 759	117
US election debates	Obama, McCain, interviewers		English	2008	47, 099	78
Principia	I. Newton	A. Motte	English	1687	203, 442	267
Usenet: comp.os.linux.misc	128, 902 users		English	1992 – 2008	$6\ 10^7$	733
Usenet: talk.origins	47, 608 users		English	1986 – 2008	$2\ 10^8$	2, 128

TABLE I: Information on the databases used. The column Nr. of Words indicates the number of word types studied, which in the USENET groups consists of all words appearing more than 10, 000 times and in the remaining databases of all words appearing more than 100 times.

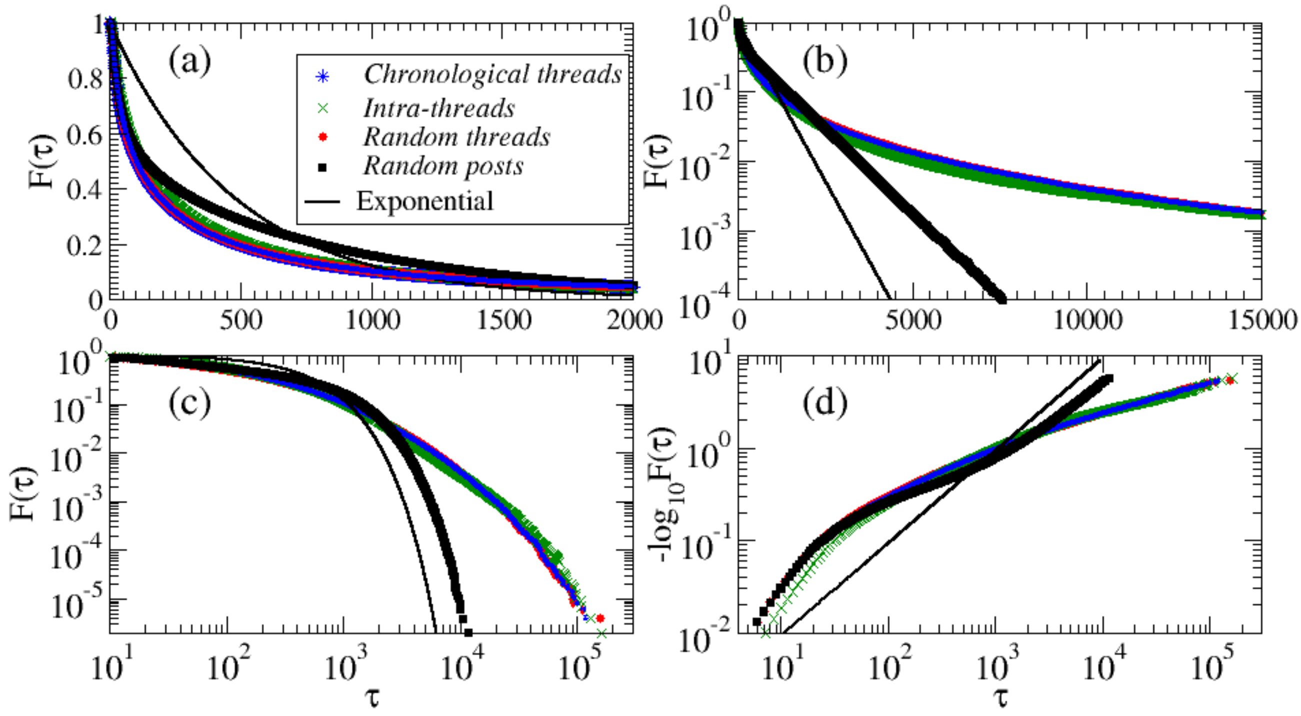

FIG. 2: Different representations of the cumulative recurrence time distribution $F(\tau)$ for the word *evolution* ($\langle\tau\rangle = 476$) in the USENET group talk.origins. The different panels correspond to (a) a linear scale, (b) a linear-log scale, (c) a log-log scale, and (d) a scale in which the stretched exponential distribution is a straight line. The different distributions correspond to the different collation schemes of the database, as indicated in the legend. The solid line corresponds to the exponential distribution with exponent $\mu = 1/\langle\tau\rangle$. To be compared with the other distributions, the *intra-threads* distribution was re-scaled by $\langle\tau\rangle/\langle\tau\rangle_{it}$, where $\langle\tau\rangle_{it} = 330$ is the average of τ within the threads. Overlapping results are found for *chronological threads*, *intra-threads*, and *random threads*. The *random posts* case follows the other distributions only for short times, after which an exponential decay arises.

B. Lemmatization

All datasets contain numerous orthographic strings related to each other as words that share the same stem. For example, the talk.origins group includes *natural*, *naturalism*, *naturalistic* and *naturally* as well as *create*, *created*, *creator*, *creature*, *creationist*, and *creationists*. Such families of word forms raise the question of whether to lemmatize (combine tokens of related words under the rubric of a single stem), or to work with each lexeme (word form) separately. Lemmatizing provides larger sample sizes, and in pilot work we have observed that the recurrence time distribution systematically deviates from an exponential distribution under strong lemmatization. However, it creates the risk of conflating cases that would reveal important differences. Given the large sample size available in the present study, we have accordingly elected to proceed conservatively with minimal lemmatization.

Words are taken to be a string of alphabetic characters separated from other strings by white space. In addition to space, tab, and newline, the characters semicolon (;), underscore (_), as well as all punctuation marks (.,!,?,:,,) are treated as white space. However, apostrophe (') and hyphen (-) were not. This means that web and email addresses are broken up into their component parts, whereas expressions such as *weren't* are *e-mail* are treated as words.

Capitalization was removed, so that instances of the same word in sentence-initial and sentence-medial position would be tabulated together. Single letter strings, apart from the personal pronoun *I* and the article *a*, were removed. Strings consisting entirely or partly of non-alphabetic characters (e.g., #,@,%,&,*) or numerals, such as *2000* and *2fer* were also eliminated. This eliminates time stamps, IP addresses, signature symbols, and other input extraneous to the present purpose. It also means that results for numbers cannot be viewed as reliable, since posters may have vacillated between typing numbers and spelling them out in the bodies of their posts. In the non-USENET databases (apart from P), numbers were kept. No further lemmatization of purely alphabetic strings was imposed, with the

result that all of the words cited just above are treated as distinct.

The decision to keep morphologically related words distinct is rooted in the psycholinguistic literature as well as in the findings of this study. The focus of the study is words occurring more than $10,000$ times in USENET groups, and 100 or more times in W, S, D, and P (100 tokens is a minimum sample for reliable fitting, as shown in Sec. II A). These words are all quite frequent and familiar to the respective audiences. Common affixes attested in the word list for talk.origins include *-s* (ambiguous between the plural and the third person singular verbal ending, cf. *forces of good*), *-ed* (ambiguous between personal past tense forms and the past participle, cf. *received wisdom*), *-ing* (ambiguous between the present participle and the adjectival suffix, cf. *fitting tribute*), the adverbial suffix *-ly*, and the nominal suffixes *-ion* and *-ity*. In the linguistic literature on morphology, such affixes are conventionally divided into inflectional affixes and derivational affixes. Derivational affixes relate words with different meanings and often different parts of speech, whereas inflectional affixes represent obligatory syntactic markings.

Lemmatization for derivational morphology would be problematic for this dataset, as it includes many examples of spurious or semantically opaque derivation. *Bother* should not be derived from *both*, nor *billion* from *bill*. Words pairs in the dataset that exhibit historical connections and partial semantic relatedness include *background/ground*, *hardly/hard* and *university/universe*. The decomposability of such words in the minds of speakers cannot be taken for granted. Though Ref. [1] argues from masked priming experiments that even non-transparent pairs, such as *hardly/hard*, and pseudo-derived pairs, such as *corner/corn* are decomposed during language comprehension, Refs. [2, 3] provide evidence that the extent and reliability of such decomposition is a complex function of word semantics and phonological structure as well as the relative frequency of the stem and the base. Derivational affixes map stems onto words of a different syntactic and semantic class, creating the potential for the complex forms to differ in their β values from the base forms. The results indeed instantiate this potential. The verb *create* (Class 3) has a higher fitted β (0.54) than the derived noun *creation* (0.45) (Class 2). The data set contains 47 pairs of adjectives and adverbs transparently derived with *-ly*, such as *perfect/perfectly* and *relative/relatively*. For 37 of these pairs, the adverbial form has a higher β than the adjective, as would be predicted from the fact that the base adjectives are mainly Class 3 whereas the adverbs are mainly Class 4.

For inflectional morphology, complex words as frequent as those we have studied are the focus of a heated debate in the psycholinguistic literature. Marcus *et al.* [4] and Pinker and Ullman [5] advocate a model in which regularly derived plurals and past tense forms are generated by rule during sentence production. Only irregular forms, such as *children* and *went* are listed in the lexicon in this model. However, other authors provide evidence from language acquisition and processing that highly frequent regular forms are memorized and stored, providing the sample from which broad regularities are induced [6–8]. Storage of the complex forms provides the potential for them to acquire idiosyncratic properties, such as semantically unpredictable meanings or unpredictable word frequencies. A number of plurals in our dataset are more frequent than their stems (e.g., *headers*, *hours*, *odds*, *parents*), an indication that they may be processed holistically by speakers and listeners [3].

To summarize, the dataset contains numerous examples for which the lemmatization of superficially related word pairs would be disputable in the light of current psycholinguistic research. Although individual posters may well have unified lexical entries for some word sets (such as nouns and their productively formed plurals), these word sets are not necessarily consistent amongst posters, and there is no objective or replicable way to determine which they are. Further, the fine-grained analysis permitted by working with individual lexemes rather than with lemmas leads to suggestive differences between related lexemes of different syntactic and semantic classes. The minimal lemmatization used here is thus the conservative choice. It is noteworthy that the same choice as here is also made in a recent major psycholinguistic study of the effects of word repetition on phonetic durations [9]. This choice is also conservative in the sense that failure to lemmatize word sets that are unified by strong cognitive relationships has the effect (on the average) of eliminating word tokens at random for the stem, introducing noise into the estimate of β. This means that the statistical reliability of patterns in β is being conservatively estimated through the use of minimal lemmatization.

C. Coding of Semantic Types

We focus on semantic type rather than syntactic part of speech because we are interested in long time scales at which syntactic constraints are not defined and the intrinsic meanings of the words supports a direct connection to the degree of permutation invariance [10, 11].

Grouping of words into the semantic types of Table 1 (main text) and Fig. 2 (main text) reflects a philosophy of using light coding in the interests of conservative and replicable claims. This intuitive description of the ladder of abstraction in semantics is reconstructed more technically in formal semantics by Refs. [10, 12, 13] and others, building on the foundational results in logic and set theory advanced in Ref. [14]. The theory can be developed either extensionally (relating words and sentences to objects and states of affairs in the world), or intensionally (relating words and sentences to concepts in the mind, which can include alternative possible worlds). Intensional semantics

is needed to make sense of the truth conditions for sentences such as *Asimov believed that humanoid robots could colonize distant galaxies.* However, we follow Refs. [10, 13] in using extensionalized notation for tutorial purposes.

The two primitive types are entities e (exemplified by proper nouns) and truth values t [10, 13]. Just as proper nouns refer to entities in the world, declarative sentences refer to truth values (any given declarative sentence is either true or false). Simple verbs such as *dies*, are more abstract, as they are functions from entities to truth values, $< e, t >$. This is because they take arguments, namely the subject (and for transitive verbs also the object). By instantiating the subject argument of *dies* with an entity, such as *Darwin*, we obtain a sentence, such as *Darwin dies*, which is either true or false. The same degree of abstraction (Class 2) is shared by common nouns, such as *author*, because common nouns are used as predicates, e.g. *Darwin is an author.* It is also shared by simple intersective adjectives, such as *red.*

The ladder of abstraction can be recursively extended by considering, for each word, the domain and image of the mapping that is implicitly associated with it by virtue of its meaning. For example, essentially quantificational nouns, such as *everyone*, are of type $<< e, t >, t >$ (Class 3) because they are characteristic functions of sets of properties of entities. Non-intersective adjectives such as *small* and *simple* share the same level of abstractness, because their interpretation is a implicit scalar function of a comparison set; a small building is bigger than a large mouse, and a simple meal is more complex than a complex molecule. This leads to the specific type $<< e, t >, < e, t >>$. Intensional verbs such as *try, discover, believe, learn,* whose meanings intrinsically involve mental states, are also coded as Class 3. Modals and degree adverbs are of even higher type (coded as Class 4), in view of typical expressions such as *might believe* and *perfectly simple* in which they modify Class 3 words.

A given word can have different types in different contexts because languages have productive processes of type shifting. *Disney* is originally a proper name for a person, of type e, and retains this type as a proper name for a corporation. However, it can be readily understood as a modifier or predicate, type $< e, t >$, in the sentence *I don't want to Disney ... we do everything Disney.* That is, the listener readily reinterprets the entity as the properties that are typical of that entity. In Ref. [12], the lexical type of a word is taken to be the highest type in which it occurs, leading to the conclusion that all nouns are generalized quantifiers, like the term *everyone.* However many recent publications, including Ref. [13], take the lowest type as basic, and we follow this scholarly trend. Since type raising is far more productive than type lowering, classifying words by their minimal type leads to sharper distinctions amongst the various sets of words.

Reference [13] presents a type ladder with seven levels, and indicates that it can be indefinitely extended. A compressed scale of types (with only four levels of abstraction) is used in order to create large enough sample sizes for high types, and to obviate the need to take stands on questions under active dispute in the formal semantics literature. Examples of the four classes are as follows:

- Class 1: Proper names. Examples: *Africa, Satan, Tim, Dejanews.*
- Class 2: Common nouns, prepositions, simple intersective adjectives, and extensional verbs. Examples: *life, man, religion, system, of, in, against, among, blue, talk, snip, come.*
- Class 3: Non-intersective adjectives, intensional verbs, temporal and locative adverbs, present participles of Class 2 and Class 3 verbs, and essentially quantificational nouns. Examples: *small, evolutionary, forty, certain, various, believe, explain, expect, ask, seldom, always, currently, earlier, somewhere, anybody, everything, someone, whatsoever.*
- Class 4: Determiners, subordinators, auxiliaries, raising and control verbs, and modal adverbs. Examples: *a, the, although, but, hence, may, can, did, let, seem, definitely, equally, supposedly.*

Type coding was done blind by the second author on words in isolation, without information about their frequency, β values, or context. The browser interface for Ref. [15], http://wordnetweb.princeton.edu/perl/webwn, was used to determine the range of parts of speech in common use for a word whose analysis was uncertain, with the posted examples then used to adjudicate the semantic class. The coding was validated by a random set of 200 words coded by a second coder – a computational and experimental linguist who was independently trained at other universities and was ignorant of the research goals of the research project. The second coder (Hannah Rohde) agreed with the second author to within one type in 99% of the cases, and 80% to the exact type. The majority of disagreements concerned ambiguity between Class 2 and Class 3.

Five percent of the words were classified as non-codable. These include subject-verb contractions (e.g., *I'm*), interjections such as *yep*, titles (e.g., *Mr.*), and routing codes and filename extensions (e.g., *http*, *uk*, *jpg*). Similarly, forms of *be* were not classified due to their extremely wide range of uses, from the proper noun *Supreme Being* to use as a copula or auxiliary (Class 4). Personal pronouns, including their possessive and reflexive forms, were also classified as non-codable because their semantic type is under dispute in the research literature. Some authors, such as von Fintel [11], treat all pronouns as operators. This conclusion is strongly supported for reflexive pronouns

(see Ref. [10]). However, discussion in Refs. [16–18] suggests that nominative and accusative personal pronouns (*he, him, they, them, etc.*) are entities instead. A post-hoc analysis of our data supports this second view, as reflexive pronouns have high β for their frequency, whereas nominative and accusative personal pronouns have low β for their frequency.

As shown in Figure 2(b,e) (main text), there was substantial within-class variation in β values for each of the four classes. Much of this variation is *bona fide*, relating to other influences besides semantic class on the permutability of specific words, including frequency as discussed in the main text. We quantify the relative importance of semantic class and frequency by measuring the amount of the variability of β explained by these two variables ($\langle\tau\rangle$ and Class). Following Ref. [19], we calculate R^2 of the linear fit of β for Class and $\log(\langle\tau\rangle)$, before and after subtracting the effect of the other variable (we assume a linear relation between classes $1-4$ and their effect on β). We obtain that semantic class accounts for 0.32 and $\log(\langle\tau\rangle)$ for 0.26 of the variance of β (the significance levels of all fits are $P < 2.2\ 10^{-16}$. A linear fit of both variables simultaneously yields $R^2 = 0.51$). Some of the remaining variability is probably due to noise in the tabulation and coding. First, the tabulation in some cases conflates semantically unrelated homonyms, such as the noun *type* and the verb *type*. Bell *et al.* [9] note and accept the same problem in their analysis of a much smaller (13, 190-word) corpus. Second, the extreme productivity of zero-derivation in English means that many words have related meanings falling in different syntactic and semantic classes. For example WordNet lists *light* as a noun (*a bright light*), as an adjective (*light blue*), and as a verb (*light the way*). The lowest class, and the one recorded, is Class 2. Though the statistically dominant usage of a word might lie in a higher class, it is not possible to determine the word-by-word usage statistics on such a large dataset of colloquial language without prohibitively laborious coding; automatic part-of-speech taggers have only reached high levels of reliability for formal writing. This consideration particularly contributes to the variance in Class 2, given the extremely large number of Class 3 verbs and adjectives that can also occur as common nouns. Third, we can consider what the result would be if the cognitive system actually distinguished homographs of different semantic classes that we have conflated. In this case, the observed signal would be a random mixture of signals with two different underlying β characteristics. Just as for the complementary case, that of failing to lemmatize words of the same class that are cognitively related, the effect would be to increase the noise in the estimation, jeopardizing the statistical power needed to establish contrasts. In summary, the coding standards used are conservative, and stronger effects might emerge if more in-depth coding were possible.

Prior work on burstiness in the document classification literature has mainly referred to syntactic categories rather than semantic types. Notably, work using the the inverse document frequency (IDF) measure — defined as the the negative log inverse of the proportion of documents in a set of documents that contain a given keyword [20] — has reported a three-way distinction between *function words*, *content words*, and *proper nouns*. [21–24]. In syntactic and psycholinguistic theory, function words (generally taken to include articles, subordinators, personal pronouns, and prepositions) are supplied by the syntax as parts of syntactic frames. Content words such as nouns, verbs, adjectives, and adverbs are inserted from the lexicon after the syntactic frame is constructed (see review in Ref. [9]). Proper names are also a syntactically coherent class because they act as complete noun phrases by themselves and do not accept determiners and modifiers. The counting distribution of function words lies close to the exponential, that of proper nouns is far off the exponential (making proper nouns a generally useful class of keywords for document retrieval), and content words are spread out in between.

Syntactic parts of speech are partially correlated with semantic type, and indeed semantic type theory arose in the context of efforts to model the regular relationships between syntax and semantics that make it possible for novel complex sentences to convey novel complex meanings (cf. Refs. [10–13, 25–27]). This partial correlation means that members of some syntactic types all belong to a single semantic type, whereas the members of others are split between two semantic or more types because of critical meaning components. Notably, all proper names are Class 1. Most common nouns are Class 2, but common nouns with an essentially quantificational meaning are Class 3. Simple extensional adjectives are Class 2 and non-intersective adjectives (whose meaning depends significantly on the noun they modify) are Class 3. Amongst verbs, simple verbs are are Class 2, intensional verbs are Class 3, and verbs such as *seem* (so-called raising and control verbs) are Class 4. Amongst adverbs, temporal and locative adverbs are Class 3 and modal adverbs are Class 4. All determiners, subordinators, and auxiliaries are Class 4. As a result, we replicate previous observations about the burstiness of proper nouns, determiners, subordinators and auxiliaries, while providing an exegesis of part of the within-category variation for nouns, verbs, adjectives, and adverbs.

As noted above, coding words by their lowest semantic type also conflates tokens that occupy two or more different syntactic roles in different sentences. Studies reporting burstiness by part of speech [28] show high within-category variability. In addition to reflecting semantic type differences, this variability may arise in part from noisiness in the syntactic coding due to the large number of noun-verb, verb-adjective, noun-adjective and preposition-adverb homophones in English. In particular, common nouns and simple verbs can be surprisingly hard to distinguish in colloquial language such as USENET language, due to the free interconversion of these parts of speech. The overall difference in behavior between nouns and verbs thus arises from the fact that a disproportionate share of verbs are intensional (intrinsically involve mental states), and therefore fall in Class 3. A paired comparison between intensional

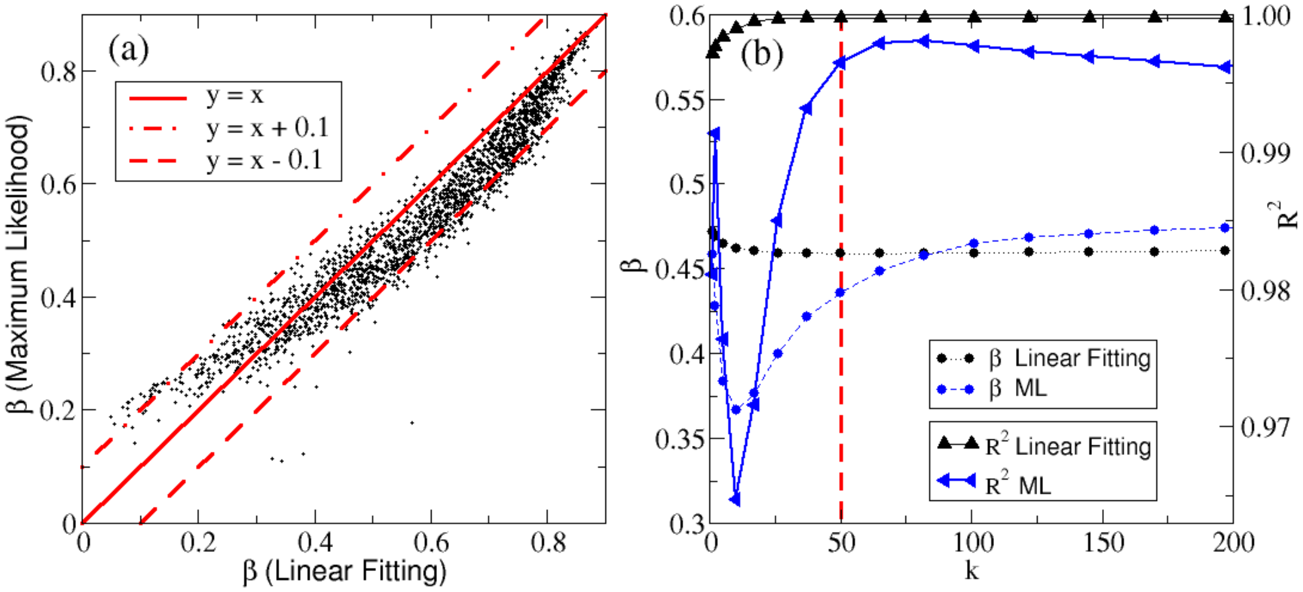

FIG. 3: Comparison between two different fitting procedures to determine β: the linear fitting described in the text and the maximum likelihood (ML) method. (a) Scatter plot of the 2, 128 words analyzed in the USENET group talk.origins for the short-time cut-off of $k = 50$. (b) Dependence of the fitted β (r.h.s. axis, circles) and of the coefficient of determination R^2 (l.h.s. axis, triangles) as a function of the short-time cut-off k for the word *theory* in the talk.origins group.

verbs and frequency-controlled common nouns is reported in the main text. For further discussion on the technical relationship of syntactic to semantic types, we signal Refs. [25–27], all of which propose formal frameworks that apply type theory in the context of language generation and parsing.

Our analysis can decorrelate semantic type and syntactic type because these operate at different time scales. Tabulation of the counting distribution for documents effectively averages the short-term behavior of words, as determined by the syntax and local discourse [29], together with the medium-term behavior (the length of the documents). Examination of the recurrence times out to long time scales permits us to separate the effects. Notably, the syntactic category of function words does not prove to be incisive for longer scales. There appear to be important distinctions within the function words (e.g., the difference between reexive and non-reexive pronouns discussed above). Further, high-type content words, such as modal adverbs, display very similar behavior to high-type function words.

II. STATISTICAL ANALYSIS

A. Fitting Procedures

In order to automatically obtain the burstiness exponent β of each word, we first transform the empirical cumulative distribution $F(\tau)$ into a representation in which a stretched exponential distribution corresponds to a straight line. This is done by plotting $-\log(F(\tau))$ against τ in log-log scale [see Figure 1(b) of the main text]. Next, the interval $\Delta\tau$ where the fitting will be performed is defined by (i) ignoring the distribution for $\tau \leq k = 50$ and (ii) removing the 20 largest τ of each distribution (exclusively in the case of the USENET groups). The reason for removing the latter is that these long times τ tend to correspond to spams and other non-English posts that escaped our first filter. For the non-Usenet databases point (ii) above was omitted. The fitting interval $\Delta\tau$ obtained through this procedure was larger than three decades in τ for 83% of the words and larger than two decades for 98.6% of the words considered in the USENET group talk.origins. We then choose points equally spaced in the logarithmic τ axis (logarithmic binning for the cumulative distribution). All results in the main text correspond to a straight line fitted to these points.

We have compared this fitting procedure to the maximum likelihood fitting [30] of the cumulative distribution $F(\tau)$. Figure 3(a) shows that similar values of β are obtained for both procedures. We chose to use the straight line fitting in our analysis because the maximum likelihood method has been found to be very sensitive to the choice of the

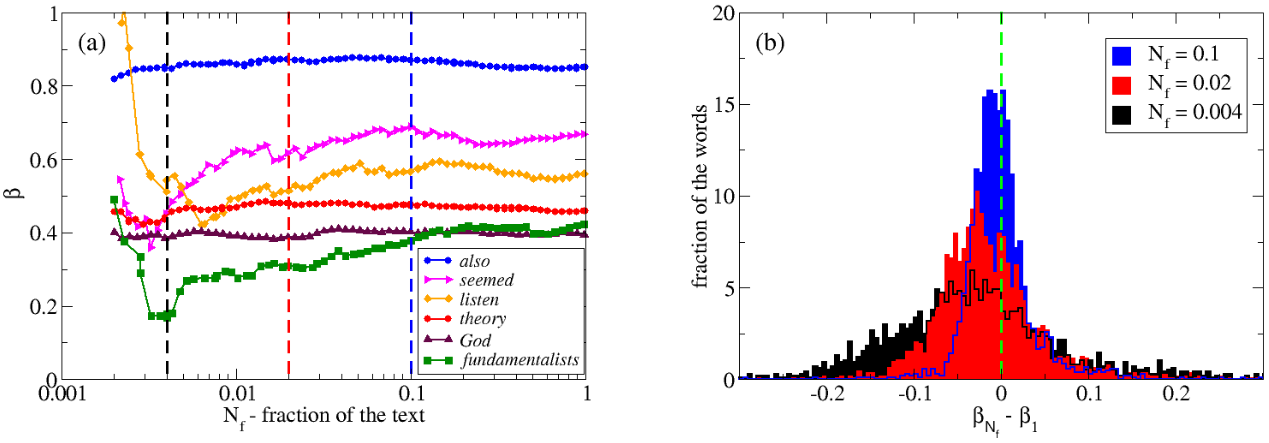

FIG. 4: Dependence of β on the size of the document measured by the number of words. The value of β was calculated using an increasing fraction N_f of the USENET group talk.origins (total size 2 10^8 words). (a) Detailed analysis of the six words used in the main text. (b) Distribution of the distance between β_{N_f}, obtained using three different fractions of the text, $N_f = 0.005, 0.02$, and 0.1, and β_1, obtained using the full text ($N_f = 1$). All words appearing more than 10,000 in the talk.origins database were considered.

cut-off k, leading to substantially worse fittings (for $k = 50$ in the talk.origins dataset, mean R^2 is 0.829 for ML and 0.983 for our linear fitting, where R^2 is the quality of fit defined below). The sensitivity of both methods to the cut-off k is illustrated in Figure 3(b) for the word *theory*.

For both procedures, we have fitted the two parameters a and β ignoring the relation between them determined by imposing that $\langle\tau\rangle = 1/\nu$ [see Eq. (2) of the main text]. The reason is that short-time deviations may affect $\langle\tau\rangle$ and therefore change the relation between a and β [see inset in Figure 1(a) of the main text]. For words exhibiting good scaling behavior over several decades, the value of the fitted a is very close to the one obtained from β and $\langle\tau\rangle = 1/\nu$. For the examples presented in Figure 1 of the main text, the corresponding results are essentially indistinguishable.

The dependence of the value of β (obtained through the linear fitting) on the size of the database is verified in Fig. 4. The results show that the value of β obtained using a finite sample of the text both over-estimates and under-estimates (depending on the word) the value of β obtained using the full text. There is a small bias towards smaller values of β. The convergence within a range of $\beta \pm 0.1$ occurs typically already for around 1% of the full text (2 10^6 words) and convergence is faster for frequent words. This corroborates the assumption that the usage of words can be modeled by a stationary process. We notice also that the less frequent words from the USENET database we analyzed appear on average 100 times in 1% of the text. This suggest that analysis of words with frequencies below the limit of 100 appearances might depend strongly on the database size, justifying our choice for the smaller databases (see Table I).

B. Quality of Fit

The quality of fitting was validated independently of the fitting method described in Sec. II A. In particular, we have discarded points neither for short times nor for long times, and we have used the cumulative distribution $F(\tau)$ in linear scale. As a measure of the quality of fitting, we adopted the coefficient of determination R^2 defined as

$$R^2 = 1 - \frac{S_{err}}{S_{tot}} \equiv 1 - \frac{\sum_j [F(\tau_j) - F_\beta(\tau_j)]^2}{\sum_i [F(\tau_i) - \langle F\rangle]^2}, \tag{1}$$

where F_β is the fitted distribution, $\langle F\rangle$ is the mean value of $F(\tau_j)$, and the sums run over all observed intervals τ_j.

The coefficient R^2 is a standard measure of quality of fit for models fitted by linear regression. Standardly, it ranges in value from 0 (the model explains none of the variation around the mean) to 1 (the model explains all of the variation around the mean). Since we compute R^2 over a larger set of data points than were used to establish the fit, there are a few cases in which the value is slightly negative, i.e. the quality of fit is worse than the mean; these are

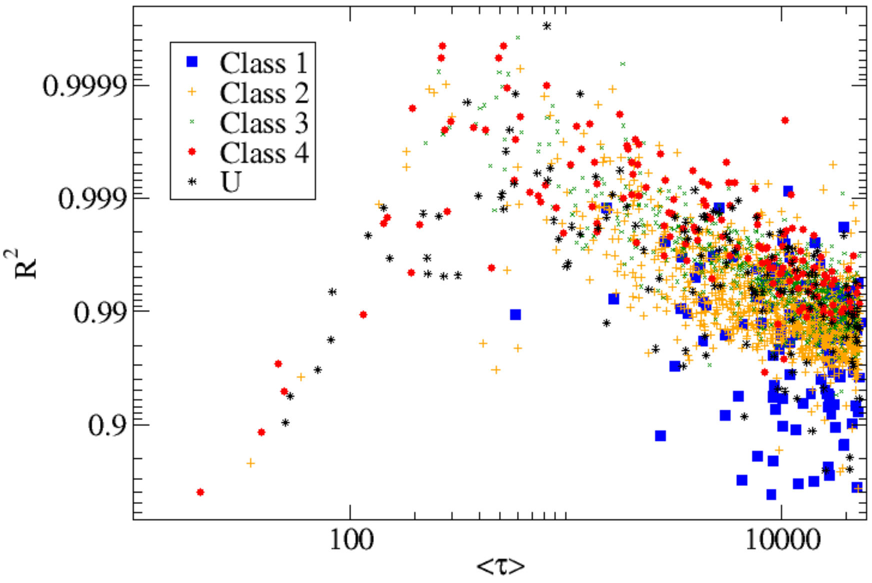

FIG. 5: Quality of fit R^2 and inverse frequency $\langle\tau\rangle$ for the $2,128$ words in the USENET group talk.origins. Different symbols and colors denote the different classes of words, as indicated in the legend.

confined to the words *cylinder* and *inches* in P and the word *Pavlovna* in W. Overall, the quality of fit is exceptionally high.

Figure 5 shows a scatter plot of the value of R^2 and the inverse frequency $\langle\tau\rangle$ of all words in talk.origins. We see that the quality of fitting increases for decreasing $\langle\tau\rangle$ (increasing ν) up to ≈ 800. This can be partially explained by the larger number of observations used to built $F(\tau)$ of these words. However, words with low $\langle\tau\rangle$ (high frequency) become very sensitive to spams and other sources of noise in the database. This partially explains the smaller values of R^2 for small $\langle\tau\rangle$ observed in Figure 5. Plots of $F(\tau)$ and the corresponding values of β, R^2, and $\langle\tau\rangle$ for all words in all databases used in this study are available in Table S1.

The residuals of all fits were computed by measuring the distance between the straight-line fit and the empirical points, in the scale where the stretched exponential distribution is a straight line [i.e., $\log_{10}(-\log_{10} F(\tau))$ x $\log_{10}(\tau)$]. In order to combine the residuals obtained for different words, the x-axis of each word was re-scaled appropriately (divided by $\langle\tau\rangle$). The combined residuals are presented in Fig. 6. The deviations for small $\tau/\langle\tau\rangle$ are related to $\tau < 50$. For long times the residuals are smaller and, for the four top panels, we find that the residuals are almost symmetrically distributed around zero. For the two lower panels the values of the residuals are smaller, what indicates a better agreement with the data. However, there is a clear oscillation around zero observed in the bottom two panels of Fig. 6. This shows that the fluctuation of different words around the fitted stretched exponential show a systematic deviation.

The systematic deviations reported above are apparent only for the two USENET databases considered. It is therefore natural to question whether they are related to the properties of these databases. One possible reason is that continuity of the text is affected by the post and thread structure. As argued in Sec. I A, the cohesion of the text is only weakly preserved for scales longer than the thread size. For times longer than the size of threads the distribution is expected to decay faster, approaching an exponential decay (similar to what was observed in Fig. 2 when the posts were shuffled). This would affect all words and be more apparent in less frequent words, for which $\langle\tau\rangle$ is larger and becomes comparable to the length of the thread. This is consistent with our observations.

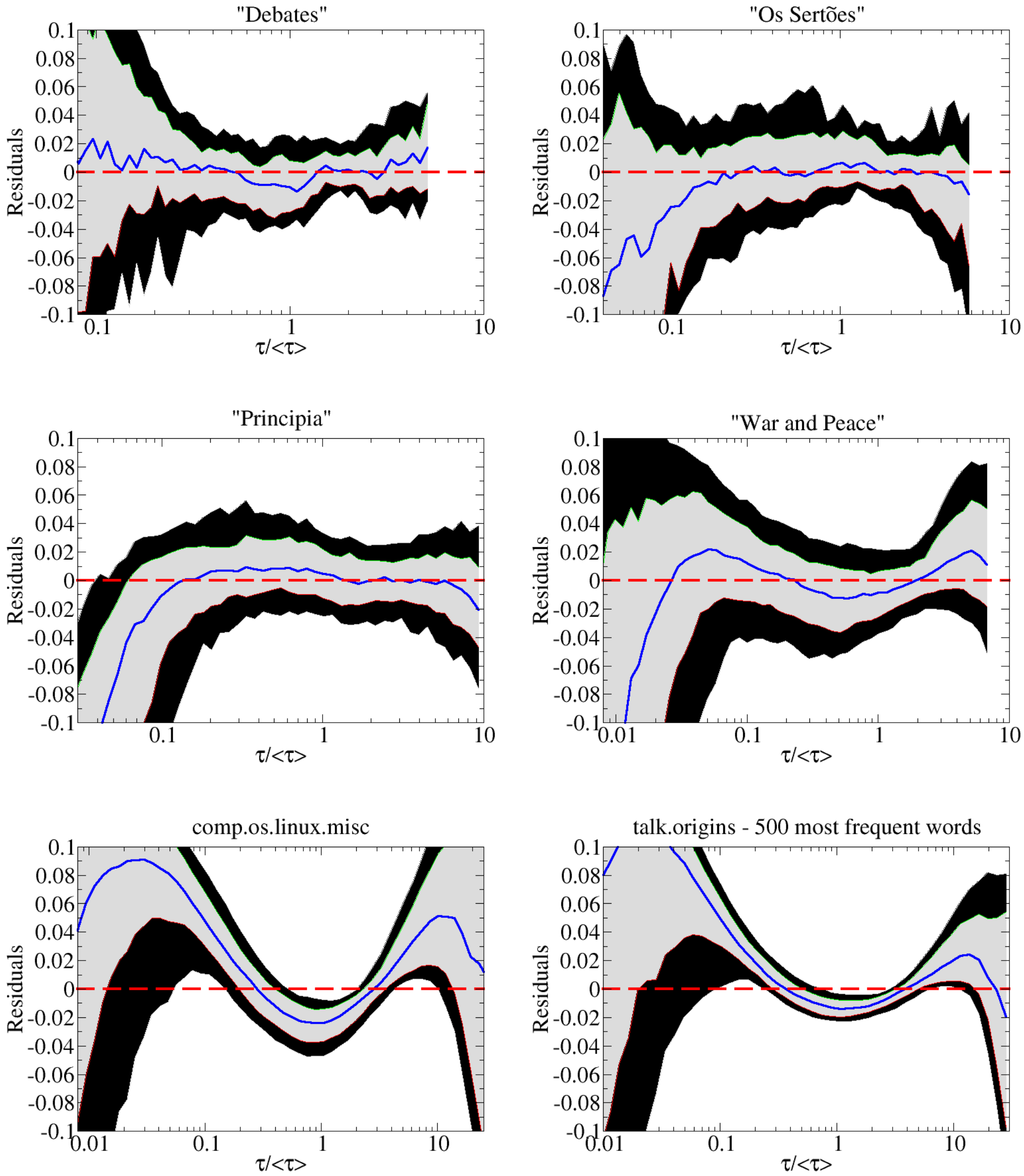

FIG. 6: Residuals of the stretched exponential distribution, measured as the distance between the empirical distribution and the straight line fit. Results of different words are combined in each of the panels. The solid line in the center is the median of the distribution across words at each $\tau/\langle\tau\rangle$, the border of the gray region corresponds to the 2nd and 6th octiles, and the border of the black region corresponds to the 1st and 7th octiles. Each panel show the results for one of the 6 databases reported in Table I, as indicated on top of each panel. For the talk.origins database the 500 most frequent words are shown. By restricting the analysis to the most frequent words we ensure that the systematic deviations observed occur also for the words with lowest R^2 (see Fig. 5). Plots including low frequency words show similar results, but with an even stronger systematic deviation. For the other databases, all words described in Table I were used.

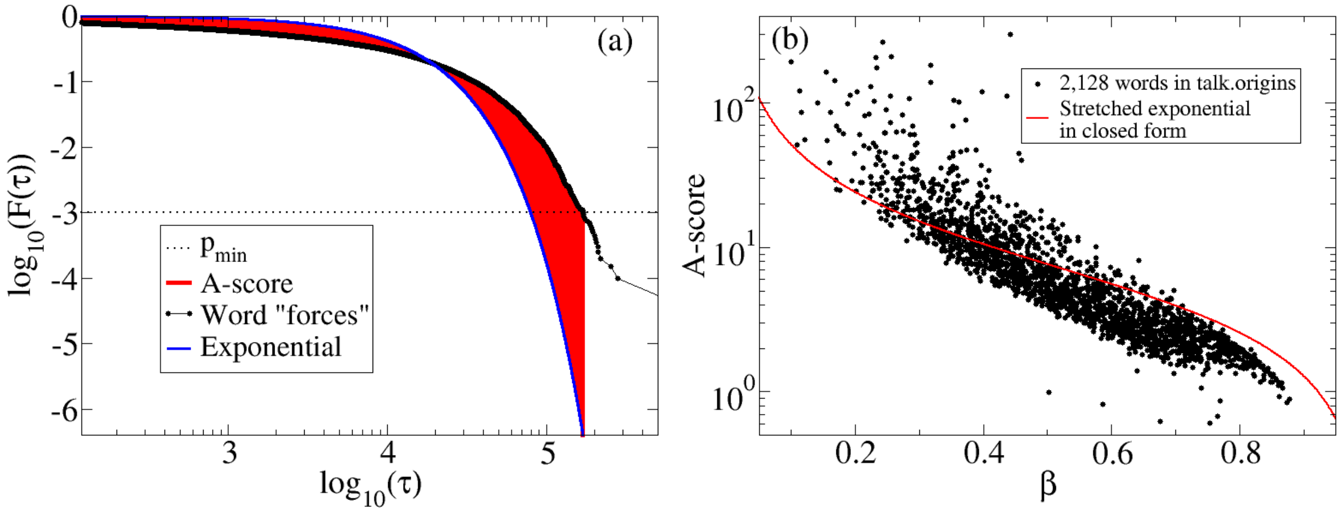

FIG. 7: Exponent β as a measure of the deviation from the exponential distribution with exponent $\mu = 1/\langle\tau\rangle$. (a) Schematic representation of the A-score as the area between the exponential prediction $F_P(\tau)$ (blue line) and the empirical cumulative recurrence distribution $F(\tau)$ (black circles) in a double logarithmic scale. (b) Scatter plot of the fitted β and the A-score for the $2,128$ words in the Usenet group talk.origins. The red line corresponds to the relation between the A-score and β for the closed form of the stretched exponential distribution (with $a = a_\beta$ determined by imposing $\langle\tau\rangle = 1/nu$). A threshold of $p_{min} = -3$ is used for all the words.

C. Deviation from the Exponential Distribution

In the main text we argue that the relation between the classes of words and the exponent β of the fitted stretched exponential distribution can be interpreted in terms of permutation invariance. This assumes that β is a valid measure of the deviation of the recurrence time distribution of each word from the corresponding exponential distribution (with exponent $\mu = \nu = 1/\langle\tau\rangle$). Here we provide additional support for this assumption. First we define the deviation between the two distributions as the area between the two curves, as indicated in Figure 7(a). We refer to this measure as the A-score of the word. Figure 7(b) shows a scatter plot of the A-score and the exponent β for all words in the Usenet group talk.origins. The tendency of decreasing A-score for increasing β supports our assumption that β measures the deviation from the exponential distribution.

We have compared the quality of the fit of the stretched exponential and of the exponential distributions. We considered the exponential function $F_{exp}(\tau) = \alpha_1 \exp(-\alpha_2 \tau)$ and we determine the two free parameters (α_1, α_2) through a procedure equivalent to the one used to obtain the two free parameters (a, β) of the stretched exponential distribution, described in Sec. II A. More precisely, (α_1, α_2) were obtained through a least squares fitting of a straight line to the empirical distribution $\log(F(\tau)) \times \tau$, after discarding short times $\tau < 50$ and the 10 longest times. We then computed the values of R^2 of the exponential fit, as described in Sec. II B, and compared to the stretched exponential fit for the words in the talk.origins database. We found that in $2,126$ out of $2,128$ words the stretched exponential provided a better fit than the exponential (larger R^2). For $2,090$ words the difference in R^2 was of at least 0.01. The median R^2 for the exponential distribution was $R^2_{median} = 0.907$, which should be compared to $R^2_{median} = 0.993$ for the stretched exponential distribution.

We found equivalent results using the χ^2 as a quantifier of the quality of fit [31]. In this case we used the (normalized) exponential distribution $F(\tau) = \exp(-\mu\tau)$ with $\mu = \nu = 1/\langle\tau\rangle$ (equivalent to fitting μ using the first moment of the distribution). The χ^2 was computed by dividing the distributions into 11 intervals of equal probability [32]. Comparing the values obtained for the exponential and stretched exponential cases we found that in $2,110$ out of $2,128$ words the stretched exponential provided a better fit than the exponential (smaller χ^2). The other cases were the words with very high frequency and very high χ^2. For $2,060$ words the value of χ^2 of the stretched exponential fit was five times smaller than the value of χ^2 of the exponential fit. The median χ^2 for the exponential distribution was $\chi^2_{median} = 32,652$, which should be compared to $\chi^2_{median} = 3,871$ for the stretched exponential distribution.

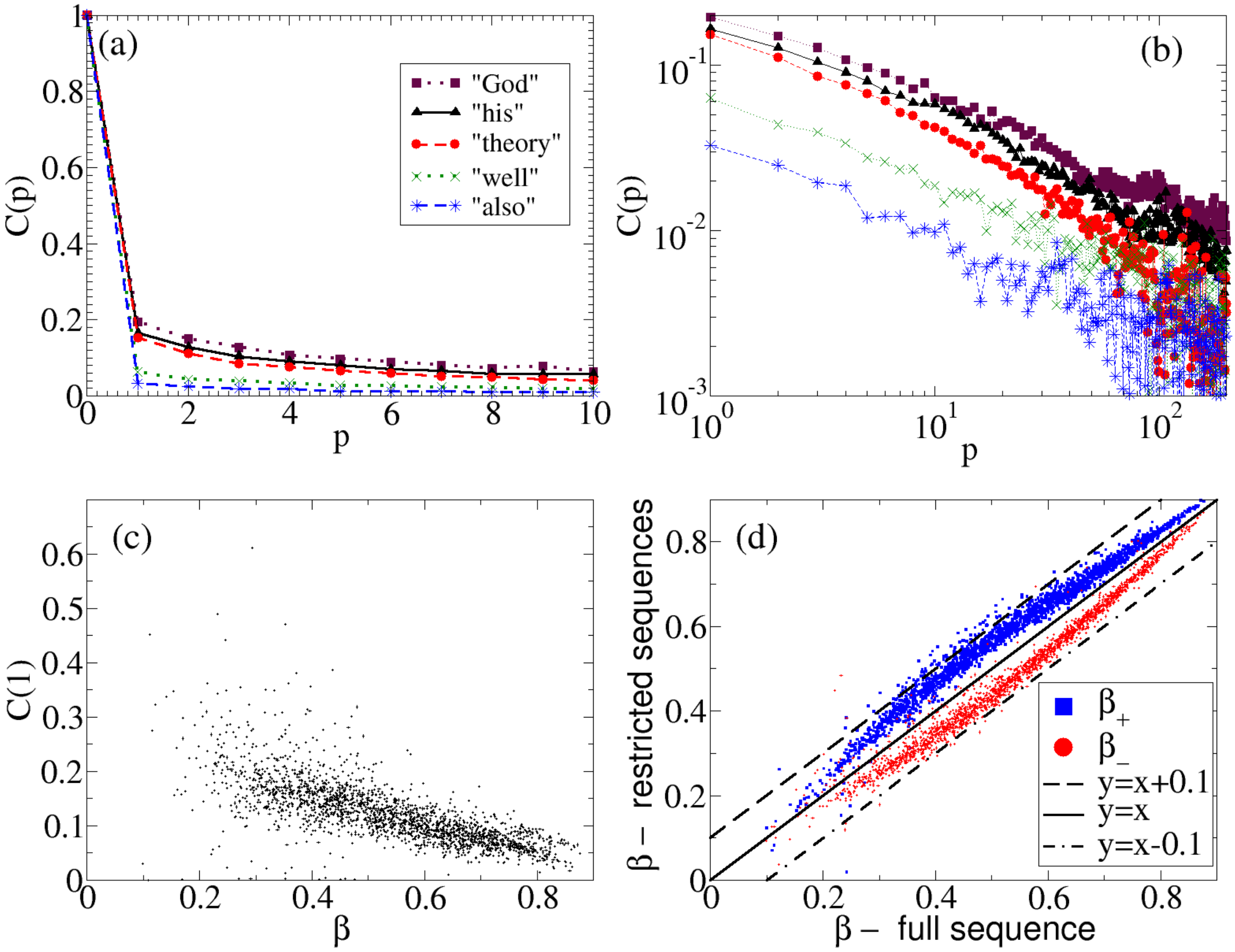

FIG. 8: Auto-correlation function (2) of the sequence of distances $\{\tau_j\} = \tau_1, \tau_2, ..., \tau_{N_w}$ for words in the USENET group talk.origins. A representation in (a) linear and (b) logarithmic scales for five words show a significant drop in of the correlation for $p = 1$ and slow decay for larger p. (c) $C(1)$ versus β for all 2,128 words analyzed. (d) Value of β obtained using the full sequence $\{\tau_j\}$ (x-axis) and two sub-sequences ($\{\tau_j\}_+$ and $\{\tau_j\}_-$, y-axis) for the 2,128 words in the talk.origins database. The two sub-sequences correspond to recurrence times (τ_j) that follow recurrence times larger (+) and smaller (−) than the median recurrence times ($\tau_{j-1} > \tau_{median}$ and $\tau_{j-1} < \tau_{median}$, respectively).

D. Correlation in $\{\tau_j\}$

The renewal model proposed in the main text asserts that no memory of previous recurrence times is present in the sequence $\{\tau_j\}$. The extent to which this is valid is quantified by calculating the auto-correlation function. The auto-correlation function $C(p)$ of a sequence $\{\tau_j\}$ is defined as a function of the distance p,

$$C(p) = \frac{1}{\sigma_\tau^2}(\langle \tau_i \tau_{i+p} \rangle - \langle \tau \rangle^2) = \frac{1}{\sigma_\tau^2} \frac{1}{N_w - p} \sum_{j=1}^{N_w - p} (\tau_j \tau_{j+p} - \langle \tau^2 \rangle), \qquad (2)$$

where $\sigma_\tau = \sqrt{\langle \tau^2 \rangle - \langle \tau \rangle^2}$ and $\langle . \rangle$ corresponds to the average over all i (the other parameters are the same as defined in the main text). The results obtained for all the words in the talk.origins database are presented in Figure 8. These results show that the correlation is relatively small already for $p = 1$ (two consecutive recurrence times) but that it decays slowly as p is increased. One approach to account for this correlation is to consider that τ_j depends on previous recurrence times (τ_k with $k < j$), as discussed in Ref. [33].

E. Independence of $\{\tau_j\}$

The correlation in $\{\tau_j\}$ described in Sec. II D quantifies the extent to which $\{\tau_j\}$ can be considered as a sequence of independent identically distributed (IID) random variables. As a consequence, it quantifies the extent to which a renewal process (as proposed in the manuscript) can be used to describe word usage. In this section, we report an additional test that quantifies how this correlation affects whether the $\{\tau_j\}$ are identically distributed.

We divide the sequence of $\{\tau_j\}$ into two equal-sized sub-sequences ($\{\tau_j\}_+$ and $\{\tau_j\}_-$) depending whether the previous time is greater (+) or smaller (−) than the median of the full sequence $\{\tau_j\}_{median}$. That is, $\tau_i \in \{\tau_j\}_+ \Leftrightarrow \tau_{i-1} > \{\tau_j\}_{median}$ and $\tau_i \in \{\tau_j\}_- \Leftrightarrow \tau_{i-1} \leq \{\tau_j\}_{median}$. Figure 8(d) shows the values of β obtained for these two sequences (β_+ and β_-, respectively) compared to the value of β of the full sequence. Each point corresponds to one of the $2,128$ words in the talk.origins database. For IID random variables all points would approach the diagonal line $y = x$. In Fig. 8(d) we find that that almost all values are within $y = x \pm 0.1$. However, the two sub-sequences are clearly distinguishable from each other: on the one hand, recurrence times following short recurrence times consistently have $\beta_- < \beta$, suggesting that they are more bursty; on the other hand, recurrence times following large recurrence times consistently have $\beta_+ > \beta$, and are thus closer to the exponential case. We believe these results, together with our previous results for the auto-correlation, provide sound evidence that the renewal process can be considered a good first order approximation to describe the burstiness observed in the large datasets we analysed. Naturally, real texts exhibit additional structures related to the small positive correlation between nearby τ_i's, i.e., short (long) recurrence times are slightly more likely to follow short (long) recurrence times, a finding that deserves further investigation elsewhere.

F. Zipf-Alekseev Distribution

In Ref. [34] the recurrence times up to $\tau = 12$ of a content word in a short literary work were described using the Zipf-Alekseev (ZA) distribution:

$$f_{ZA}(\tau) = f_1 \tau^{-\alpha - b\ln(\tau)} ,$$

or, equivalently,

$$y_{ZA}(\tau) \equiv (f_{ZA}/f_1)^{1/\ln(\tau)} = A\ \tau^{-b}, \tag{3}$$

where $A = \exp(-\alpha)$. The ZA was broadly motivated by dynamic processes of conceptual elaboration identified in psychology.

In Fig. 9(a,b), examples of the distribution $y(\tau) = (f(\tau)/f_{max})^{1/\ln(\tau)}$ are shown for the words in the talk.origins and War and Peace databases (to avoid the cases when $f_1 = f(\tau = 1) = 0$ we replace f_1 in (3) by the empirically obtained maximum of $f(\tau)$, denoted f_{max}). The fits of the two parameters A, b in (3) is analogous to the fit performed for the stretched exponential distribution described in Sec. II A (the same values of the cutoff k and trimming of the largest times). The comparison between the empirical results and the two fits (stretched exponential and ZA distributions) is presented in Fig. 9(a-f). The results are shown in double logarithmic scales where (3) appears as a straight-line. The ZA distribution is nearly as good as the stretched exponential distribution in describing the behavior for short times and for very bursty words (small β, e.g., thematic nouns as originally investigated in Ref. [34]). However, the description is very poor for long τ and for words that are closer to the exponential distribution (large β). A clear signature of this behavior is the downward concavity apparent in Fig. 9(a,b), which we observed in the absolute majority of words we investigated. This is in agreement with the stretched exponential prediction and in disagreement with the (straight line) prediction of the ZA distribution (3). In Figs 9(c-f) we confirm this observation by plotting the residuals of the fits of a large number of words in our database for the stretched exponential [Fig. 9(c,d)] and the ZA [Fig. 9(e,f)] distributions. It is apparent that in the tail of the distribution the residuals of the stretched exponential are smaller in size than those of the ZA distribution. The concavity mentioned above appears as systematic deviations in panel (e,f), where the residuals of the fit of the ZA distribution show that for almost all words the ZA distribution (i) underestimates $f(\tau)$ for $\tau \approx \langle\tau\rangle$ and (ii) overestimates $f(\tau)$ for long τ. Other functions with an asymptotic decay between exponential and power law have recently been used to describe the recurrence time distributions, and are candidates for future investigation [32, 35].

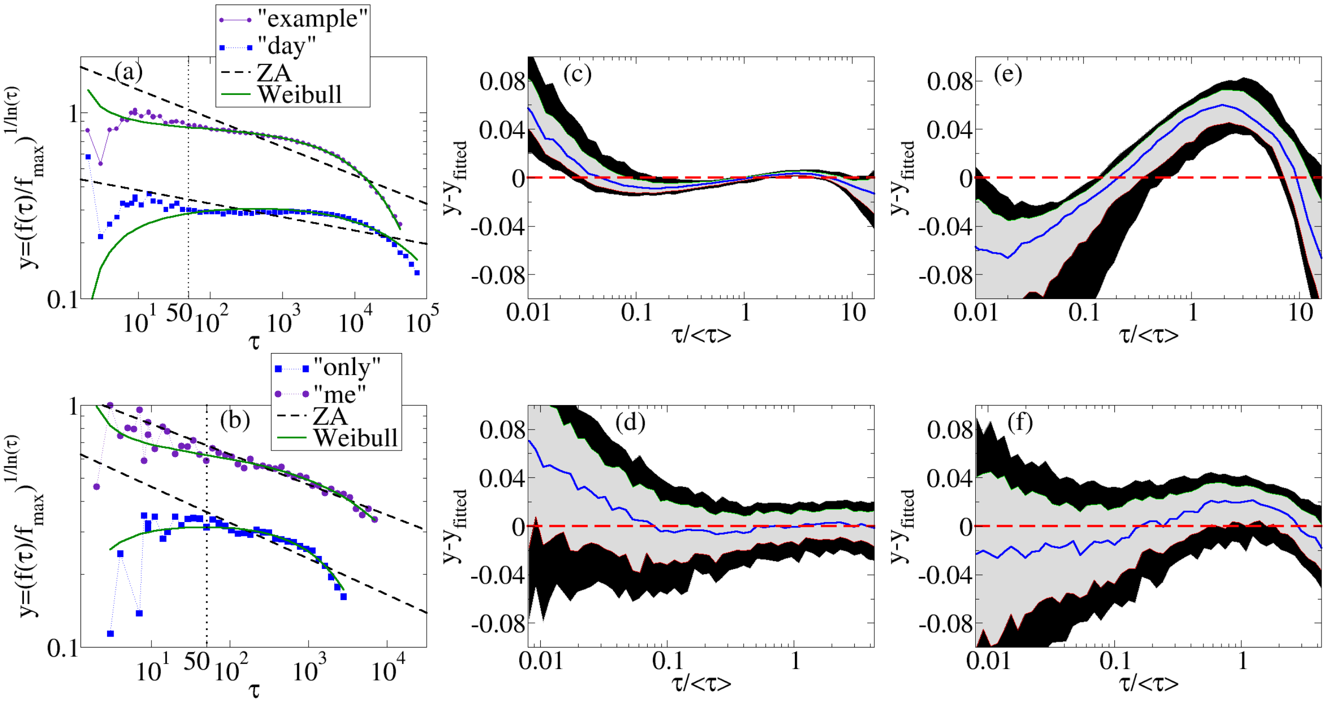

FIG. 9: Comparison between the Zipf-Alekseev (ZA) distribution (3) proposed in Ref. [34] and the stretched exponential distribution proposed in our manuscript. Top row: the 500 most frequent words in the talk.origins database. By restricting the analysis to the most frequent words we ensure that the results are not an artifact of the noise in the USENET database (similar plots are obtained using all words in our database). Bottom row: the 633 most frequent words in the *War and Peace* database. (a,b) Example of two words showing that for long $\langle\tau\rangle$ the empirical distribution decays faster than the ZA distribution. (c,d) Residuals of the stretched exponential (Weibull) distribution. (e,f) Residuals of the ZA distribution. In (c-f), the solid blue line is the median, the border of the gray region corresponds to the 2nd and 6th octiles, and the border of the black region corresponds to the 1st and 7th octiles.

III. COUNTING MODELS

A. Counting Distribution

An important quantity in the document classification literature is the counting or occupancy distribution $G_s(x)$, defined as the probability of finding s tokens (occurrences of a specific word) in a text of x words. Here again a Poisson process can be taken as a starting point (fixed probability μ of using the word), leading to distribution

$$G^{Poisson}_{s,\mu}(x) = \frac{e^{-\mu x}(\mu x)^s}{s!}, \tag{4}$$

where μx is the expected number of tokens.

It was soon realized [36] that the probability of using content words strongly depends on the document and that a Poisson process fails to generate, for example, the observed distribution of keywords for the collection of all books in a library. For these cases, models typically assume that different documents have different probabilities of word usage μ's. Consider that these probabilities are distributed according to $\Phi(\mu)$. The fraction of documents having s occurrences of the word is then given by

$$G_s(x) = \int_0^\infty \Phi(\mu) C^{Poisson}_{s,\mu}(x) d\mu. \tag{5}$$

This type of model is called a Poisson mixtures model in Ref. [23]. The focus is usually on the dependence on s for an approximately constant x (document size). Perhaps the most popular choice to model the broad variety of μ's across documents is to consider $\Phi(\mu)$ to be a gamma distribution

$$\Phi_{qr}(\mu) = \frac{\mu^{N-1} e^{-\mu/q}}{q^r \Gamma(q)},$$

in which case one obtains a negative binomial counting distribution [23, 37]

$$G_s(x) = \frac{\Gamma(q+s)}{s!\Gamma(q)}(rx)^k(1+rx)^{-q-s}, \tag{6}$$

where q, r are parameters and Γ is the gamma function.

An important difference between these studies and our approach is that we treated the full text of our database as a single document produced as the outcome of a single process. We can partially relate our approach to Eq. (5) by considering that the text is composed of different topics. Inside each topic the probability of using the specific word is fixed equal to μ, but μ varies from topic to topic according to $\Phi(\mu)$ (e.g., following a gamma distribution). However, in spirit this is still substantially different from our approach: we consider a continuous process and, as a result, we do not assume that boundaries between topics are well-defined, or that the probability of occurrence of a specific word is constant over any segment of the text.

Let us now restrict ourselves to the class of renewal processes considered in the manuscript. First we notice that from the definition of renewal process (sequence $\{\tau_j\}$ is independent and identically distributed) that this stochastic process is completely defined by $F(\tau)$. Consider now the probability of finding no occurrence of the word up to time $x = \tau$. This is given both by $G_{s=0}(\tau)$ and $F(\tau)$, an observation that leads to the following relation between the counting distribution $F(\tau)$ and the cumulative recurrence time distribution $G_s(x)$ (see also [38]):

$$F(\tau) = G_{s=0}(\tau). \tag{7}$$

Applying (7) to the distribution (4) we recover the exponential distribution of recurrence times $F(\tau) = \exp(-\mu\tau)$. Applying (7) to the negative binomial (6) we obtain

$$F(\tau) = (1 + r\tau)^{-q}, \tag{8}$$

which corresponds to a power-law distribution for long τ. Our empirical analysis of different databases show that most words have a recurrence time distribution that lies between the exponential and the power-law distributions obtained from the most common counting models, and that they are well described by a stretched exponential.

B. Hazard Function

The hazard function $m(t)$ is defined as the probability of having an event (an occurrence of word) at time t given that no event happened until t:

$$m(t) := Pr(\tau = t|\tau \geq t) = \frac{Pr(\tau = t)}{Pr(\tau \geq t)} = \frac{f(t)}{F(t)}. \tag{9}$$

Inverting this relationship by noting that $f(t) = dF(t)/dt$ we obtain:

$$F(\tau) = \exp(-\int_0^\tau m(t)dt), \tag{10}$$

which corresponds to Eq. (3) of the main text. This shows that Eq. (4) of the main text corresponds to the hazard function of the stretched exponential distribution. It predicts a power-law decay in time. Figure 10 shows the results obtained by applying Eq. (9) to words of the talk.origins USENET group and of the novel *War and Peace*. The decay of $m(t)$ with t is a signature of burstiness. More important, the *straight-line* decay in the log-log scale corresponds, in the case of renewal processes, to a stretched exponential distribution for the resulting recurrence time distribution. The examples shown in Fig. 10 are representative of the words in our database, and the observed empirical power-law decay of $m(t)$ over two or more decades is in strong support of Eq. (4) in our manuscript. For some words in the USENET databases (specially at low frequencies) we observe a slower decay of $m(t)$ for long times (e.g., *fundamentalists* in Fig. 10a), suggesting that for extremely long times the process possibly loses memory and the recurrence time distribution decays exponentially. This observation is in agreement with the interpretation of the residuals for the stretched exponential (see Fig. 6 above).

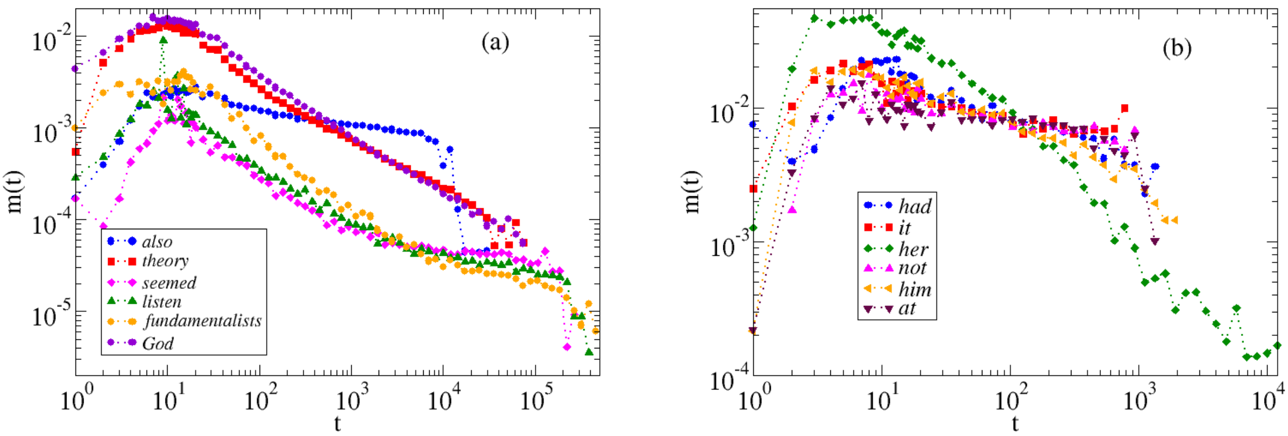

FIG. 10: Hazard function (9) of (a) the six words of the talk.origins USENET group shown in the manuscript and (b) six frequent words ($\nu \approx 0.001$) of the novel *War and Peace*.

[1] Marslen-Wilson, W. D. and Tyler, L. K., *Morphology, language and the brain: The decompositional substrate for language comprehension*, Philosophical Transactions of the Royal Society B: Biological Sciences **362**, 823–836 (2007).
[2] Baayen, H. and Schreuder, R., *War and Peace: Morphemes and full forms in a noninteractive activation parallel dual-route model*, Brain and Language **68**, 27–32 (1999).
[3] Hay, J., *Causes and Consequences of Word Structure*, Routledge (2003).
[4] Marcus, G. F., Brinkmann, U., Clahsen, H., Wiese, R., and Pinker, S., *German inflection: The exception that proves the rule*, *Cognitive Psychology* **29**, 189–256 (1995).
[5] Pinker, S. and Ullman, M., *The past and future of the past tense*, TRENDS in Cognitive Sciences **6**, 456–463 (2002).
[6] Hare, M. L., Ford, M., and Marslen-Wilson, W. D., *Ambiguity and frequency effects in regular verb inflection.* In Bybee, J. and Hopper, P. (eds.), *Frequency and the emergence of linguistic structure*, John Benjamins (2001), pp. 181–200.
[7] McClelland, J. L and Patterson, K., *Rules or connections in past tense inflections*, TRENDS in Cognitive Sciences **6**, 465–471 (2002).
[8] McClelland, J.L. and Patterson, K., *'Word or rules' cannot exploit the regularity in exceptions*, TRENDS in Cognitive Sciences **6**, 464–465 (2002).
[9] Bell, A., Brenier, J., Gregory, M., Girand, C., and Jurafsky, D., *Predictability effects on durations of content and function words in conversational English*, Journal of Memory and Language **60**, 92–111 (2009).
[10] van Benthem, J., *Fine-structure in categorial semantics.* In Rosner, M. and Johnson, R. (eds.) *Computational Linguistics and Formal Semantics*, Cambridge University Press (1992), pp. 127–157.
[11] von Fintel, K., *The formal semantics of grammaticalization*, Proceedings of NELS 25: Papers from the Workshops on Language Acquisition & Language Change GLSA, Vol. 2 (1994), pp. 175–189.
[12] Montague, R., *The proper treatment of quantification in ordinary English.* In Hintikka, J., Moravscik, J., and Suppes, J. (eds.), *Approaches to Natural Language*, Reidel (1973), pp. 373–398.
[13] Partee, B. H., *Syntactic categories and semantic type.* In Rosner, M. and Johnson, R. (eds.), *Computational Linguistics and Formal Semantics*, Cambridge University Press (1992), pp. 97–126.
[14] Whitehead, A. N. and Russell, B. *Principia Mathematica*, 3 vols, Cambridge University Press (1910, 1912, 1913).
[15] Fellbaum, C. (ed.), *WordNet: An electronic lexical database*, MIT Press (1998).
[16] Heim, I., *E-Type pronouns and donkey anaphora*, Linguistics and Philosophy **13**, 137–177 (1990).
[17] Cooper, R., *The Interpretation of pronouns.* In Heny, F. and Schnelle H. (eds.), *Syntax and Semantics 10*, Academic Press (1979), pp. 61–92.
[18] Evans, G., *Pronouns*, Linguistic Inquiry **11**, 337–361 (1980).
[19] Kruskal, W., Relative Importance by Averaging Over Orderings, *The American Statistician* **41**, 6 (1987).
[20] Jones, K. S., *A statistical interpretation of term specificity and its application in retrieval*, Journal of Documentation **28**, 11–21 (1972).
[21] Yang, Y., *An evaluation of statistical approaches to text categorization*, Information Retrieval **1**, 69–90 (1999).
[22] Nigam K., McCallum A., Thrun S., and Mitchell T., *Text classification from labeled and unlabeled documents*, Machine Learning **39**, 103–134 (2000).
[23] Church, K. W. and Gale, W. A., *Poisson Mixtures*, Natural Language Engineering **1**, 163–190 (1995).
[24] Church, K., *Empirical estimates of adaptation: The chance of two Noriegas is closer to* $p/2$ *than* p^2, Proceedings of Coling, 180–186 (2000).
[25] Pollard, C. and Sag, I., *Head-driven Phrase-Structure Grammar*, University of Chicago Press (1994).
[26] Steedman, M., *The Syntactic Process*, MIT Press (2000).
[27] Kempson, R., Meyer-Viol, W., and Gabbay, D., *Dynamic syntax: The Flow of Natural Language Understanding*, Blackwell (2001).
[28] Montemurro, M. A., Zanette, D. H., *Entropic analysis of the role of words in literary texts*, Advances in Complex Systems **5**, 7–17 (2002).
[29] Grosz, B., Joshi, A., and Weinstein, S., *Centering: A framework for modeling the local coherence of discourse*, Computational Linguistics **21**, 203–226 (1995).
[30] Laherrere, J. and Sornette, D., *Stretched exponential distributions in nature and economy: "Fat tails" with characteristic scales*, European Physical Journal B **2**, 525–539 (1998).
[31] D'Agostino, R. B. and Stephens, M. A. *Goodness-of-fit techniques*, Marcel Dekker, New York (1986).
[32] Politi, M. and Scalas, E. *Fitting the empirical distribution of intertrade durations*, Physica A **387**, 2025–2034 (2008).
[33] Engle, R. F. and Russel, J.R., *Autoregressive conditional duration: a new model for irregularly spaced transaction data*, Econometrica **66**, 1127–1162 (1998).
[34] Hrebicek, L. *Text laws*, in *Quantitative Linguistics, an International Handbook*, Altmann G., Piotrowski R.G. eds., Walter de Gruyer, Berlin, p. 348 (2005)
[35] Corral, R., Ferrer-i-Cancho, R., Boleda, G., and Diaz-Guilera, A. *Universal complex structures in written language*, preprint arXiv:physics.soc-ph/0901.2924v1 (2009).
[36] Bookstein, A. and Swanson, D., *Probabilistic models for automatic indexing*, Journal of the American Society for Information Science **25**, 312 (1974).
[37] Mosteller, F. and Wallace, D. L., *Inference in an authorship problem - a comparative-study of discrimination methods applied to authorship of disputed federalist papers*, J. Am. Stat. Assoc. **58**, 275 (1963).

[38] McShane, B., Adrian, M., Bradlow, E. T., and Fader, P. *Count models based on Weibull interarrival times* Journal of Business and Economic Statistics **26**, 369–378 (2008).
