# Supplementary figures and images for: Beyond Word Frequency: Bursts, Lulls, and Scaling in the Temporal Distributions of Words (part 2 of 2)
Source: PLoS One. 2009 Nov 11;4(11):e7678. doi: 10.1371/journal.pone.0007678 (PMC2770836; doi:10.1371/journal.pone.0007678)

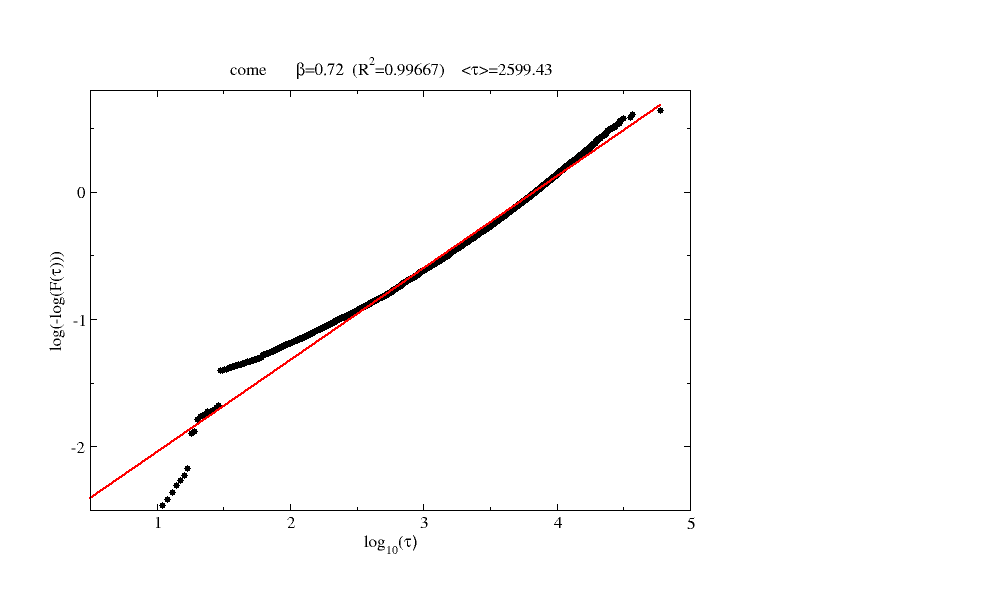

Supplement: Table S1 — Detailed information on the statistical analysis of all words that were studied (six databases). (31.88 MB TAR) [file pone.0007678.s002.tar › recurrence/comp/come.png]

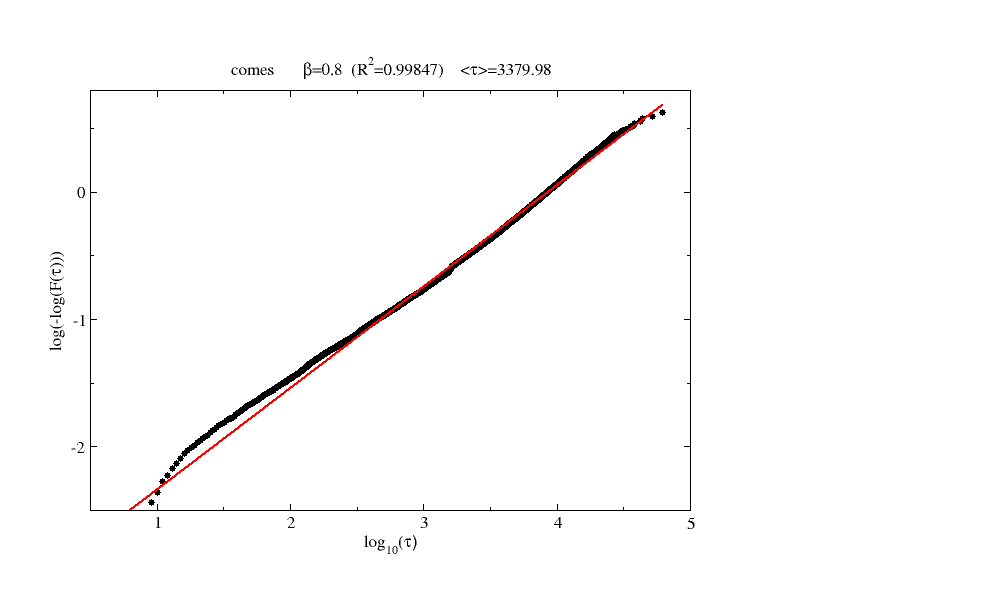

Supplement: Table S1 — Detailed information on the statistical analysis of all words that were studied (six databases). (31.88 MB TAR) [file pone.0007678.s002.tar › recurrence/comp/comes.png]

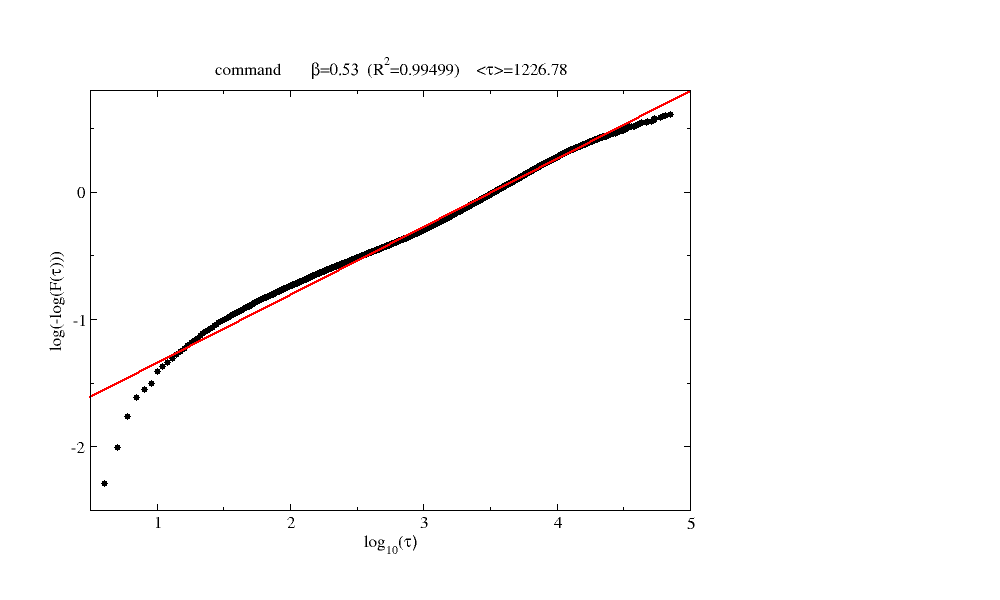

Supplement: Table S1 — Detailed information on the statistical analysis of all words that were studied (six databases). (31.88 MB TAR) [file pone.0007678.s002.tar › recurrence/comp/command.png]

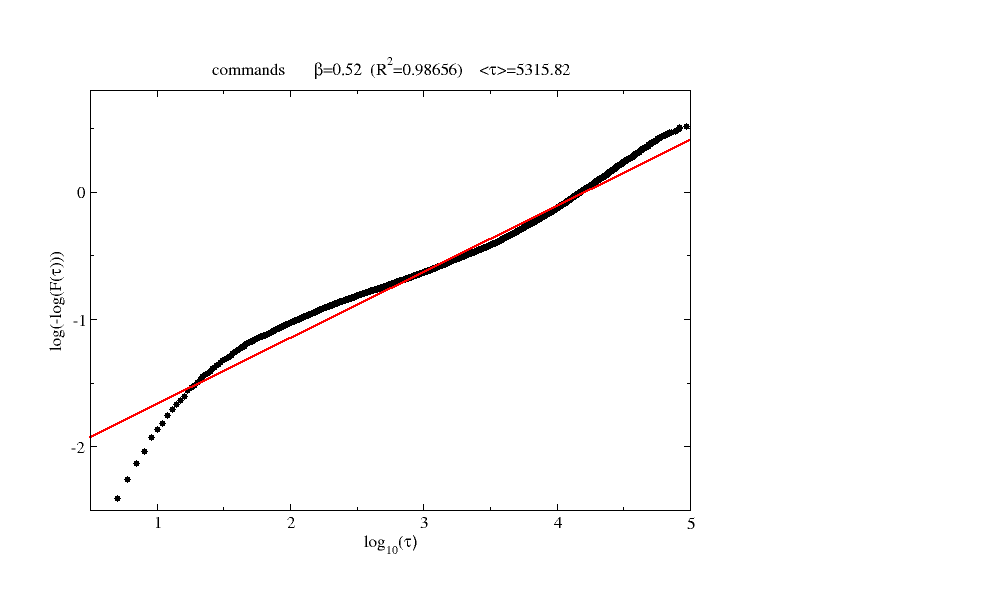

Supplement: Table S1 — Detailed information on the statistical analysis of all words that were studied (six databases). (31.88 MB TAR) [file pone.0007678.s002.tar › recurrence/comp/commands.png]

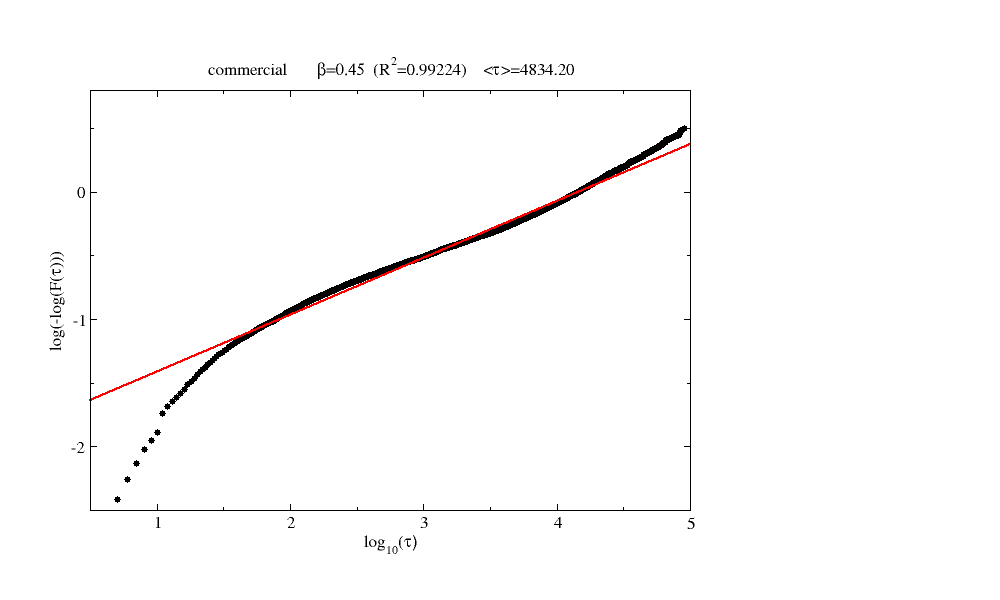

Supplement: Table S1 — Detailed information on the statistical analysis of all words that were studied (six databases). (31.88 MB TAR) [file pone.0007678.s002.tar › recurrence/comp/commercial.png]

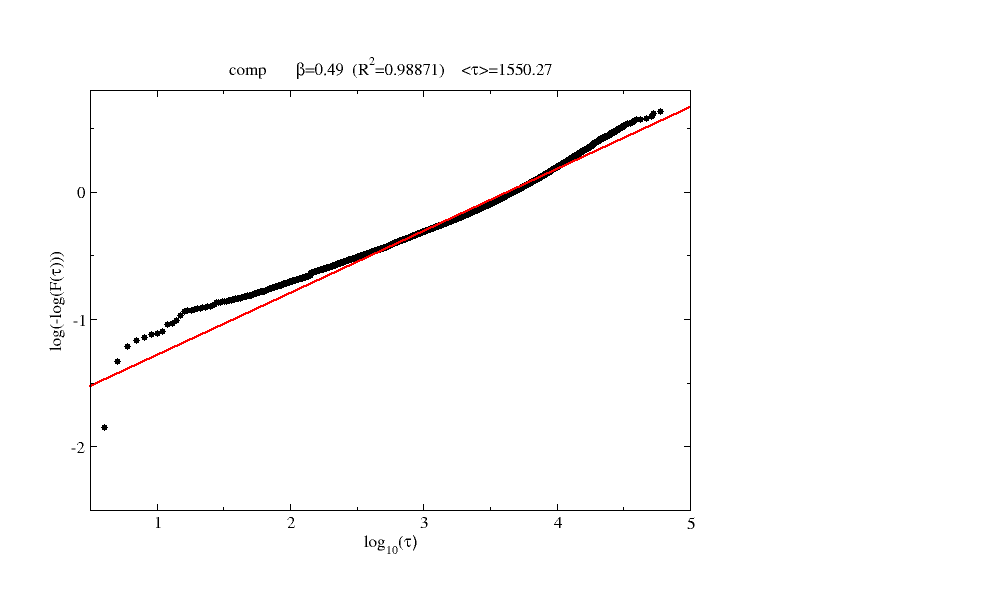

Supplement: Table S1 — Detailed information on the statistical analysis of all words that were studied (six databases). (31.88 MB TAR) [file pone.0007678.s002.tar › recurrence/comp/comp.png]

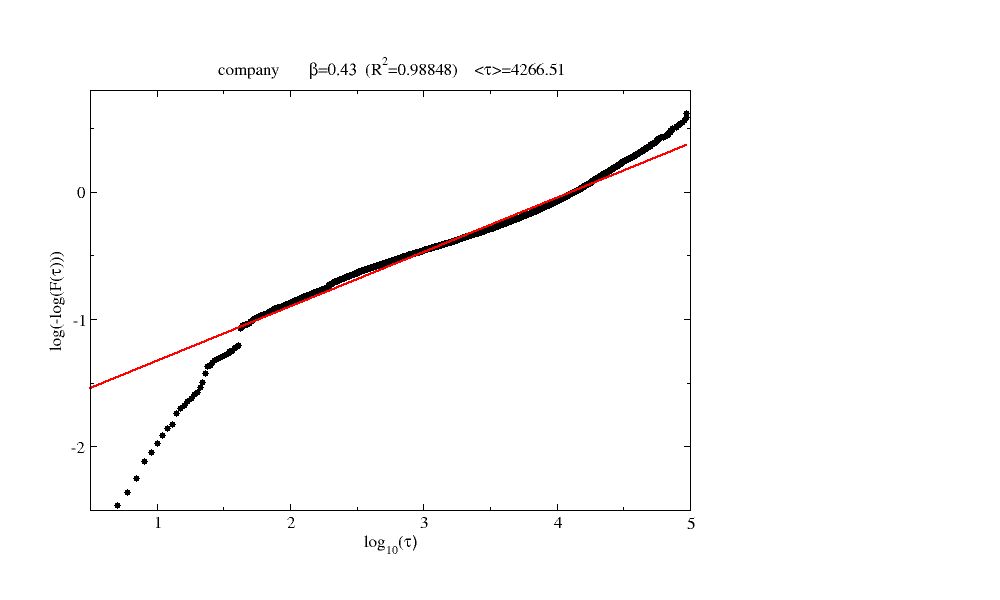

Supplement: Table S1 — Detailed information on the statistical analysis of all words that were studied (six databases). (31.88 MB TAR) [file pone.0007678.s002.tar › recurrence/comp/company.png]

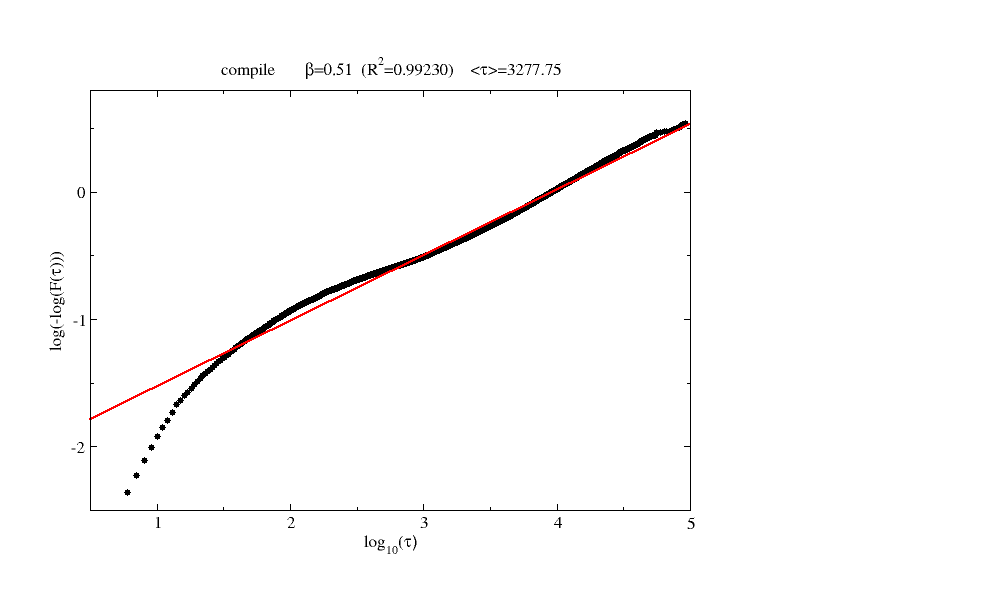

Supplement: Table S1 — Detailed information on the statistical analysis of all words that were studied (six databases). (31.88 MB TAR) [file pone.0007678.s002.tar › recurrence/comp/compile.png]

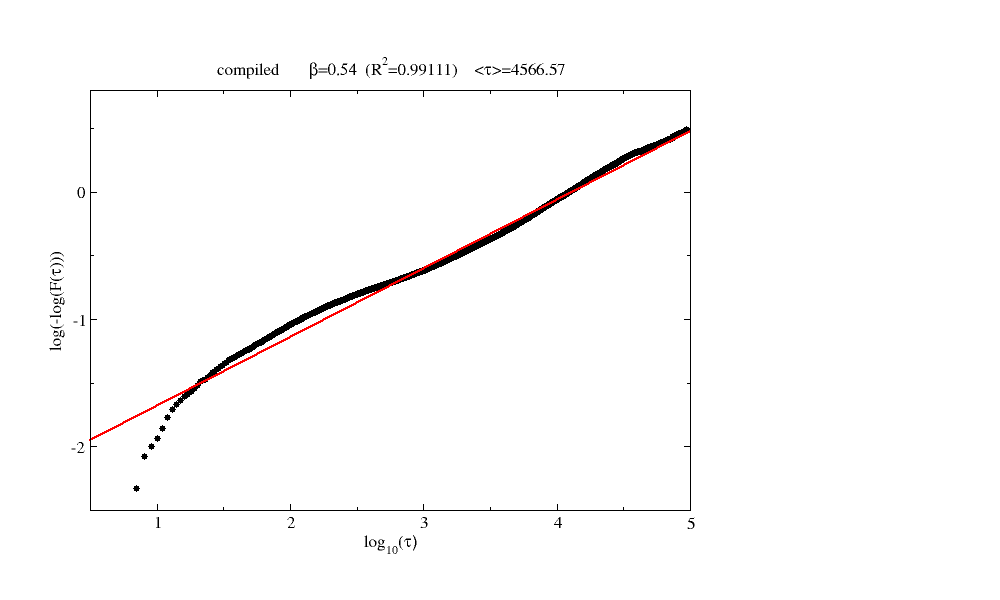

Supplement: Table S1 — Detailed information on the statistical analysis of all words that were studied (six databases). (31.88 MB TAR) [file pone.0007678.s002.tar › recurrence/comp/compiled.png]

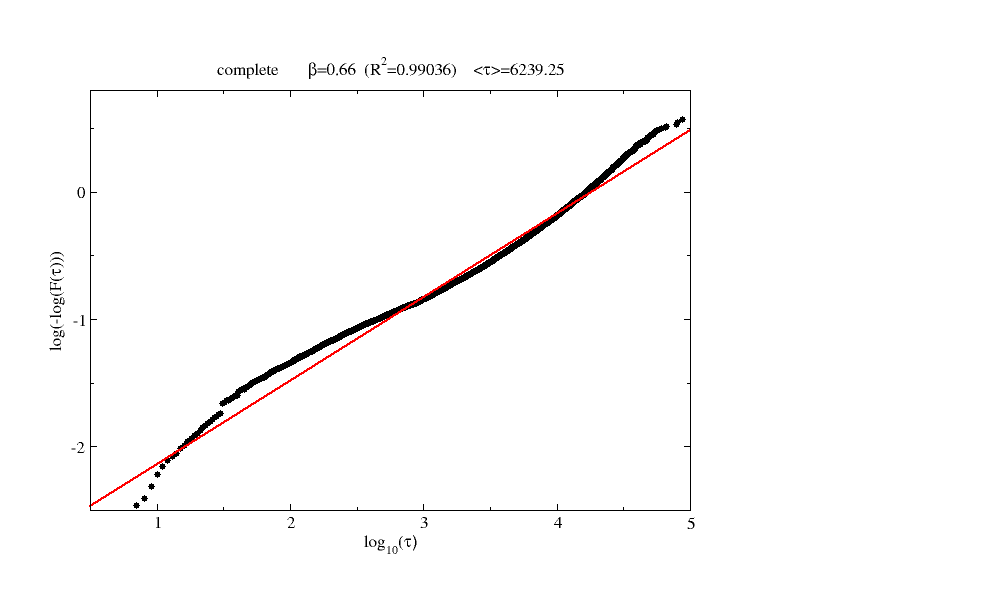

Supplement: Table S1 — Detailed information on the statistical analysis of all words that were studied (six databases). (31.88 MB TAR) [file pone.0007678.s002.tar › recurrence/comp/complete.png]

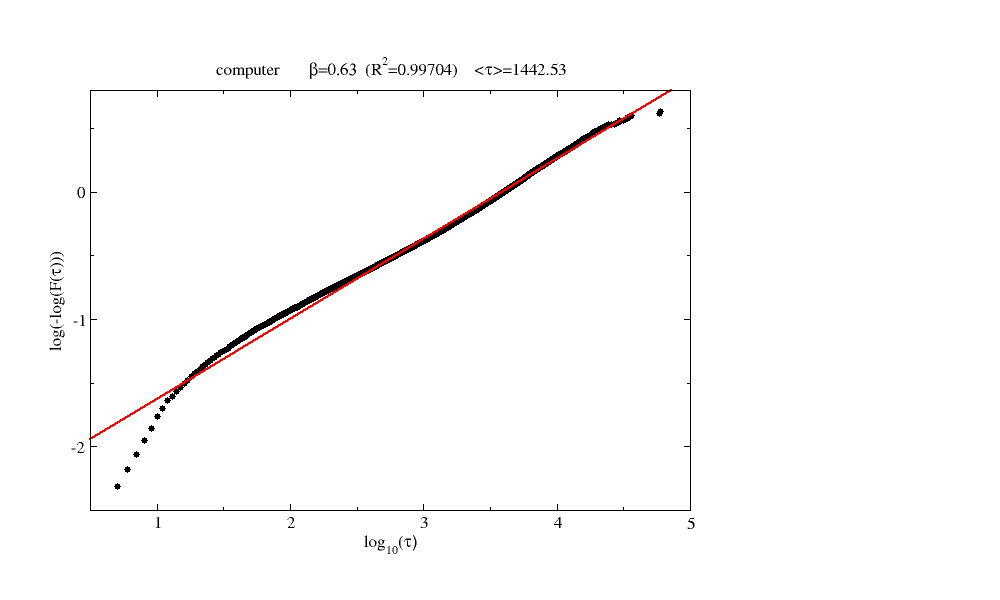

Supplement: Table S1 — Detailed information on the statistical analysis of all words that were studied (six databases). (31.88 MB TAR) [file pone.0007678.s002.tar › recurrence/comp/computer.png]

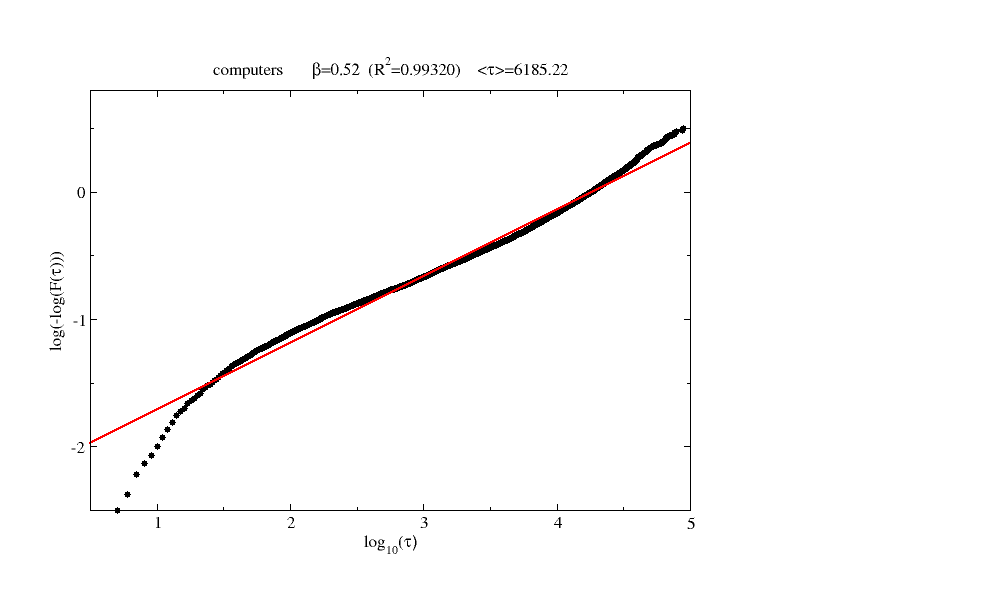

Supplement: Table S1 — Detailed information on the statistical analysis of all words that were studied (six databases). (31.88 MB TAR) [file pone.0007678.s002.tar › recurrence/comp/computers.png]

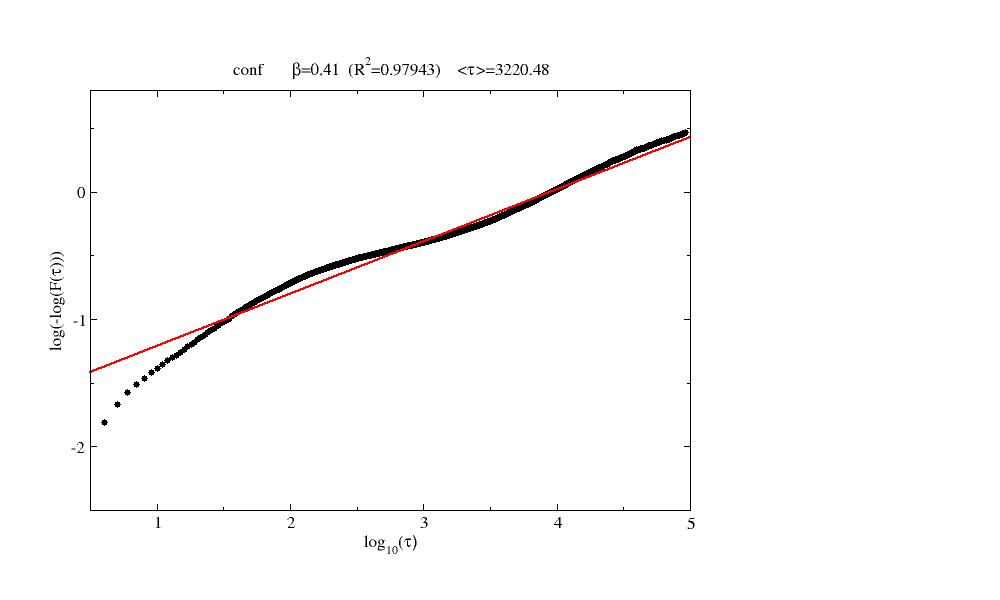

Supplement: Table S1 — Detailed information on the statistical analysis of all words that were studied (six databases). (31.88 MB TAR) [file pone.0007678.s002.tar › recurrence/comp/conf.png]

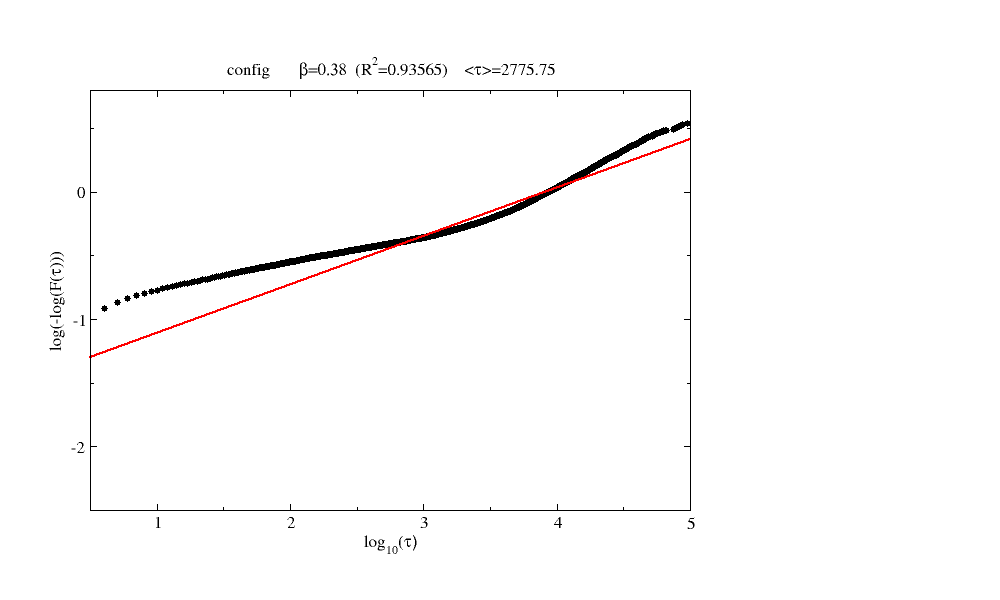

Supplement: Table S1 — Detailed information on the statistical analysis of all words that were studied (six databases). (31.88 MB TAR) [file pone.0007678.s002.tar › recurrence/comp/config.png]

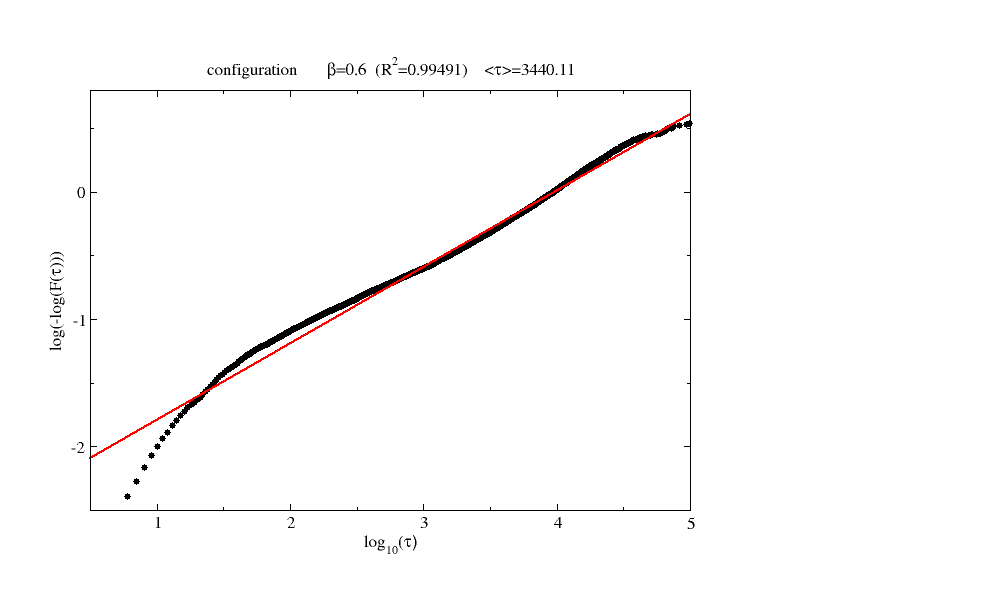

Supplement: Table S1 — Detailed information on the statistical analysis of all words that were studied (six databases). (31.88 MB TAR) [file pone.0007678.s002.tar › recurrence/comp/configuration.png]

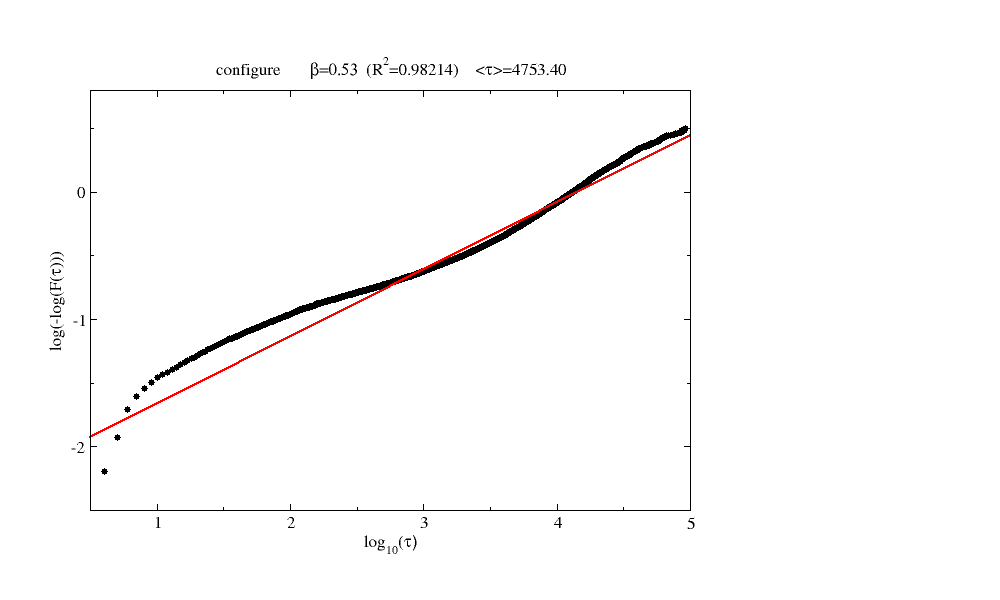

Supplement: Table S1 — Detailed information on the statistical analysis of all words that were studied (six databases). (31.88 MB TAR) [file pone.0007678.s002.tar › recurrence/comp/configure.png]

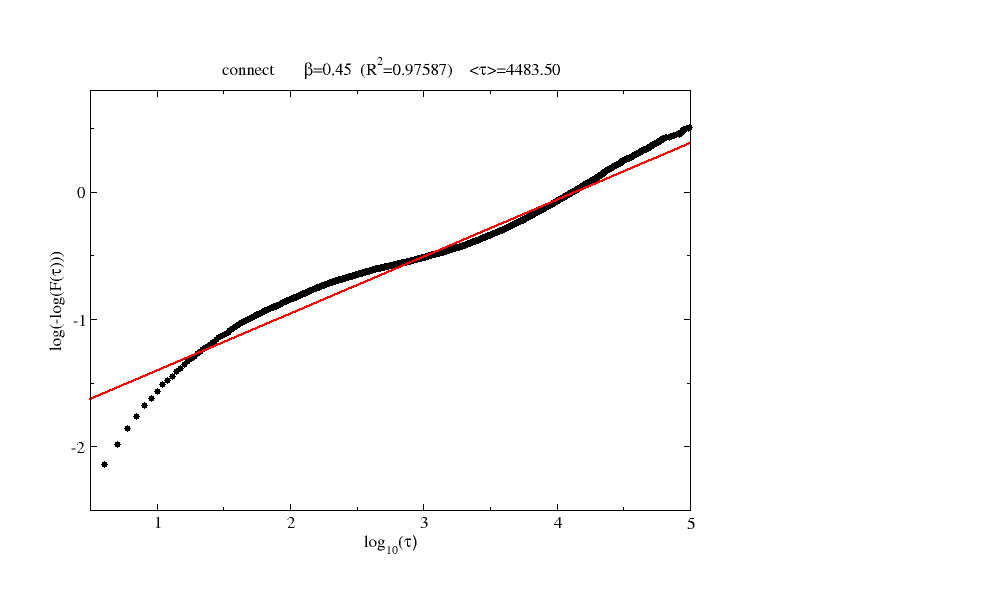

Supplement: Table S1 — Detailed information on the statistical analysis of all words that were studied (six databases). (31.88 MB TAR) [file pone.0007678.s002.tar › recurrence/comp/connect.png]

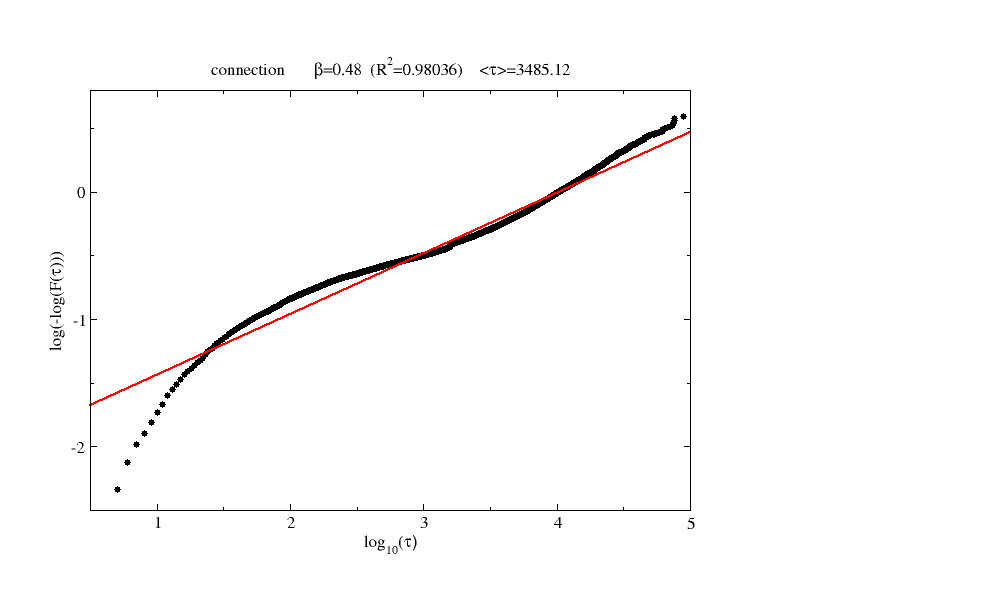

Supplement: Table S1 — Detailed information on the statistical analysis of all words that were studied (six databases). (31.88 MB TAR) [file pone.0007678.s002.tar › recurrence/comp/connection.png]

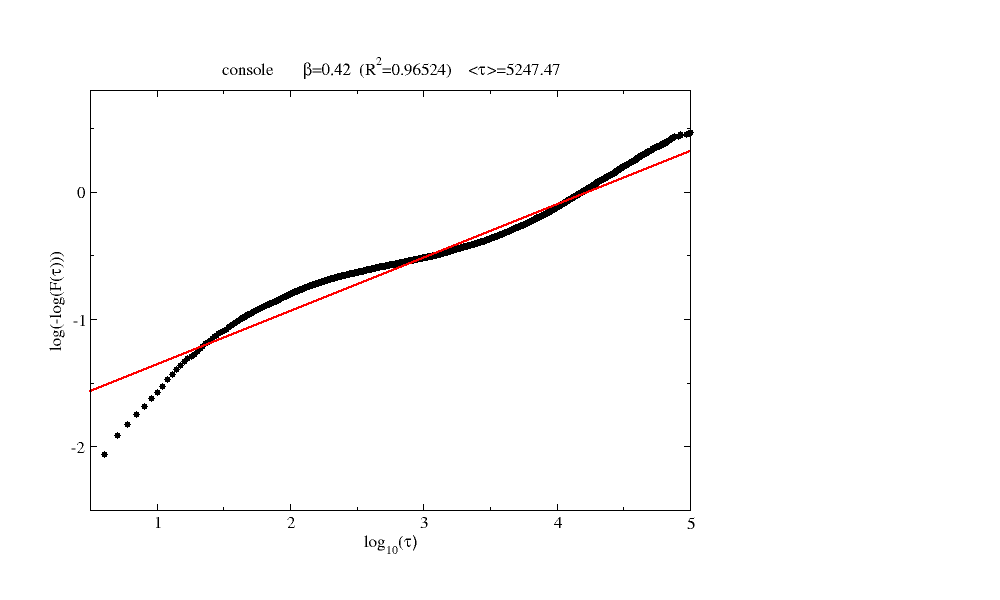

Supplement: Table S1 — Detailed information on the statistical analysis of all words that were studied (six databases). (31.88 MB TAR) [file pone.0007678.s002.tar › recurrence/comp/console.png]

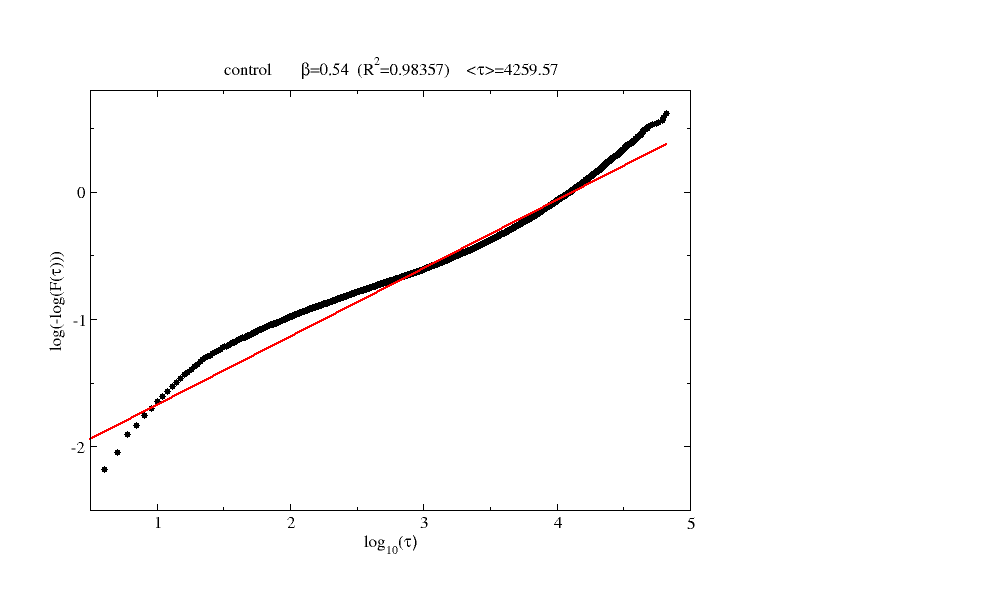

Supplement: Table S1 — Detailed information on the statistical analysis of all words that were studied (six databases). (31.88 MB TAR) [file pone.0007678.s002.tar › recurrence/comp/control.png]

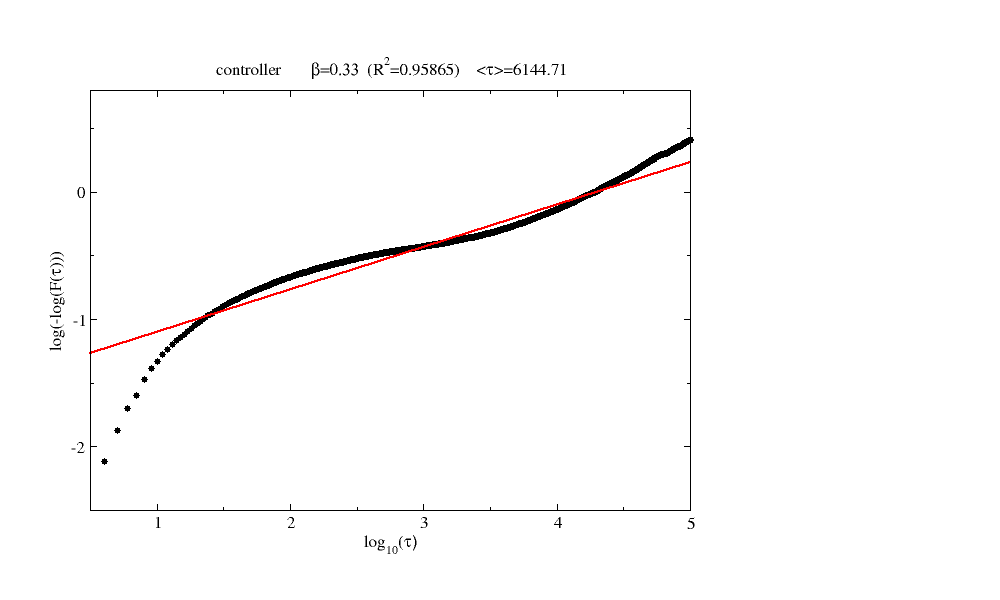

Supplement: Table S1 — Detailed information on the statistical analysis of all words that were studied (six databases). (31.88 MB TAR) [file pone.0007678.s002.tar › recurrence/comp/controller.png]

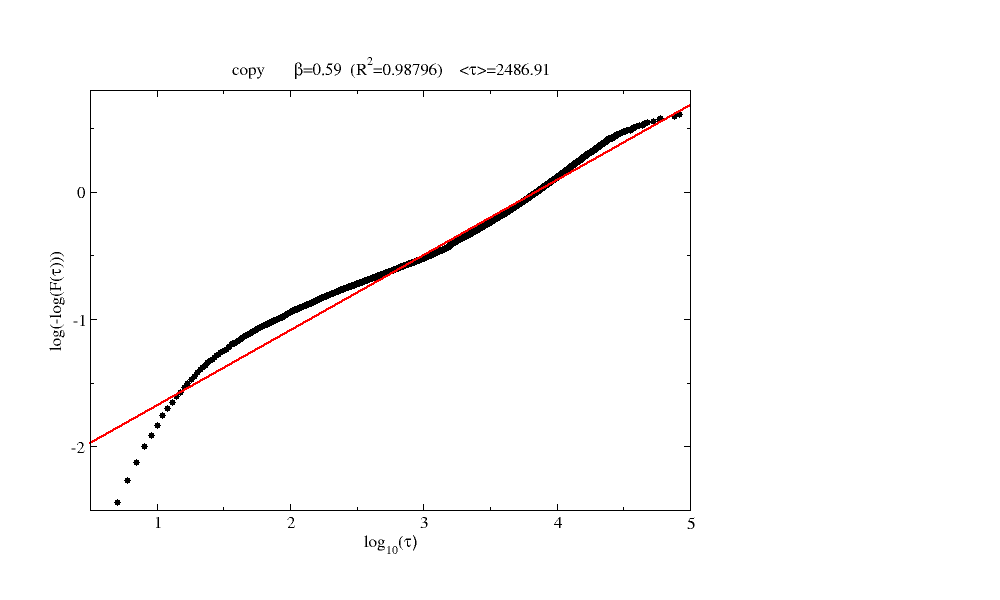

Supplement: Table S1 — Detailed information on the statistical analysis of all words that were studied (six databases). (31.88 MB TAR) [file pone.0007678.s002.tar › recurrence/comp/copy.png]

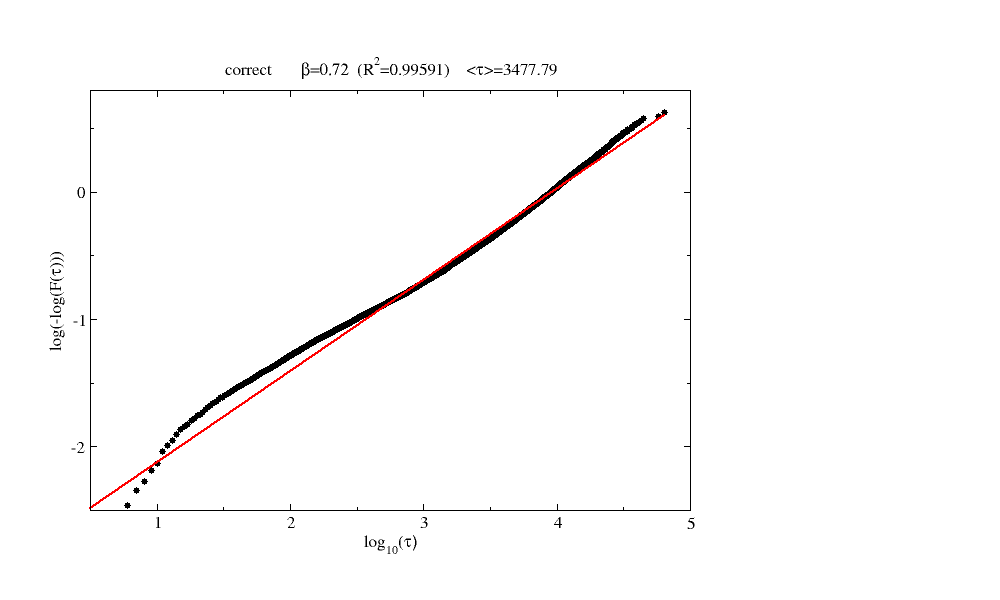

Supplement: Table S1 — Detailed information on the statistical analysis of all words that were studied (six databases). (31.88 MB TAR) [file pone.0007678.s002.tar › recurrence/comp/correct.png]

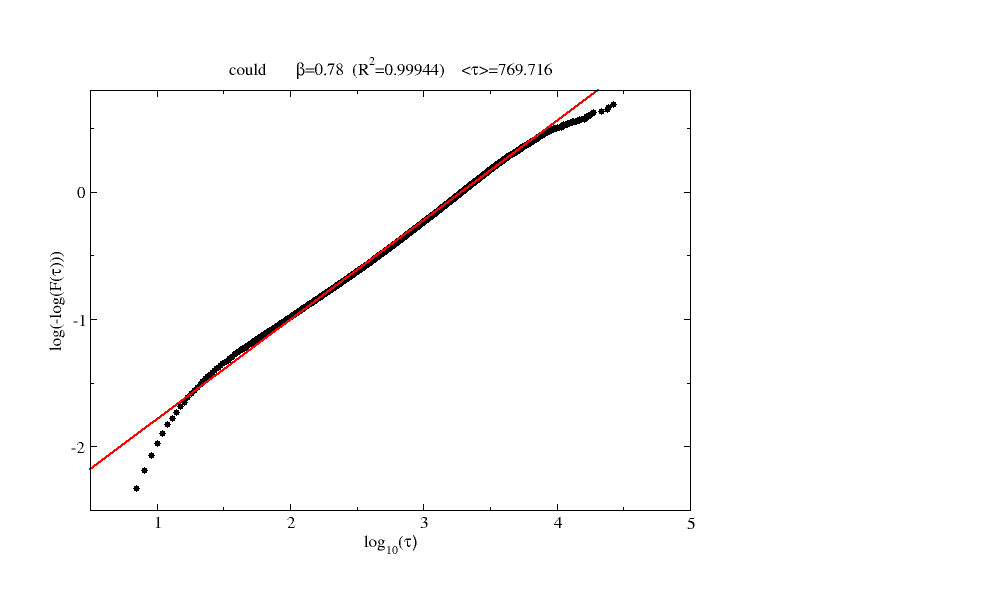

Supplement: Table S1 — Detailed information on the statistical analysis of all words that were studied (six databases). (31.88 MB TAR) [file pone.0007678.s002.tar › recurrence/comp/could.png]

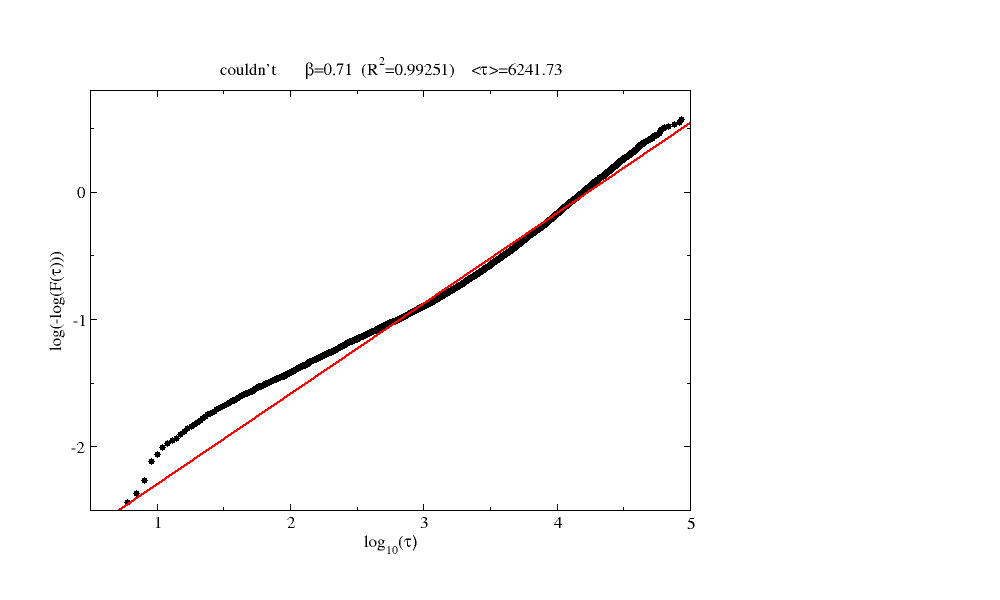

Supplement: Table S1 — Detailed information on the statistical analysis of all words that were studied (six databases). (31.88 MB TAR) [file pone.0007678.s002.tar › recurrence/comp/couldn't.png]

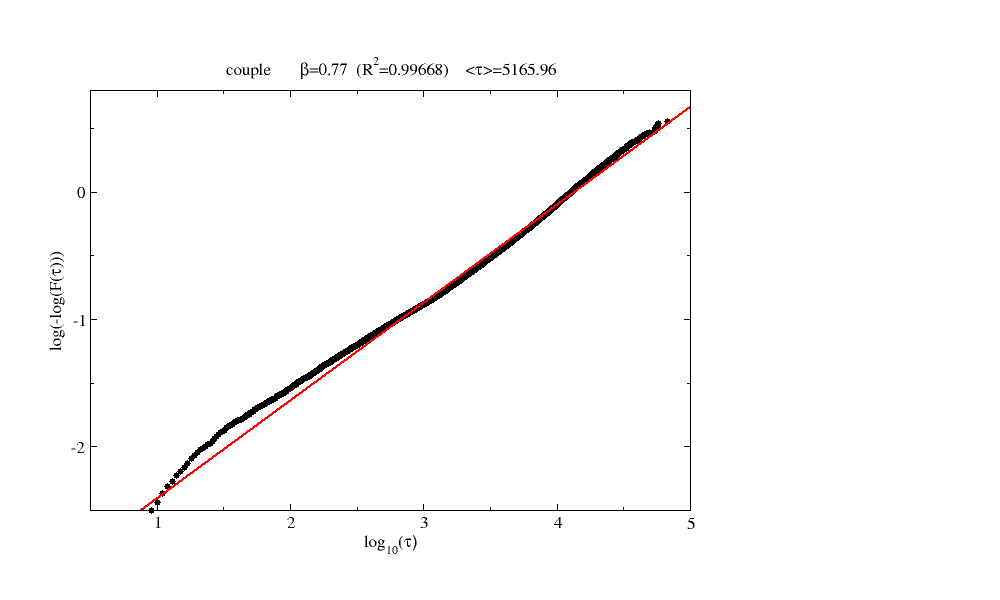

Supplement: Table S1 — Detailed information on the statistical analysis of all words that were studied (six databases). (31.88 MB TAR) [file pone.0007678.s002.tar › recurrence/comp/couple.png]

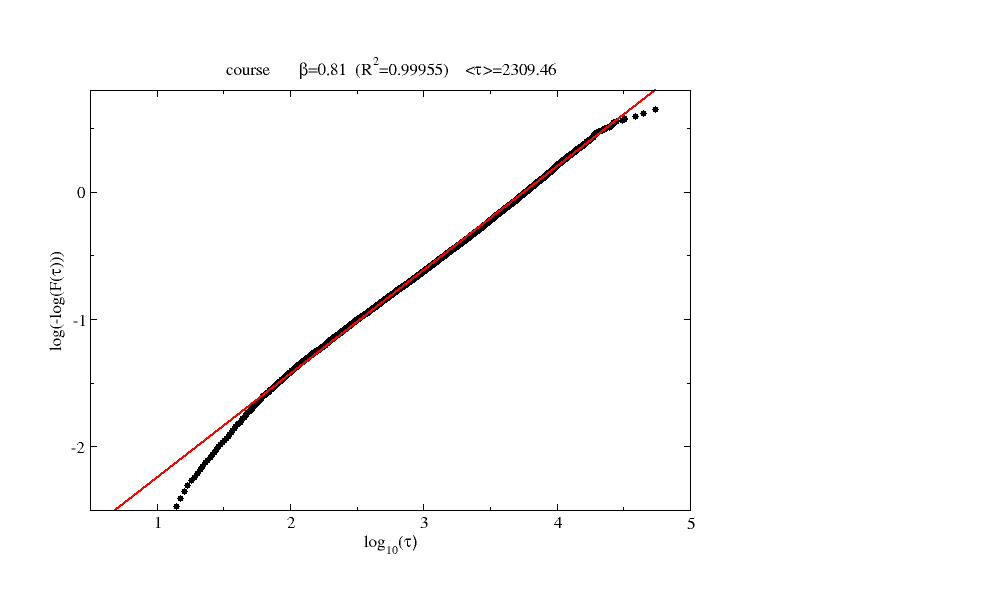

Supplement: Table S1 — Detailed information on the statistical analysis of all words that were studied (six databases). (31.88 MB TAR) [file pone.0007678.s002.tar › recurrence/comp/course.png]

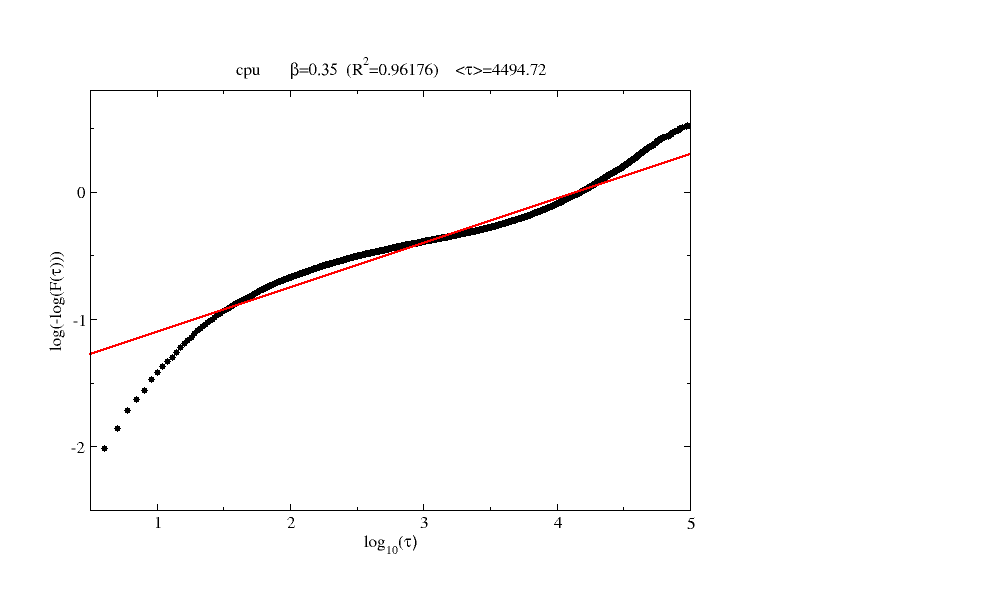

Supplement: Table S1 — Detailed information on the statistical analysis of all words that were studied (six databases). (31.88 MB TAR) [file pone.0007678.s002.tar › recurrence/comp/cpu.png]

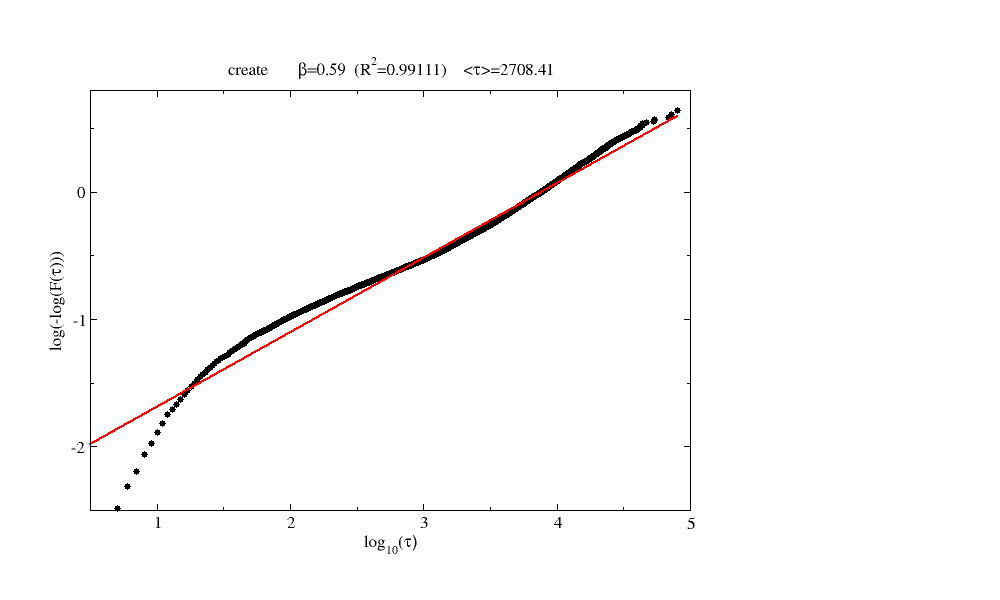

Supplement: Table S1 — Detailed information on the statistical analysis of all words that were studied (six databases). (31.88 MB TAR) [file pone.0007678.s002.tar › recurrence/comp/create.png]

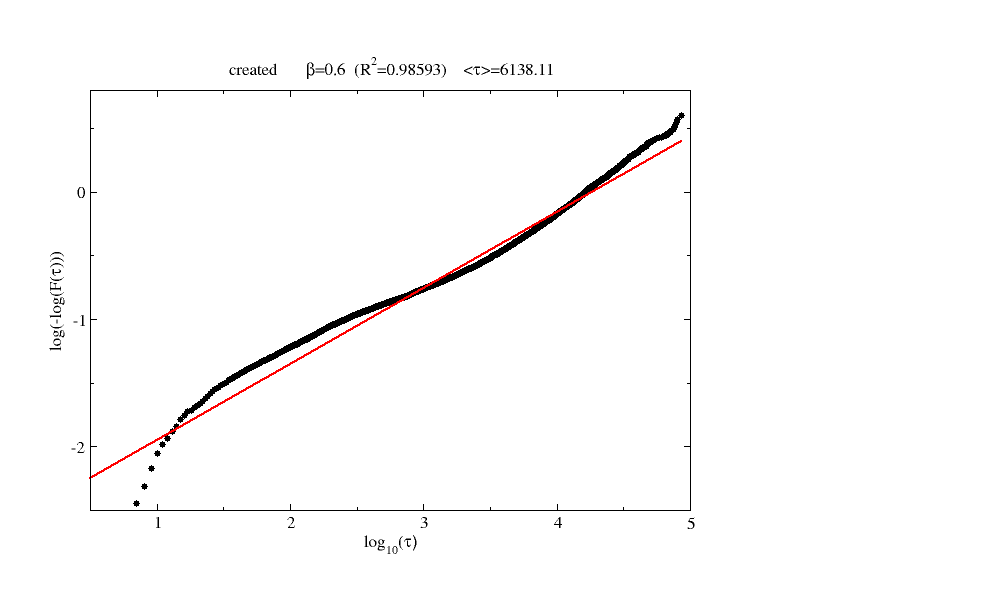

Supplement: Table S1 — Detailed information on the statistical analysis of all words that were studied (six databases). (31.88 MB TAR) [file pone.0007678.s002.tar › recurrence/comp/created.png]

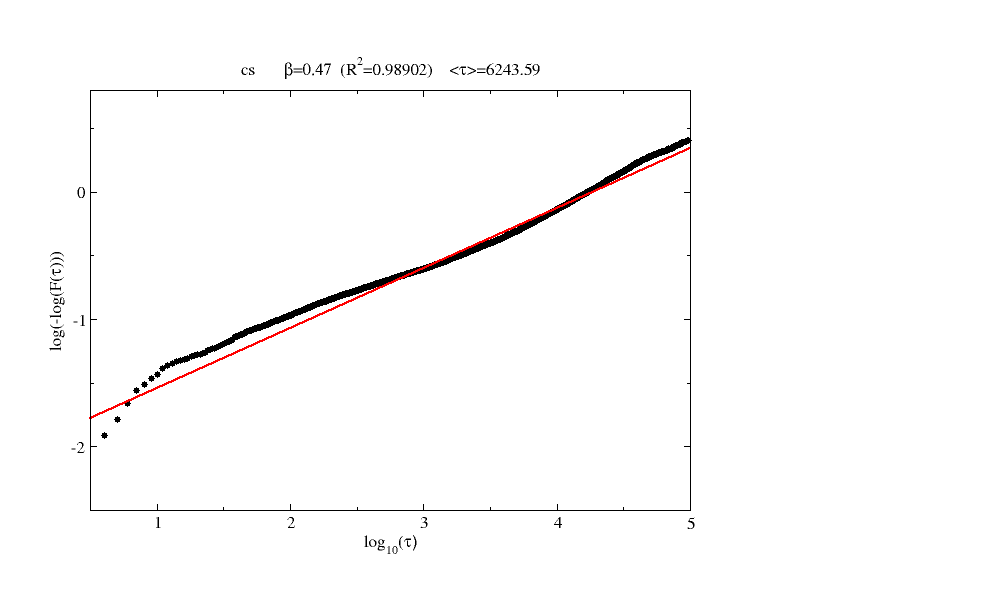

Supplement: Table S1 — Detailed information on the statistical analysis of all words that were studied (six databases). (31.88 MB TAR) [file pone.0007678.s002.tar › recurrence/comp/cs.png]

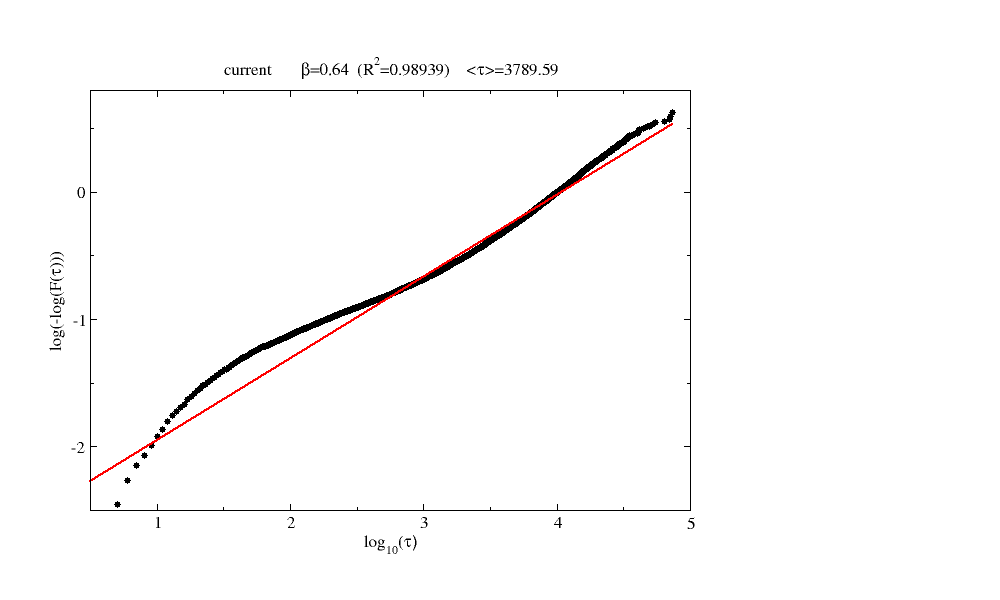

Supplement: Table S1 — Detailed information on the statistical analysis of all words that were studied (six databases). (31.88 MB TAR) [file pone.0007678.s002.tar › recurrence/comp/current.png]

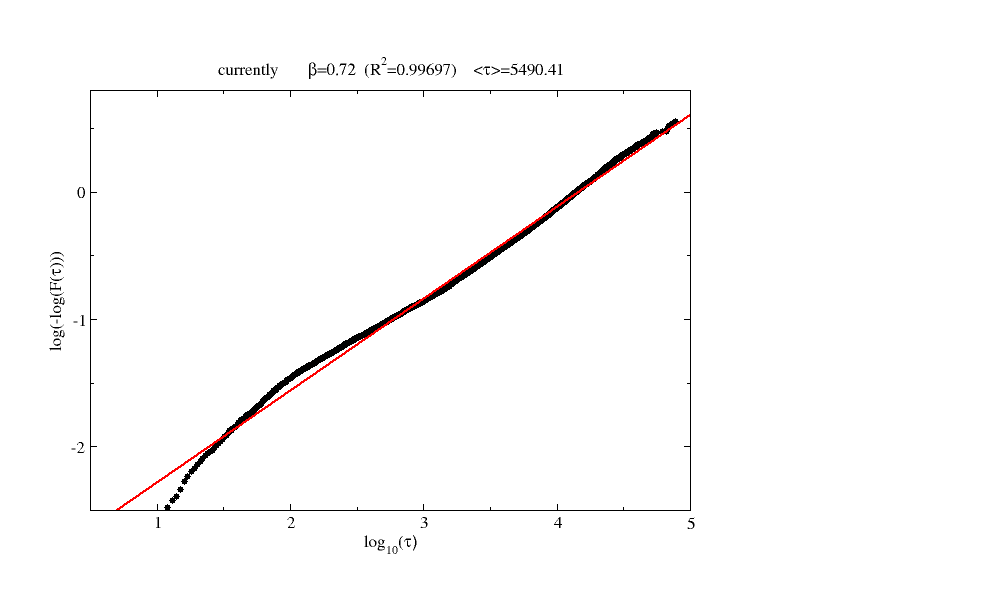

Supplement: Table S1 — Detailed information on the statistical analysis of all words that were studied (six databases). (31.88 MB TAR) [file pone.0007678.s002.tar › recurrence/comp/currently.png]

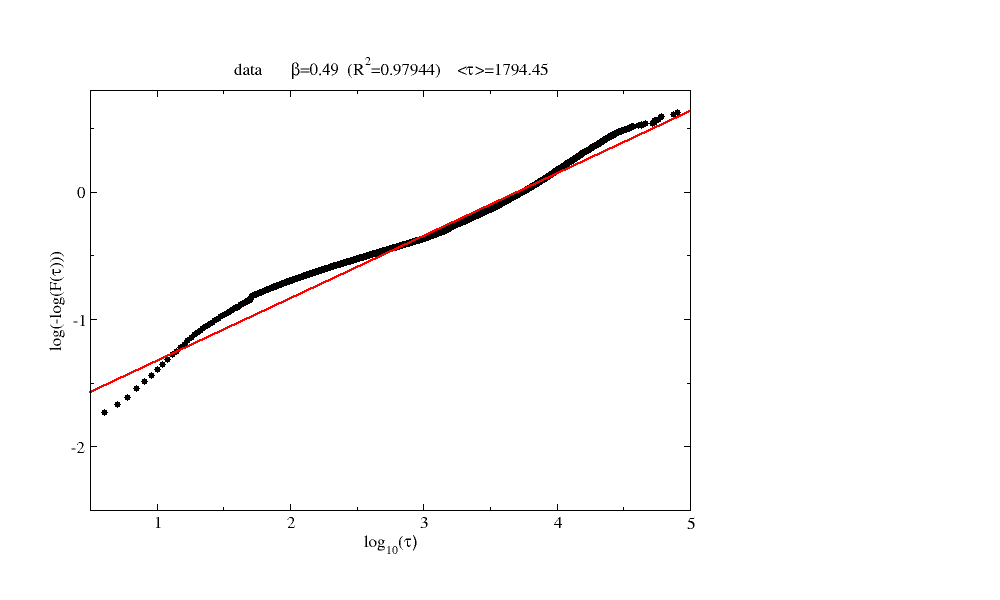

Supplement: Table S1 — Detailed information on the statistical analysis of all words that were studied (six databases). (31.88 MB TAR) [file pone.0007678.s002.tar › recurrence/comp/data.png]

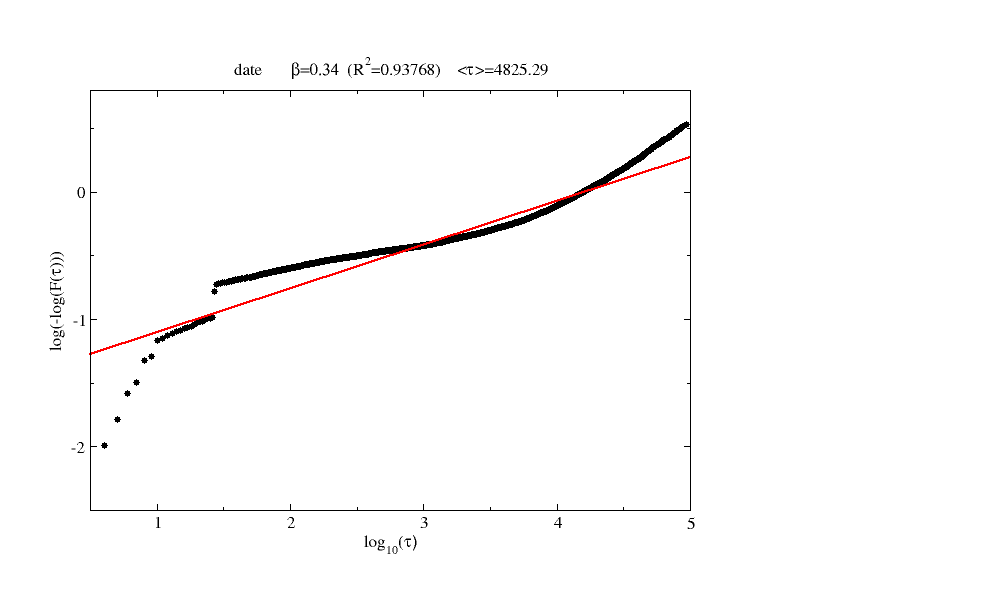

Supplement: Table S1 — Detailed information on the statistical analysis of all words that were studied (six databases). (31.88 MB TAR) [file pone.0007678.s002.tar › recurrence/comp/date.png]

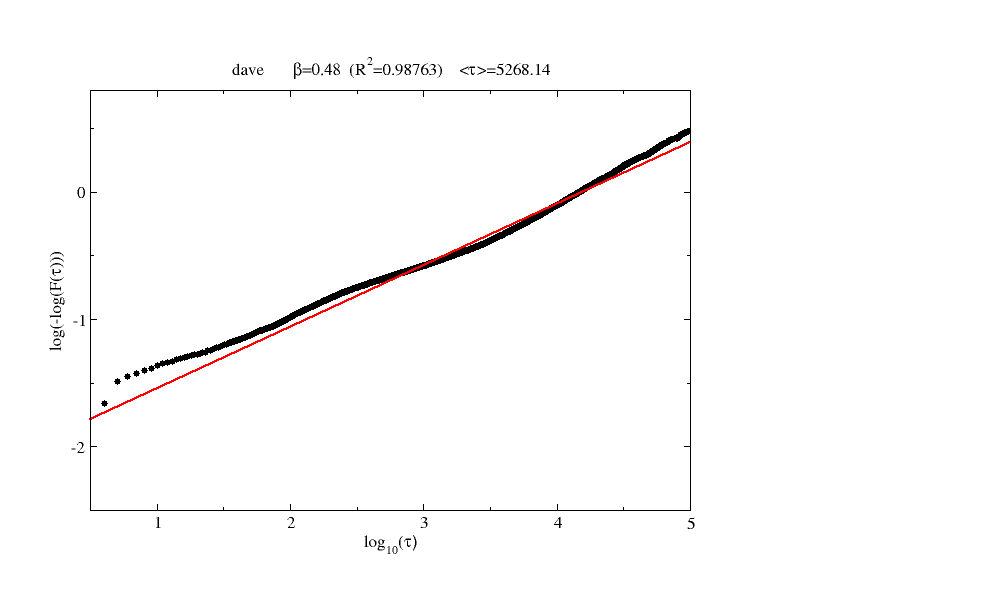

Supplement: Table S1 — Detailed information on the statistical analysis of all words that were studied (six databases). (31.88 MB TAR) [file pone.0007678.s002.tar › recurrence/comp/dave.png]

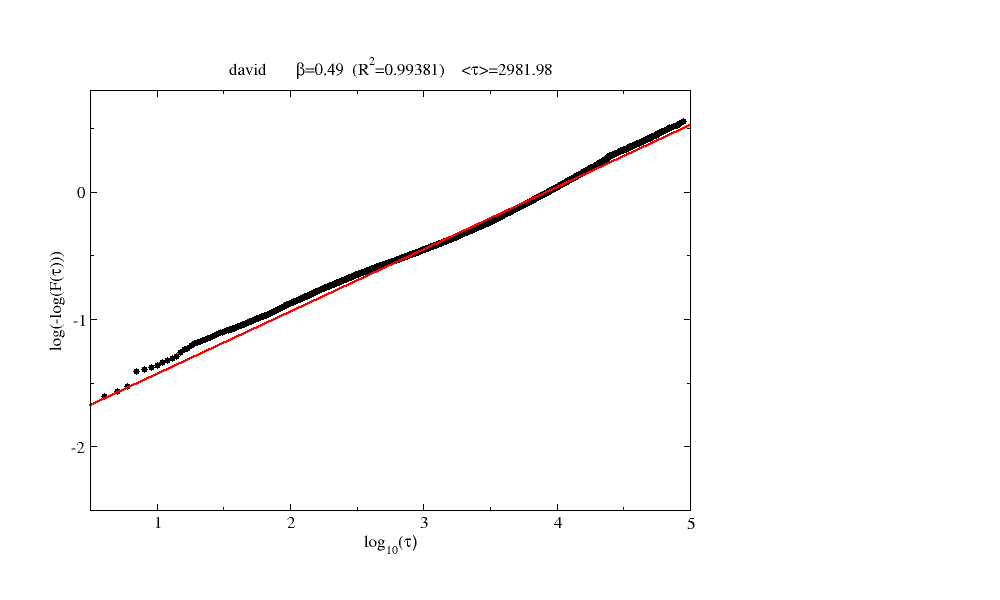

Supplement: Table S1 — Detailed information on the statistical analysis of all words that were studied (six databases). (31.88 MB TAR) [file pone.0007678.s002.tar › recurrence/comp/david.png]

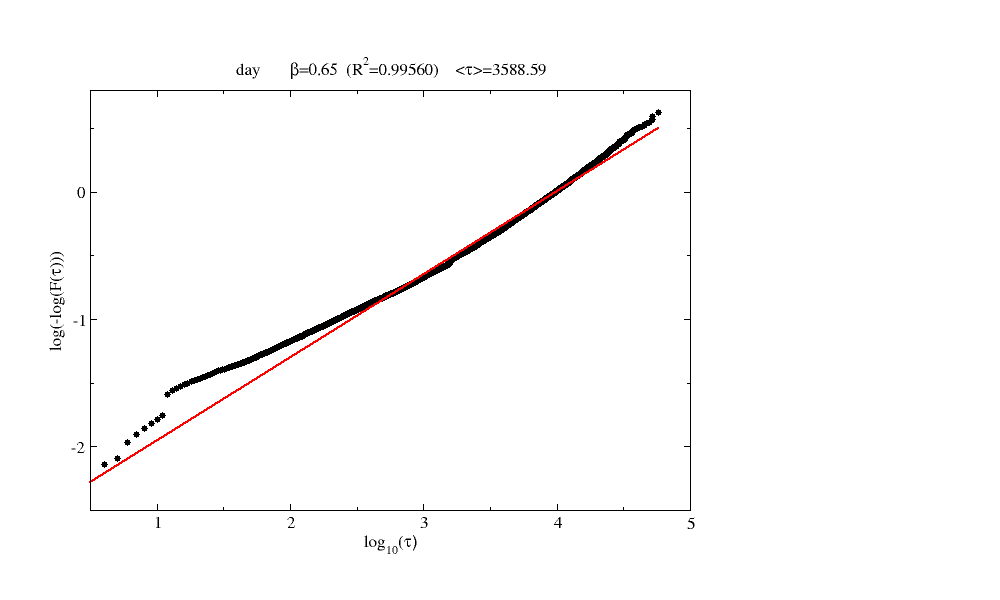

Supplement: Table S1 — Detailed information on the statistical analysis of all words that were studied (six databases). (31.88 MB TAR) [file pone.0007678.s002.tar › recurrence/comp/day.png]

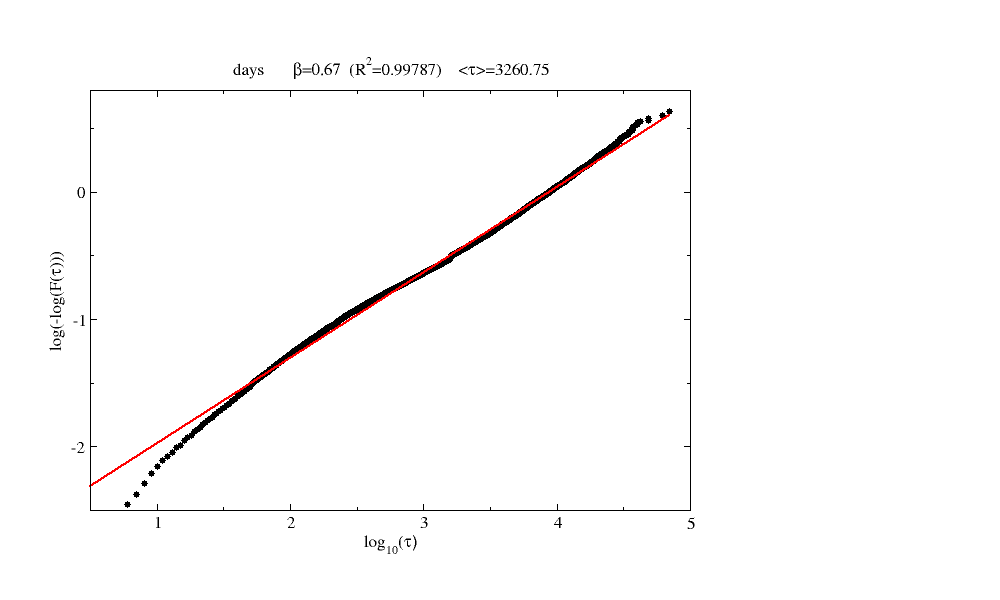

Supplement: Table S1 — Detailed information on the statistical analysis of all words that were studied (six databases). (31.88 MB TAR) [file pone.0007678.s002.tar › recurrence/comp/days.png]

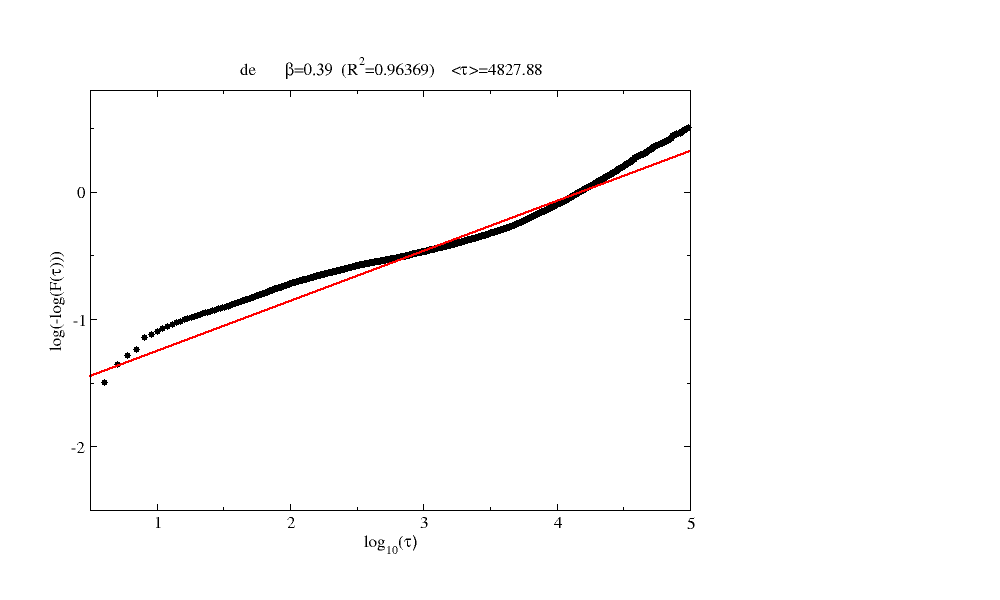

Supplement: Table S1 — Detailed information on the statistical analysis of all words that were studied (six databases). (31.88 MB TAR) [file pone.0007678.s002.tar › recurrence/comp/de.png]

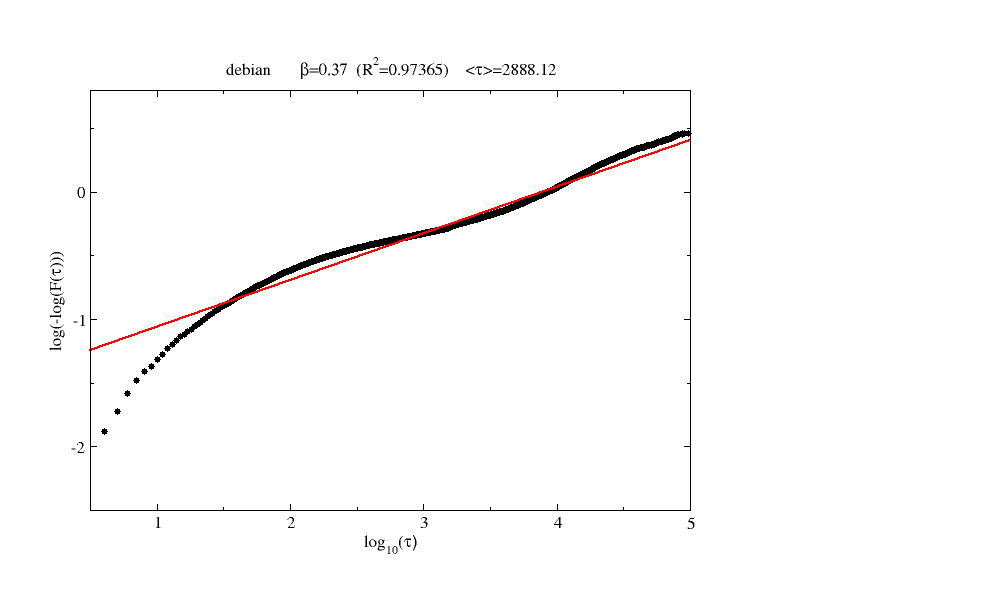

Supplement: Table S1 — Detailed information on the statistical analysis of all words that were studied (six databases). (31.88 MB TAR) [file pone.0007678.s002.tar › recurrence/comp/debian.png]

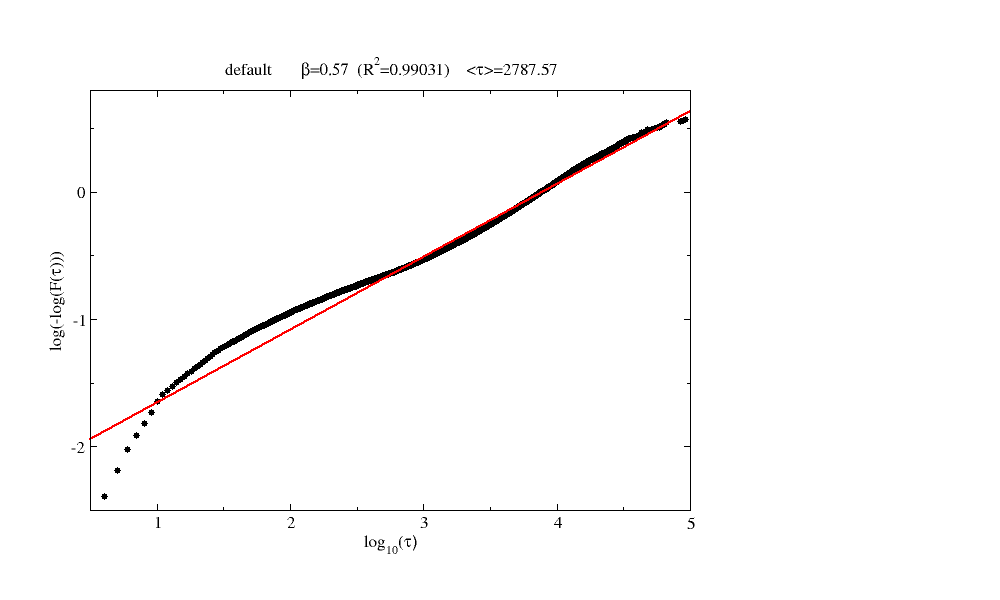

Supplement: Table S1 — Detailed information on the statistical analysis of all words that were studied (six databases). (31.88 MB TAR) [file pone.0007678.s002.tar › recurrence/comp/default.png]

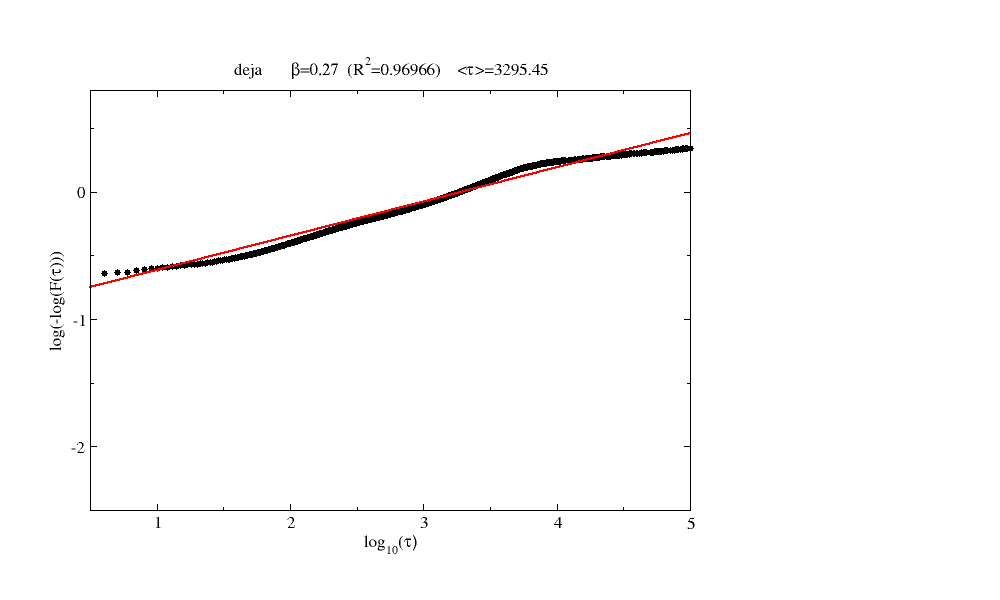

Supplement: Table S1 — Detailed information on the statistical analysis of all words that were studied (six databases). (31.88 MB TAR) [file pone.0007678.s002.tar › recurrence/comp/deja.png]

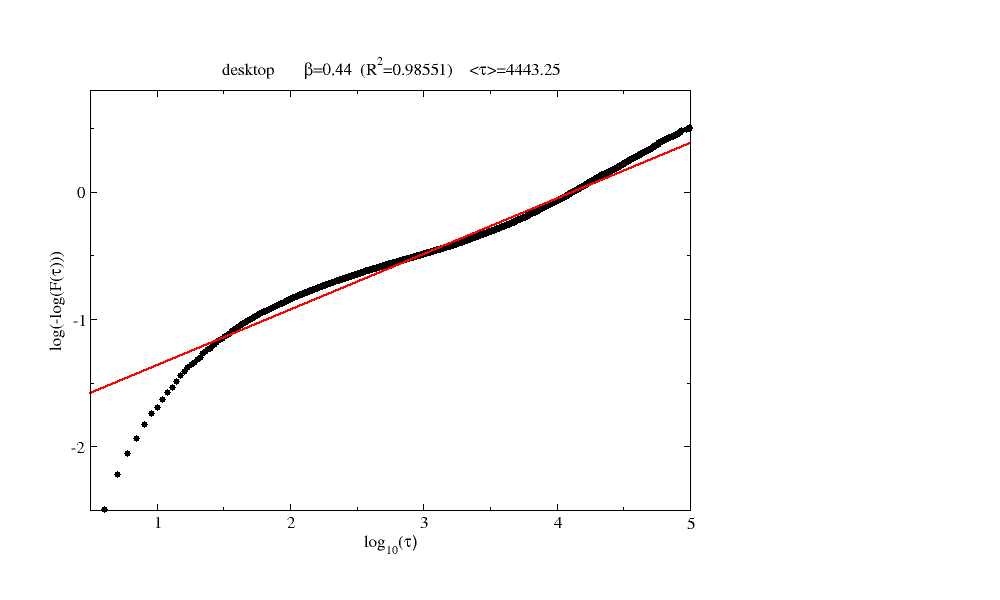

Supplement: Table S1 — Detailed information on the statistical analysis of all words that were studied (six databases). (31.88 MB TAR) [file pone.0007678.s002.tar › recurrence/comp/desktop.png]

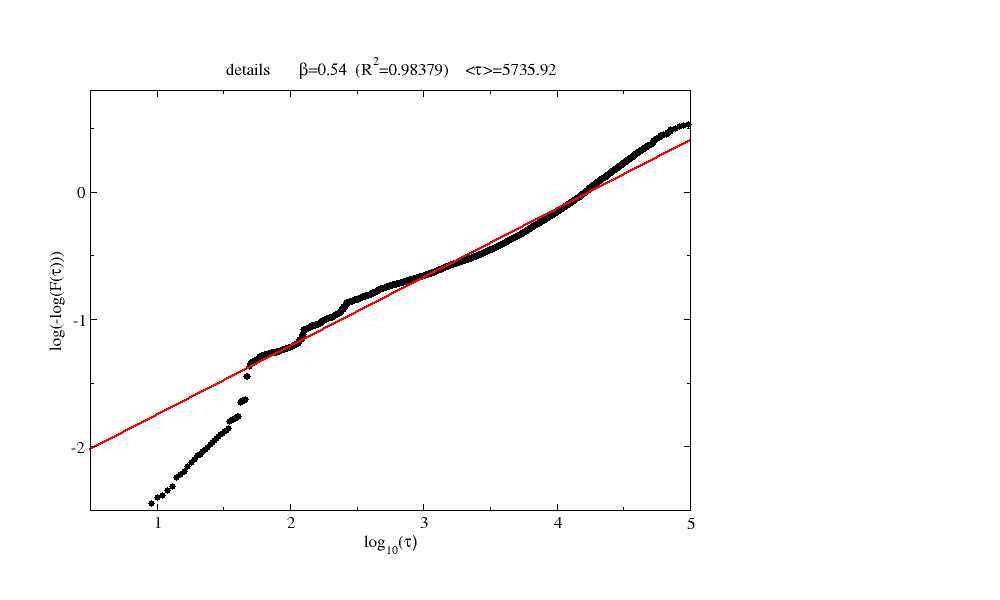

Supplement: Table S1 — Detailed information on the statistical analysis of all words that were studied (six databases). (31.88 MB TAR) [file pone.0007678.s002.tar › recurrence/comp/details.png]

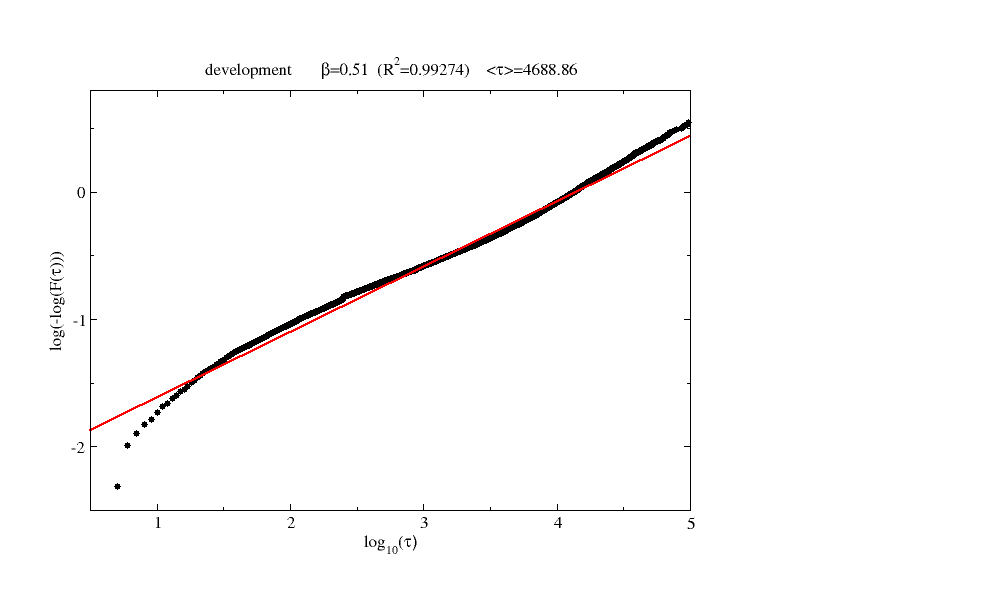

Supplement: Table S1 — Detailed information on the statistical analysis of all words that were studied (six databases). (31.88 MB TAR) [file pone.0007678.s002.tar › recurrence/comp/development.png]

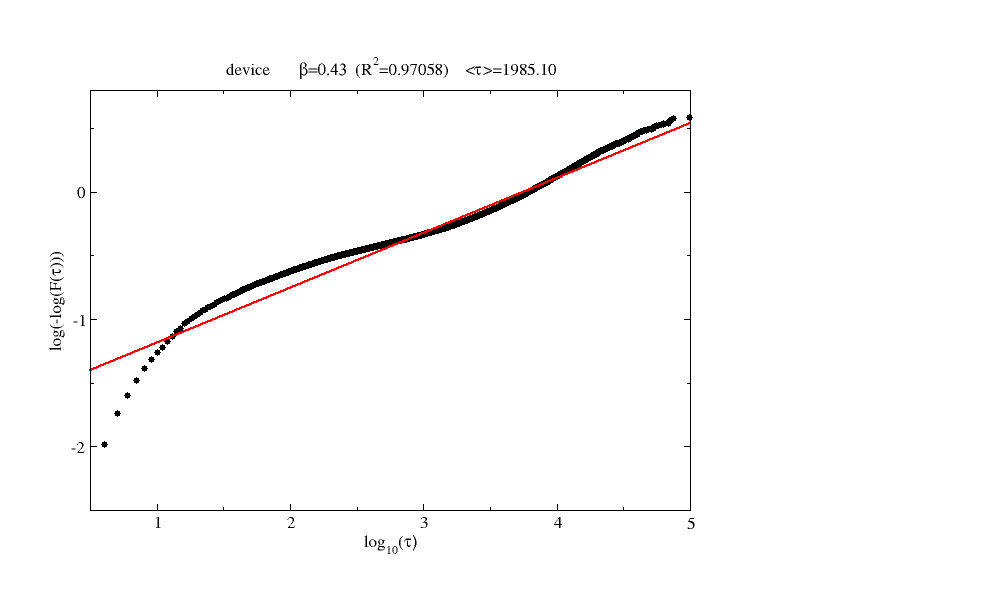

Supplement: Table S1 — Detailed information on the statistical analysis of all words that were studied (six databases). (31.88 MB TAR) [file pone.0007678.s002.tar › recurrence/comp/device.png]

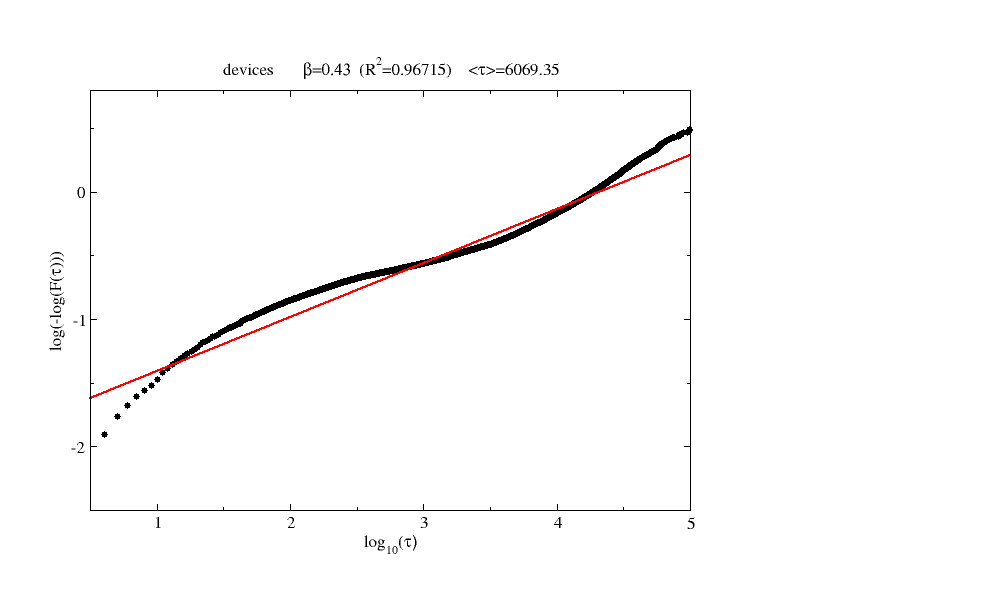

Supplement: Table S1 — Detailed information on the statistical analysis of all words that were studied (six databases). (31.88 MB TAR) [file pone.0007678.s002.tar › recurrence/comp/devices.png]

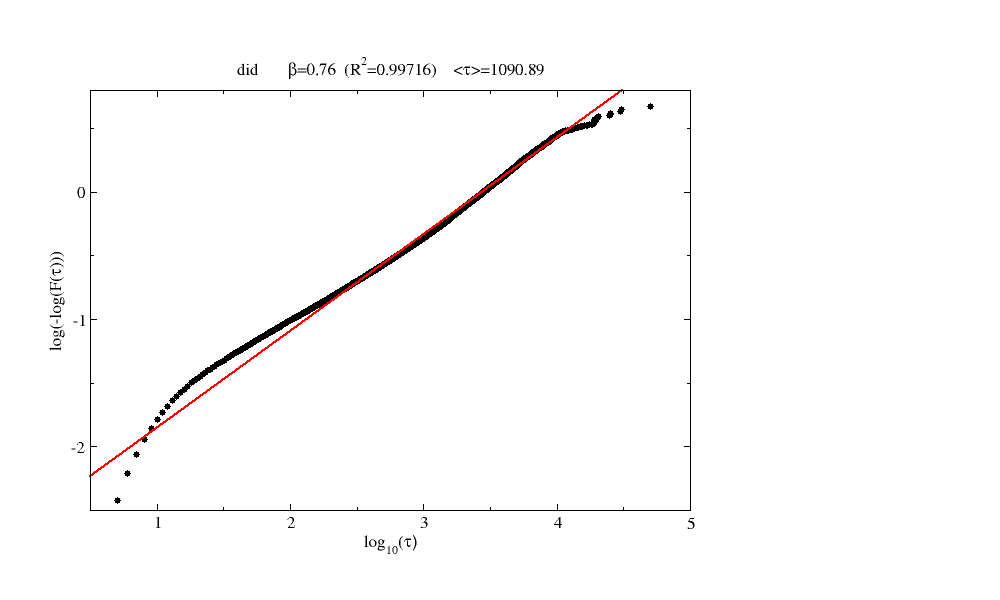

Supplement: Table S1 — Detailed information on the statistical analysis of all words that were studied (six databases). (31.88 MB TAR) [file pone.0007678.s002.tar › recurrence/comp/did.png]

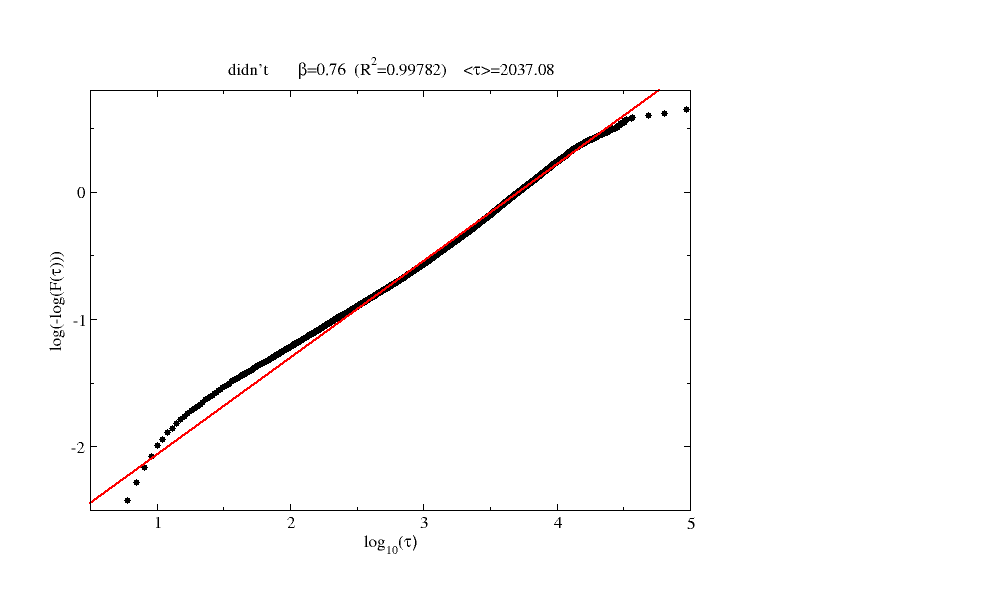

Supplement: Table S1 — Detailed information on the statistical analysis of all words that were studied (six databases). (31.88 MB TAR) [file pone.0007678.s002.tar › recurrence/comp/didn't.png]

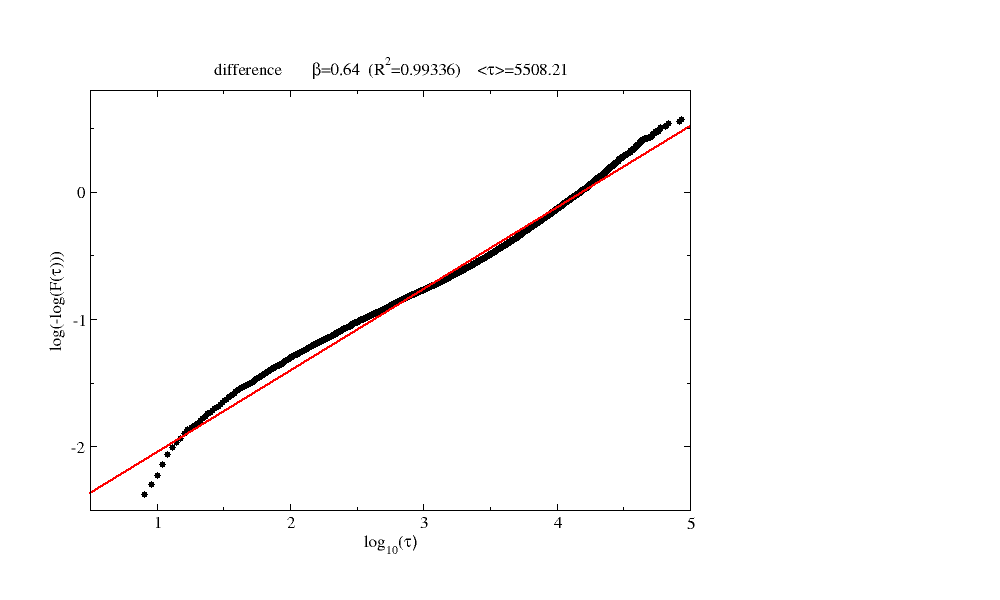

Supplement: Table S1 — Detailed information on the statistical analysis of all words that were studied (six databases). (31.88 MB TAR) [file pone.0007678.s002.tar › recurrence/comp/difference.png]

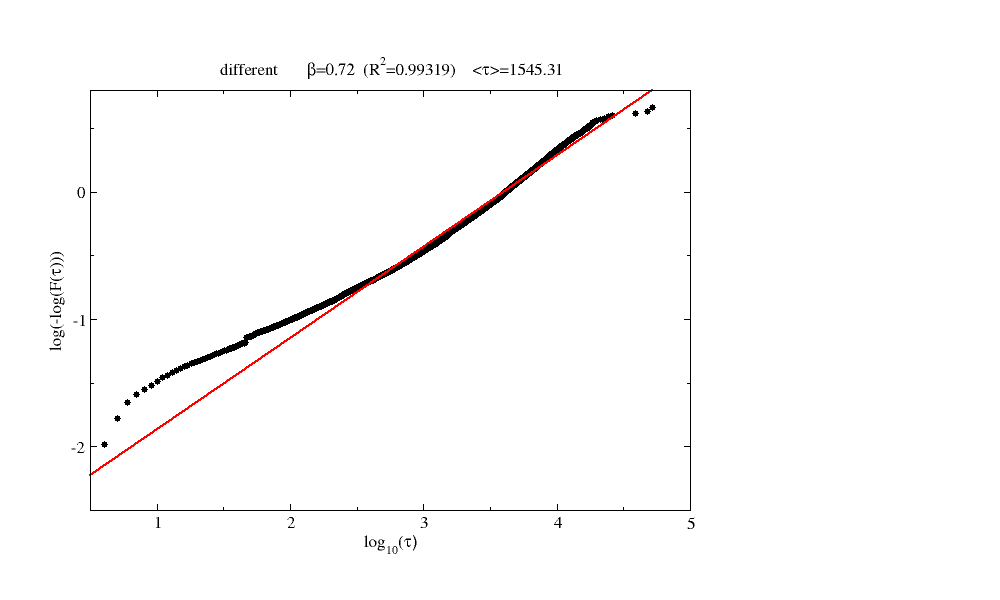

Supplement: Table S1 — Detailed information on the statistical analysis of all words that were studied (six databases). (31.88 MB TAR) [file pone.0007678.s002.tar › recurrence/comp/different.png]

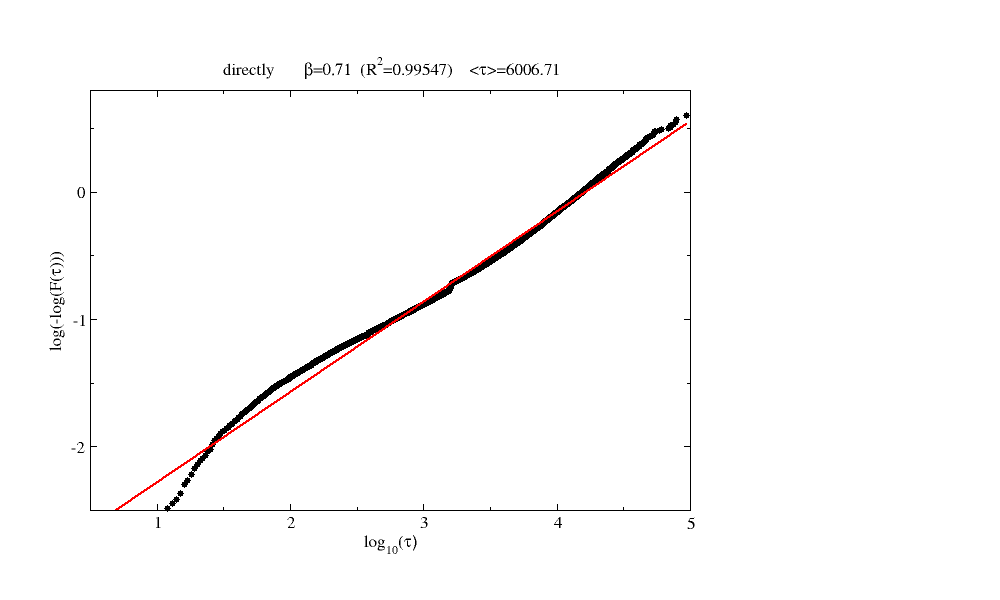

Supplement: Table S1 — Detailed information on the statistical analysis of all words that were studied (six databases). (31.88 MB TAR) [file pone.0007678.s002.tar › recurrence/comp/directly.png]

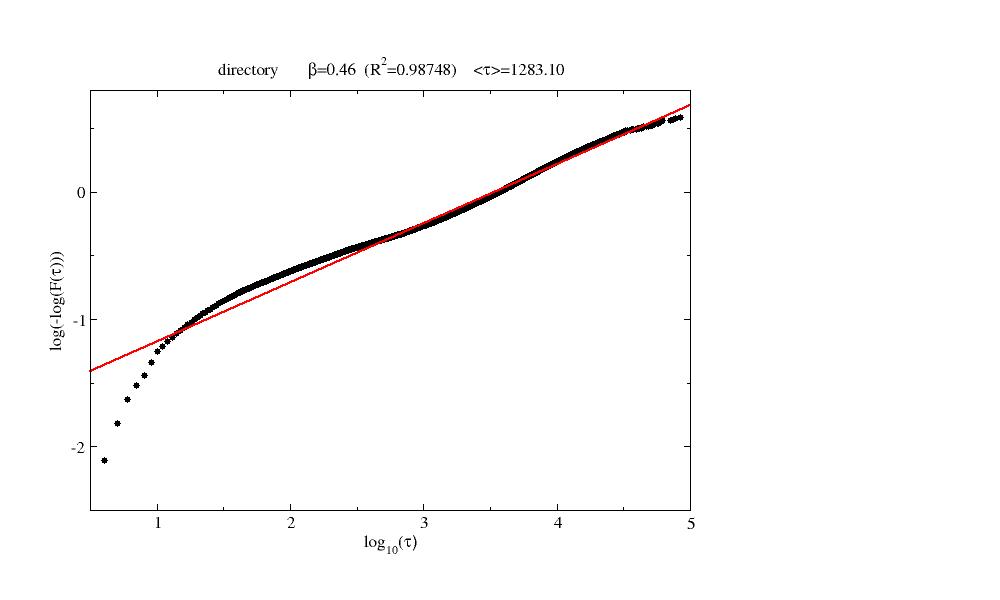

Supplement: Table S1 — Detailed information on the statistical analysis of all words that were studied (six databases). (31.88 MB TAR) [file pone.0007678.s002.tar › recurrence/comp/directory.png]

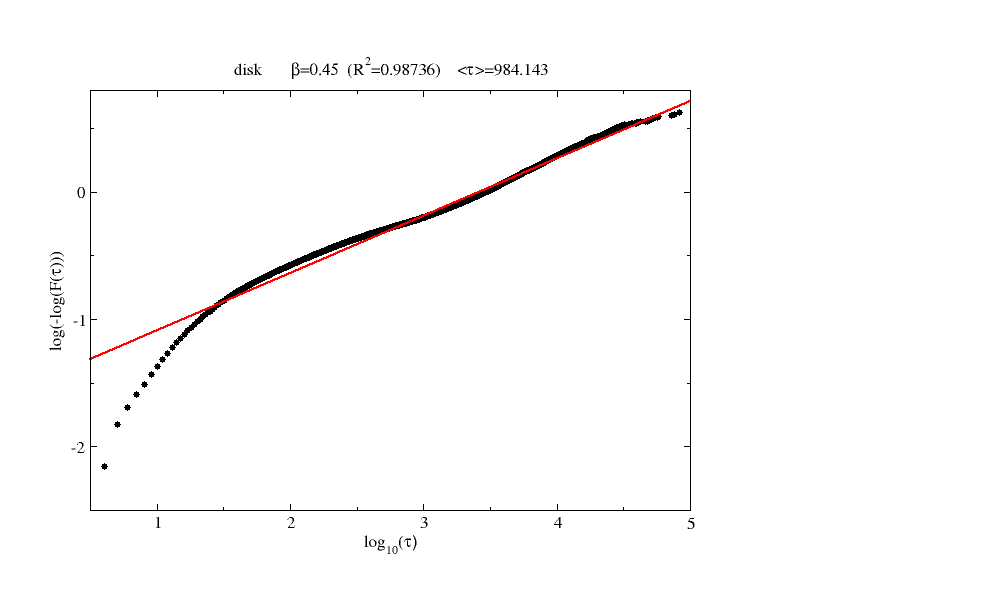

Supplement: Table S1 — Detailed information on the statistical analysis of all words that were studied (six databases). (31.88 MB TAR) [file pone.0007678.s002.tar › recurrence/comp/disk.png]

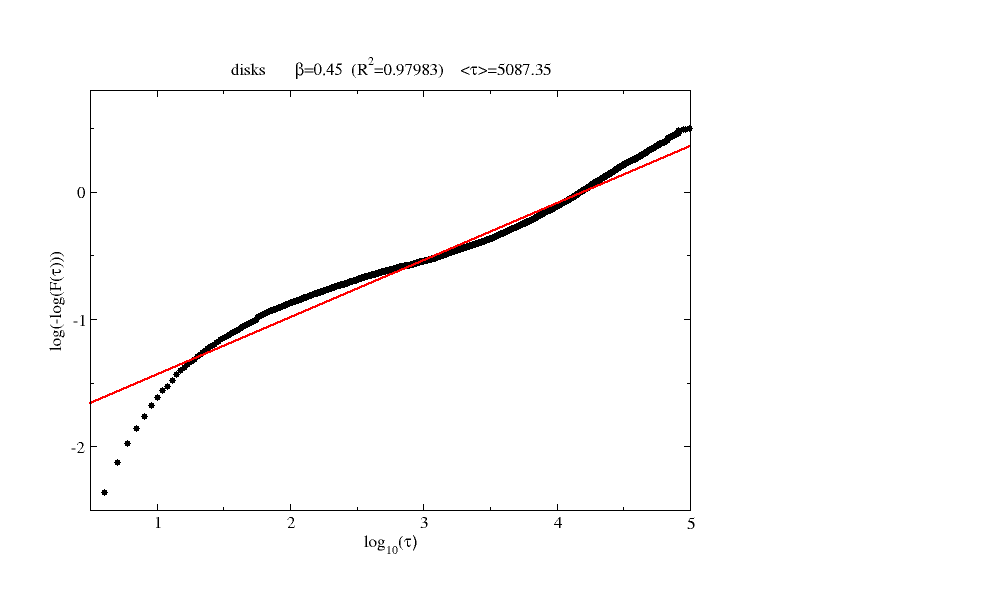

Supplement: Table S1 — Detailed information on the statistical analysis of all words that were studied (six databases). (31.88 MB TAR) [file pone.0007678.s002.tar › recurrence/comp/disks.png]

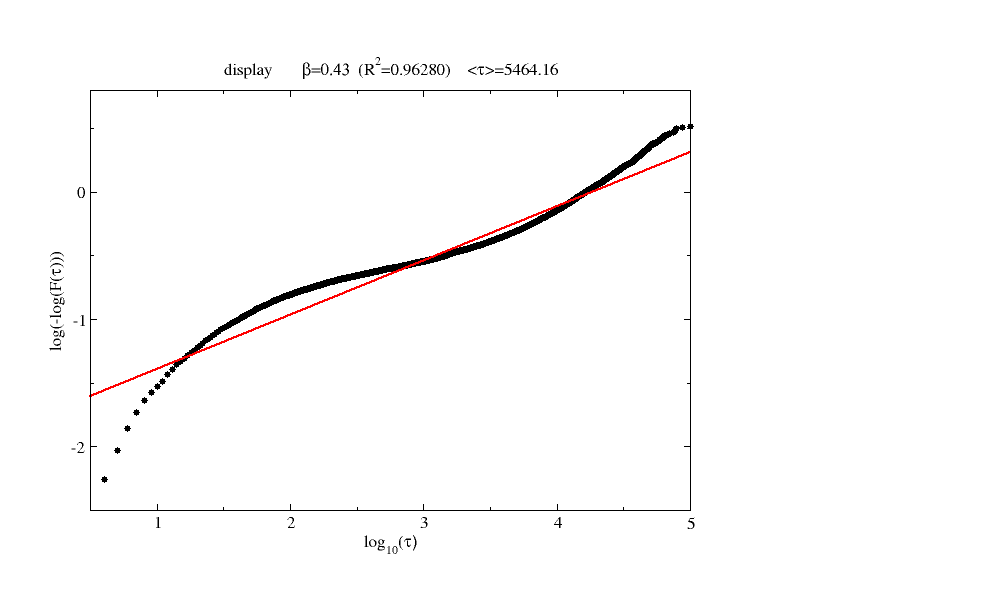

Supplement: Table S1 — Detailed information on the statistical analysis of all words that were studied (six databases). (31.88 MB TAR) [file pone.0007678.s002.tar › recurrence/comp/display.png]

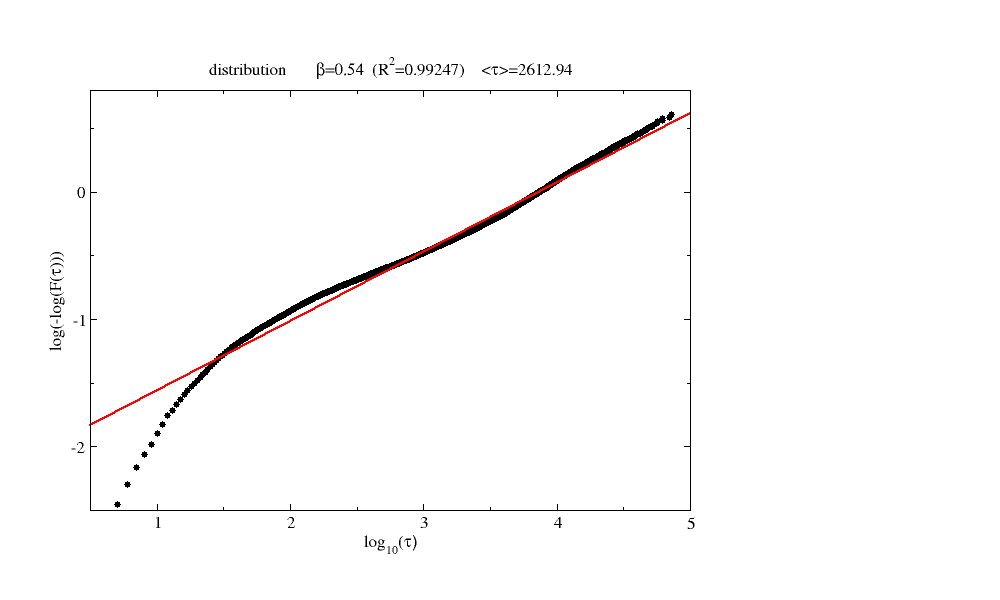

Supplement: Table S1 — Detailed information on the statistical analysis of all words that were studied (six databases). (31.88 MB TAR) [file pone.0007678.s002.tar › recurrence/comp/distribution.png]

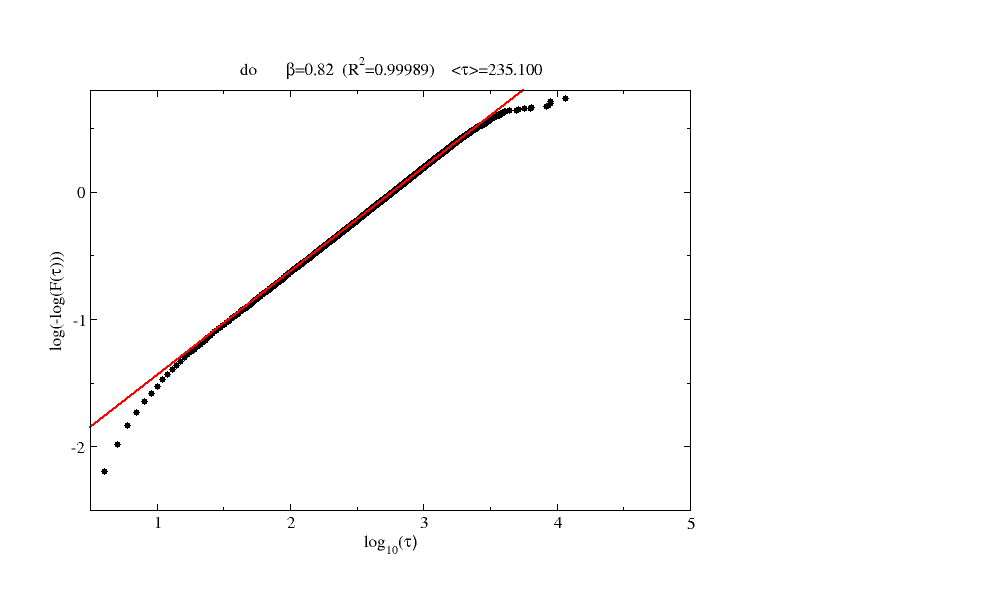

Supplement: Table S1 — Detailed information on the statistical analysis of all words that were studied (six databases). (31.88 MB TAR) [file pone.0007678.s002.tar › recurrence/comp/do.png]

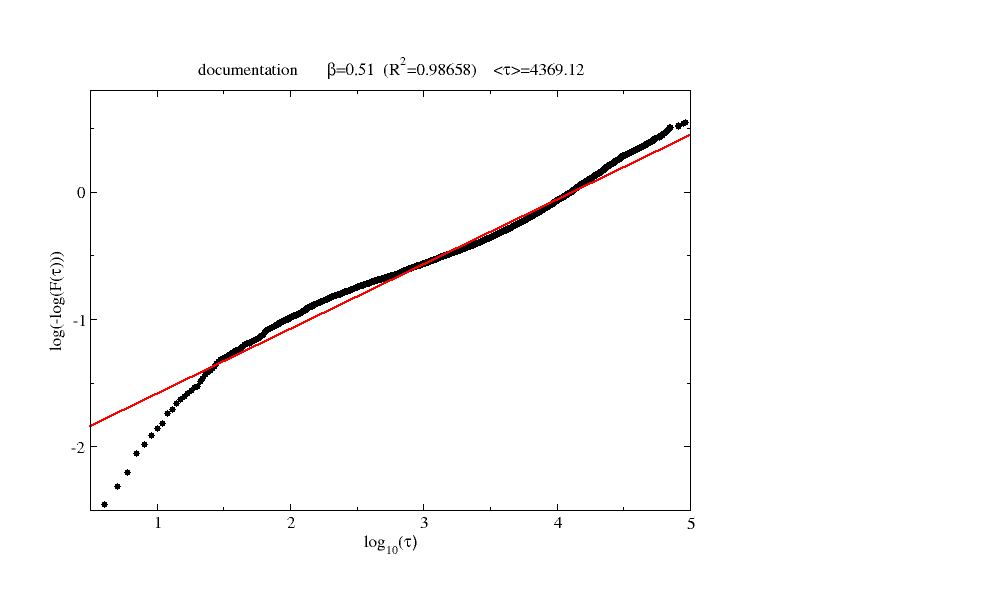

Supplement: Table S1 — Detailed information on the statistical analysis of all words that were studied (six databases). (31.88 MB TAR) [file pone.0007678.s002.tar › recurrence/comp/documentation.png]

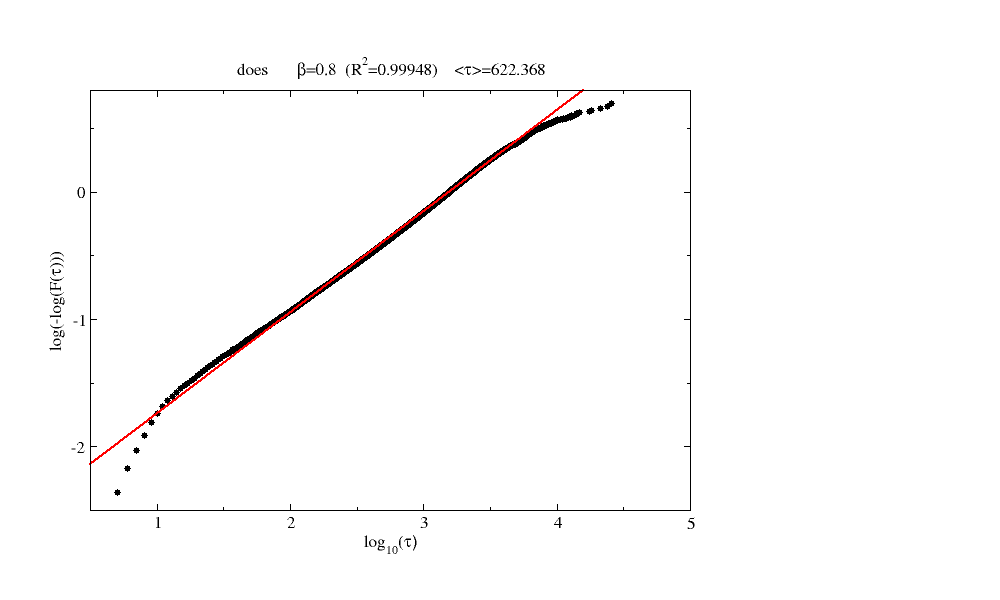

Supplement: Table S1 — Detailed information on the statistical analysis of all words that were studied (six databases). (31.88 MB TAR) [file pone.0007678.s002.tar › recurrence/comp/does.png]

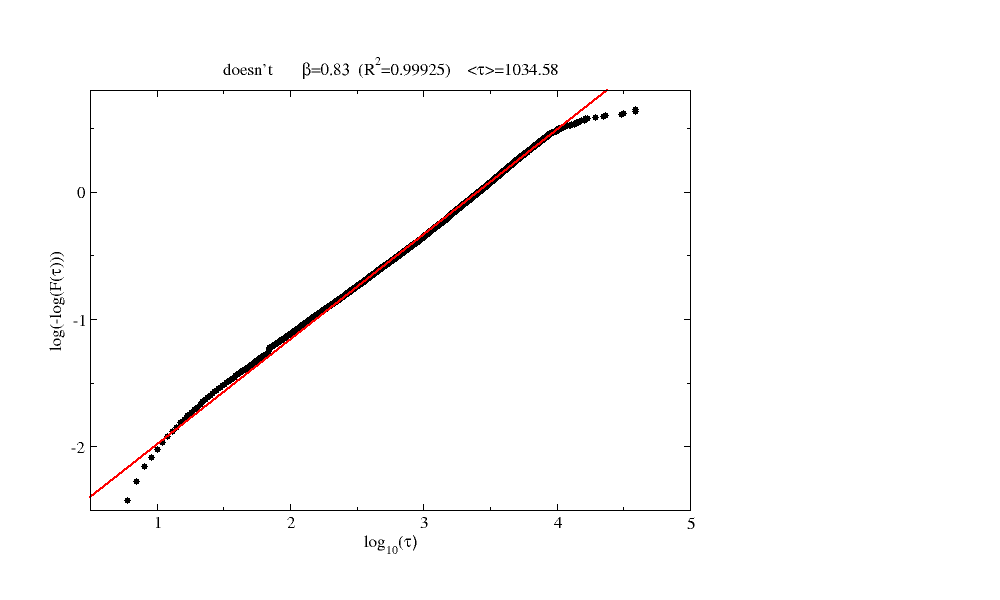

Supplement: Table S1 — Detailed information on the statistical analysis of all words that were studied (six databases). (31.88 MB TAR) [file pone.0007678.s002.tar › recurrence/comp/doesn't.png]

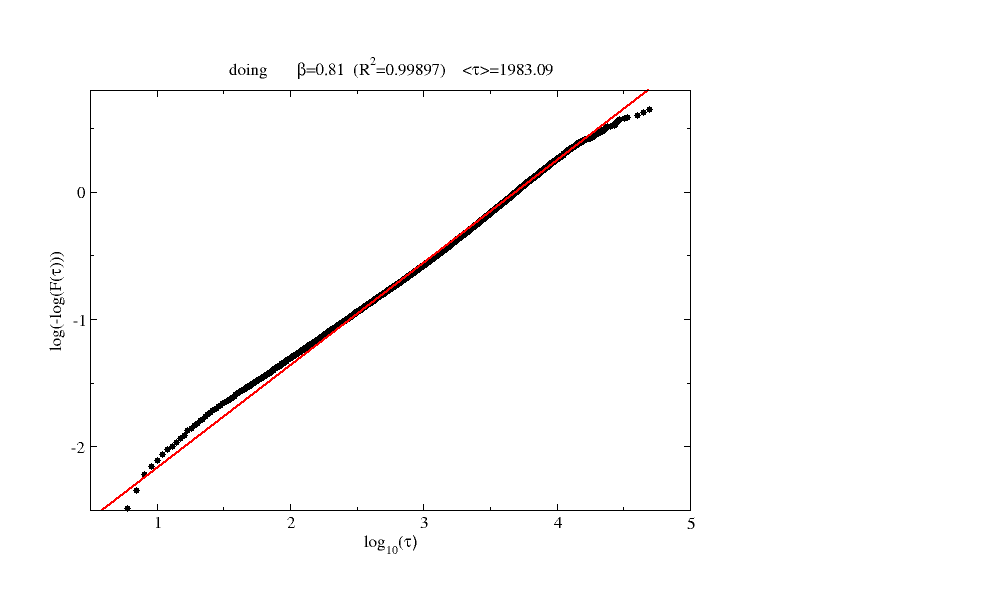

Supplement: Table S1 — Detailed information on the statistical analysis of all words that were studied (six databases). (31.88 MB TAR) [file pone.0007678.s002.tar › recurrence/comp/doing.png]

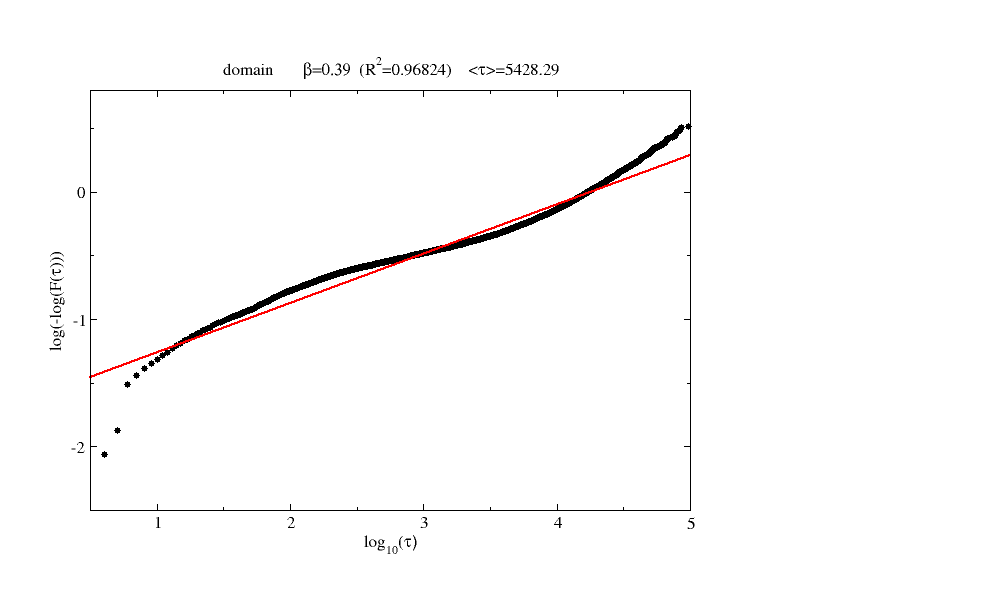

Supplement: Table S1 — Detailed information on the statistical analysis of all words that were studied (six databases). (31.88 MB TAR) [file pone.0007678.s002.tar › recurrence/comp/domain.png]

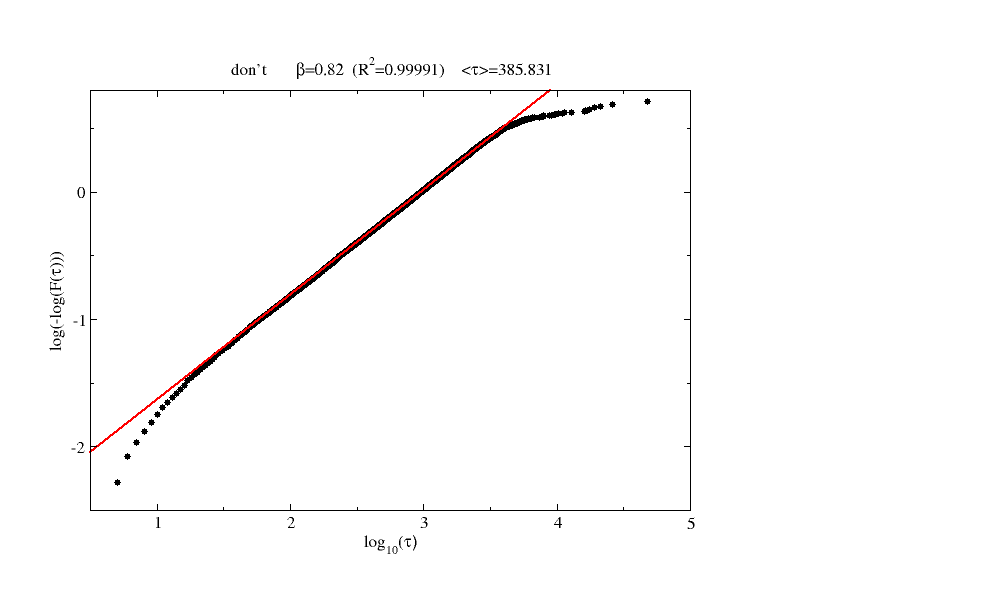

Supplement: Table S1 — Detailed information on the statistical analysis of all words that were studied (six databases). (31.88 MB TAR) [file pone.0007678.s002.tar › recurrence/comp/don't.png]

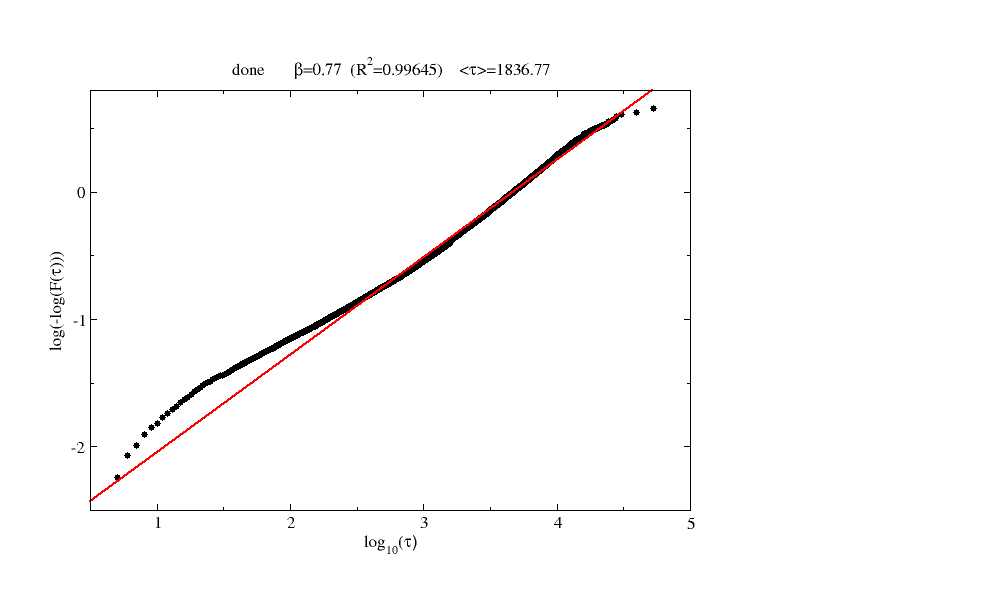

Supplement: Table S1 — Detailed information on the statistical analysis of all words that were studied (six databases). (31.88 MB TAR) [file pone.0007678.s002.tar › recurrence/comp/done.png]

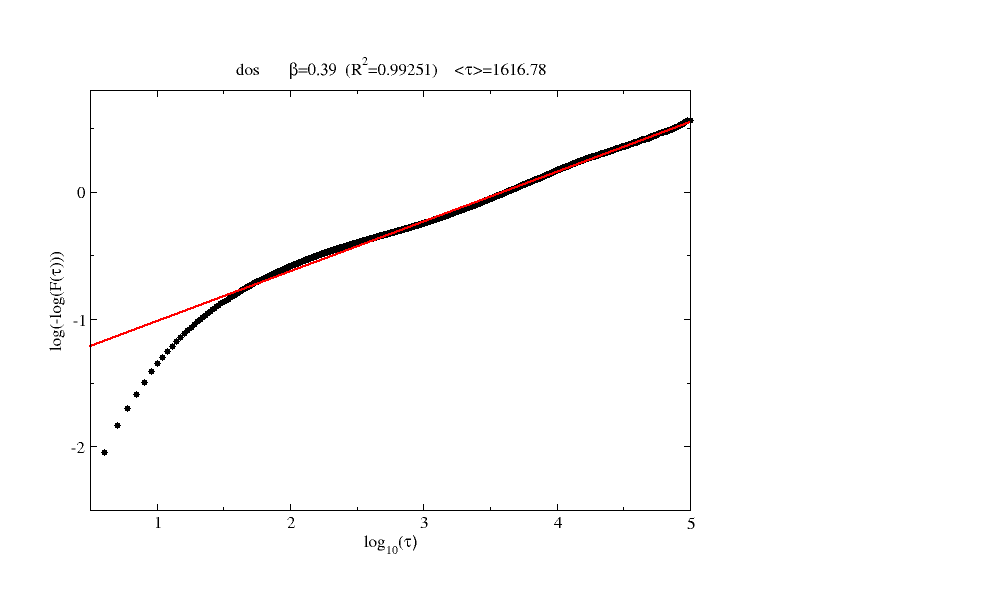

Supplement: Table S1 — Detailed information on the statistical analysis of all words that were studied (six databases). (31.88 MB TAR) [file pone.0007678.s002.tar › recurrence/comp/dos.png]

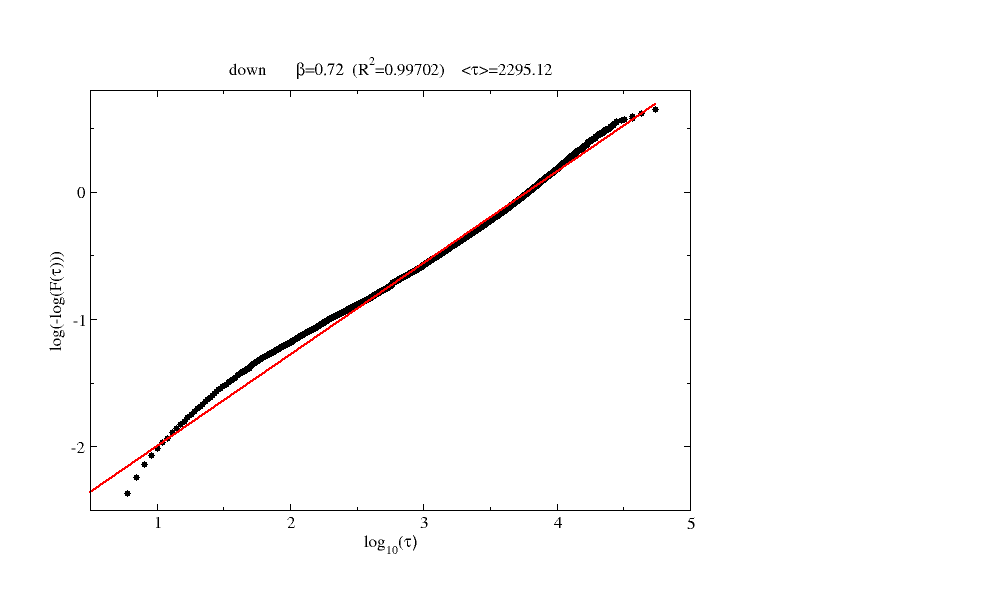

Supplement: Table S1 — Detailed information on the statistical analysis of all words that were studied (six databases). (31.88 MB TAR) [file pone.0007678.s002.tar › recurrence/comp/down.png]

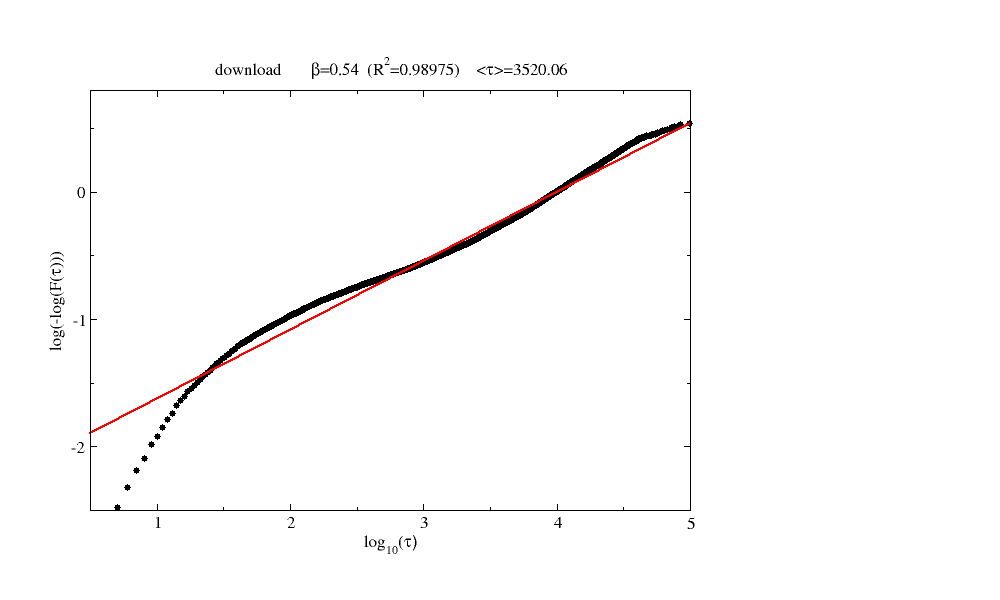

Supplement: Table S1 — Detailed information on the statistical analysis of all words that were studied (six databases). (31.88 MB TAR) [file pone.0007678.s002.tar › recurrence/comp/download.png]

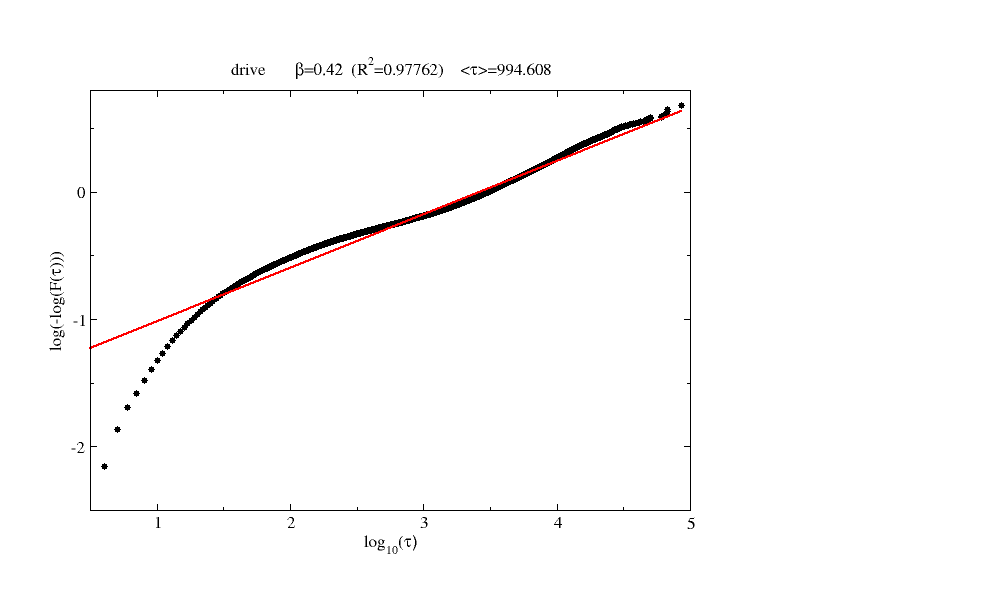

Supplement: Table S1 — Detailed information on the statistical analysis of all words that were studied (six databases). (31.88 MB TAR) [file pone.0007678.s002.tar › recurrence/comp/drive.png]

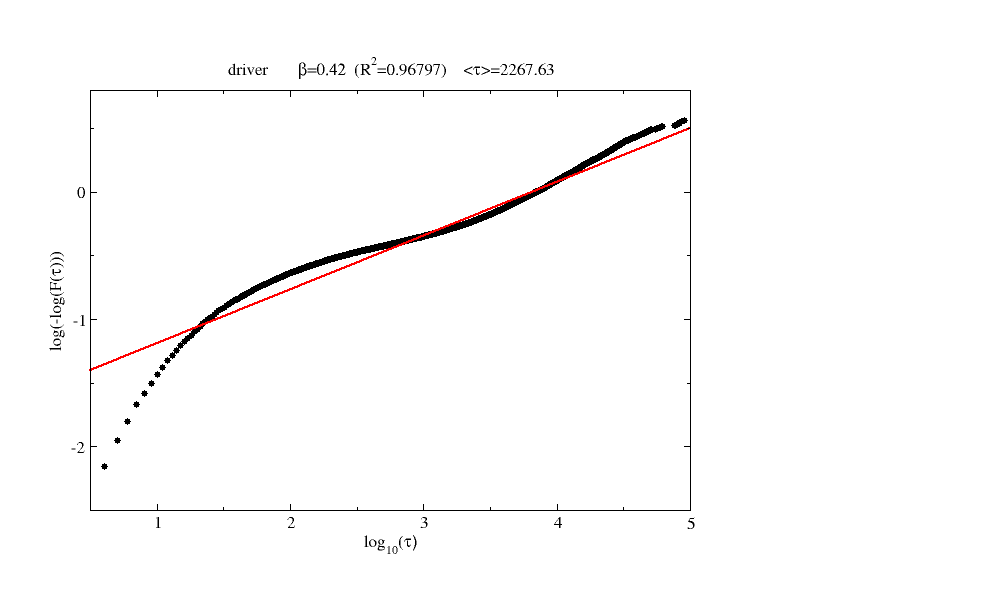

Supplement: Table S1 — Detailed information on the statistical analysis of all words that were studied (six databases). (31.88 MB TAR) [file pone.0007678.s002.tar › recurrence/comp/driver.png]

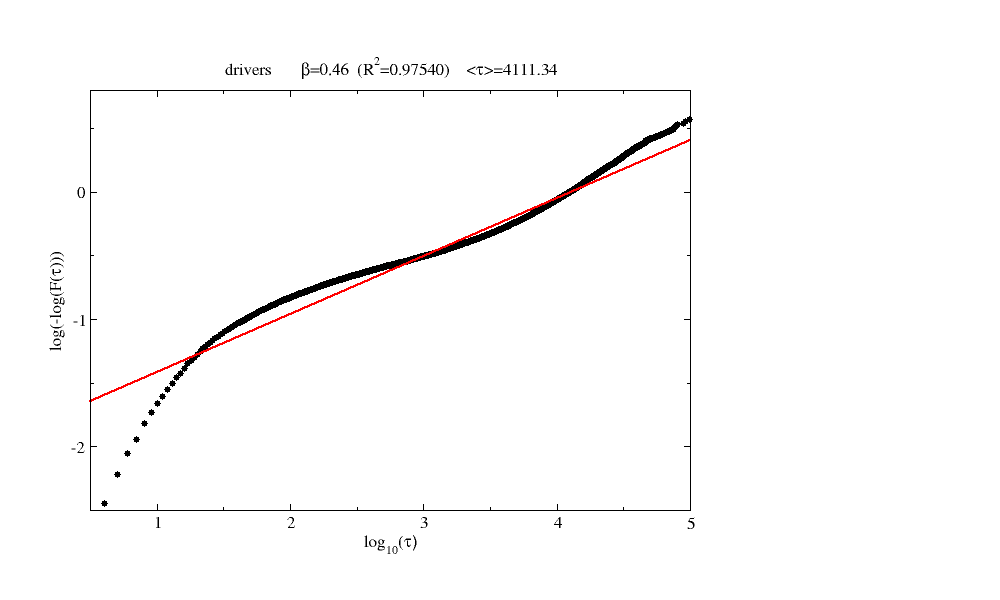

Supplement: Table S1 — Detailed information on the statistical analysis of all words that were studied (six databases). (31.88 MB TAR) [file pone.0007678.s002.tar › recurrence/comp/drivers.png]

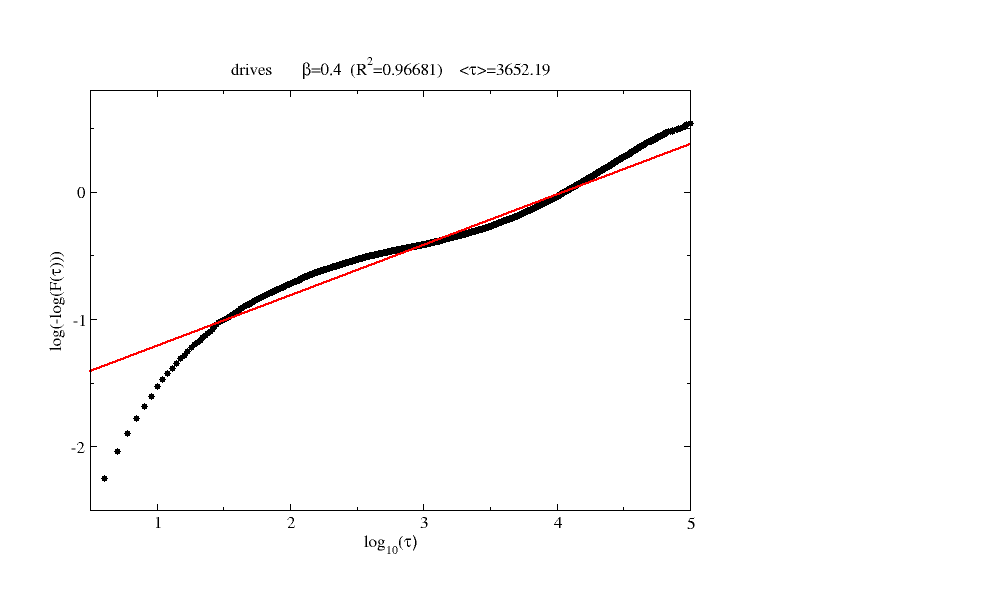

Supplement: Table S1 — Detailed information on the statistical analysis of all words that were studied (six databases). (31.88 MB TAR) [file pone.0007678.s002.tar › recurrence/comp/drives.png]

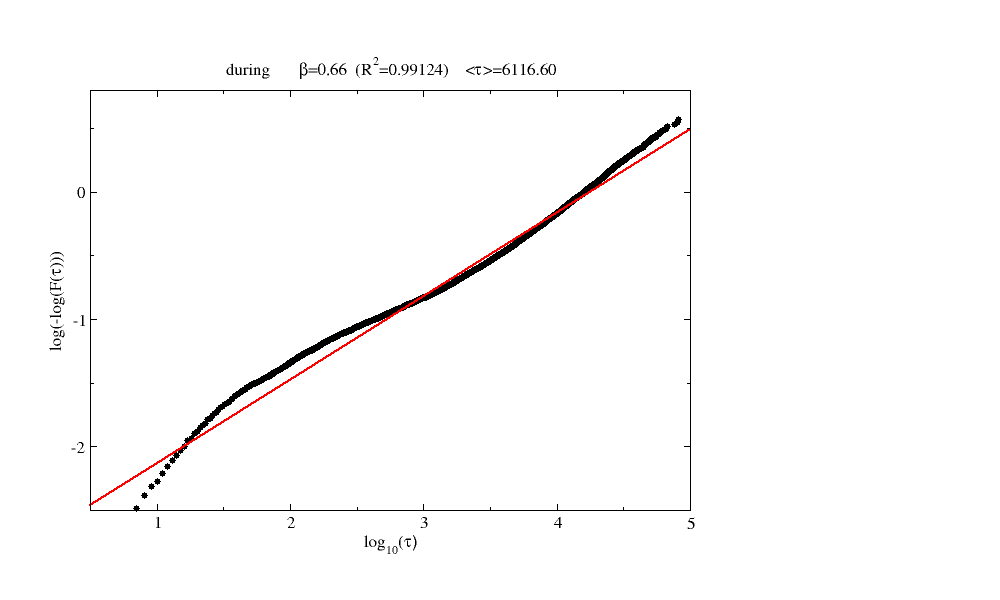

Supplement: Table S1 — Detailed information on the statistical analysis of all words that were studied (six databases). (31.88 MB TAR) [file pone.0007678.s002.tar › recurrence/comp/during.png]

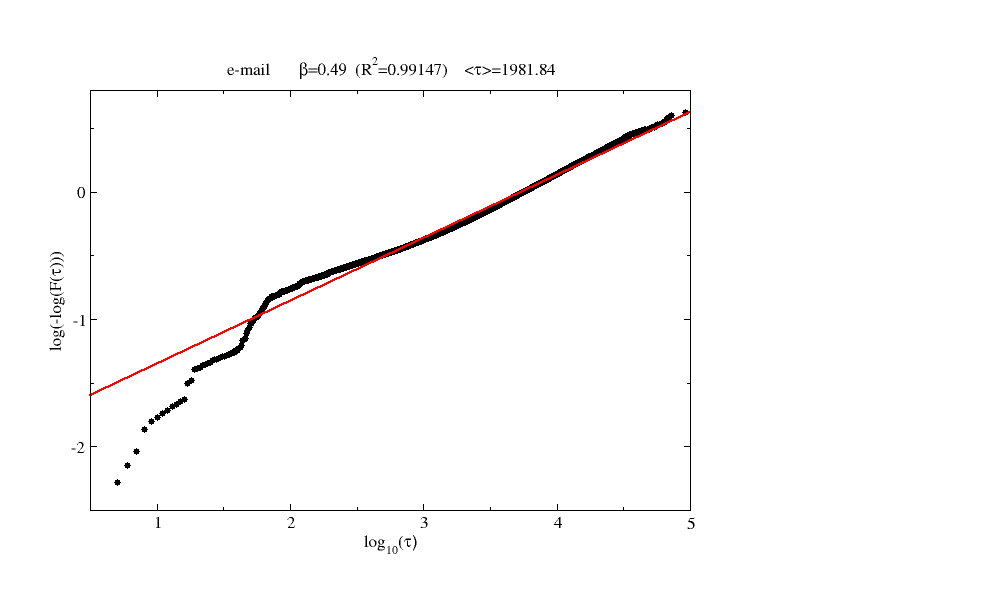

Supplement: Table S1 — Detailed information on the statistical analysis of all words that were studied (six databases). (31.88 MB TAR) [file pone.0007678.s002.tar › recurrence/comp/e-mail.png]

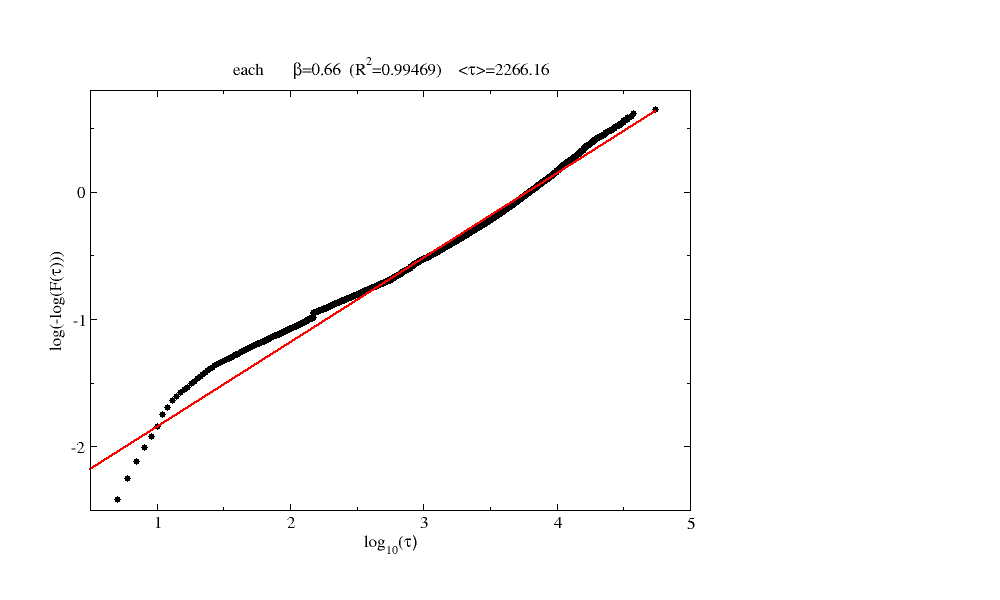

Supplement: Table S1 — Detailed information on the statistical analysis of all words that were studied (six databases). (31.88 MB TAR) [file pone.0007678.s002.tar › recurrence/comp/each.png]

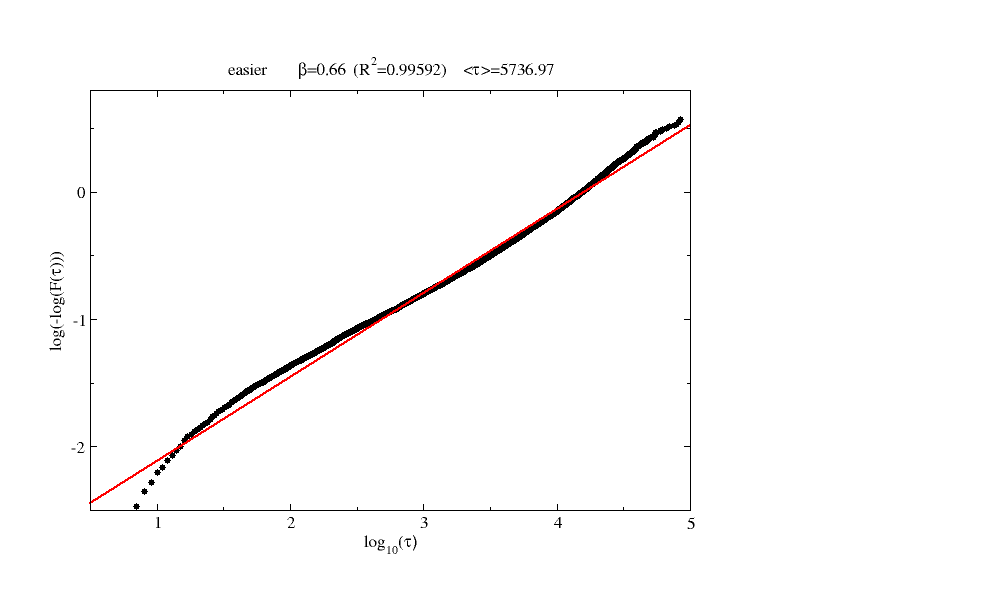

Supplement: Table S1 — Detailed information on the statistical analysis of all words that were studied (six databases). (31.88 MB TAR) [file pone.0007678.s002.tar › recurrence/comp/easier.png]

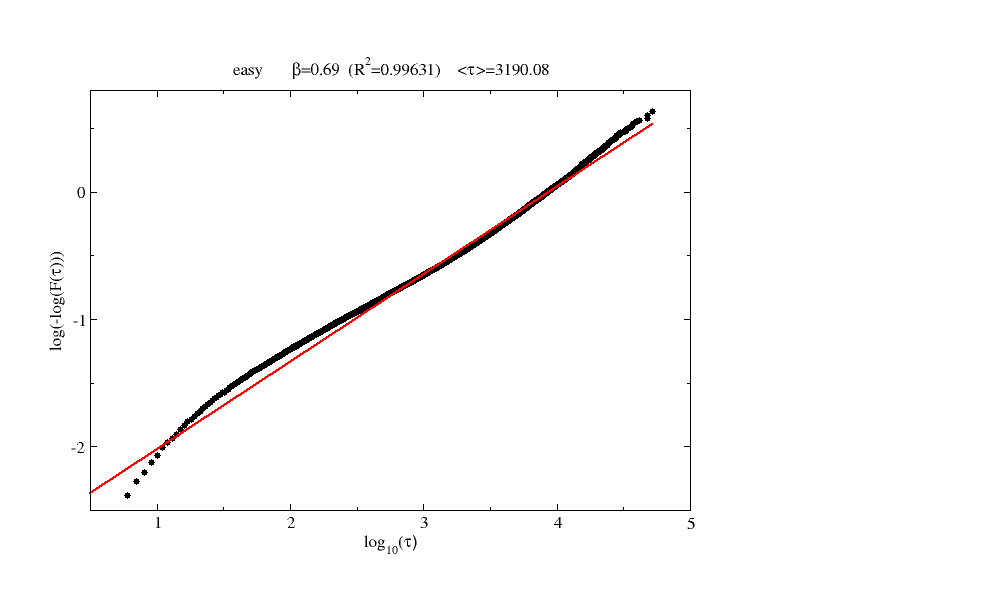

Supplement: Table S1 — Detailed information on the statistical analysis of all words that were studied (six databases). (31.88 MB TAR) [file pone.0007678.s002.tar › recurrence/comp/easy.png]

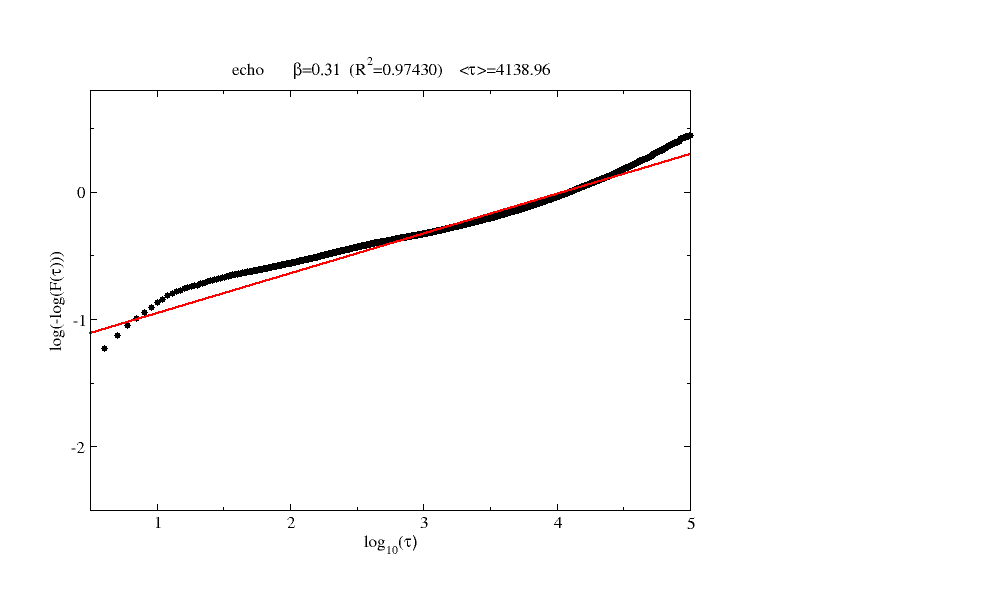

Supplement: Table S1 — Detailed information on the statistical analysis of all words that were studied (six databases). (31.88 MB TAR) [file pone.0007678.s002.tar › recurrence/comp/echo.png]

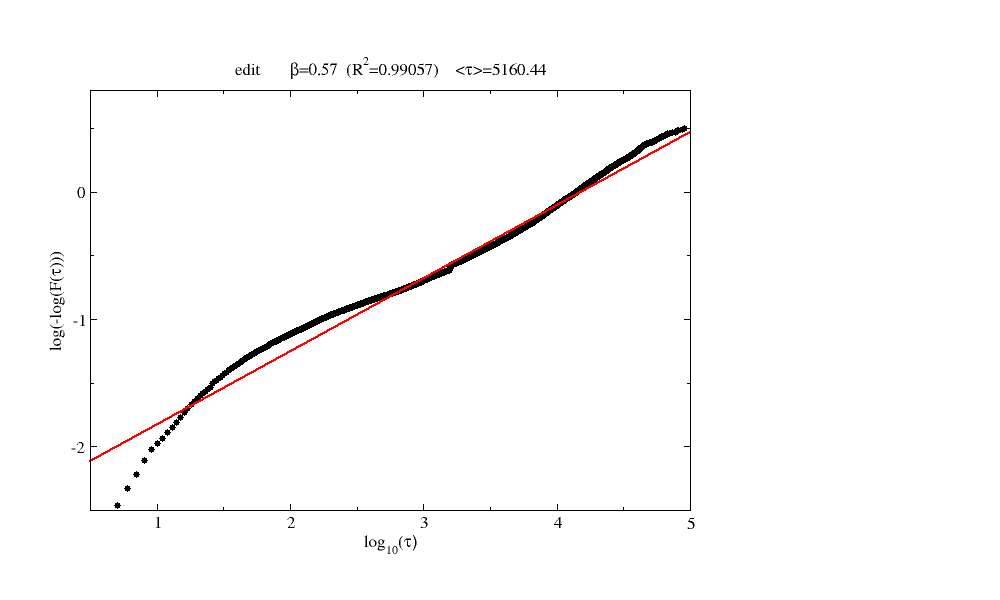

Supplement: Table S1 — Detailed information on the statistical analysis of all words that were studied (six databases). (31.88 MB TAR) [file pone.0007678.s002.tar › recurrence/comp/edit.png]

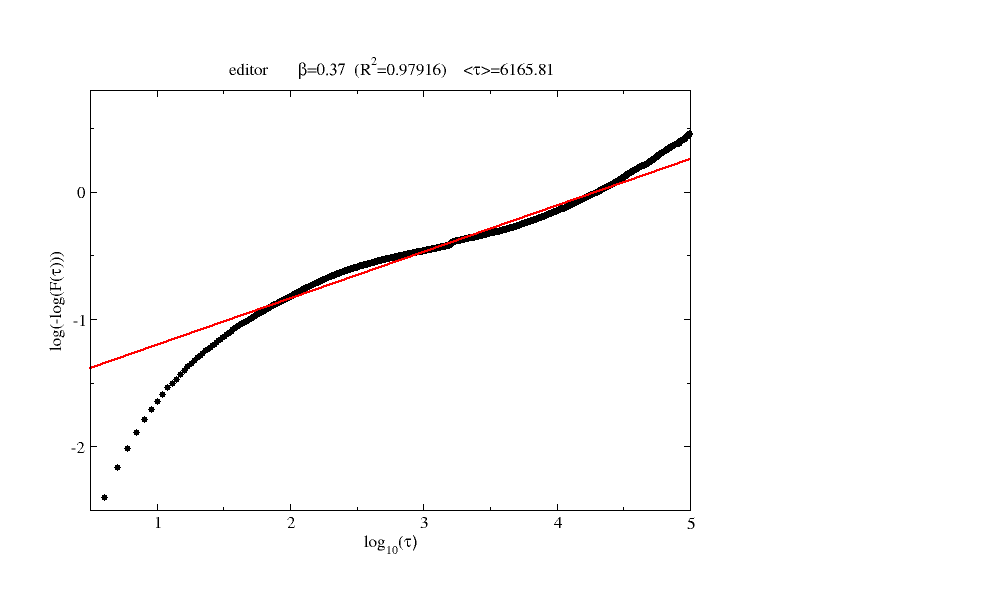

Supplement: Table S1 — Detailed information on the statistical analysis of all words that were studied (six databases). (31.88 MB TAR) [file pone.0007678.s002.tar › recurrence/comp/editor.png]

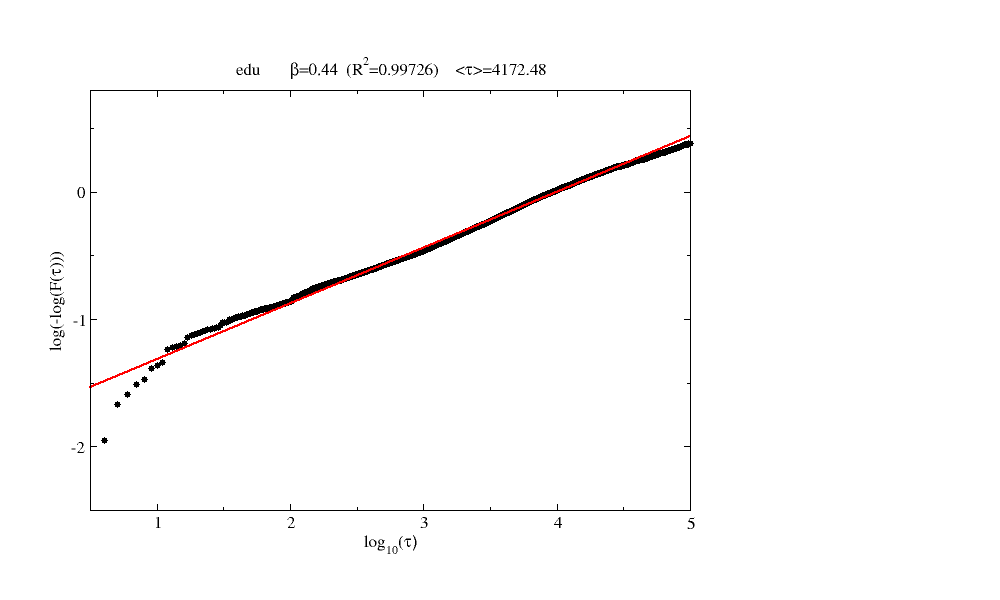

Supplement: Table S1 — Detailed information on the statistical analysis of all words that were studied (six databases). (31.88 MB TAR) [file pone.0007678.s002.tar › recurrence/comp/edu.png]

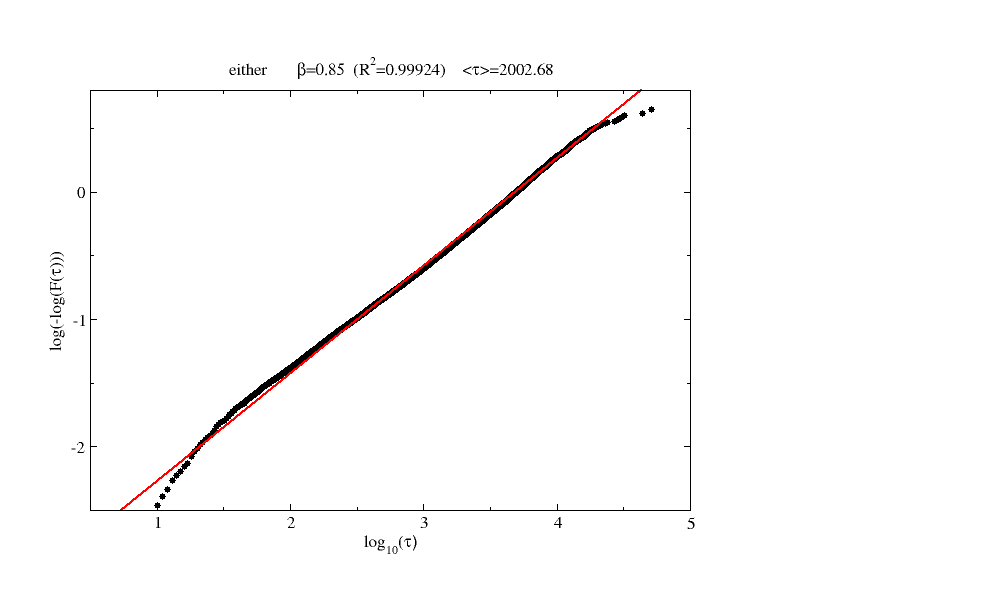

Supplement: Table S1 — Detailed information on the statistical analysis of all words that were studied (six databases). (31.88 MB TAR) [file pone.0007678.s002.tar › recurrence/comp/either.png]

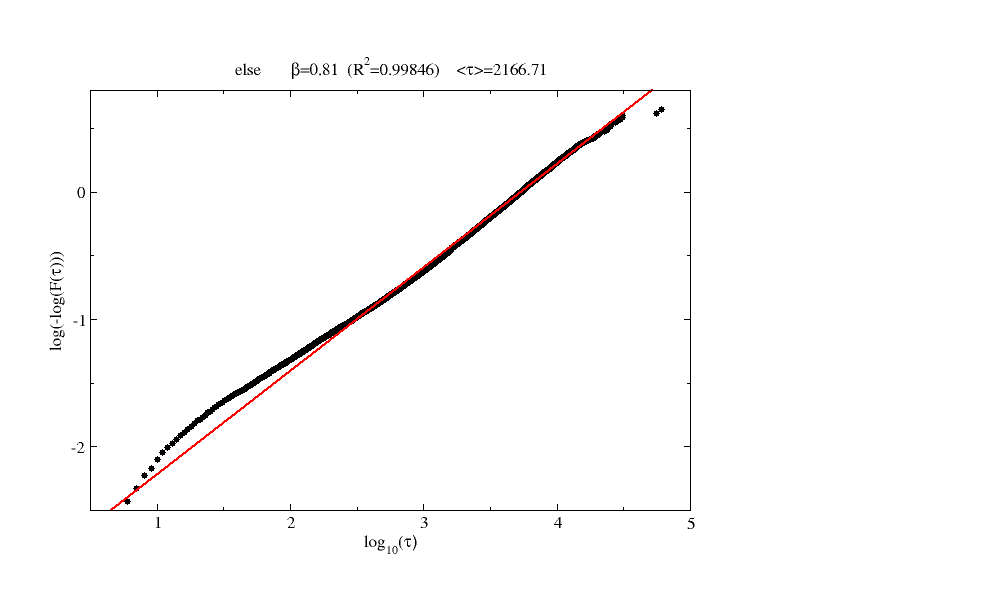

Supplement: Table S1 — Detailed information on the statistical analysis of all words that were studied (six databases). (31.88 MB TAR) [file pone.0007678.s002.tar › recurrence/comp/else.png]

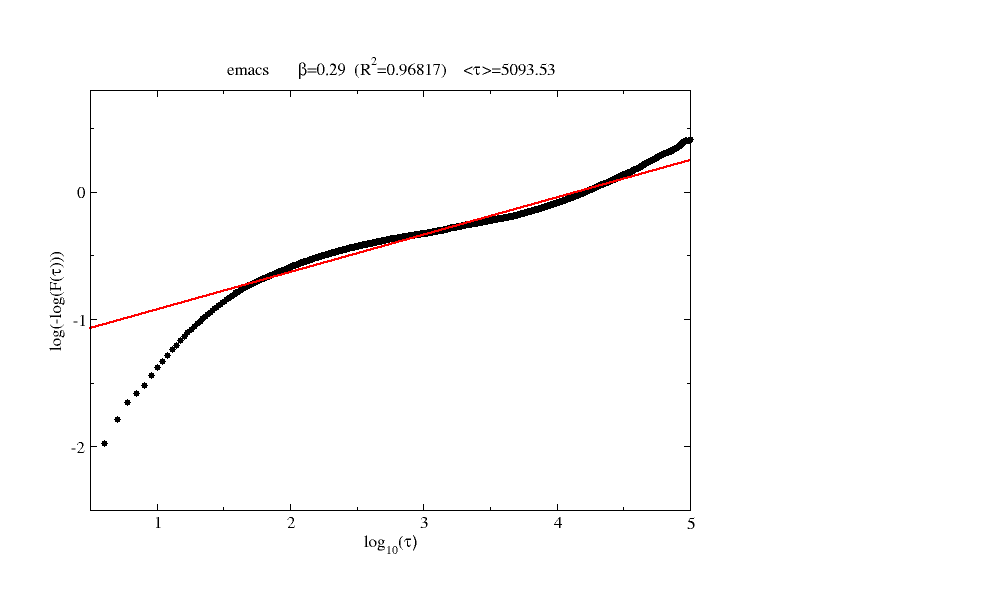

Supplement: Table S1 — Detailed information on the statistical analysis of all words that were studied (six databases). (31.88 MB TAR) [file pone.0007678.s002.tar › recurrence/comp/emacs.png]

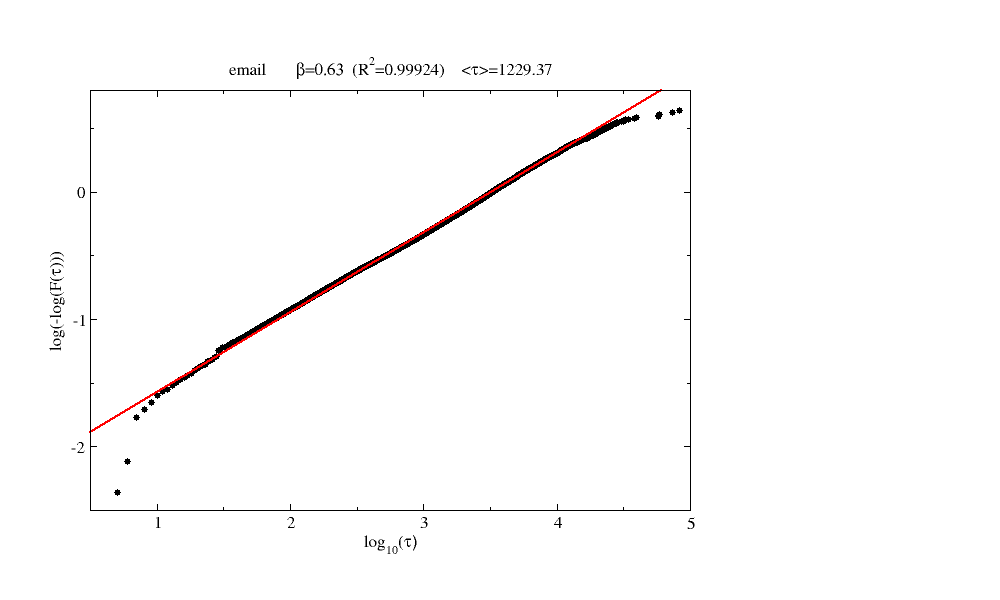

Supplement: Table S1 — Detailed information on the statistical analysis of all words that were studied (six databases). (31.88 MB TAR) [file pone.0007678.s002.tar › recurrence/comp/email.png]

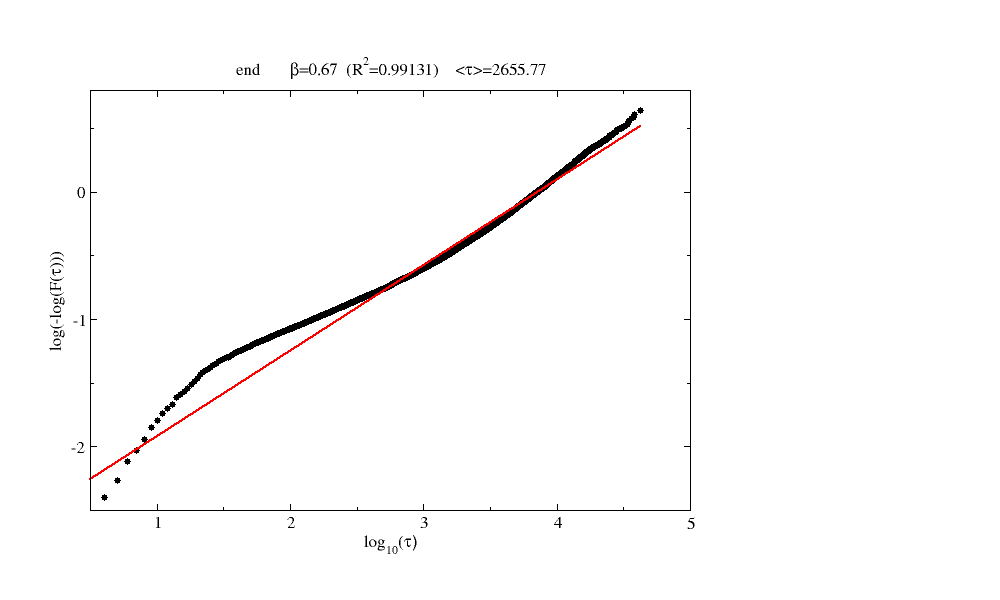

Supplement: Table S1 — Detailed information on the statistical analysis of all words that were studied (six databases). (31.88 MB TAR) [file pone.0007678.s002.tar › recurrence/comp/end.png]

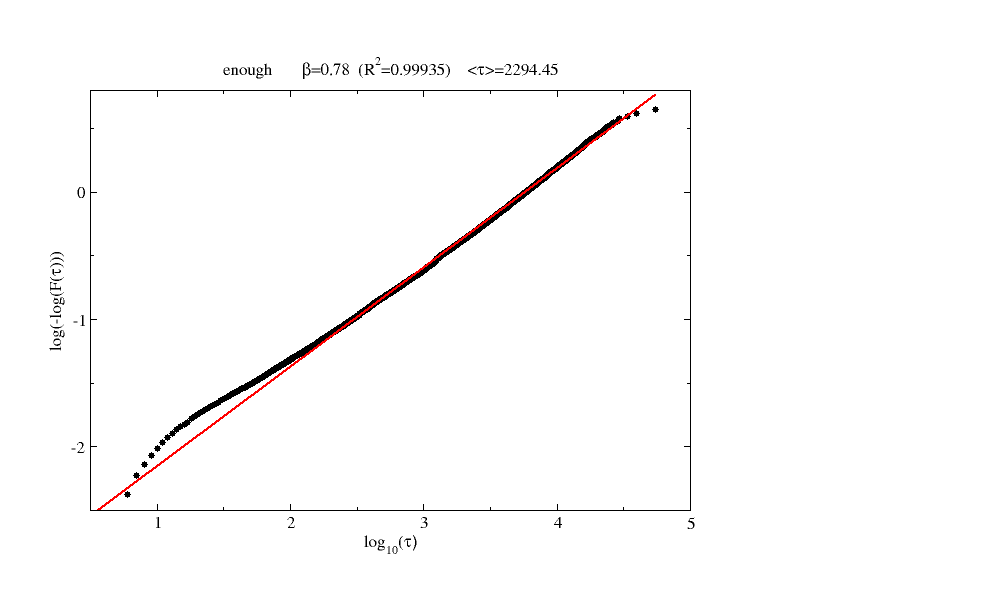

Supplement: Table S1 — Detailed information on the statistical analysis of all words that were studied (six databases). (31.88 MB TAR) [file pone.0007678.s002.tar › recurrence/comp/enough.png]

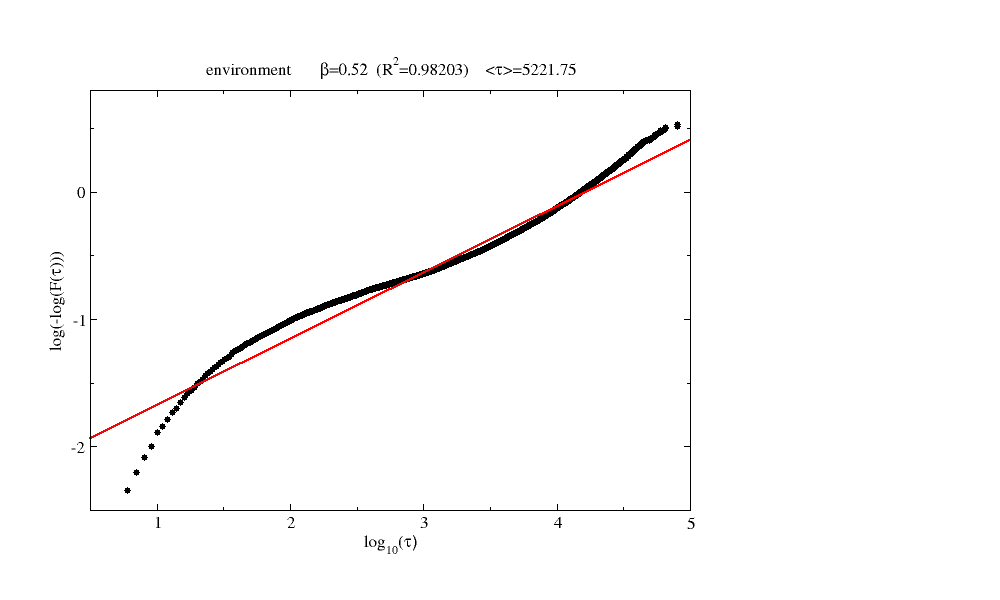

Supplement: Table S1 — Detailed information on the statistical analysis of all words that were studied (six databases). (31.88 MB TAR) [file pone.0007678.s002.tar › recurrence/comp/environment.png]

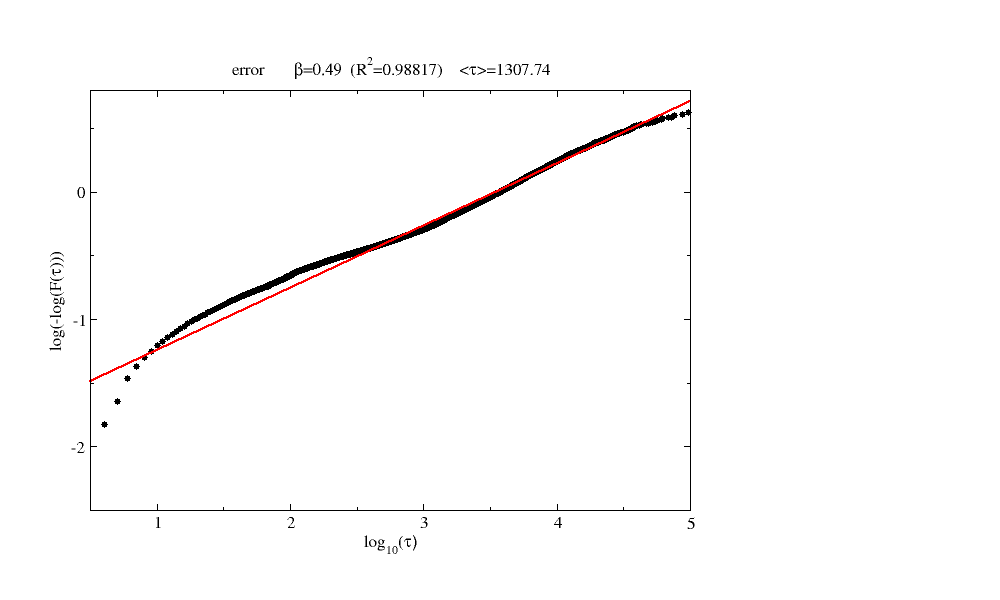

Supplement: Table S1 — Detailed information on the statistical analysis of all words that were studied (six databases). (31.88 MB TAR) [file pone.0007678.s002.tar › recurrence/comp/error.png]

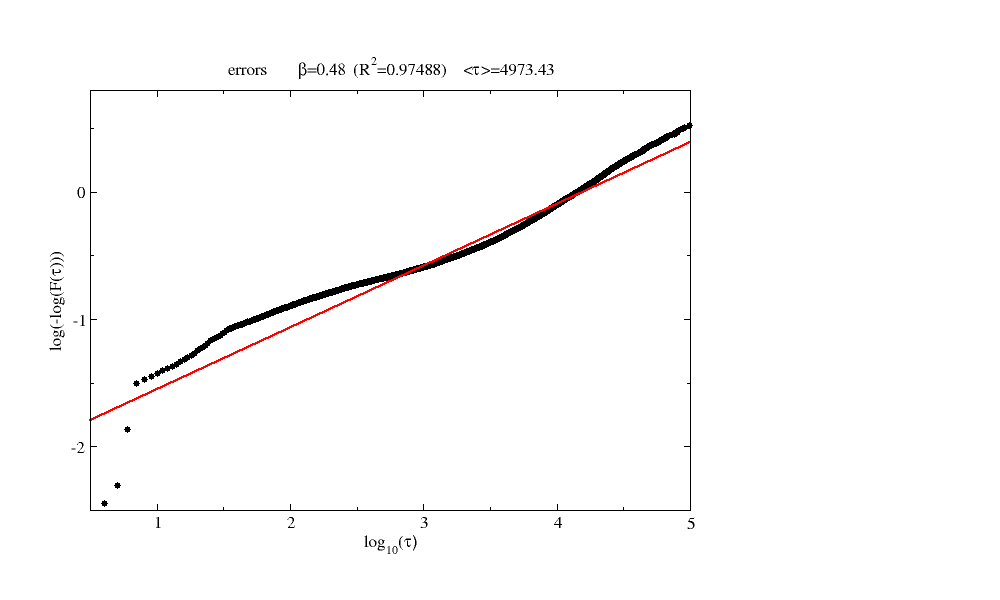

Supplement: Table S1 — Detailed information on the statistical analysis of all words that were studied (six databases). (31.88 MB TAR) [file pone.0007678.s002.tar › recurrence/comp/errors.png]

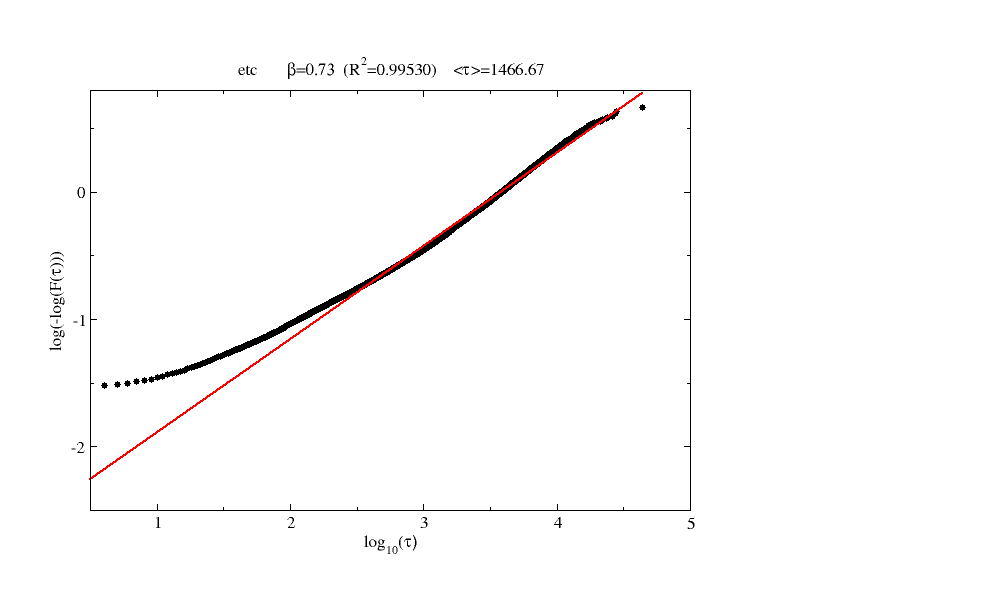

Supplement: Table S1 — Detailed information on the statistical analysis of all words that were studied (six databases). (31.88 MB TAR) [file pone.0007678.s002.tar › recurrence/comp/etc.png]

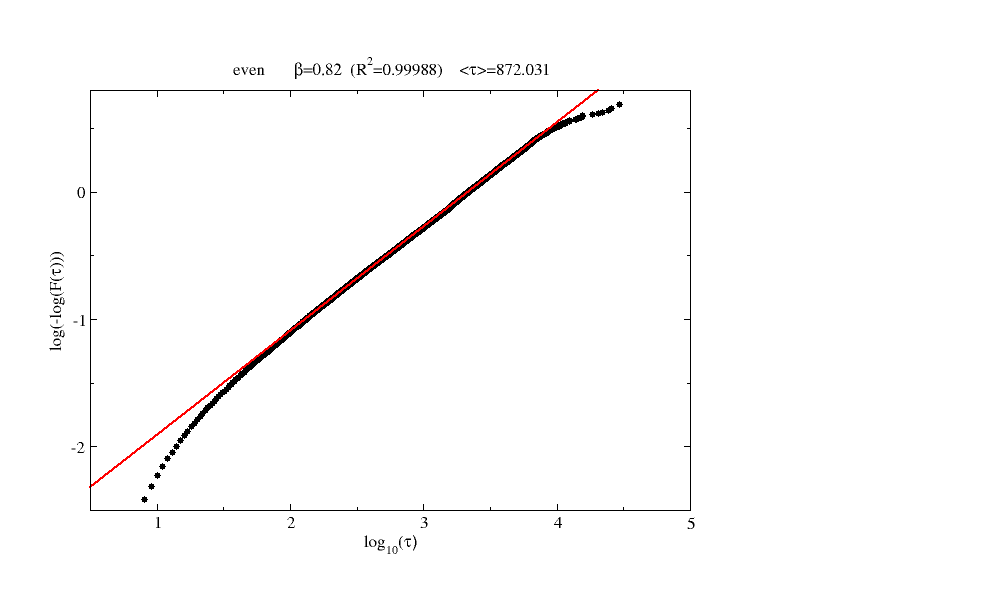

Supplement: Table S1 — Detailed information on the statistical analysis of all words that were studied (six databases). (31.88 MB TAR) [file pone.0007678.s002.tar › recurrence/comp/even.png]

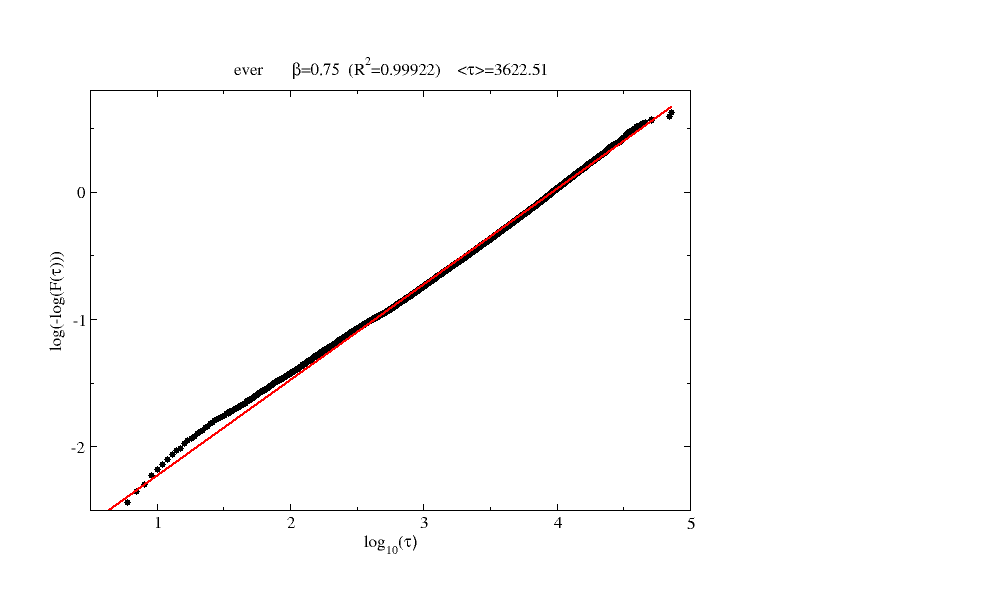

Supplement: Table S1 — Detailed information on the statistical analysis of all words that were studied (six databases). (31.88 MB TAR) [file pone.0007678.s002.tar › recurrence/comp/ever.png]

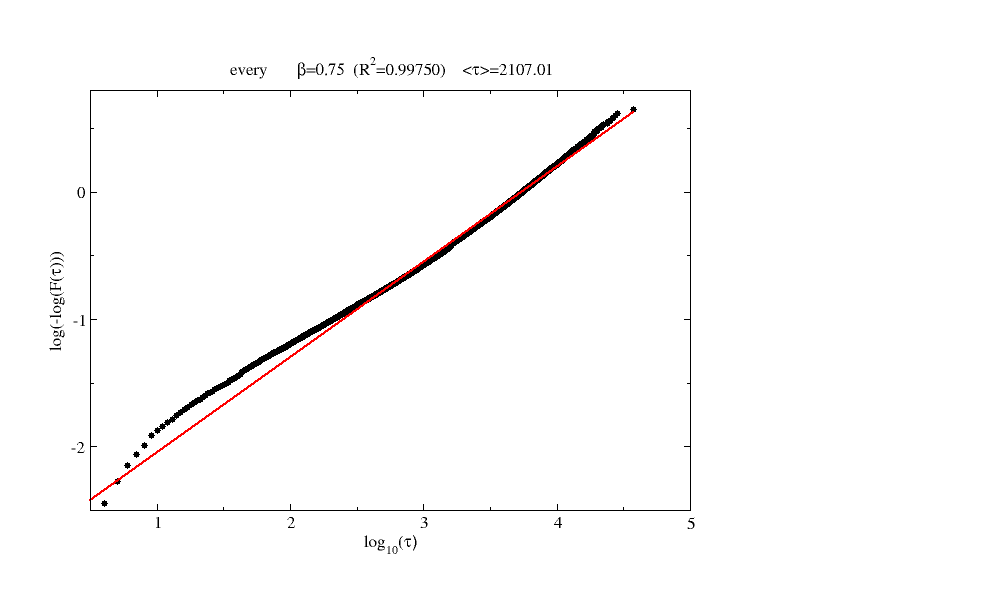

Supplement: Table S1 — Detailed information on the statistical analysis of all words that were studied (six databases). (31.88 MB TAR) [file pone.0007678.s002.tar › recurrence/comp/every.png]

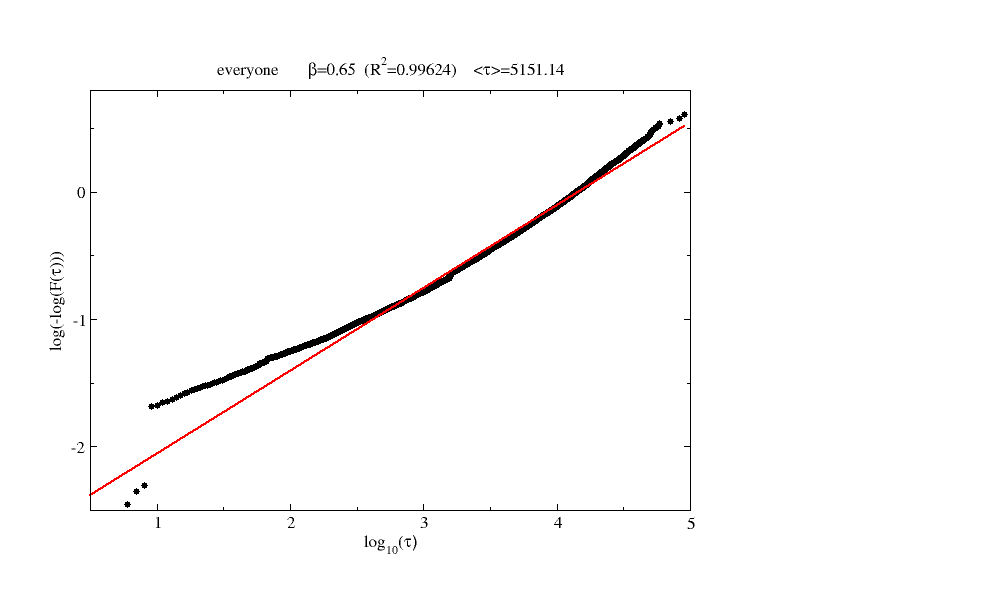

Supplement: Table S1 — Detailed information on the statistical analysis of all words that were studied (six databases). (31.88 MB TAR) [file pone.0007678.s002.tar › recurrence/comp/everyone.png]

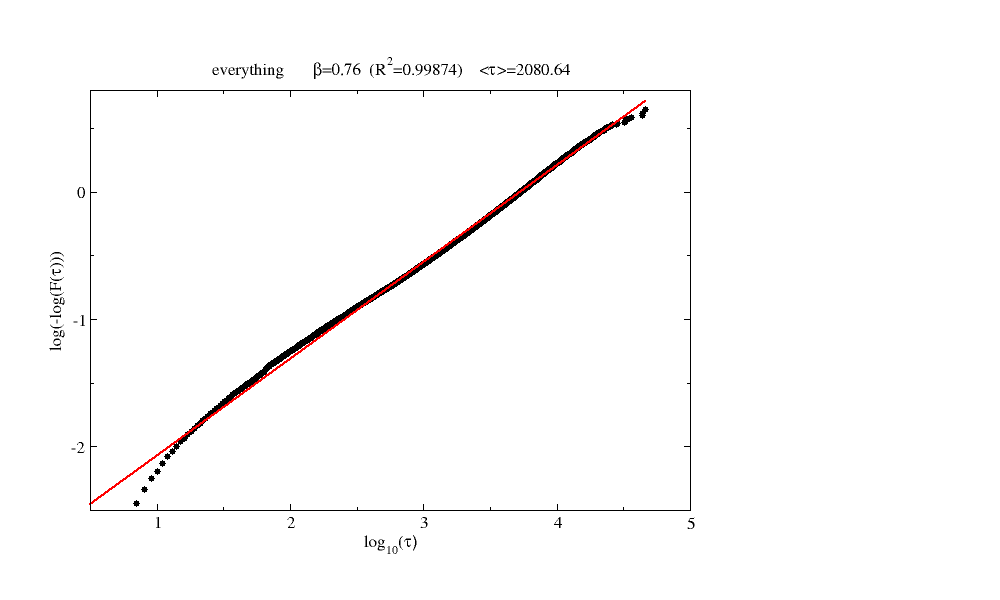

Supplement: Table S1 — Detailed information on the statistical analysis of all words that were studied (six databases). (31.88 MB TAR) [file pone.0007678.s002.tar › recurrence/comp/everything.png]

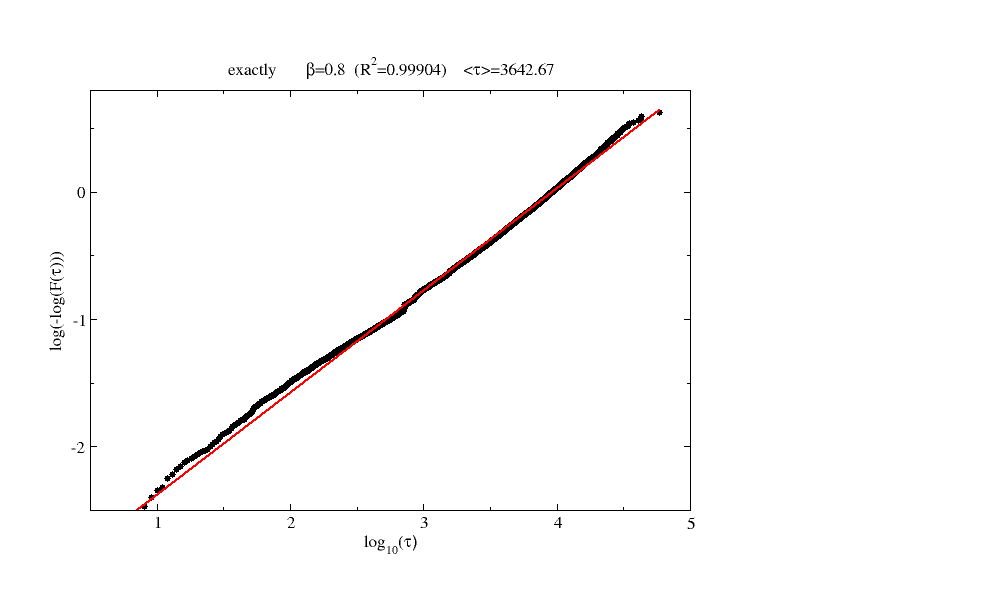

Supplement: Table S1 — Detailed information on the statistical analysis of all words that were studied (six databases). (31.88 MB TAR) [file pone.0007678.s002.tar › recurrence/comp/exactly.png]

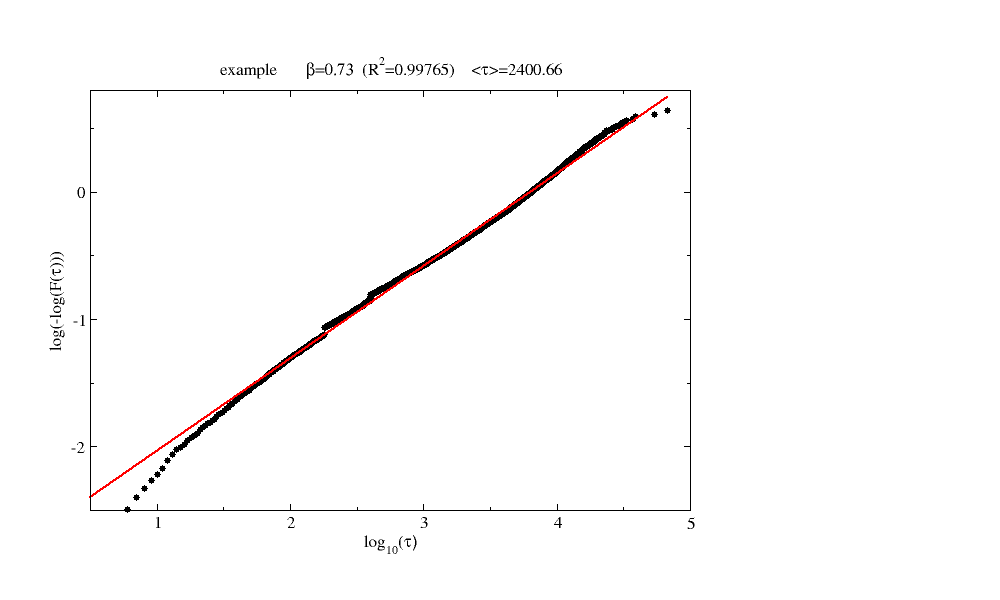

Supplement: Table S1 — Detailed information on the statistical analysis of all words that were studied (six databases). (31.88 MB TAR) [file pone.0007678.s002.tar › recurrence/comp/example.png]

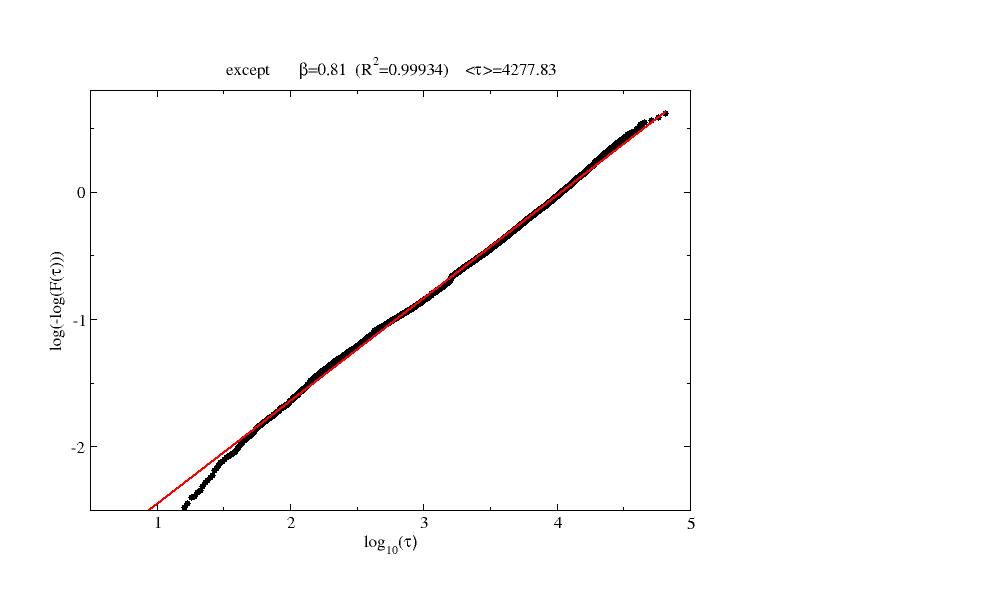

Supplement: Table S1 — Detailed information on the statistical analysis of all words that were studied (six databases). (31.88 MB TAR) [file pone.0007678.s002.tar › recurrence/comp/except.png]
