# Supplementary figures and images for: Beyond Word Frequency: Bursts, Lulls, and Scaling in the Temporal Distributions of Words (part 1 of 2)
Source: PLoS One. 2009 Nov 11;4(11):e7678. doi: 10.1371/journal.pone.0007678 (PMC2770836; doi:10.1371/journal.pone.0007678)

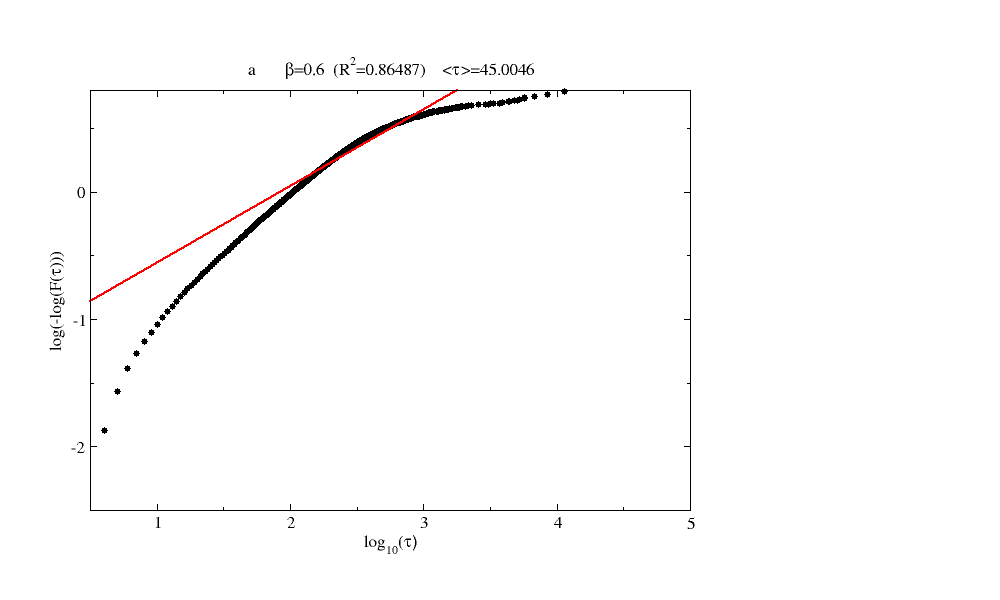

Supplement: Table S1 — Detailed information on the statistical analysis of all words that were studied (six databases). (31.88 MB TAR) [file pone.0007678.s002.tar › recurrence/comp/a.png]

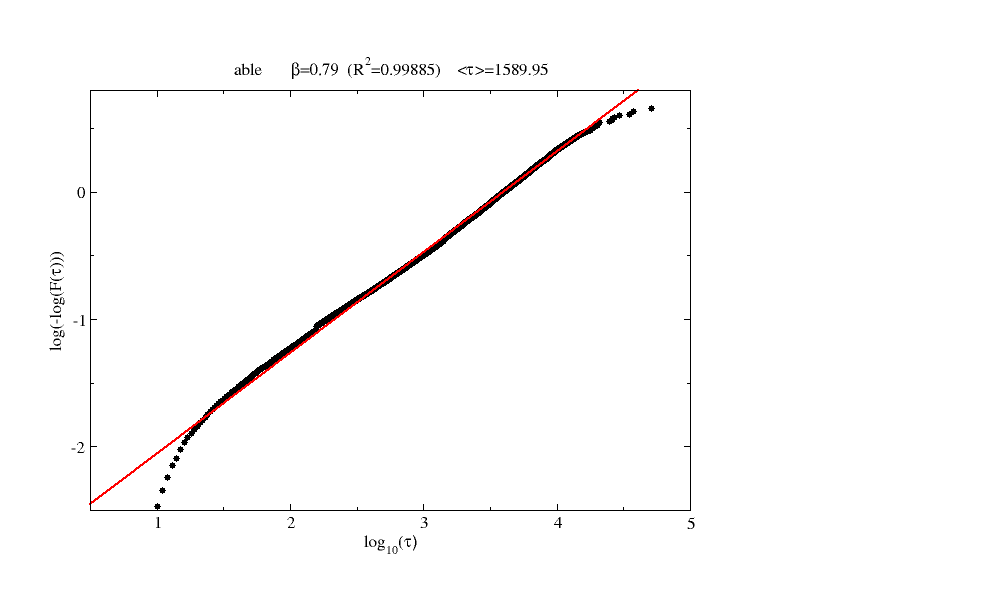

Supplement: Table S1 — Detailed information on the statistical analysis of all words that were studied (six databases). (31.88 MB TAR) [file pone.0007678.s002.tar › recurrence/comp/able.png]

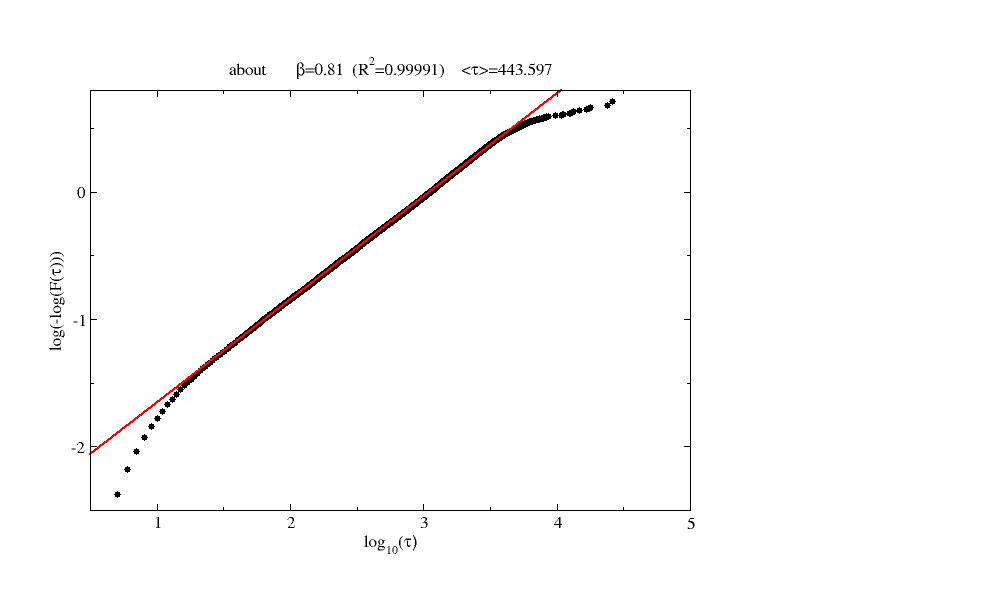

Supplement: Table S1 — Detailed information on the statistical analysis of all words that were studied (six databases). (31.88 MB TAR) [file pone.0007678.s002.tar › recurrence/comp/about.png]

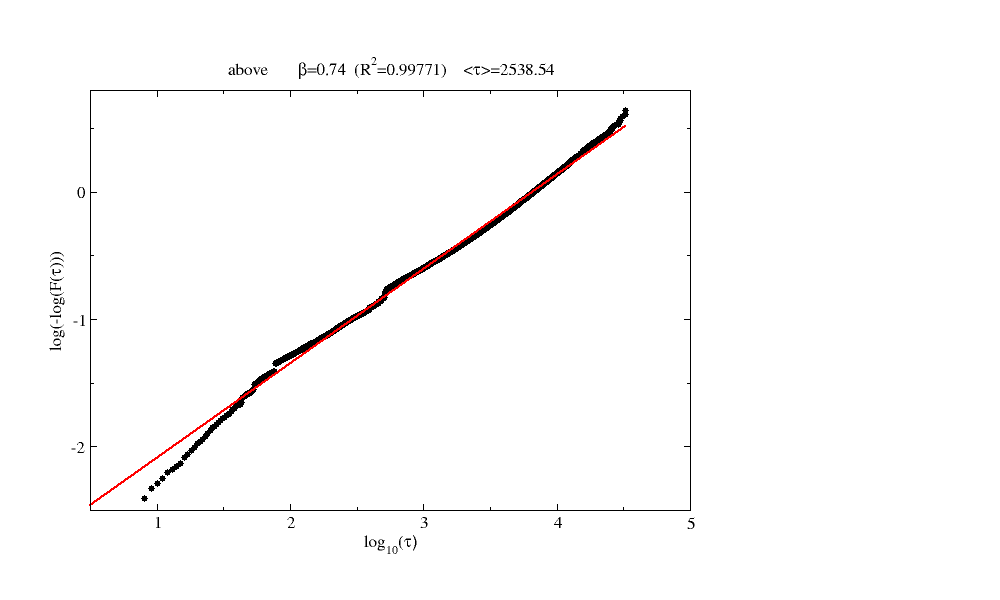

Supplement: Table S1 — Detailed information on the statistical analysis of all words that were studied (six databases). (31.88 MB TAR) [file pone.0007678.s002.tar › recurrence/comp/above.png]

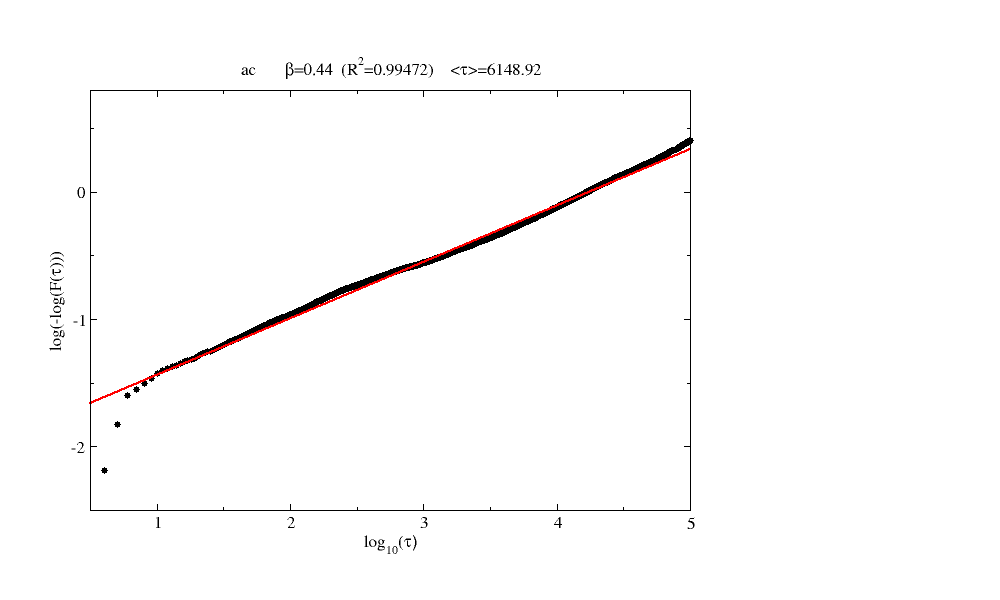

Supplement: Table S1 — Detailed information on the statistical analysis of all words that were studied (six databases). (31.88 MB TAR) [file pone.0007678.s002.tar › recurrence/comp/ac.png]

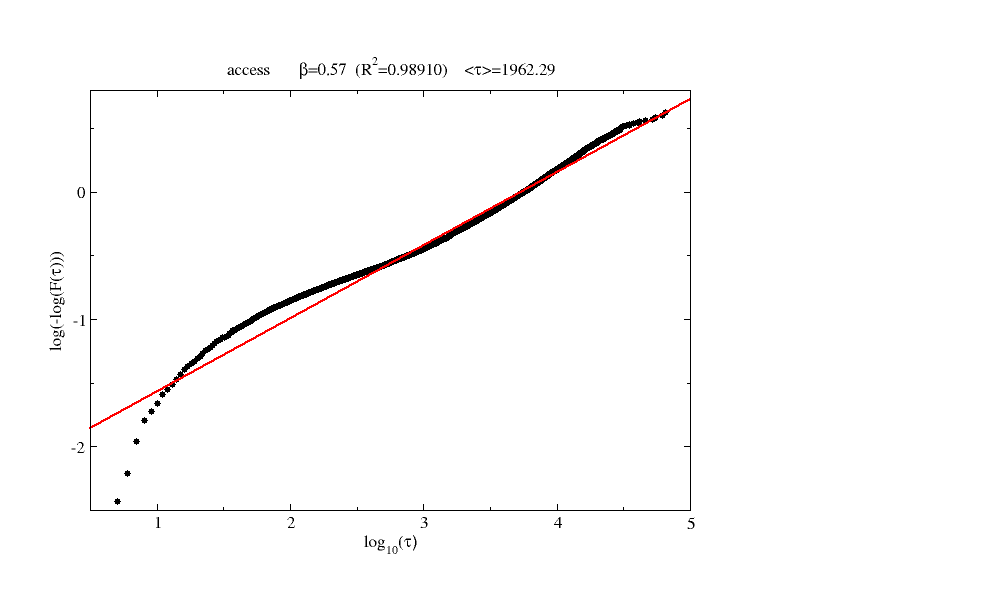

Supplement: Table S1 — Detailed information on the statistical analysis of all words that were studied (six databases). (31.88 MB TAR) [file pone.0007678.s002.tar › recurrence/comp/access.png]

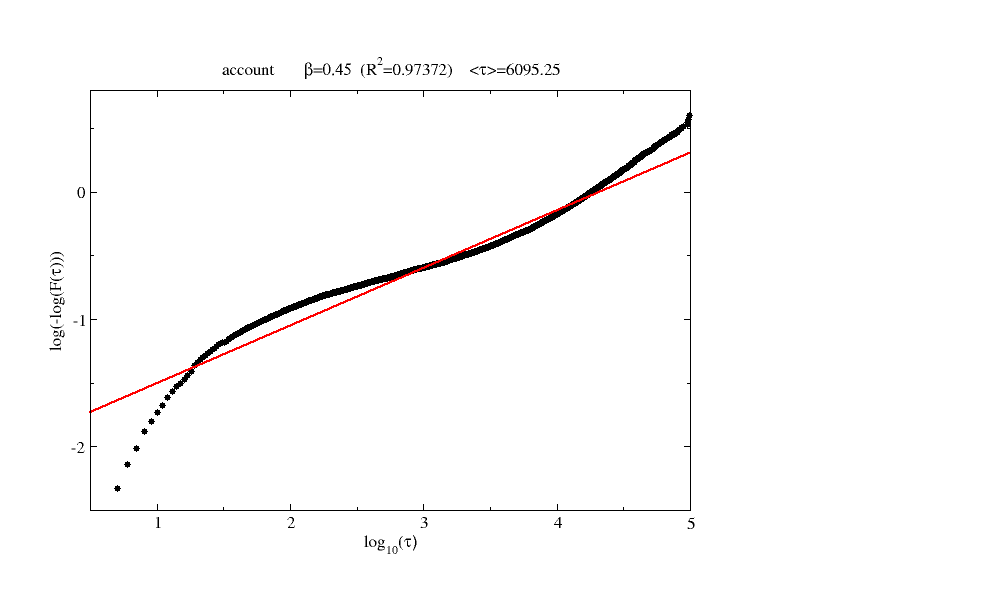

Supplement: Table S1 — Detailed information on the statistical analysis of all words that were studied (six databases). (31.88 MB TAR) [file pone.0007678.s002.tar › recurrence/comp/account.png]

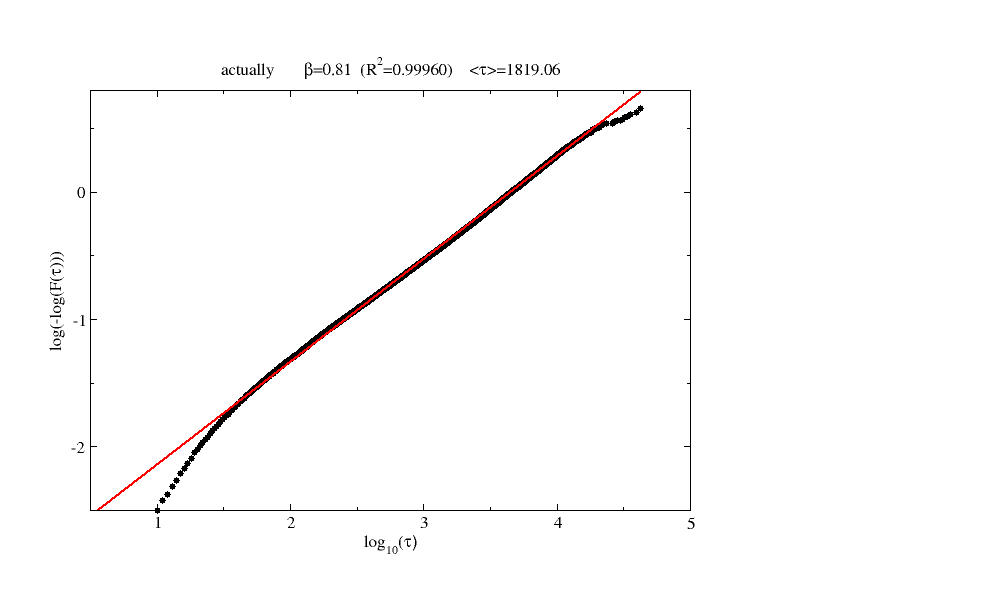

Supplement: Table S1 — Detailed information on the statistical analysis of all words that were studied (six databases). (31.88 MB TAR) [file pone.0007678.s002.tar › recurrence/comp/actually.png]

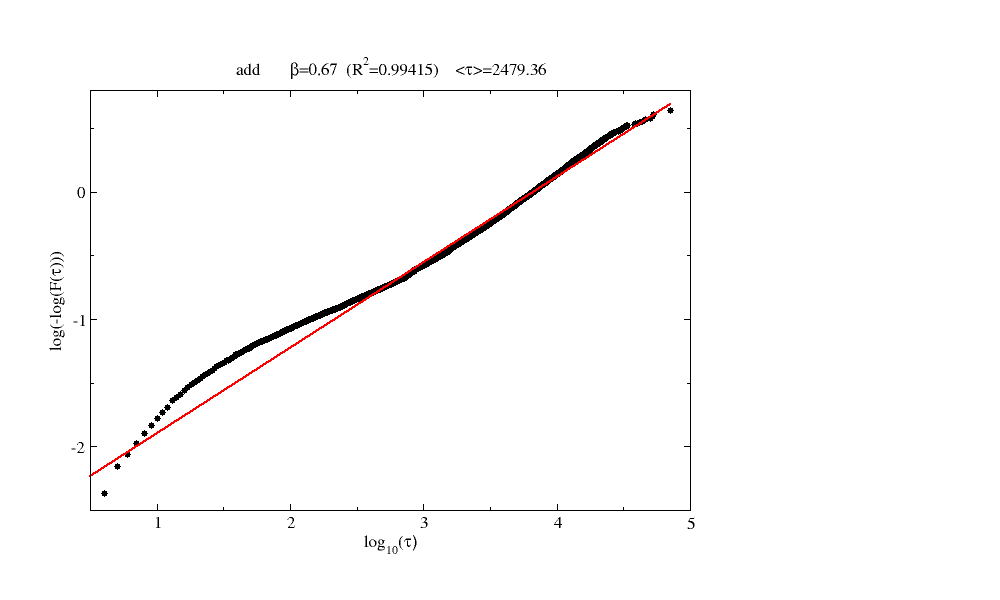

Supplement: Table S1 — Detailed information on the statistical analysis of all words that were studied (six databases). (31.88 MB TAR) [file pone.0007678.s002.tar › recurrence/comp/add.png]

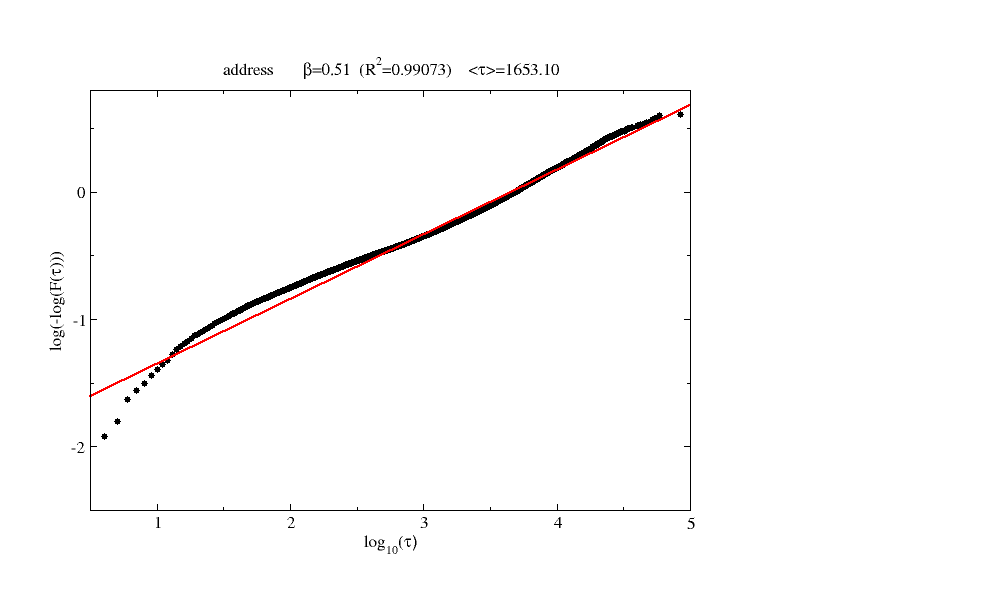

Supplement: Table S1 — Detailed information on the statistical analysis of all words that were studied (six databases). (31.88 MB TAR) [file pone.0007678.s002.tar › recurrence/comp/address.png]

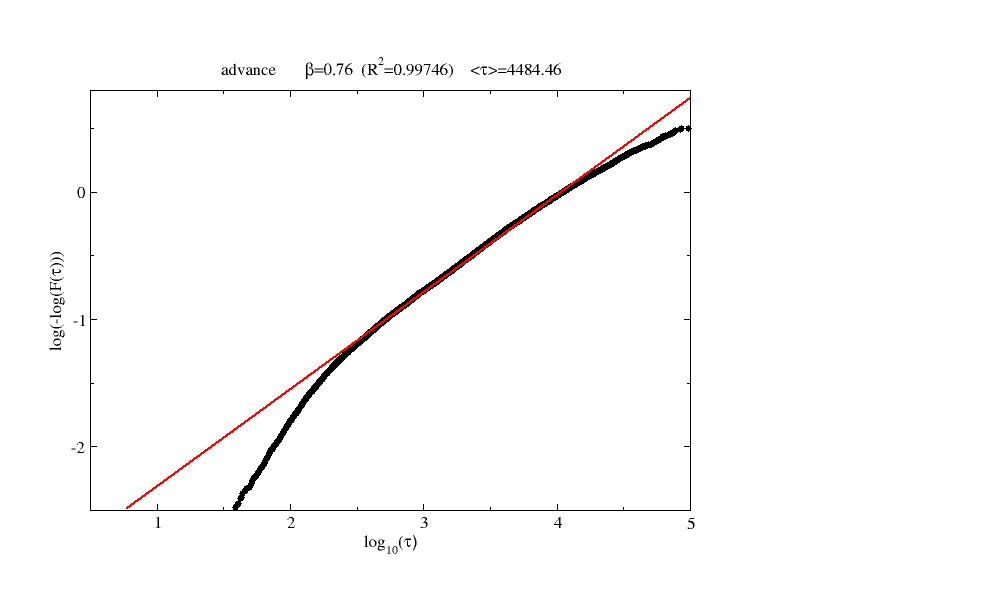

Supplement: Table S1 — Detailed information on the statistical analysis of all words that were studied (six databases). (31.88 MB TAR) [file pone.0007678.s002.tar › recurrence/comp/advance.png]

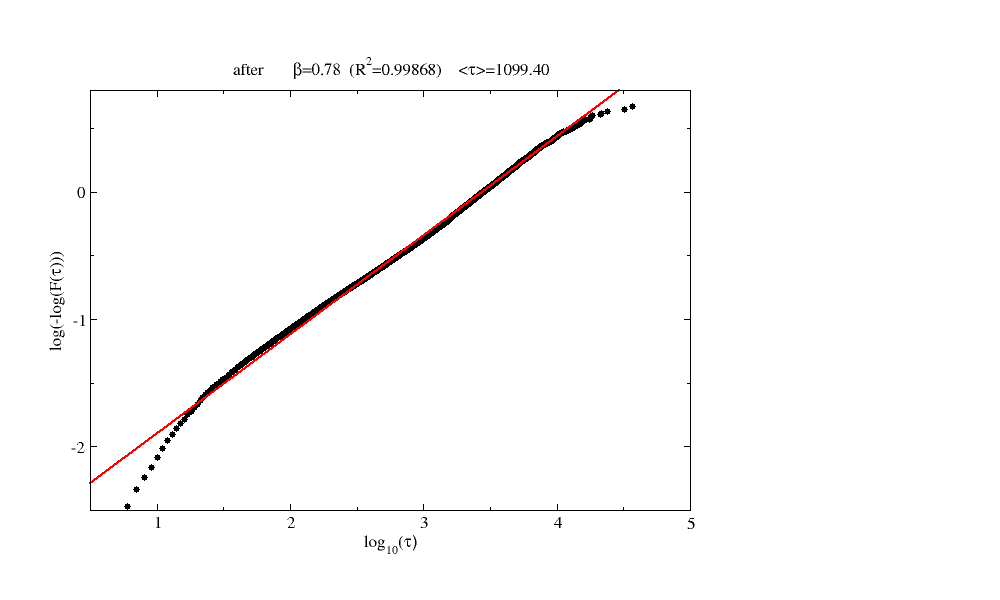

Supplement: Table S1 — Detailed information on the statistical analysis of all words that were studied (six databases). (31.88 MB TAR) [file pone.0007678.s002.tar › recurrence/comp/after.png]

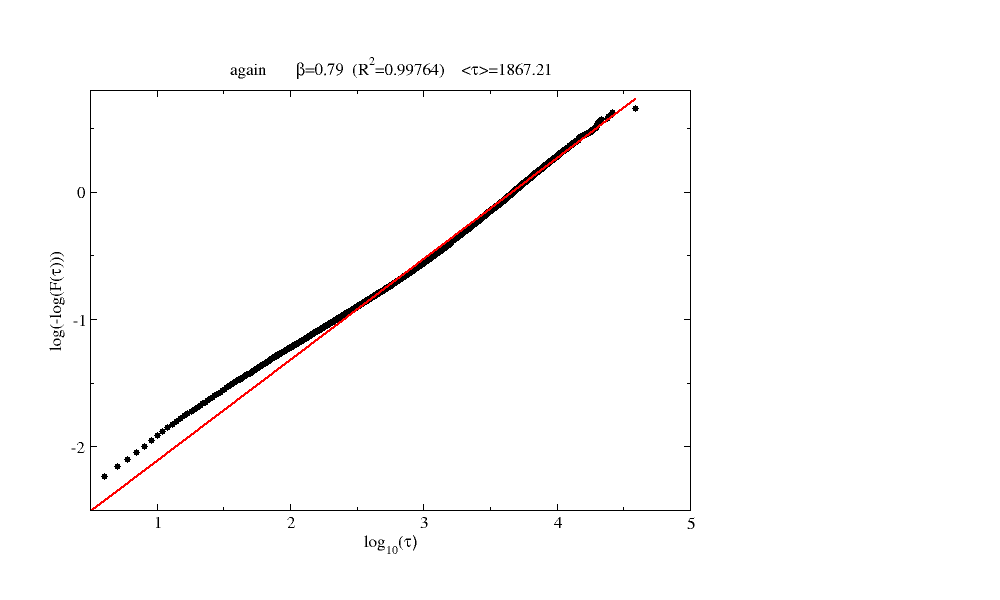

Supplement: Table S1 — Detailed information on the statistical analysis of all words that were studied (six databases). (31.88 MB TAR) [file pone.0007678.s002.tar › recurrence/comp/again.png]

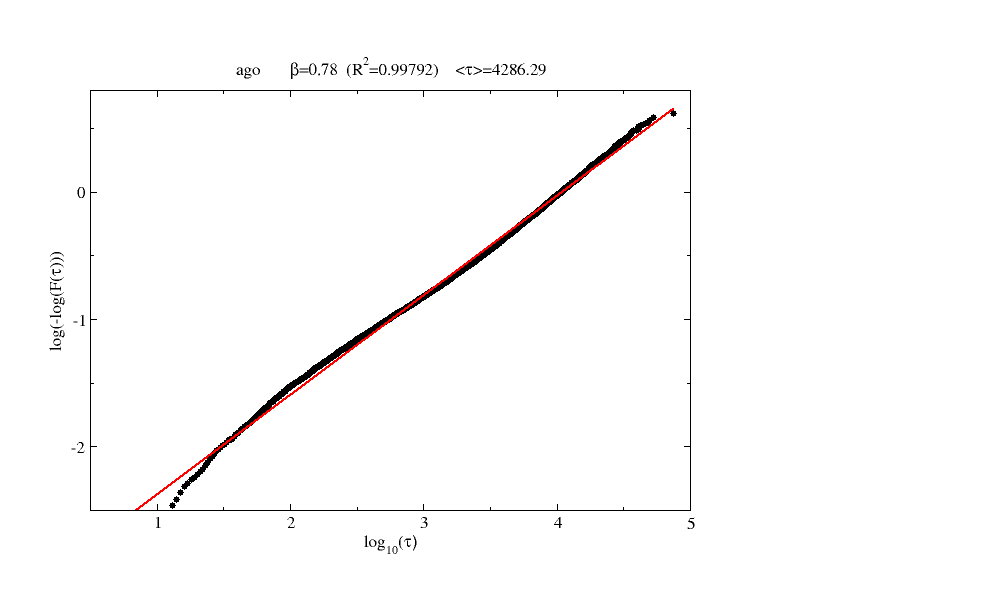

Supplement: Table S1 — Detailed information on the statistical analysis of all words that were studied (six databases). (31.88 MB TAR) [file pone.0007678.s002.tar › recurrence/comp/ago.png]

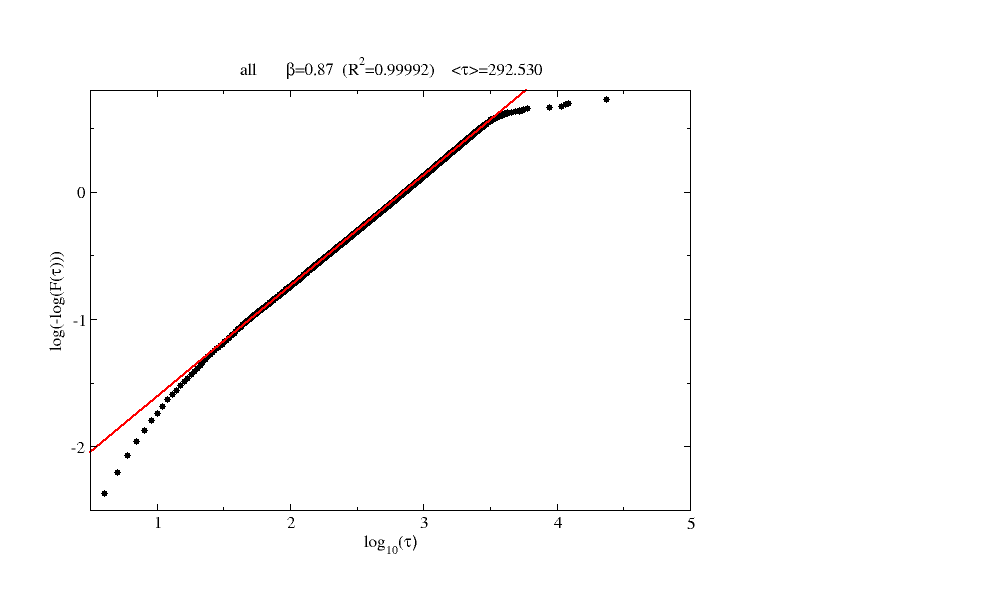

Supplement: Table S1 — Detailed information on the statistical analysis of all words that were studied (six databases). (31.88 MB TAR) [file pone.0007678.s002.tar › recurrence/comp/all.png]

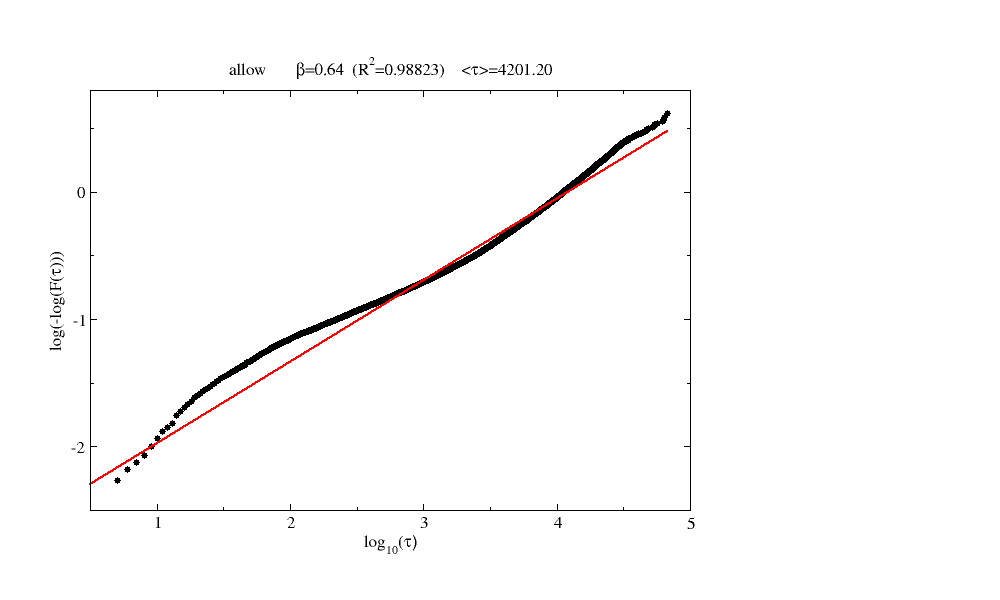

Supplement: Table S1 — Detailed information on the statistical analysis of all words that were studied (six databases). (31.88 MB TAR) [file pone.0007678.s002.tar › recurrence/comp/allow.png]

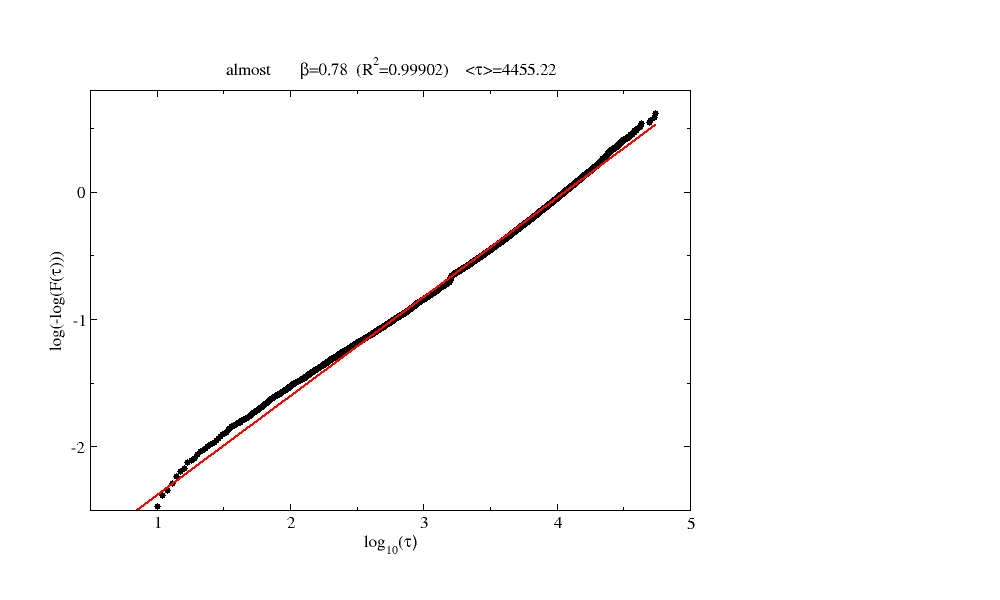

Supplement: Table S1 — Detailed information on the statistical analysis of all words that were studied (six databases). (31.88 MB TAR) [file pone.0007678.s002.tar › recurrence/comp/almost.png]

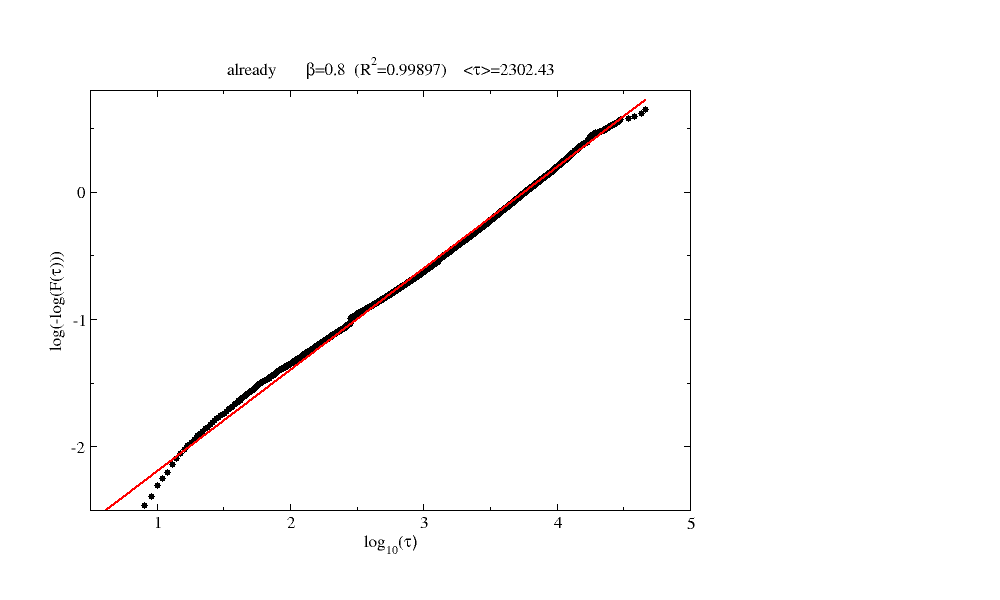

Supplement: Table S1 — Detailed information on the statistical analysis of all words that were studied (six databases). (31.88 MB TAR) [file pone.0007678.s002.tar › recurrence/comp/already.png]

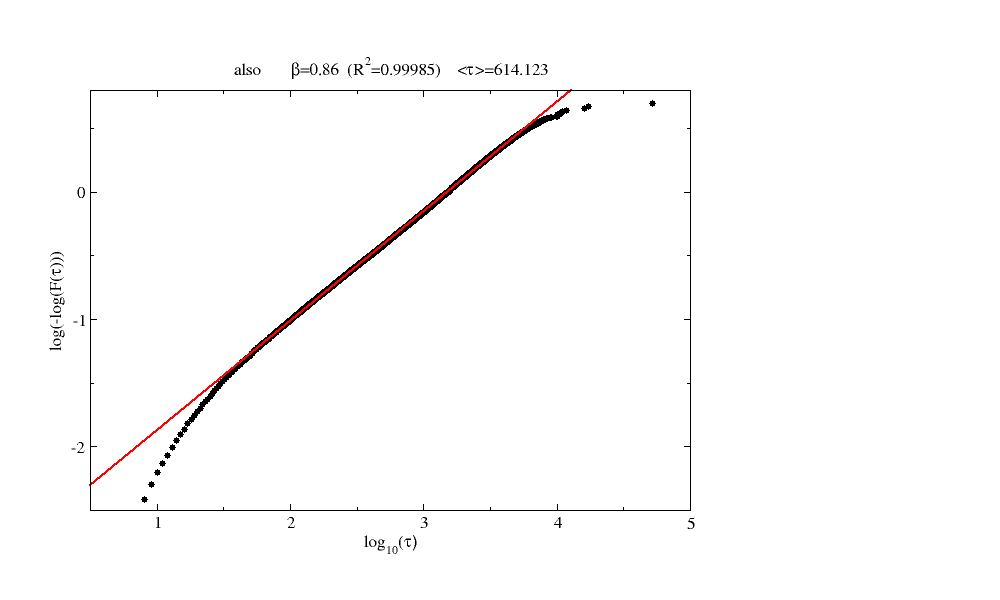

Supplement: Table S1 — Detailed information on the statistical analysis of all words that were studied (six databases). (31.88 MB TAR) [file pone.0007678.s002.tar › recurrence/comp/also.png]

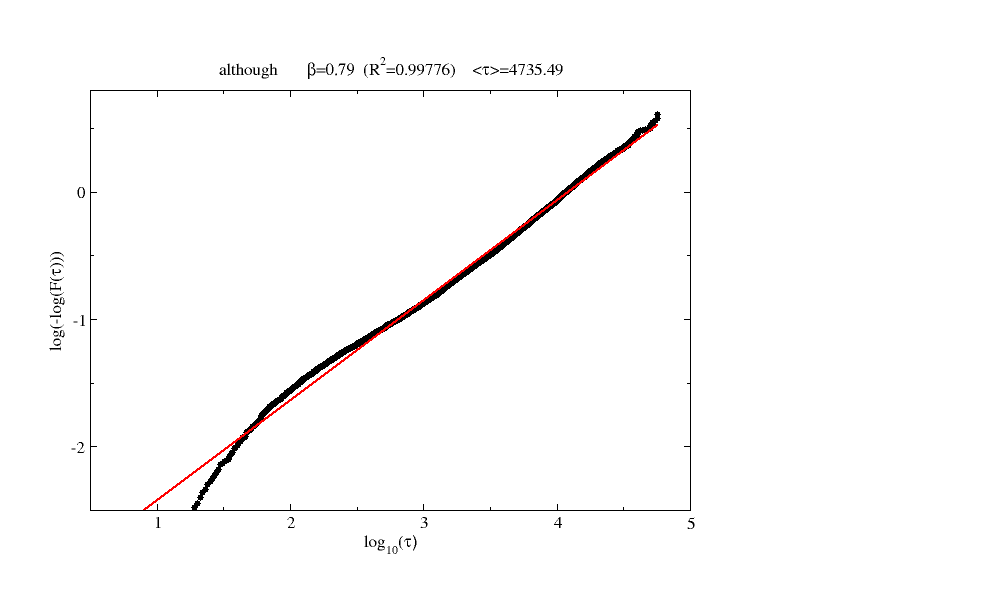

Supplement: Table S1 — Detailed information on the statistical analysis of all words that were studied (six databases). (31.88 MB TAR) [file pone.0007678.s002.tar › recurrence/comp/although.png]

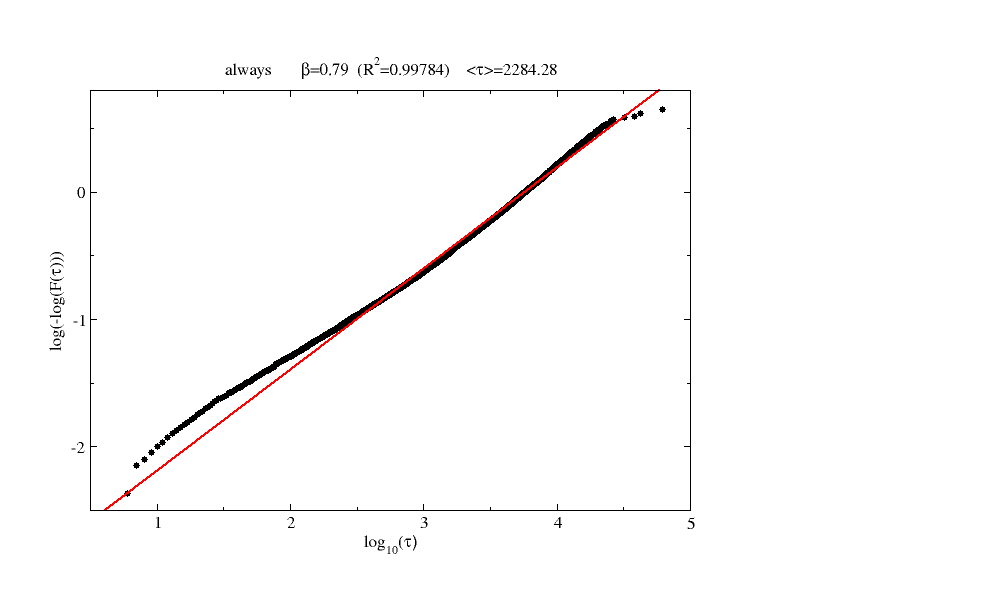

Supplement: Table S1 — Detailed information on the statistical analysis of all words that were studied (six databases). (31.88 MB TAR) [file pone.0007678.s002.tar › recurrence/comp/always.png]

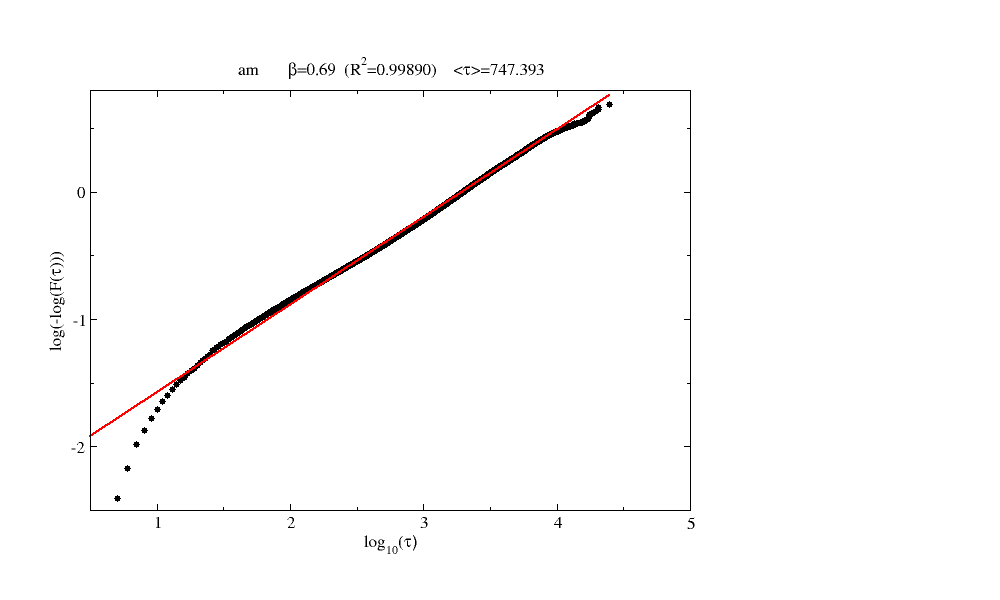

Supplement: Table S1 — Detailed information on the statistical analysis of all words that were studied (six databases). (31.88 MB TAR) [file pone.0007678.s002.tar › recurrence/comp/am.png]

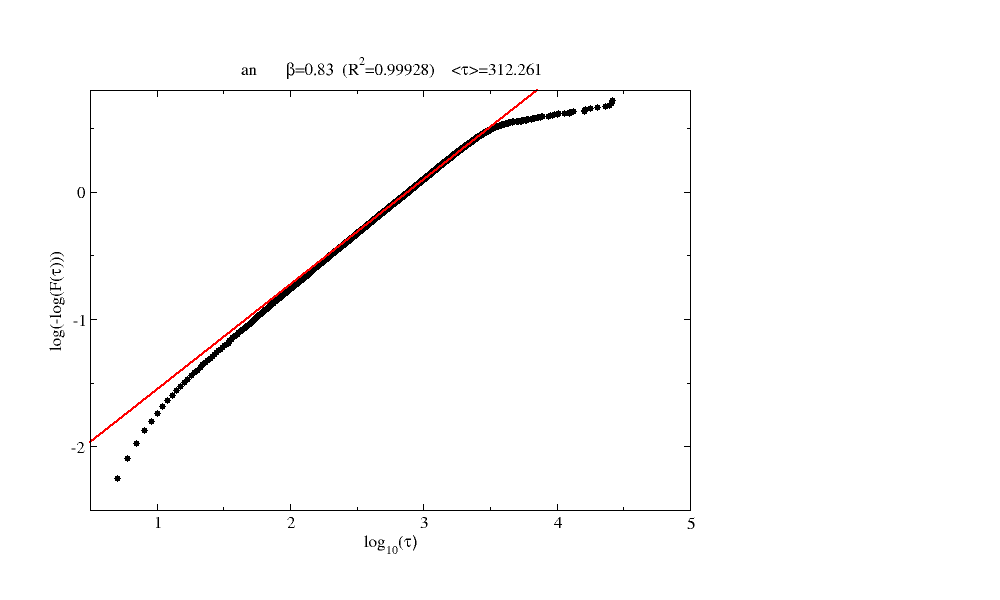

Supplement: Table S1 — Detailed information on the statistical analysis of all words that were studied (six databases). (31.88 MB TAR) [file pone.0007678.s002.tar › recurrence/comp/an.png]

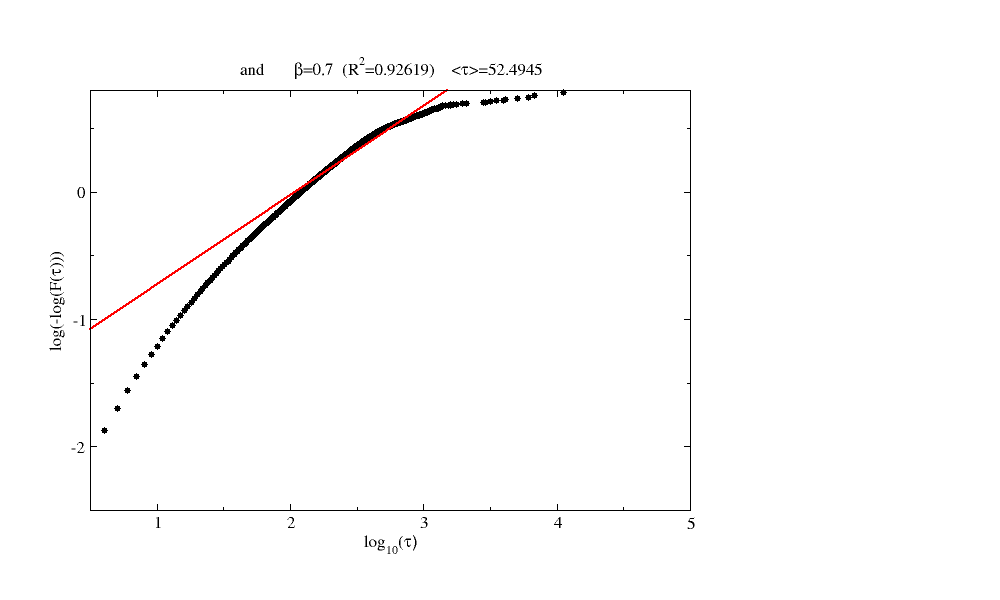

Supplement: Table S1 — Detailed information on the statistical analysis of all words that were studied (six databases). (31.88 MB TAR) [file pone.0007678.s002.tar › recurrence/comp/and.png]

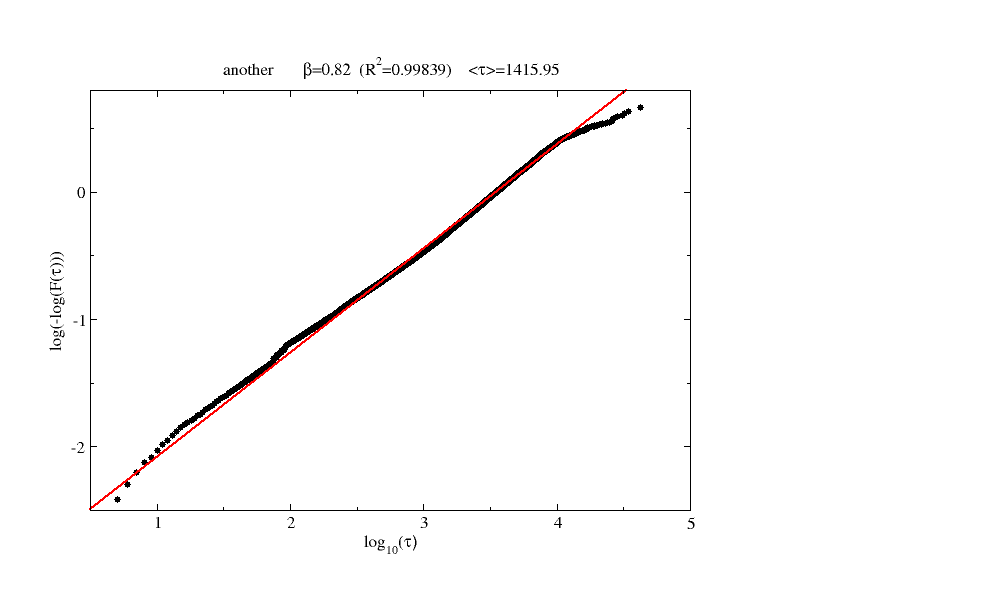

Supplement: Table S1 — Detailed information on the statistical analysis of all words that were studied (six databases). (31.88 MB TAR) [file pone.0007678.s002.tar › recurrence/comp/another.png]

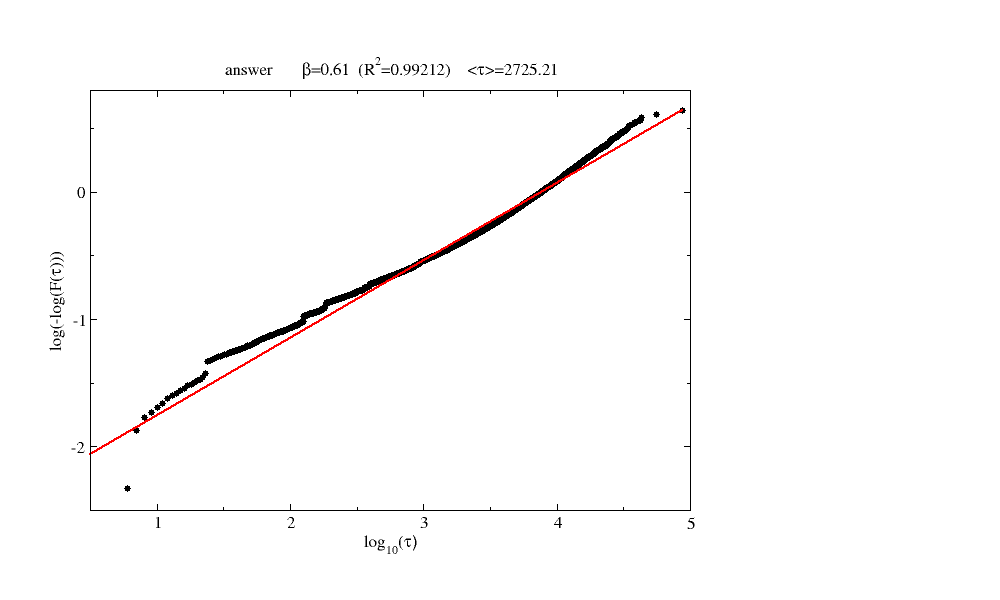

Supplement: Table S1 — Detailed information on the statistical analysis of all words that were studied (six databases). (31.88 MB TAR) [file pone.0007678.s002.tar › recurrence/comp/answer.png]

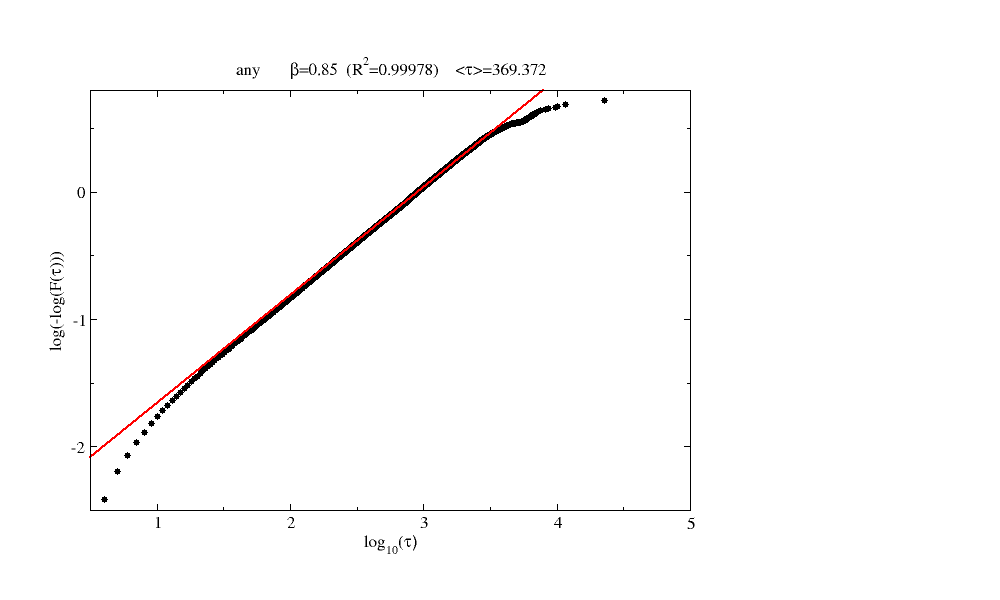

Supplement: Table S1 — Detailed information on the statistical analysis of all words that were studied (six databases). (31.88 MB TAR) [file pone.0007678.s002.tar › recurrence/comp/any.png]

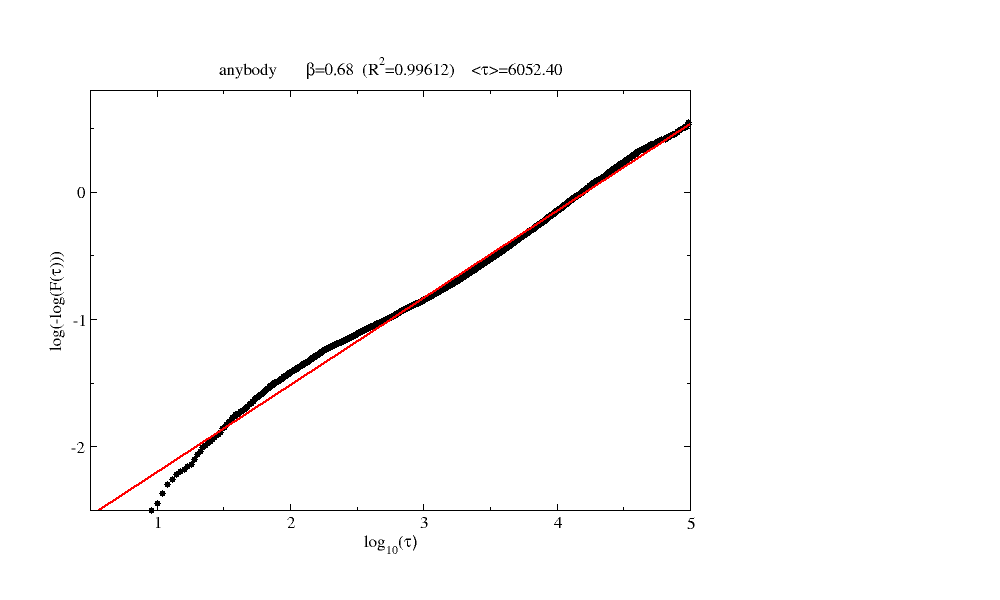

Supplement: Table S1 — Detailed information on the statistical analysis of all words that were studied (six databases). (31.88 MB TAR) [file pone.0007678.s002.tar › recurrence/comp/anybody.png]

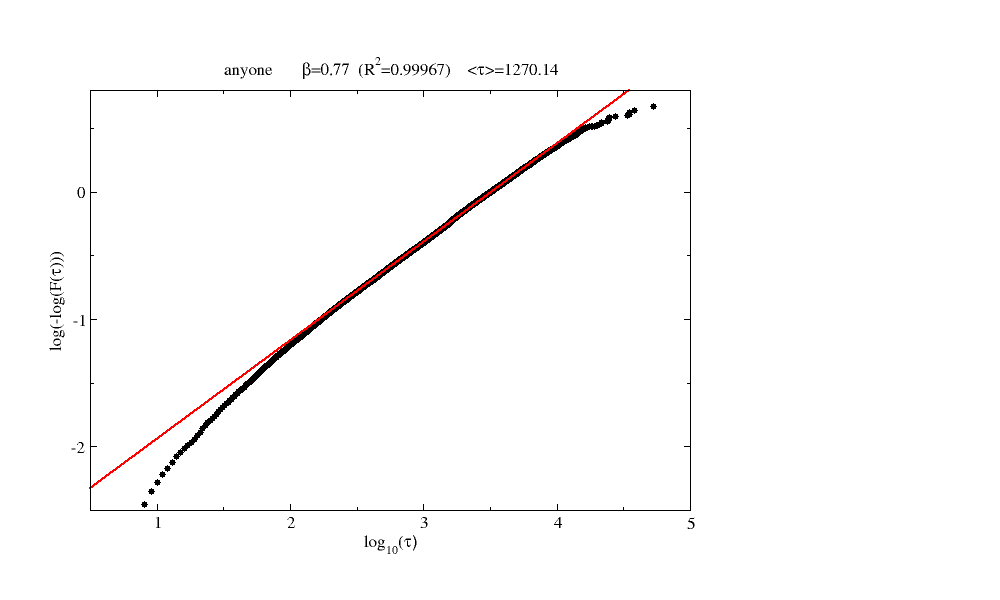

Supplement: Table S1 — Detailed information on the statistical analysis of all words that were studied (six databases). (31.88 MB TAR) [file pone.0007678.s002.tar › recurrence/comp/anyone.png]

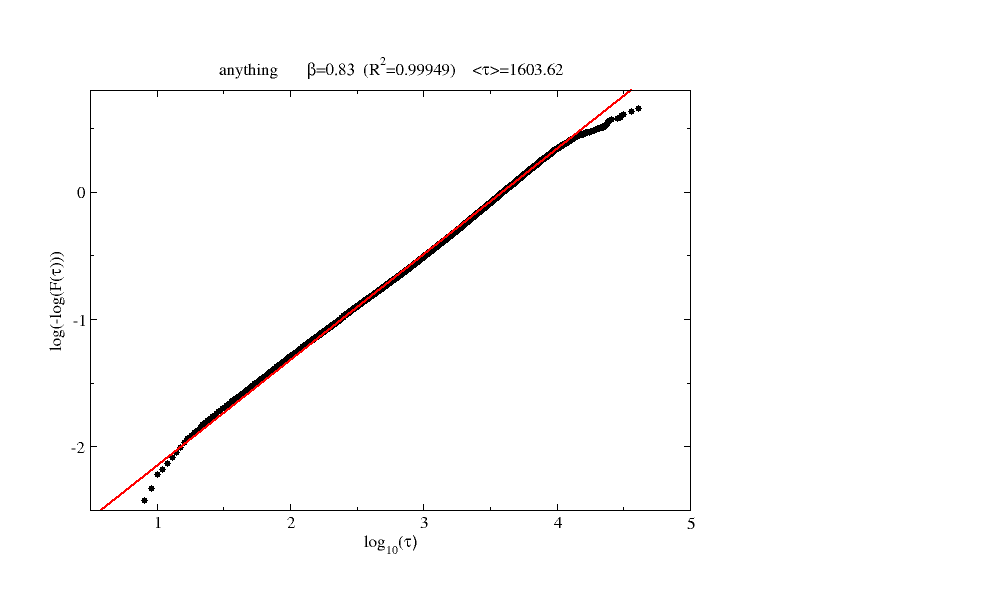

Supplement: Table S1 — Detailed information on the statistical analysis of all words that were studied (six databases). (31.88 MB TAR) [file pone.0007678.s002.tar › recurrence/comp/anything.png]

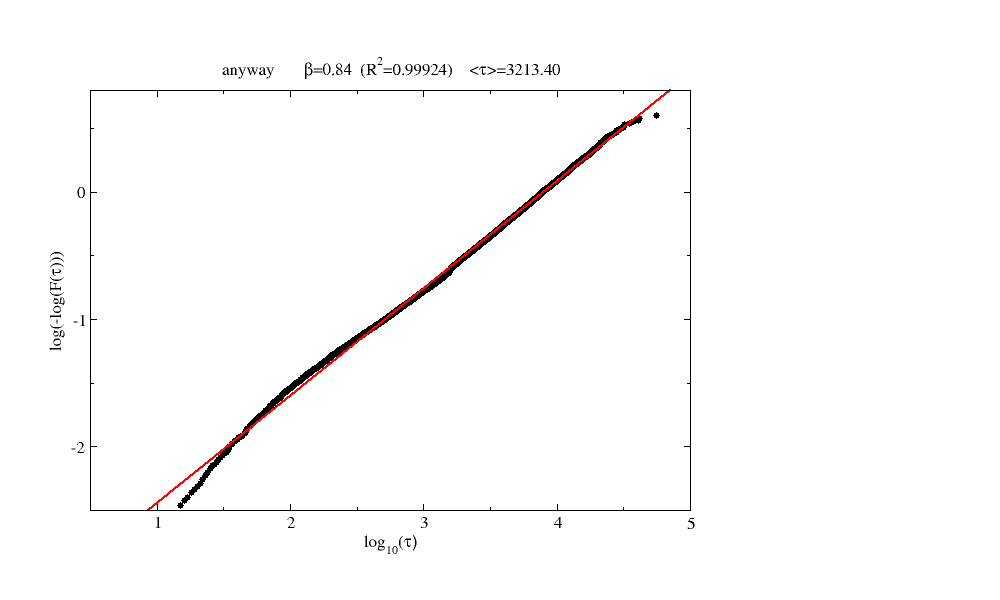

Supplement: Table S1 — Detailed information on the statistical analysis of all words that were studied (six databases). (31.88 MB TAR) [file pone.0007678.s002.tar › recurrence/comp/anyway.png]

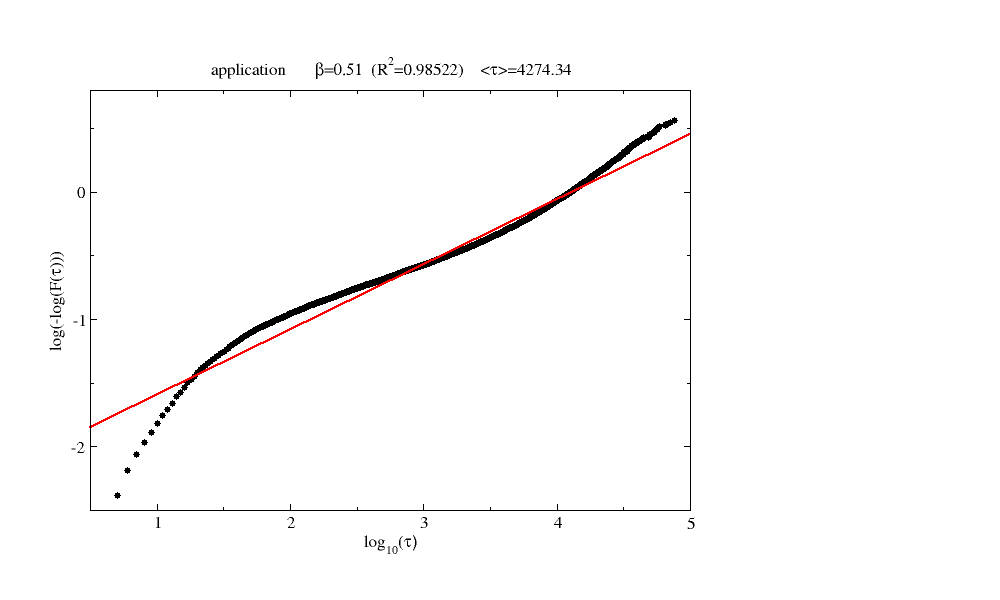

Supplement: Table S1 — Detailed information on the statistical analysis of all words that were studied (six databases). (31.88 MB TAR) [file pone.0007678.s002.tar › recurrence/comp/application.png]

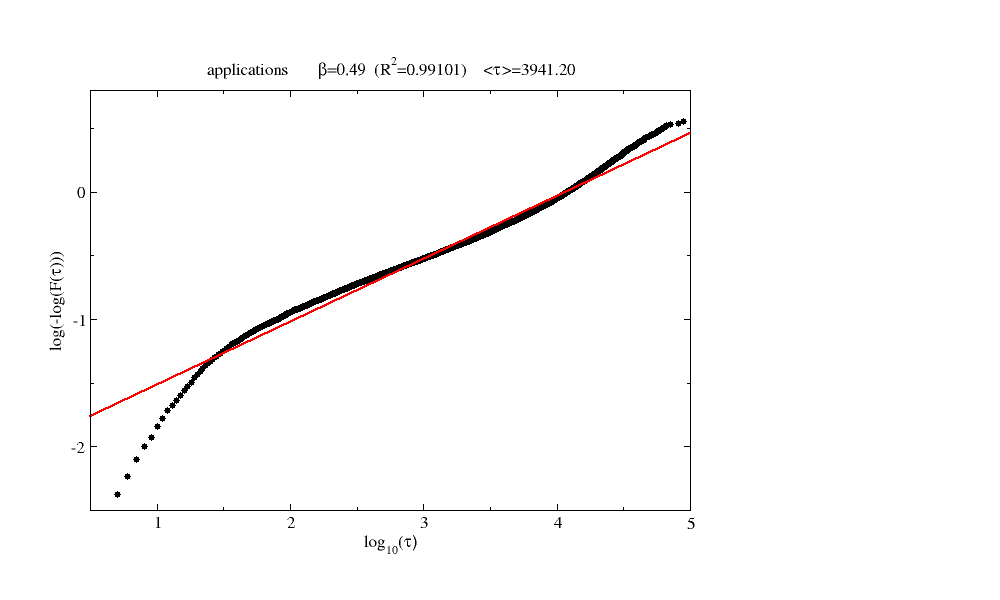

Supplement: Table S1 — Detailed information on the statistical analysis of all words that were studied (six databases). (31.88 MB TAR) [file pone.0007678.s002.tar › recurrence/comp/applications.png]

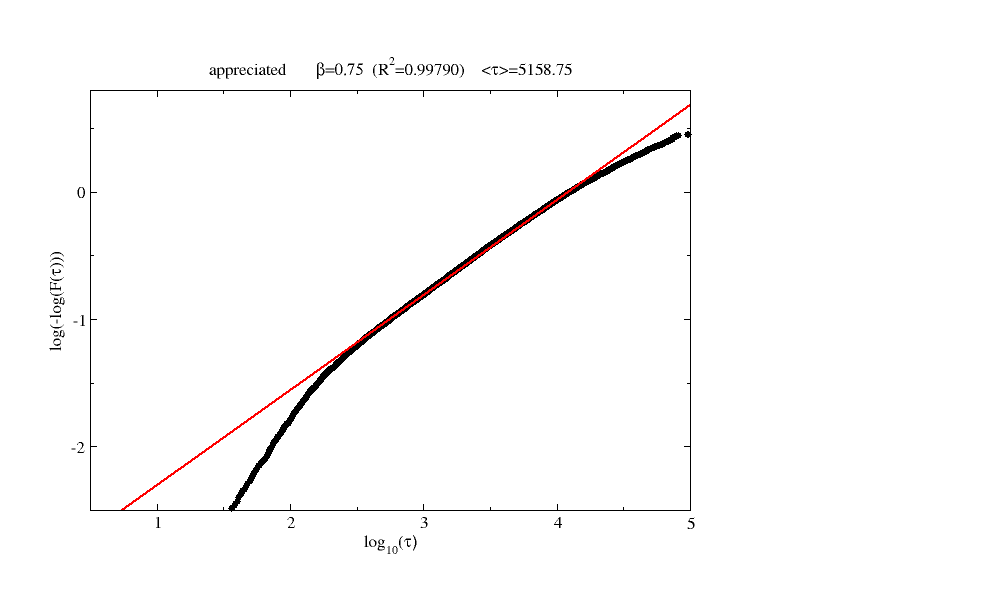

Supplement: Table S1 — Detailed information on the statistical analysis of all words that were studied (six databases). (31.88 MB TAR) [file pone.0007678.s002.tar › recurrence/comp/appreciated.png]

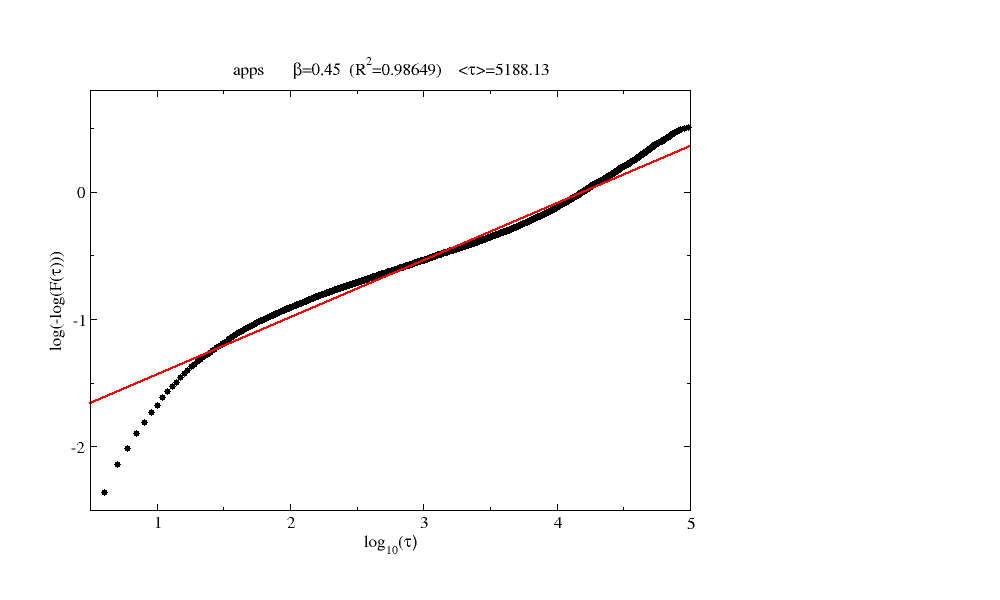

Supplement: Table S1 — Detailed information on the statistical analysis of all words that were studied (six databases). (31.88 MB TAR) [file pone.0007678.s002.tar › recurrence/comp/apps.png]

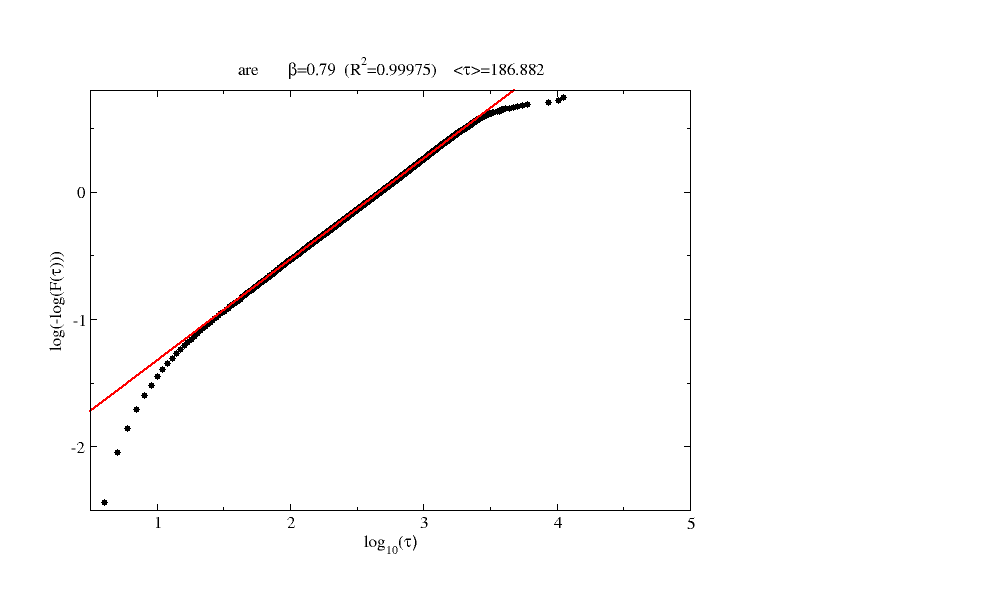

Supplement: Table S1 — Detailed information on the statistical analysis of all words that were studied (six databases). (31.88 MB TAR) [file pone.0007678.s002.tar › recurrence/comp/are.png]

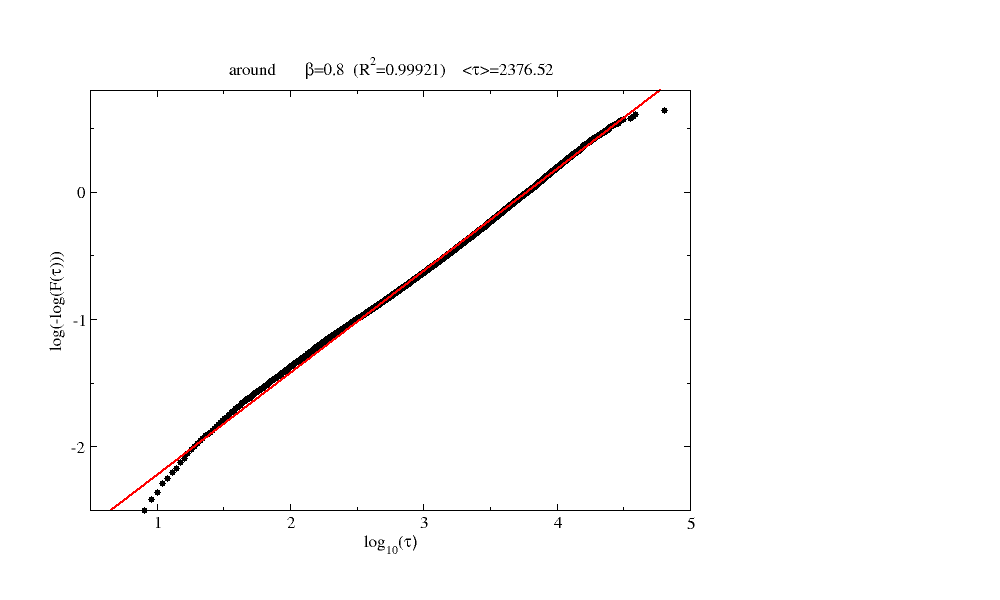

Supplement: Table S1 — Detailed information on the statistical analysis of all words that were studied (six databases). (31.88 MB TAR) [file pone.0007678.s002.tar › recurrence/comp/around.png]

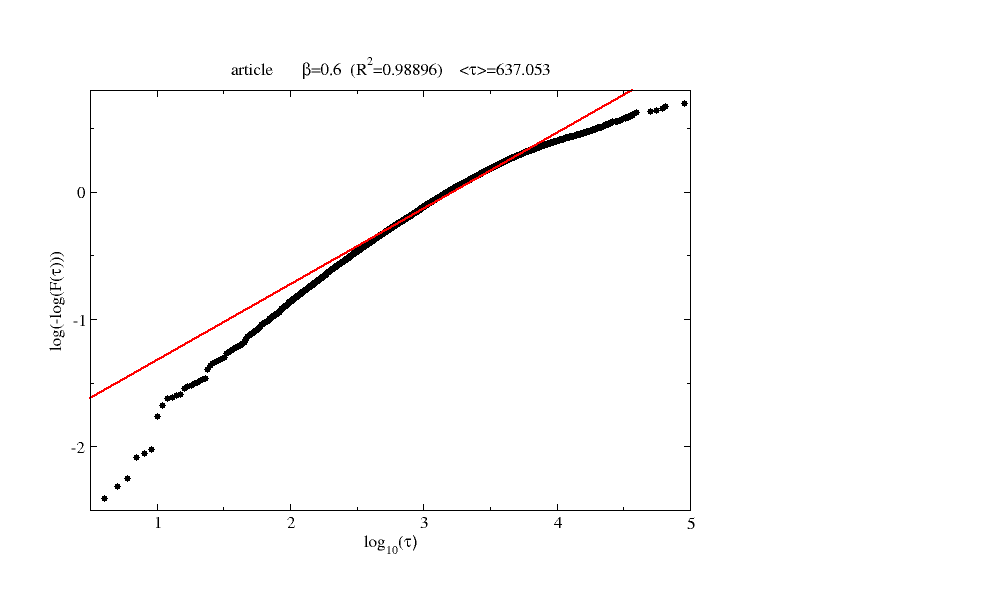

Supplement: Table S1 — Detailed information on the statistical analysis of all words that were studied (six databases). (31.88 MB TAR) [file pone.0007678.s002.tar › recurrence/comp/article.png]

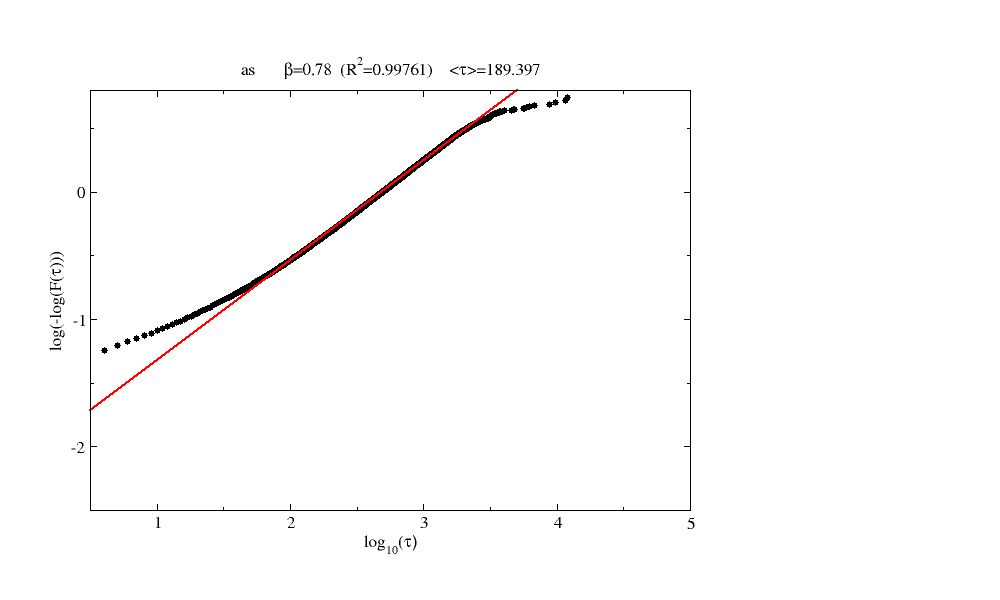

Supplement: Table S1 — Detailed information on the statistical analysis of all words that were studied (six databases). (31.88 MB TAR) [file pone.0007678.s002.tar › recurrence/comp/as.png]

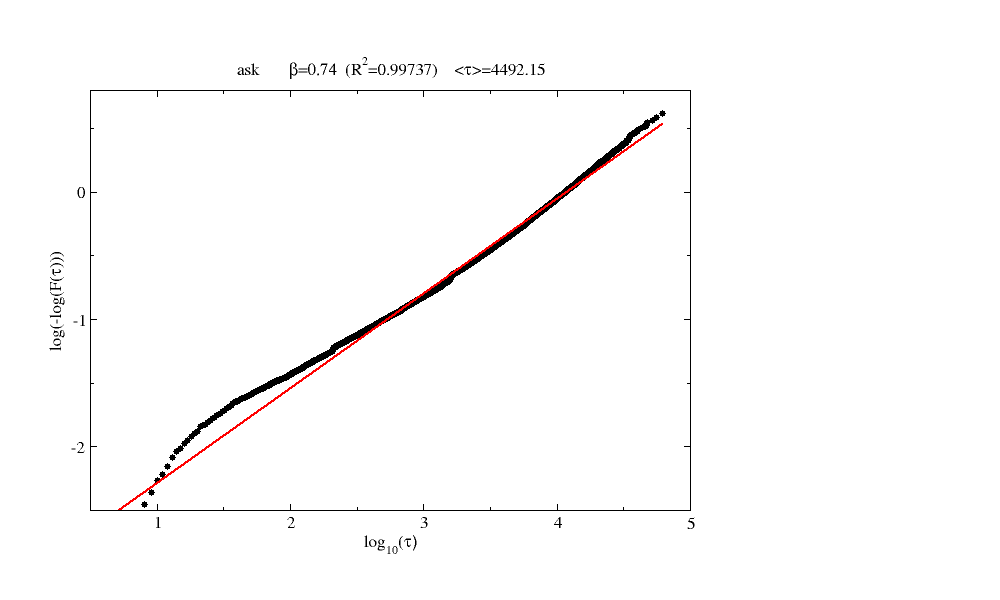

Supplement: Table S1 — Detailed information on the statistical analysis of all words that were studied (six databases). (31.88 MB TAR) [file pone.0007678.s002.tar › recurrence/comp/ask.png]

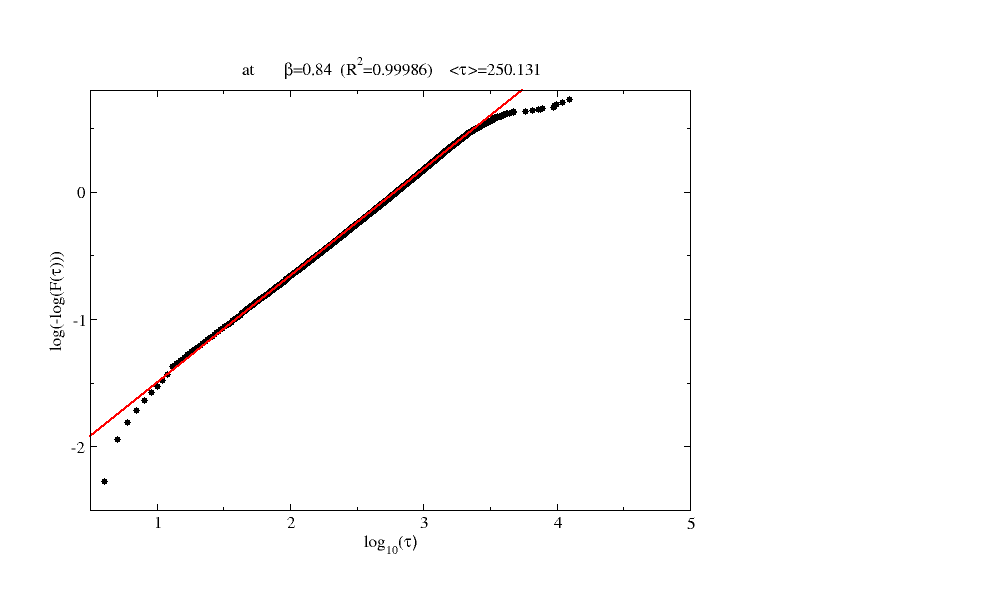

Supplement: Table S1 — Detailed information on the statistical analysis of all words that were studied (six databases). (31.88 MB TAR) [file pone.0007678.s002.tar › recurrence/comp/at.png]

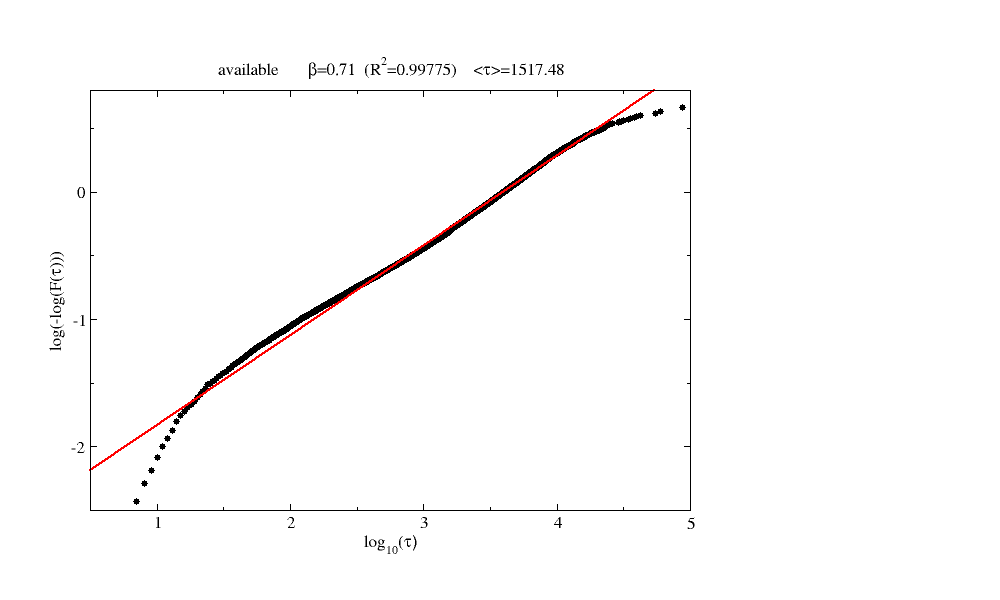

Supplement: Table S1 — Detailed information on the statistical analysis of all words that were studied (six databases). (31.88 MB TAR) [file pone.0007678.s002.tar › recurrence/comp/available.png]

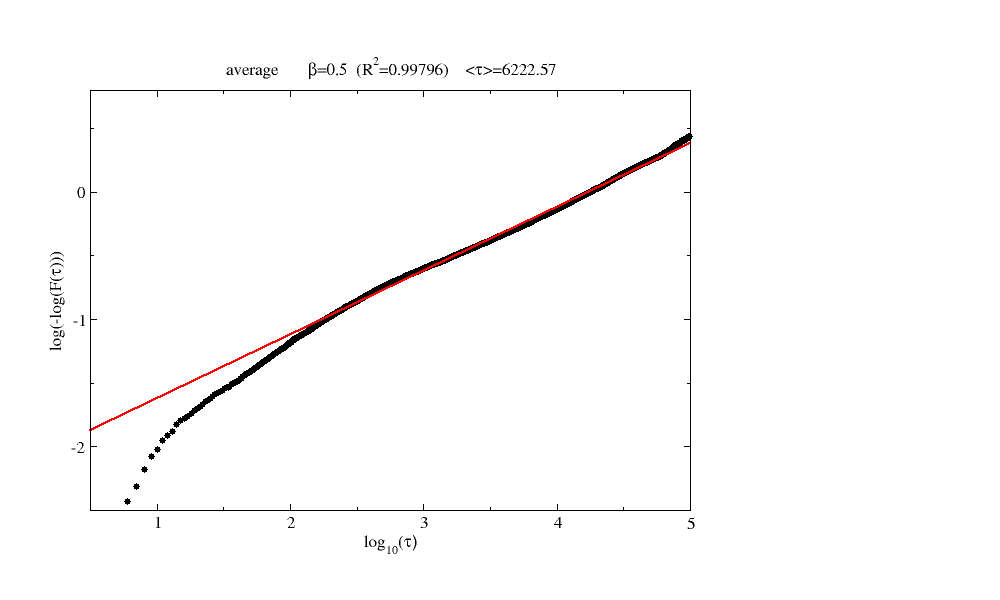

Supplement: Table S1 — Detailed information on the statistical analysis of all words that were studied (six databases). (31.88 MB TAR) [file pone.0007678.s002.tar › recurrence/comp/average.png]

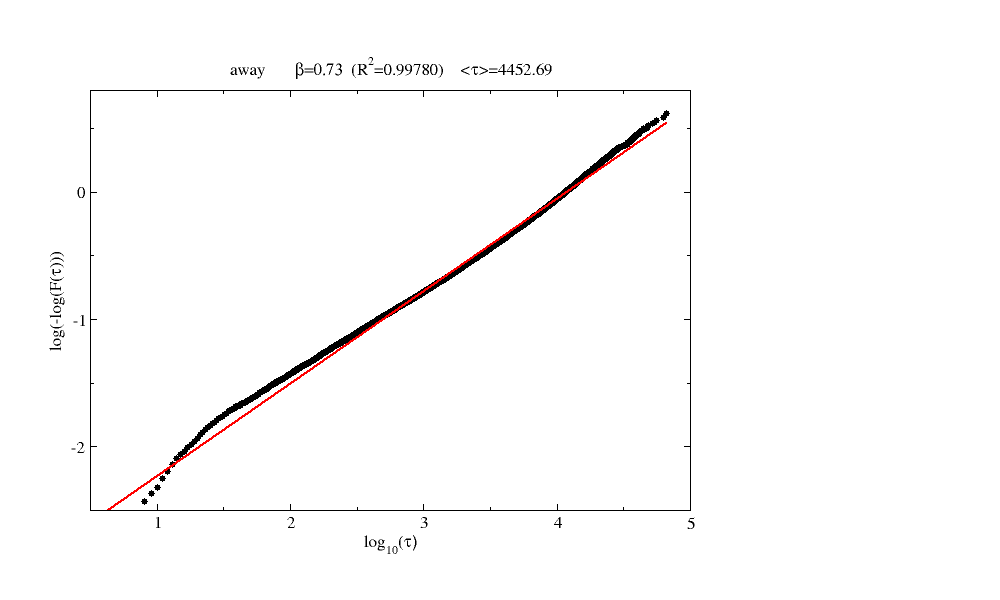

Supplement: Table S1 — Detailed information on the statistical analysis of all words that were studied (six databases). (31.88 MB TAR) [file pone.0007678.s002.tar › recurrence/comp/away.png]

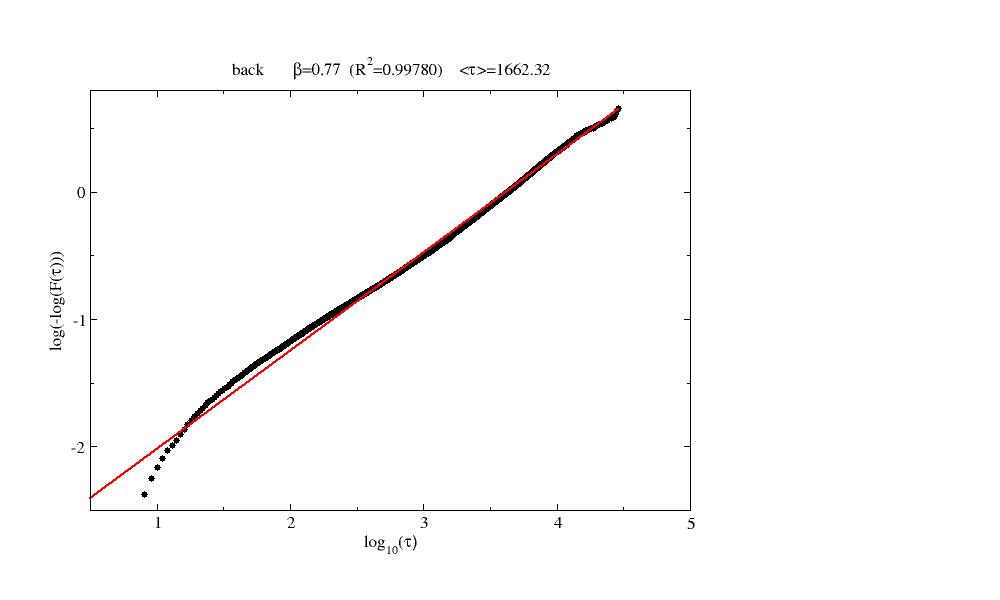

Supplement: Table S1 — Detailed information on the statistical analysis of all words that were studied (six databases). (31.88 MB TAR) [file pone.0007678.s002.tar › recurrence/comp/back.png]

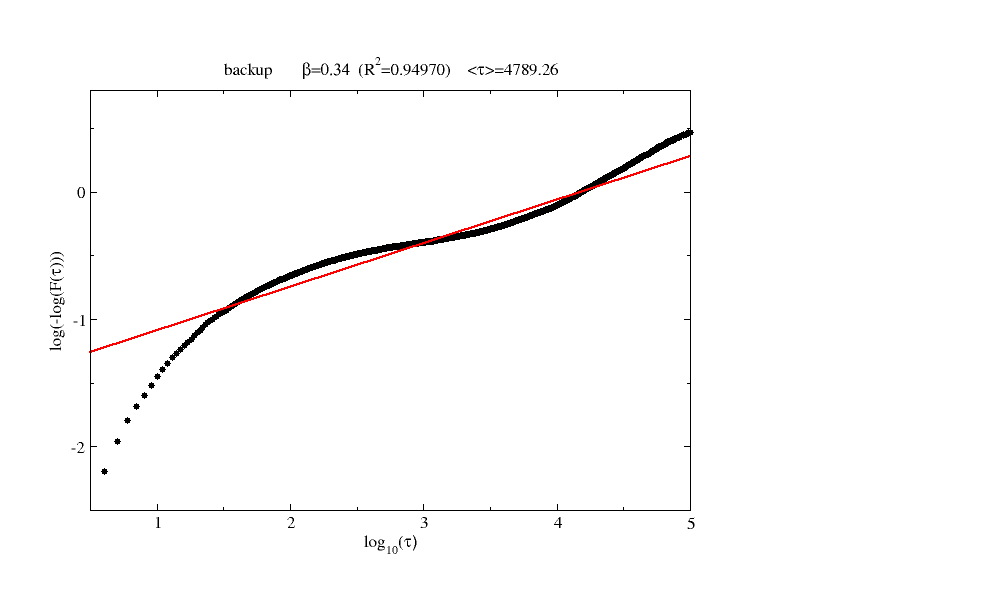

Supplement: Table S1 — Detailed information on the statistical analysis of all words that were studied (six databases). (31.88 MB TAR) [file pone.0007678.s002.tar › recurrence/comp/backup.png]

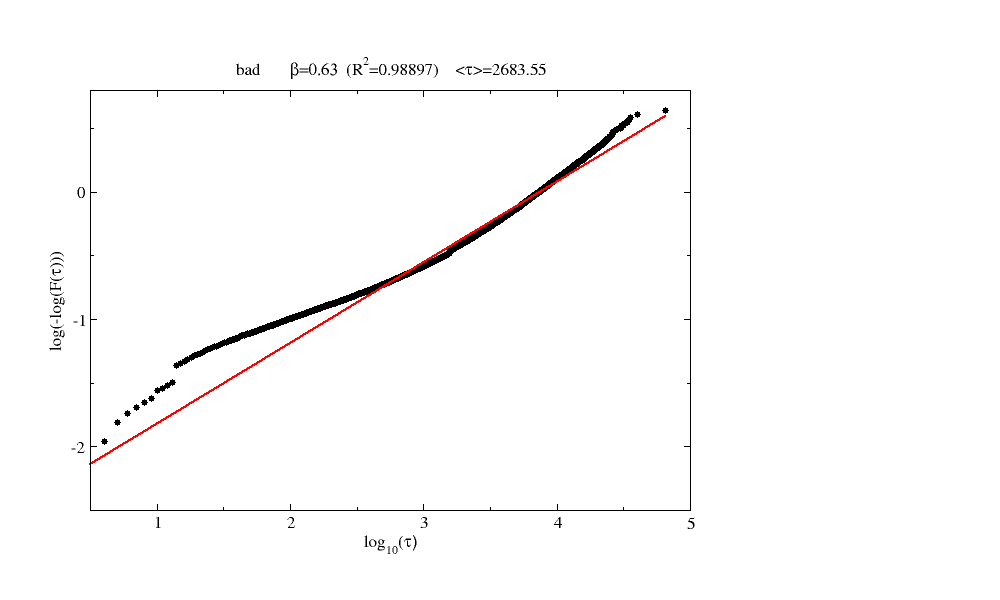

Supplement: Table S1 — Detailed information on the statistical analysis of all words that were studied (six databases). (31.88 MB TAR) [file pone.0007678.s002.tar › recurrence/comp/bad.png]

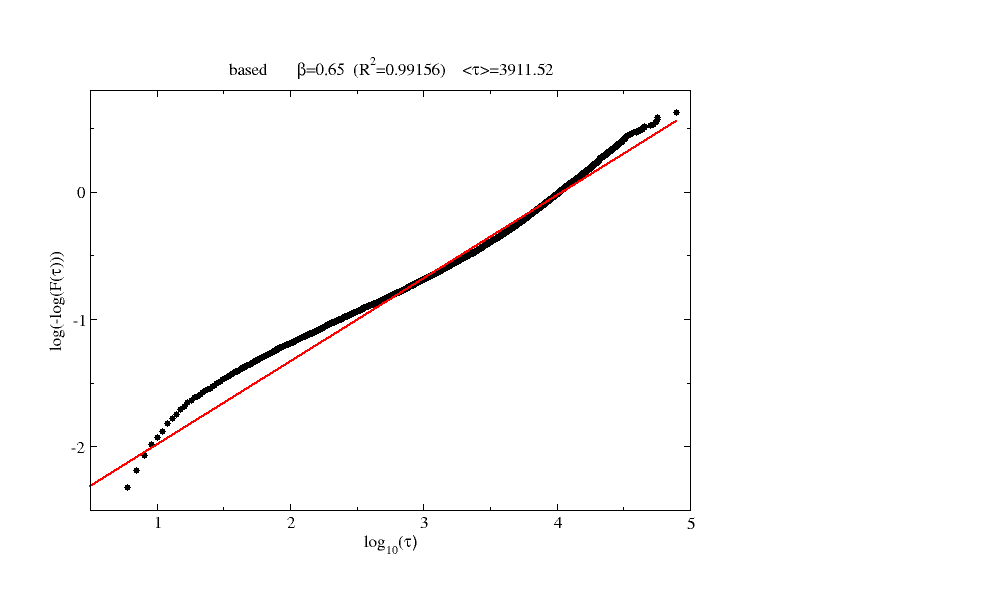

Supplement: Table S1 — Detailed information on the statistical analysis of all words that were studied (six databases). (31.88 MB TAR) [file pone.0007678.s002.tar › recurrence/comp/based.png]

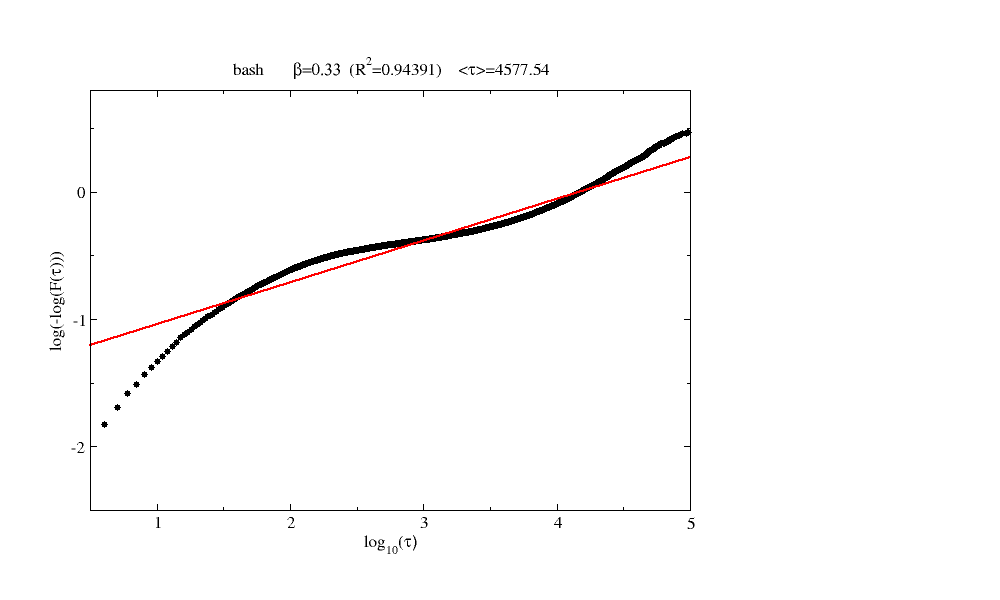

Supplement: Table S1 — Detailed information on the statistical analysis of all words that were studied (six databases). (31.88 MB TAR) [file pone.0007678.s002.tar › recurrence/comp/bash.png]

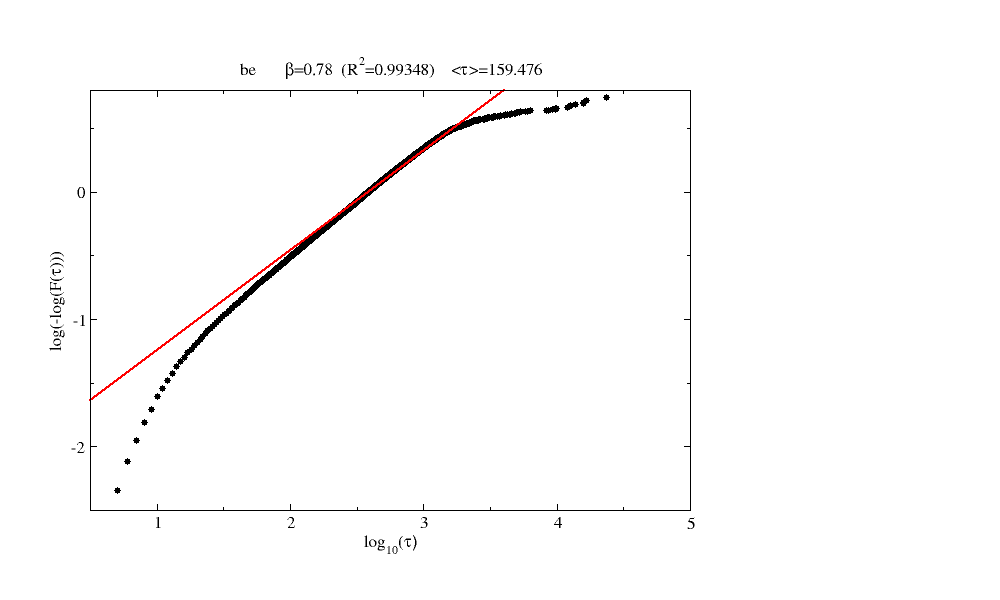

Supplement: Table S1 — Detailed information on the statistical analysis of all words that were studied (six databases). (31.88 MB TAR) [file pone.0007678.s002.tar › recurrence/comp/be.png]

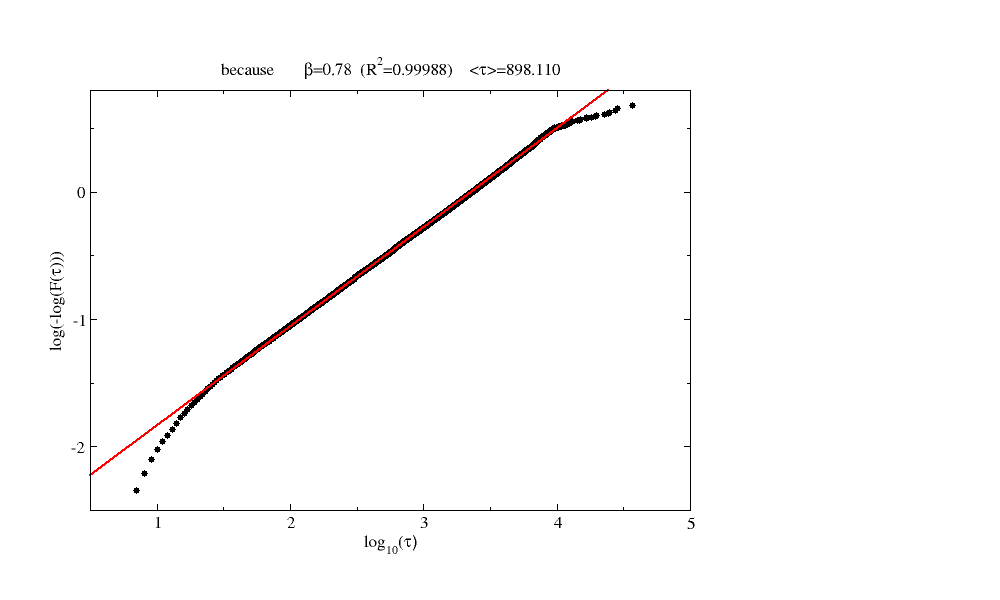

Supplement: Table S1 — Detailed information on the statistical analysis of all words that were studied (six databases). (31.88 MB TAR) [file pone.0007678.s002.tar › recurrence/comp/because.png]

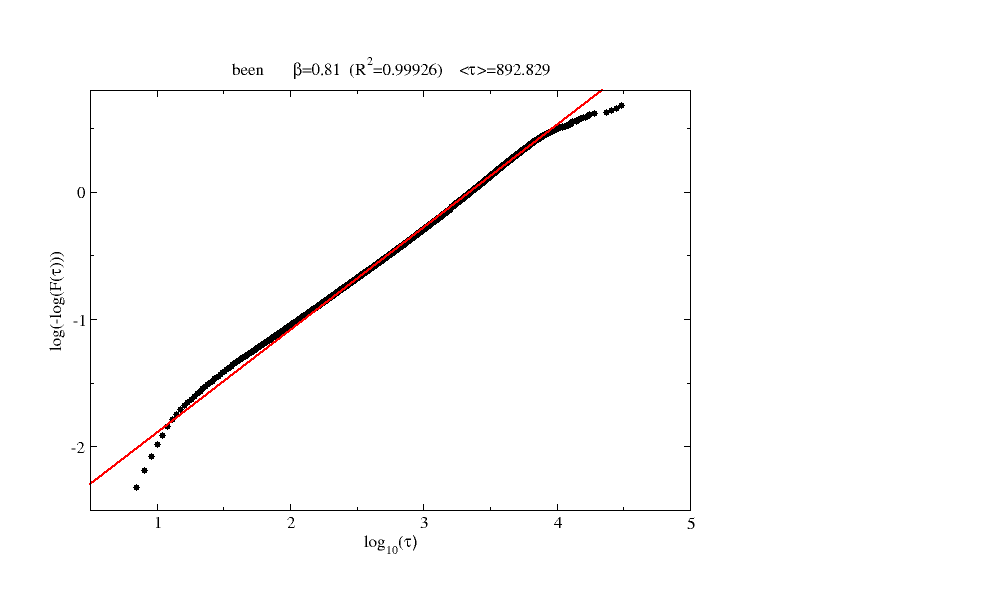

Supplement: Table S1 — Detailed information on the statistical analysis of all words that were studied (six databases). (31.88 MB TAR) [file pone.0007678.s002.tar › recurrence/comp/been.png]

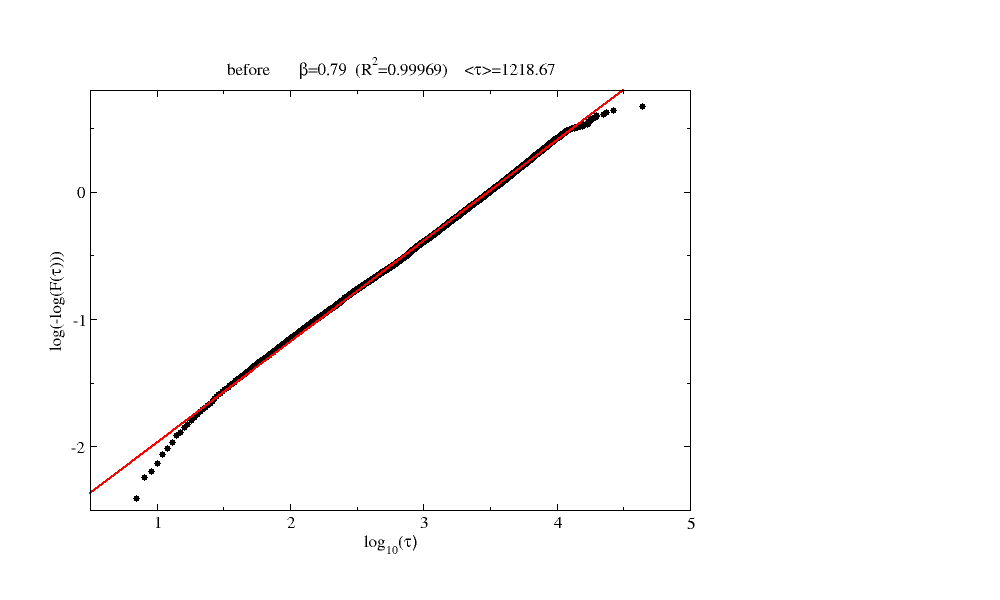

Supplement: Table S1 — Detailed information on the statistical analysis of all words that were studied (six databases). (31.88 MB TAR) [file pone.0007678.s002.tar › recurrence/comp/before.png]

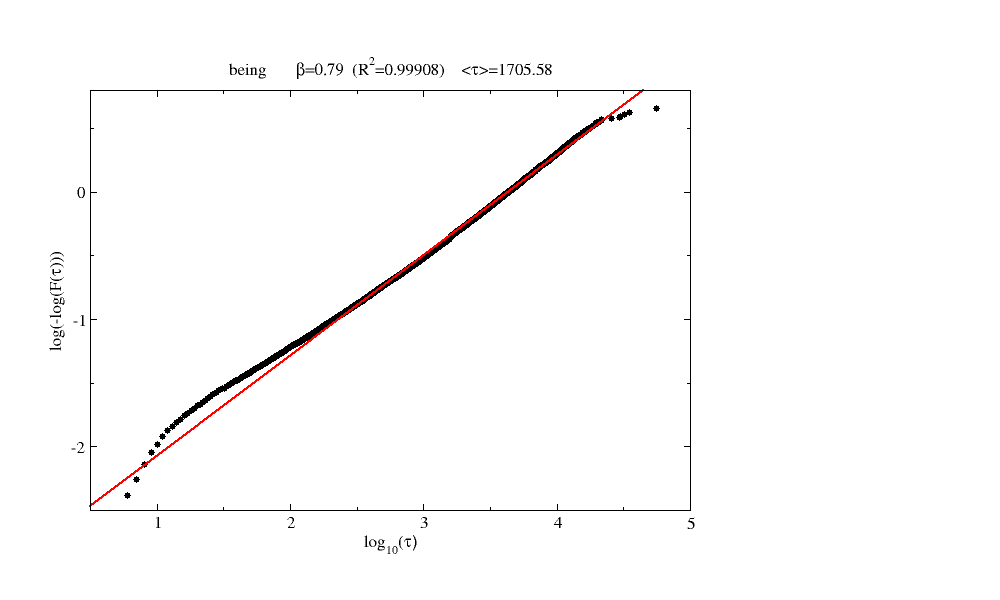

Supplement: Table S1 — Detailed information on the statistical analysis of all words that were studied (six databases). (31.88 MB TAR) [file pone.0007678.s002.tar › recurrence/comp/being.png]

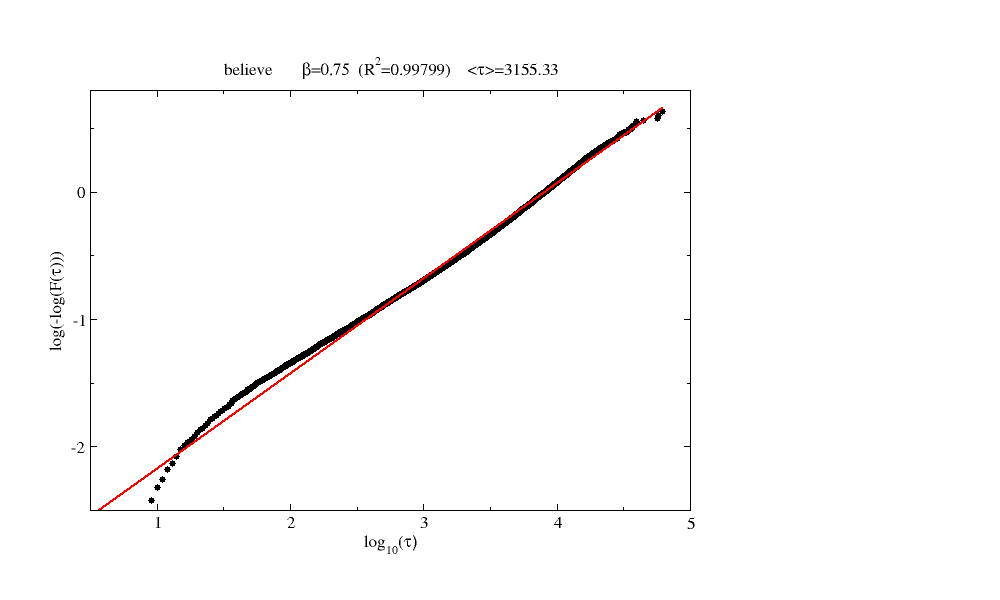

Supplement: Table S1 — Detailed information on the statistical analysis of all words that were studied (six databases). (31.88 MB TAR) [file pone.0007678.s002.tar › recurrence/comp/believe.png]

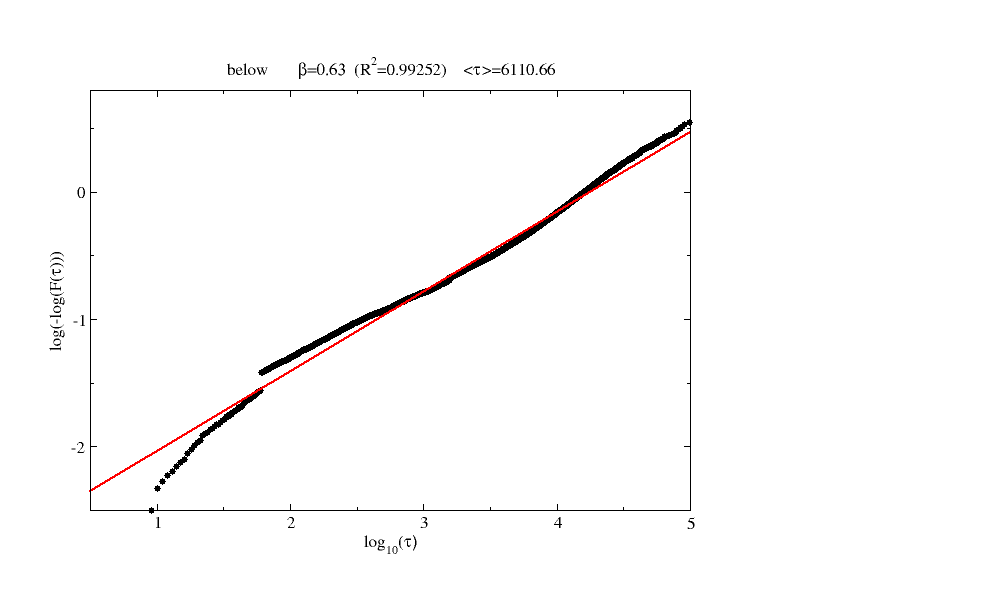

Supplement: Table S1 — Detailed information on the statistical analysis of all words that were studied (six databases). (31.88 MB TAR) [file pone.0007678.s002.tar › recurrence/comp/below.png]

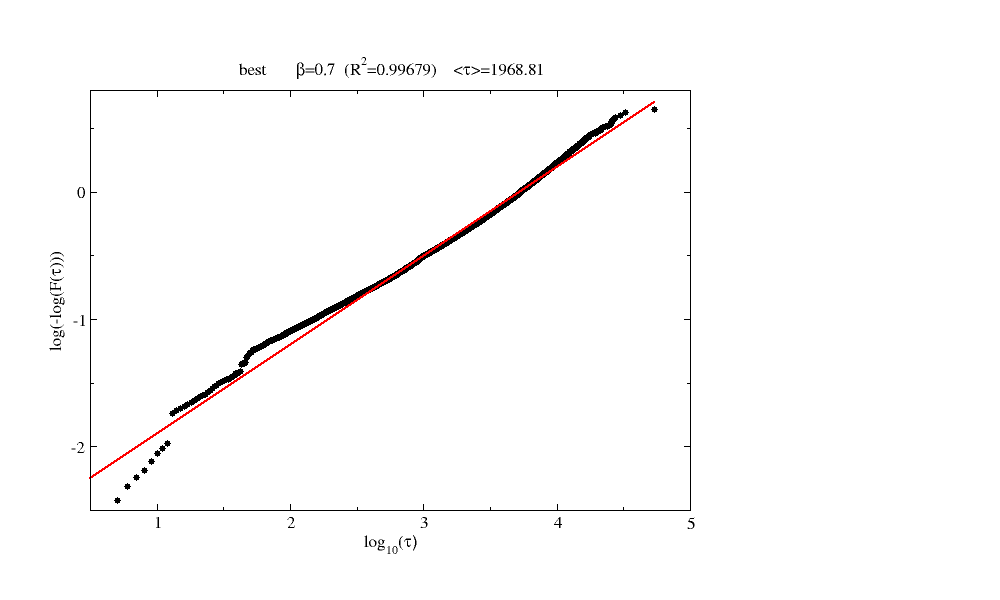

Supplement: Table S1 — Detailed information on the statistical analysis of all words that were studied (six databases). (31.88 MB TAR) [file pone.0007678.s002.tar › recurrence/comp/best.png]

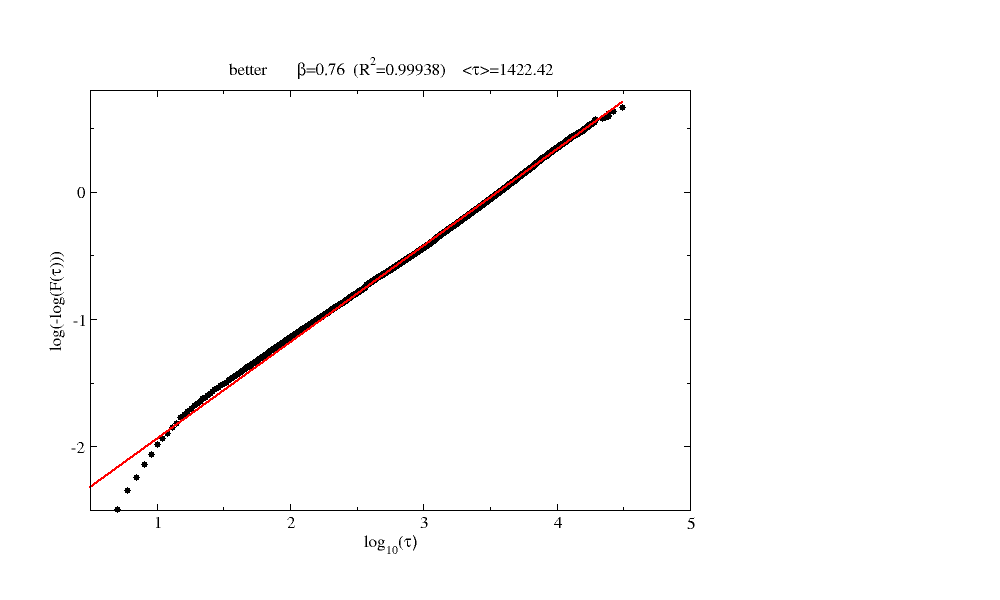

Supplement: Table S1 — Detailed information on the statistical analysis of all words that were studied (six databases). (31.88 MB TAR) [file pone.0007678.s002.tar › recurrence/comp/better.png]

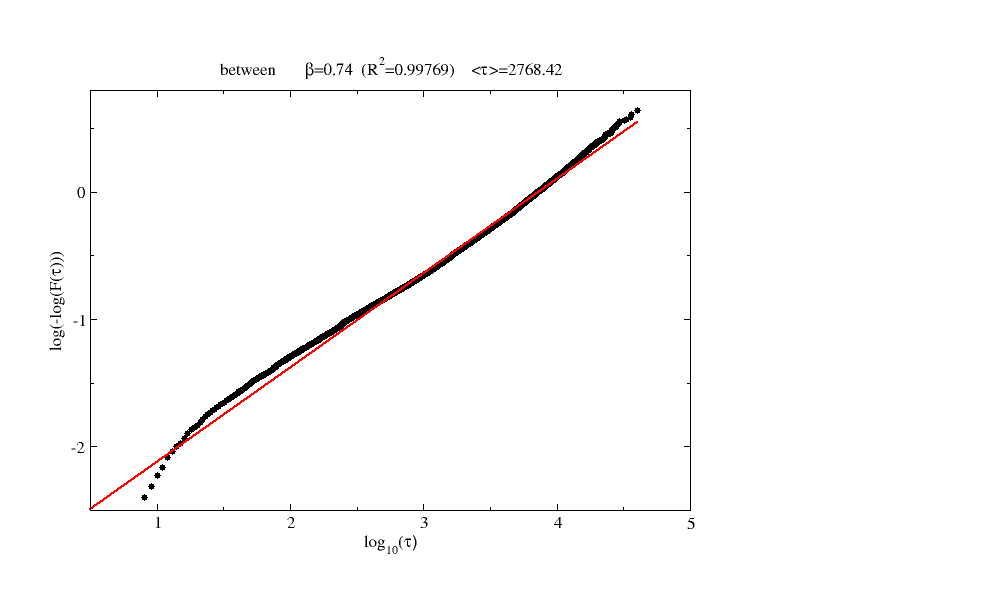

Supplement: Table S1 — Detailed information on the statistical analysis of all words that were studied (six databases). (31.88 MB TAR) [file pone.0007678.s002.tar › recurrence/comp/between.png]

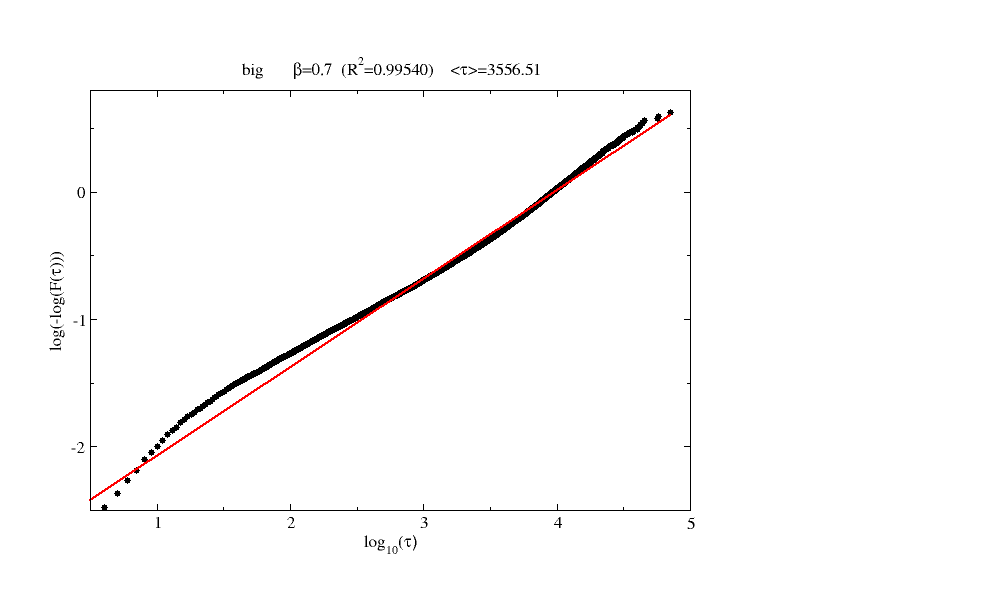

Supplement: Table S1 — Detailed information on the statistical analysis of all words that were studied (six databases). (31.88 MB TAR) [file pone.0007678.s002.tar › recurrence/comp/big.png]

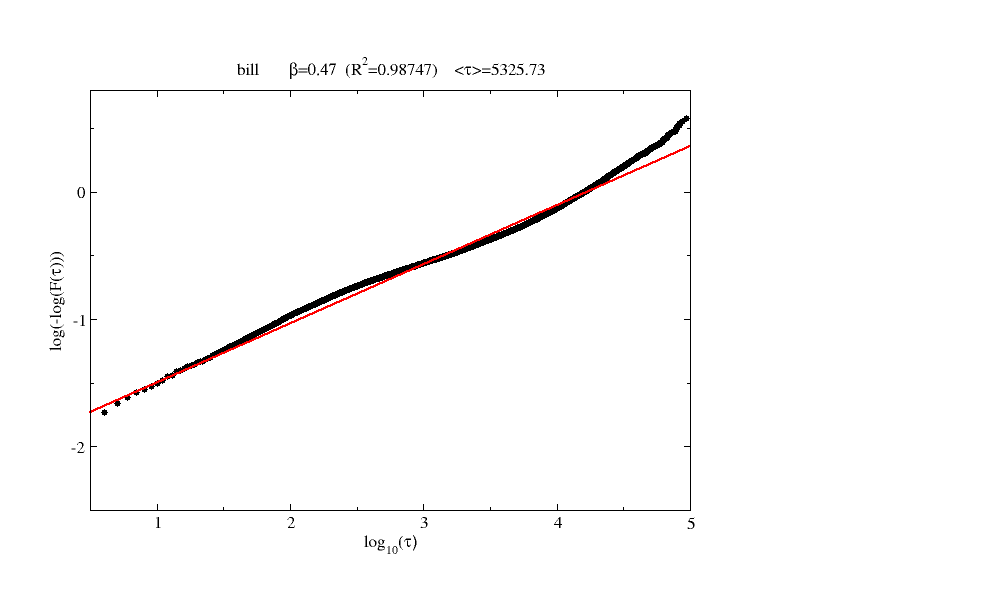

Supplement: Table S1 — Detailed information on the statistical analysis of all words that were studied (six databases). (31.88 MB TAR) [file pone.0007678.s002.tar › recurrence/comp/bill.png]

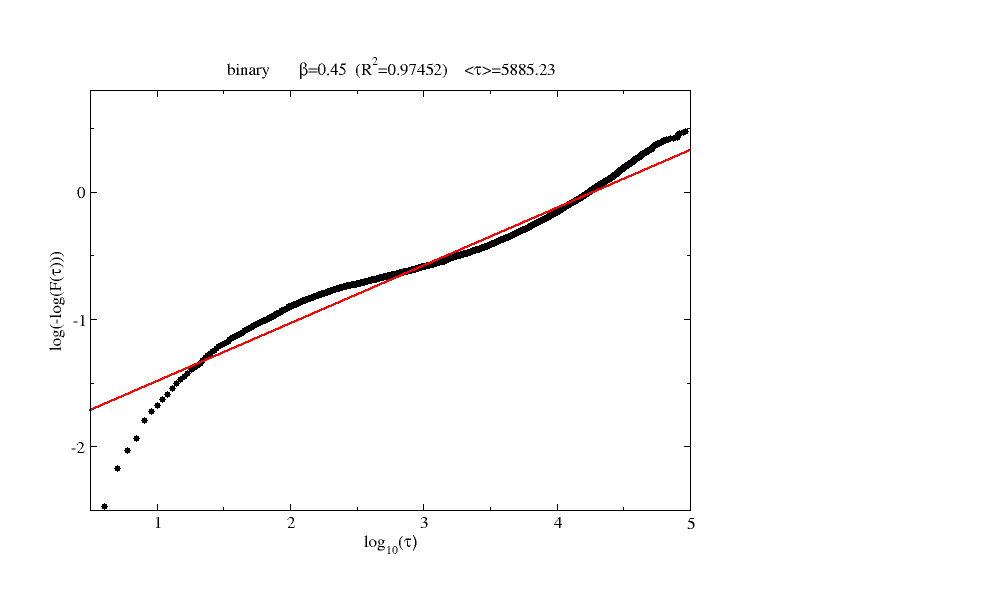

Supplement: Table S1 — Detailed information on the statistical analysis of all words that were studied (six databases). (31.88 MB TAR) [file pone.0007678.s002.tar › recurrence/comp/binary.png]

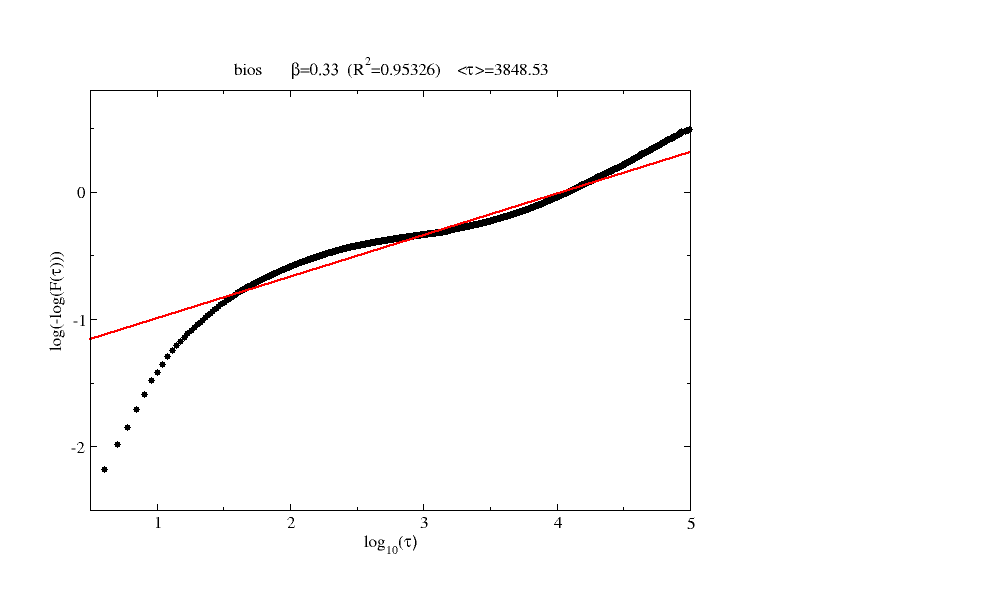

Supplement: Table S1 — Detailed information on the statistical analysis of all words that were studied (six databases). (31.88 MB TAR) [file pone.0007678.s002.tar › recurrence/comp/bios.png]

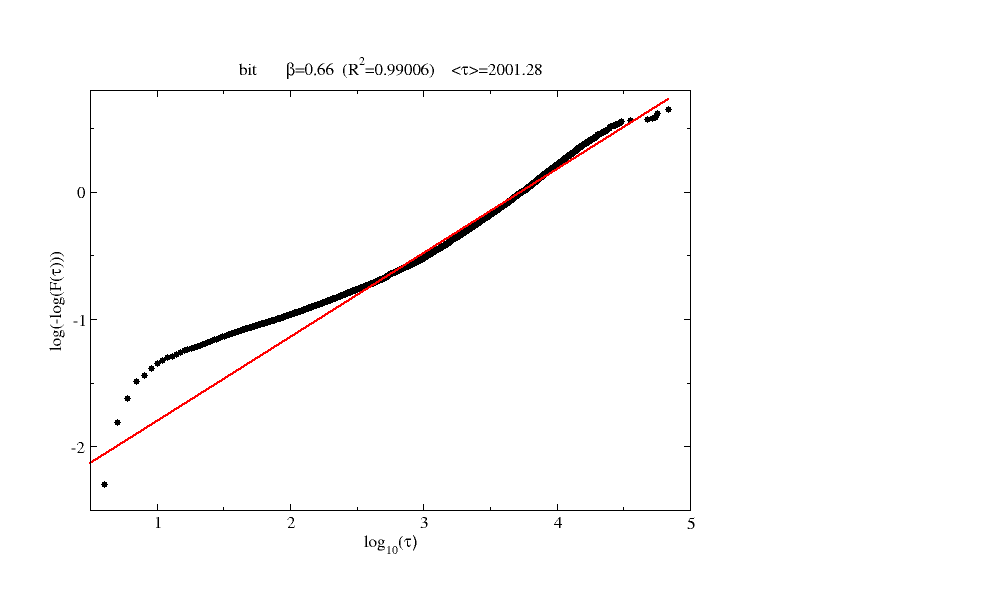

Supplement: Table S1 — Detailed information on the statistical analysis of all words that were studied (six databases). (31.88 MB TAR) [file pone.0007678.s002.tar › recurrence/comp/bit.png]

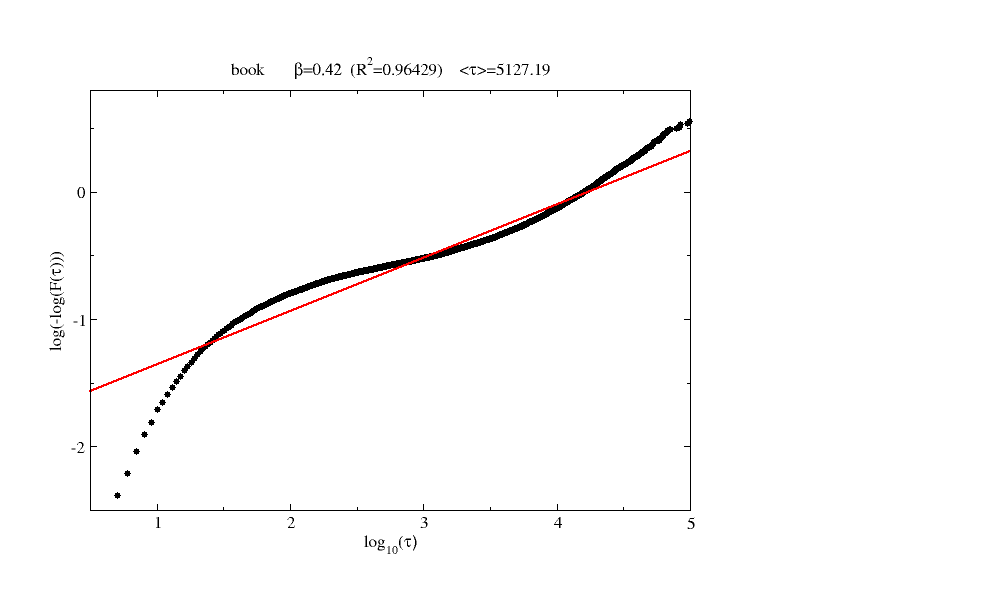

Supplement: Table S1 — Detailed information on the statistical analysis of all words that were studied (six databases). (31.88 MB TAR) [file pone.0007678.s002.tar › recurrence/comp/book.png]

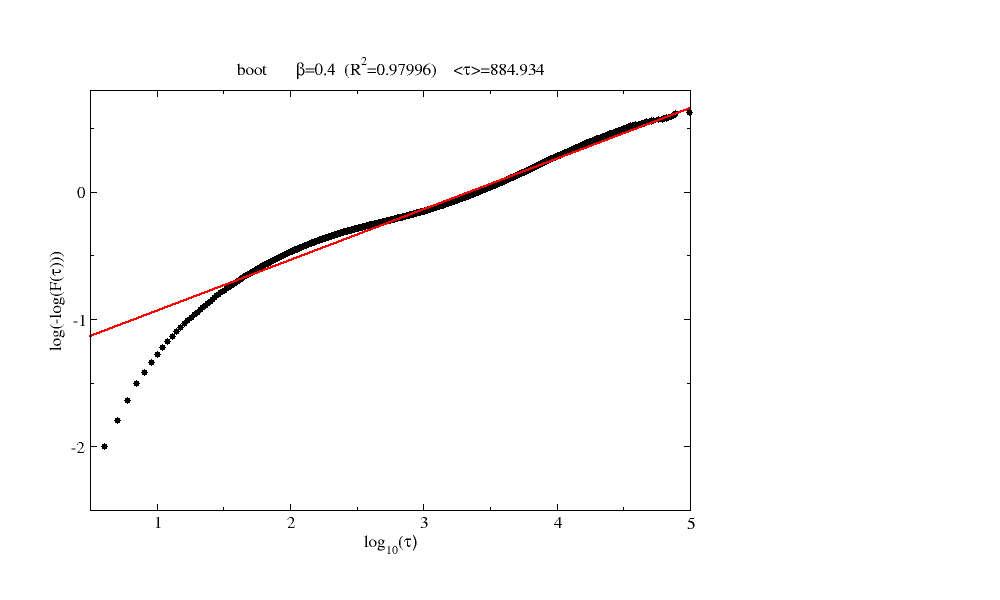

Supplement: Table S1 — Detailed information on the statistical analysis of all words that were studied (six databases). (31.88 MB TAR) [file pone.0007678.s002.tar › recurrence/comp/boot.png]

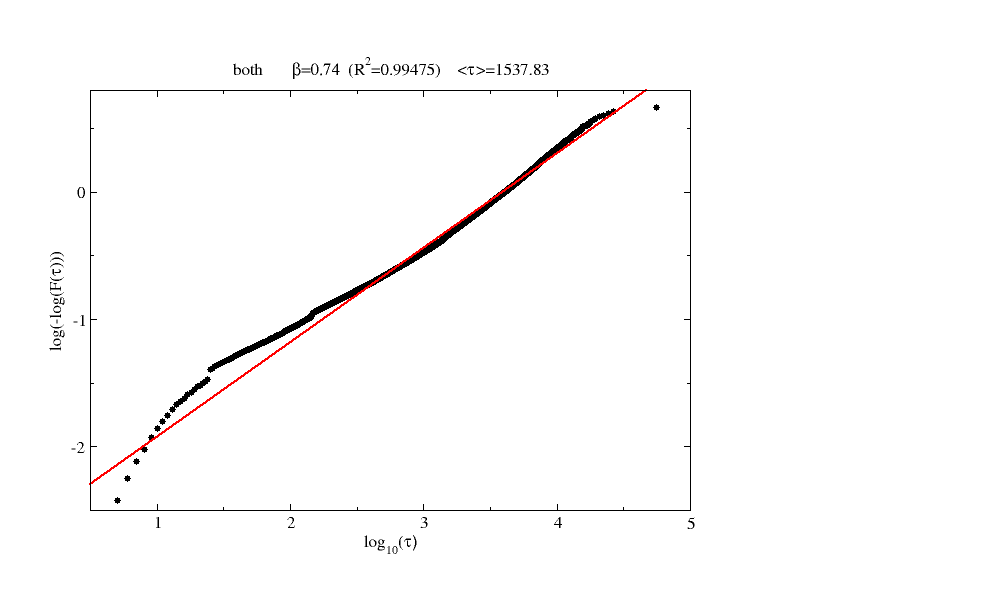

Supplement: Table S1 — Detailed information on the statistical analysis of all words that were studied (six databases). (31.88 MB TAR) [file pone.0007678.s002.tar › recurrence/comp/both.png]

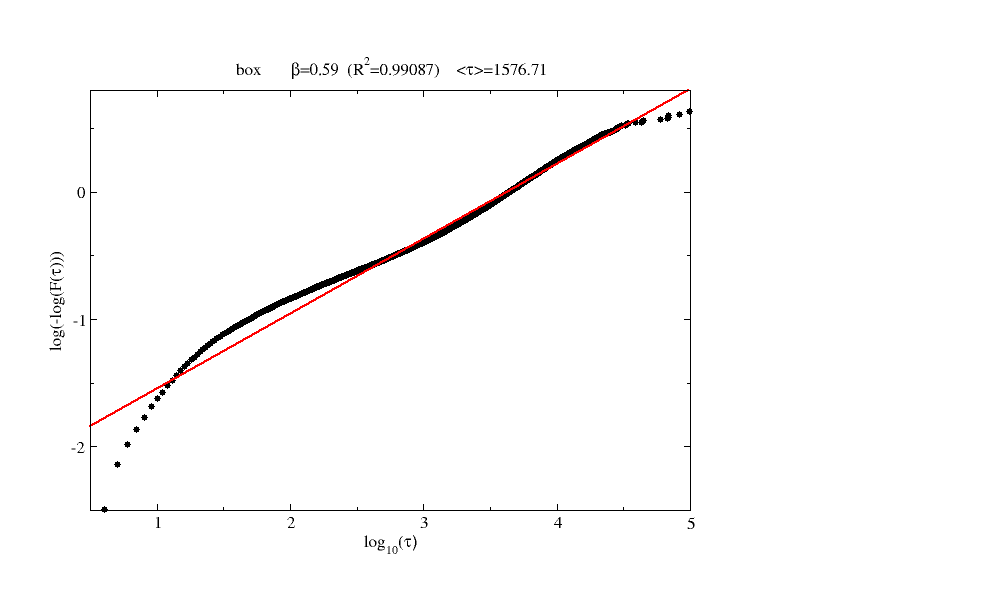

Supplement: Table S1 — Detailed information on the statistical analysis of all words that were studied (six databases). (31.88 MB TAR) [file pone.0007678.s002.tar › recurrence/comp/box.png]

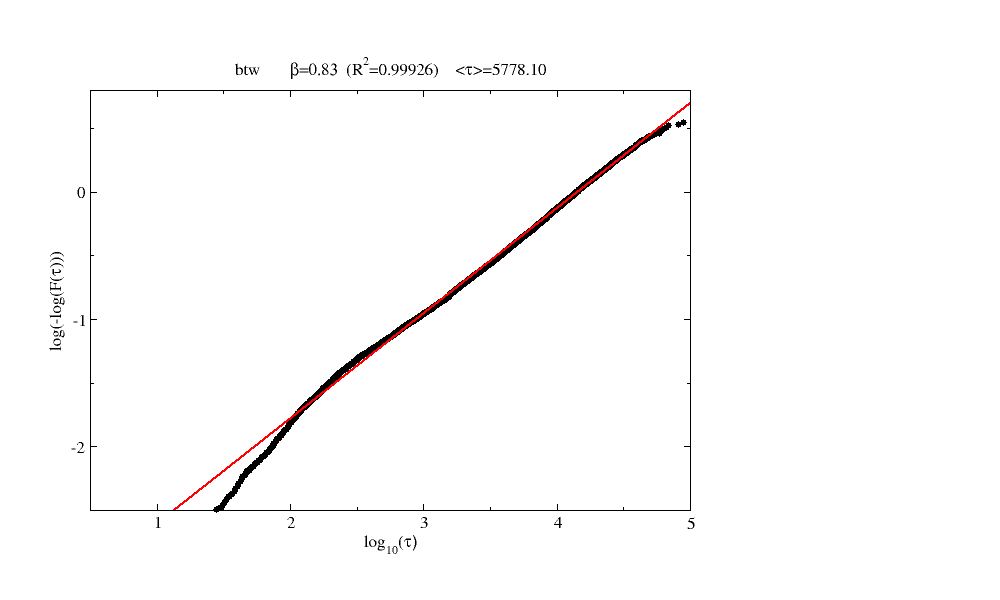

Supplement: Table S1 — Detailed information on the statistical analysis of all words that were studied (six databases). (31.88 MB TAR) [file pone.0007678.s002.tar › recurrence/comp/btw.png]

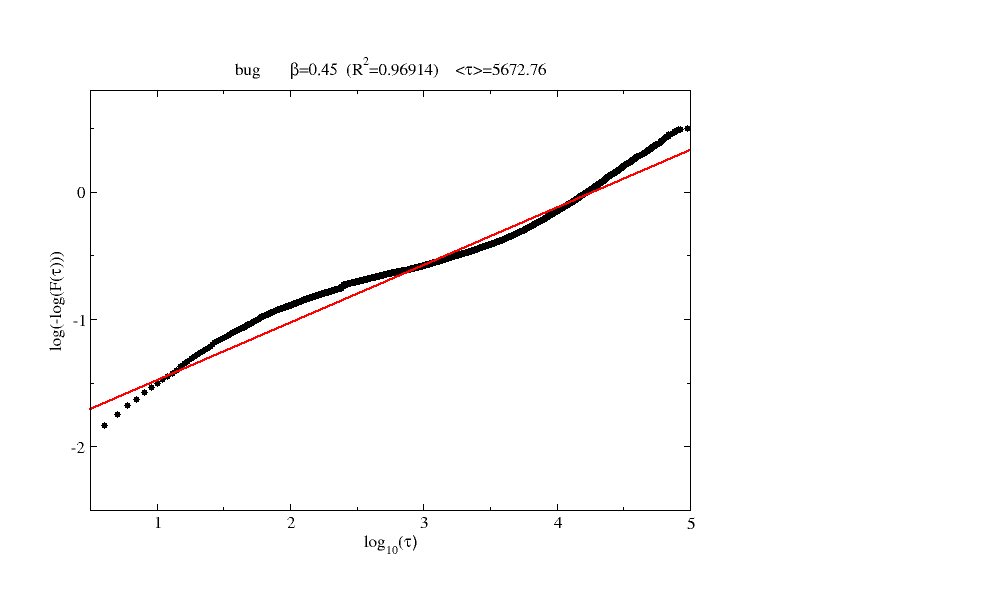

Supplement: Table S1 — Detailed information on the statistical analysis of all words that were studied (six databases). (31.88 MB TAR) [file pone.0007678.s002.tar › recurrence/comp/bug.png]

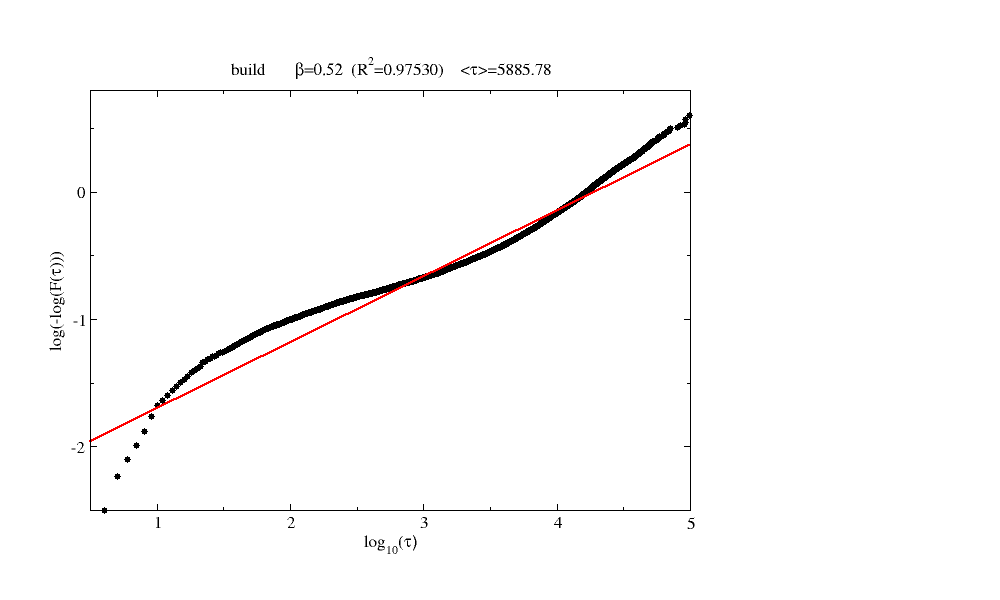

Supplement: Table S1 — Detailed information on the statistical analysis of all words that were studied (six databases). (31.88 MB TAR) [file pone.0007678.s002.tar › recurrence/comp/build.png]

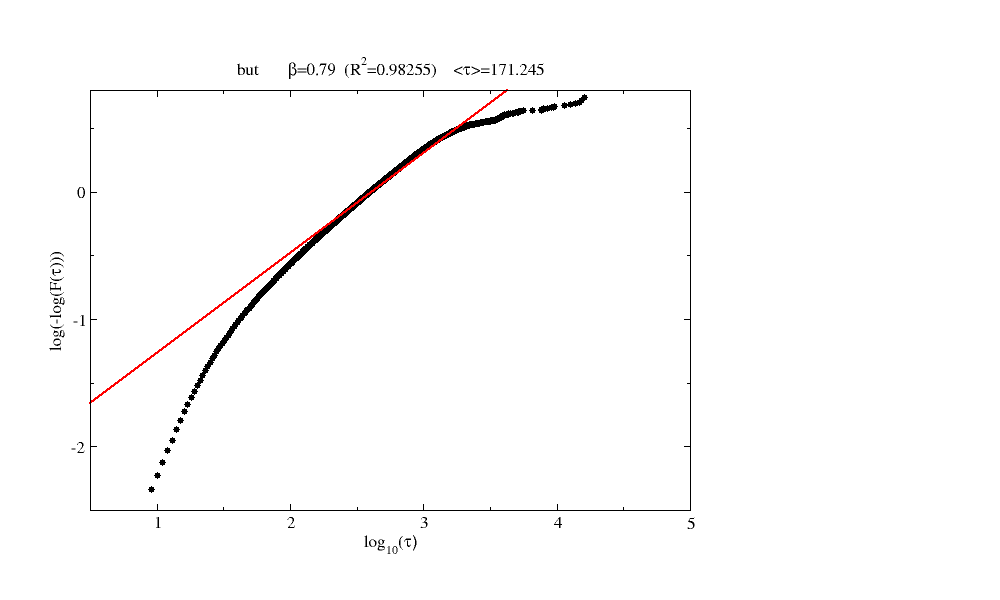

Supplement: Table S1 — Detailed information on the statistical analysis of all words that were studied (six databases). (31.88 MB TAR) [file pone.0007678.s002.tar › recurrence/comp/but.png]

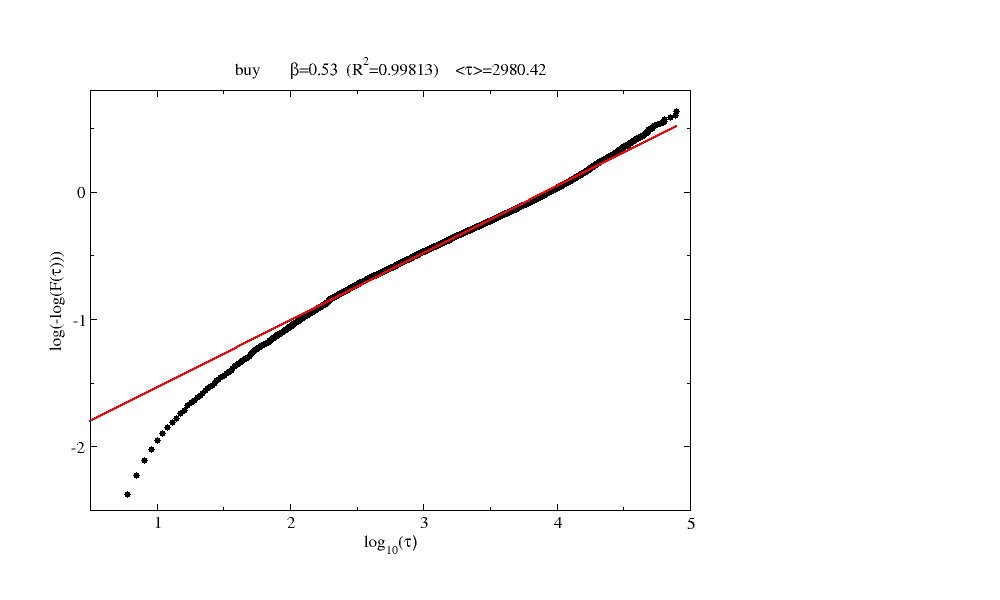

Supplement: Table S1 — Detailed information on the statistical analysis of all words that were studied (six databases). (31.88 MB TAR) [file pone.0007678.s002.tar › recurrence/comp/buy.png]

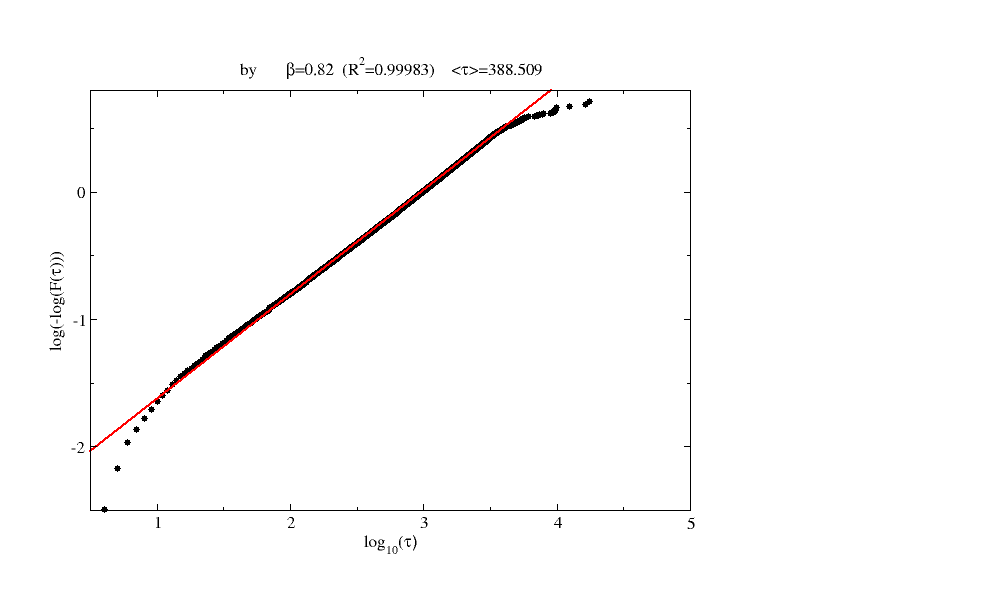

Supplement: Table S1 — Detailed information on the statistical analysis of all words that were studied (six databases). (31.88 MB TAR) [file pone.0007678.s002.tar › recurrence/comp/by.png]

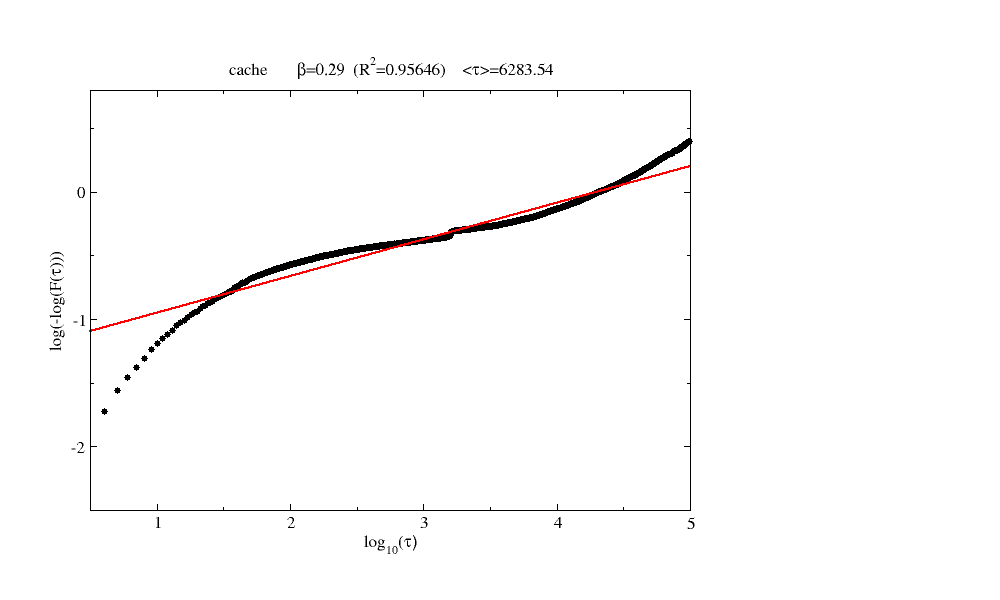

Supplement: Table S1 — Detailed information on the statistical analysis of all words that were studied (six databases). (31.88 MB TAR) [file pone.0007678.s002.tar › recurrence/comp/cache.png]

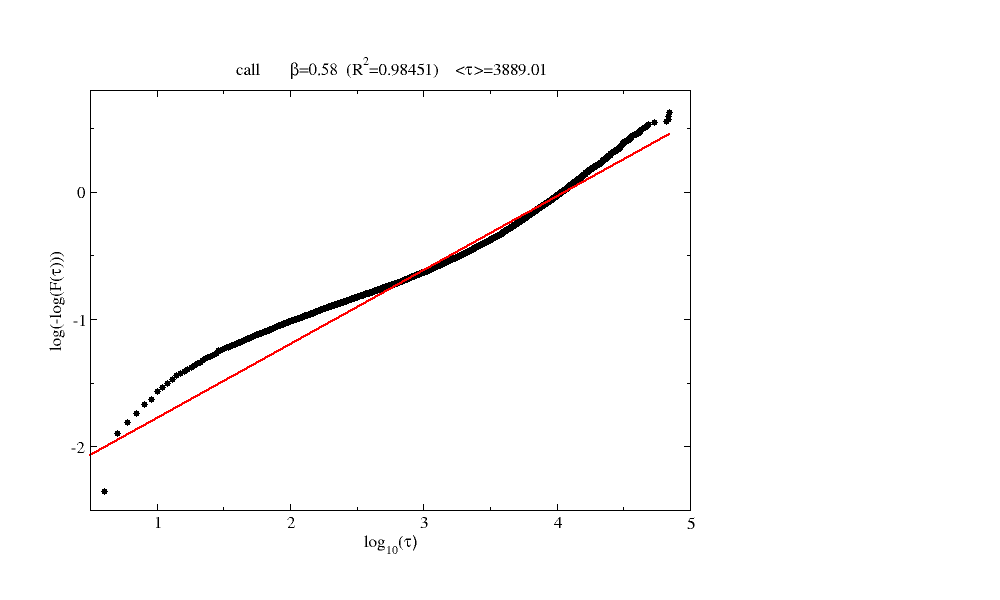

Supplement: Table S1 — Detailed information on the statistical analysis of all words that were studied (six databases). (31.88 MB TAR) [file pone.0007678.s002.tar › recurrence/comp/call.png]

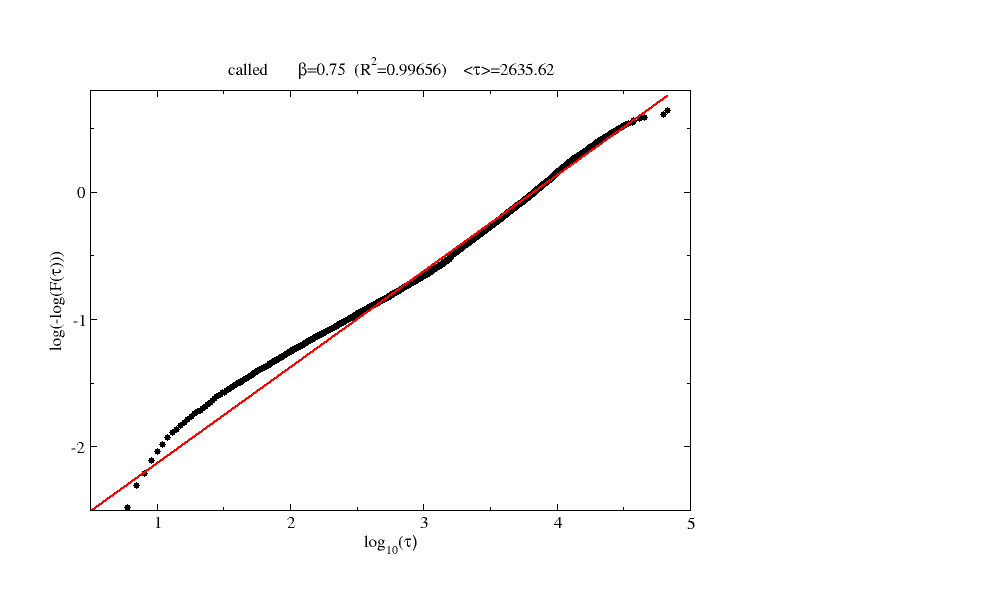

Supplement: Table S1 — Detailed information on the statistical analysis of all words that were studied (six databases). (31.88 MB TAR) [file pone.0007678.s002.tar › recurrence/comp/called.png]

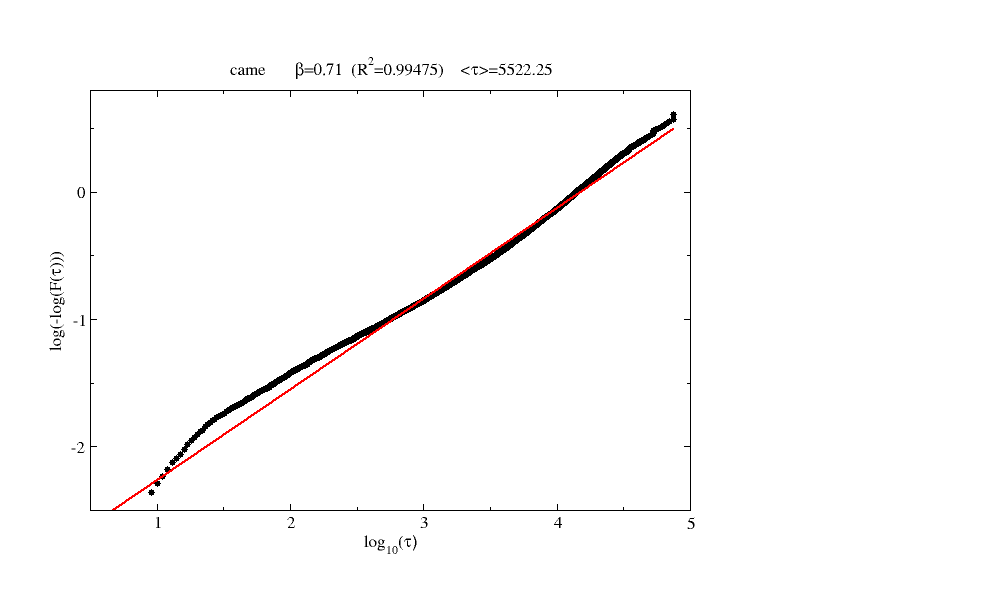

Supplement: Table S1 — Detailed information on the statistical analysis of all words that were studied (six databases). (31.88 MB TAR) [file pone.0007678.s002.tar › recurrence/comp/came.png]

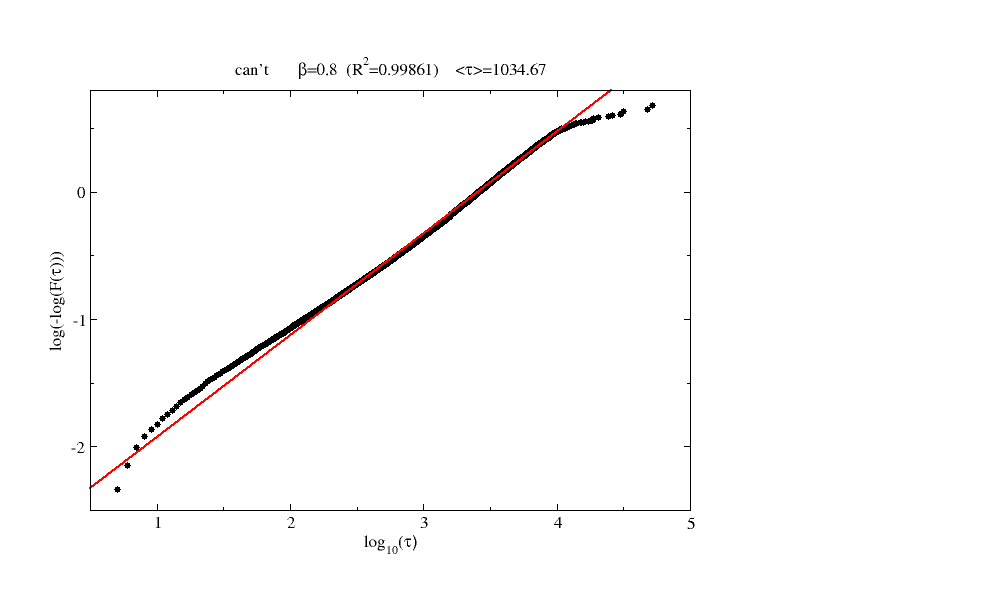

Supplement: Table S1 — Detailed information on the statistical analysis of all words that were studied (six databases). (31.88 MB TAR) [file pone.0007678.s002.tar › recurrence/comp/can't.png]

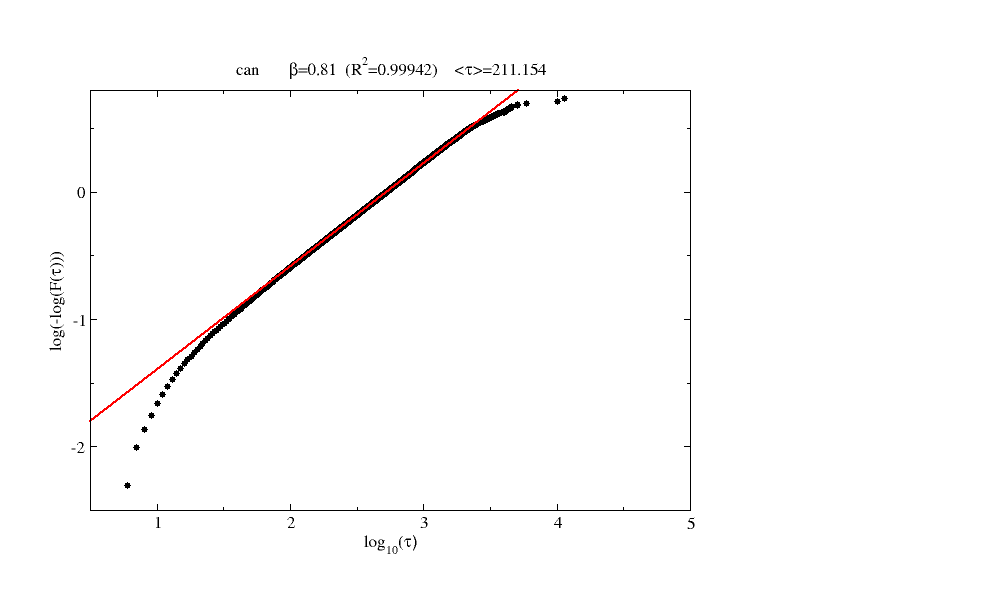

Supplement: Table S1 — Detailed information on the statistical analysis of all words that were studied (six databases). (31.88 MB TAR) [file pone.0007678.s002.tar › recurrence/comp/can.png]

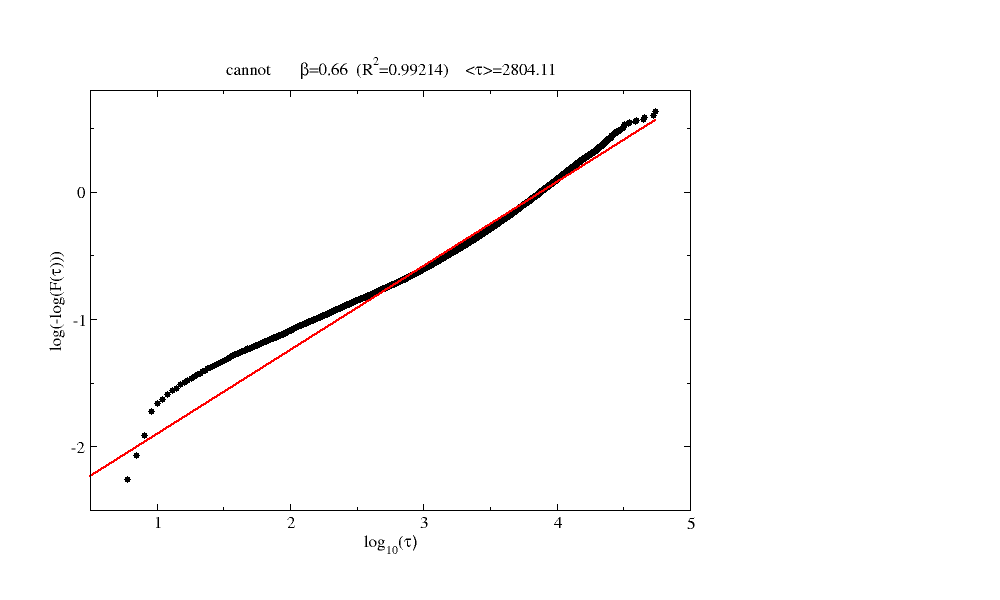

Supplement: Table S1 — Detailed information on the statistical analysis of all words that were studied (six databases). (31.88 MB TAR) [file pone.0007678.s002.tar › recurrence/comp/cannot.png]

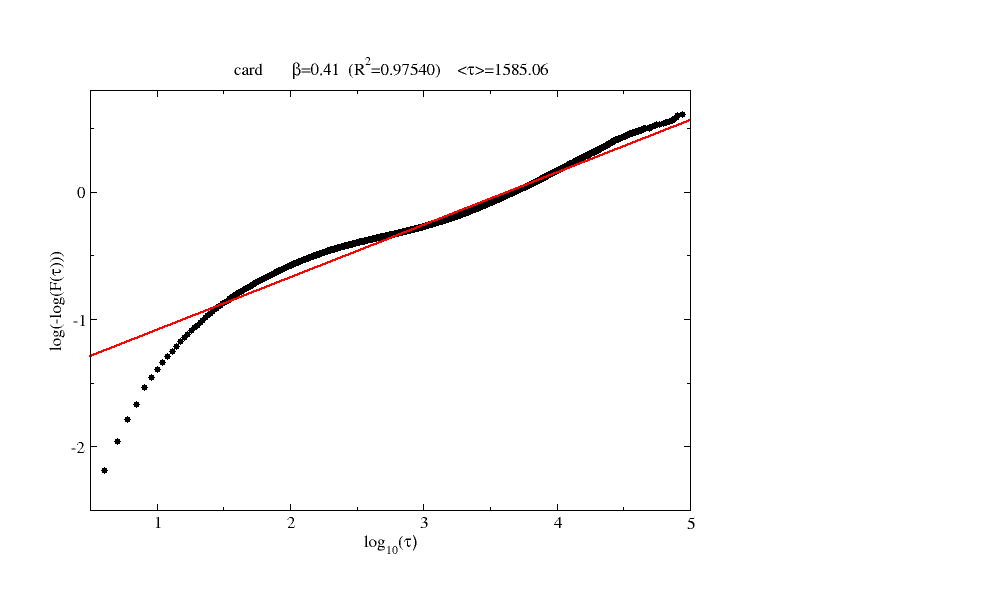

Supplement: Table S1 — Detailed information on the statistical analysis of all words that were studied (six databases). (31.88 MB TAR) [file pone.0007678.s002.tar › recurrence/comp/card.png]

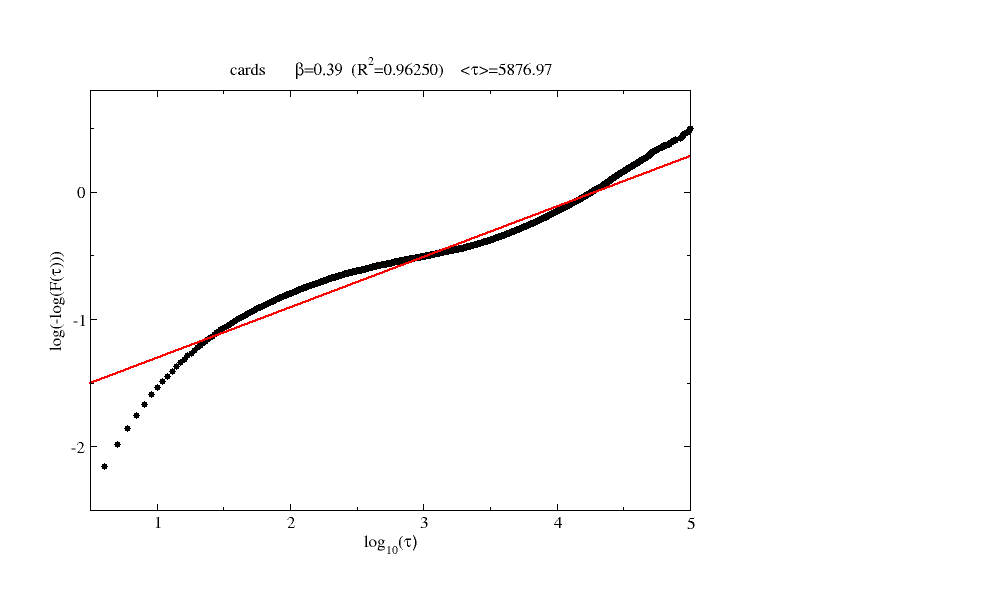

Supplement: Table S1 — Detailed information on the statistical analysis of all words that were studied (six databases). (31.88 MB TAR) [file pone.0007678.s002.tar › recurrence/comp/cards.png]

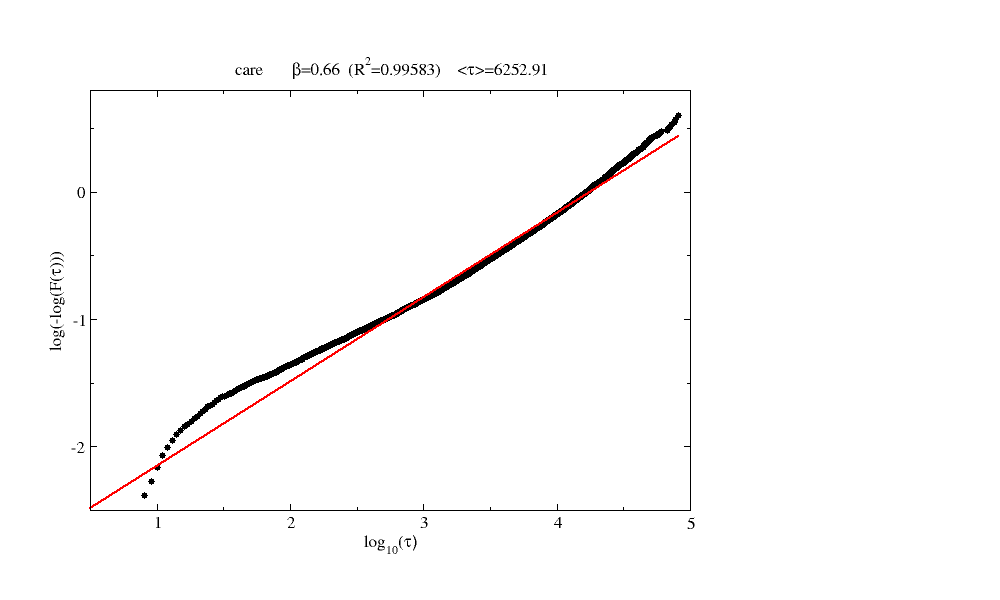

Supplement: Table S1 — Detailed information on the statistical analysis of all words that were studied (six databases). (31.88 MB TAR) [file pone.0007678.s002.tar › recurrence/comp/care.png]

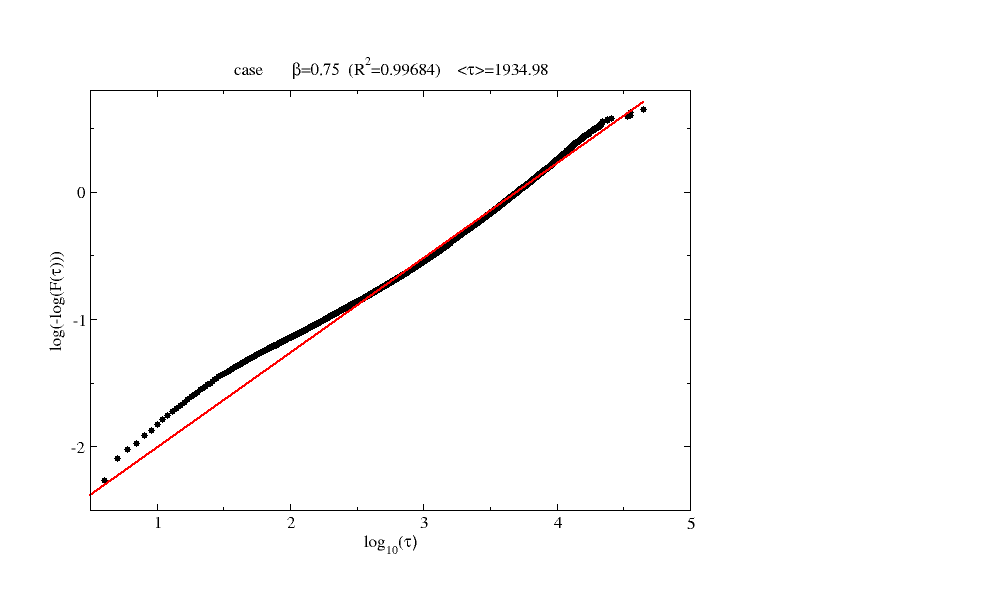

Supplement: Table S1 — Detailed information on the statistical analysis of all words that were studied (six databases). (31.88 MB TAR) [file pone.0007678.s002.tar › recurrence/comp/case.png]

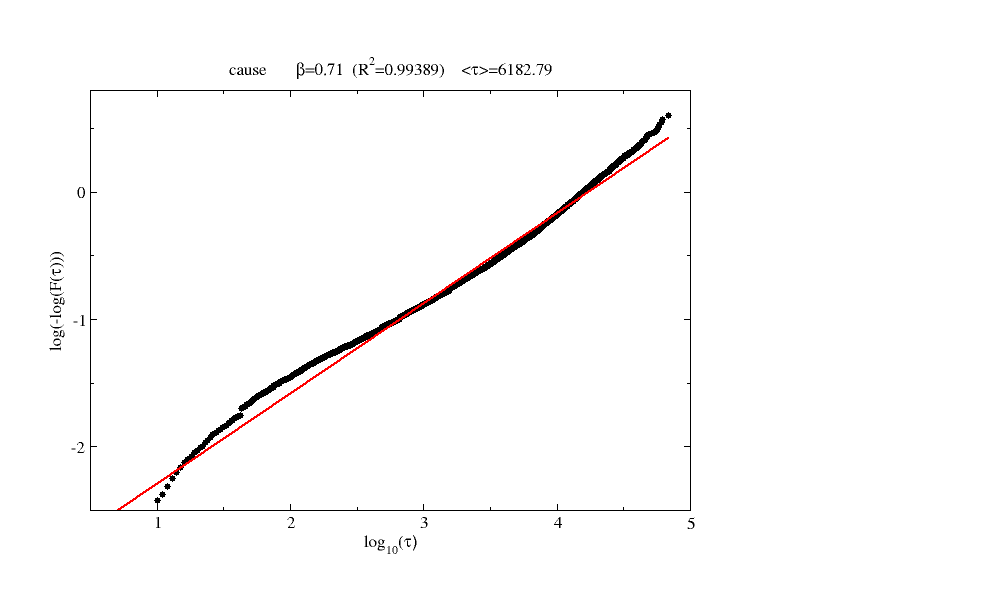

Supplement: Table S1 — Detailed information on the statistical analysis of all words that were studied (six databases). (31.88 MB TAR) [file pone.0007678.s002.tar › recurrence/comp/cause.png]

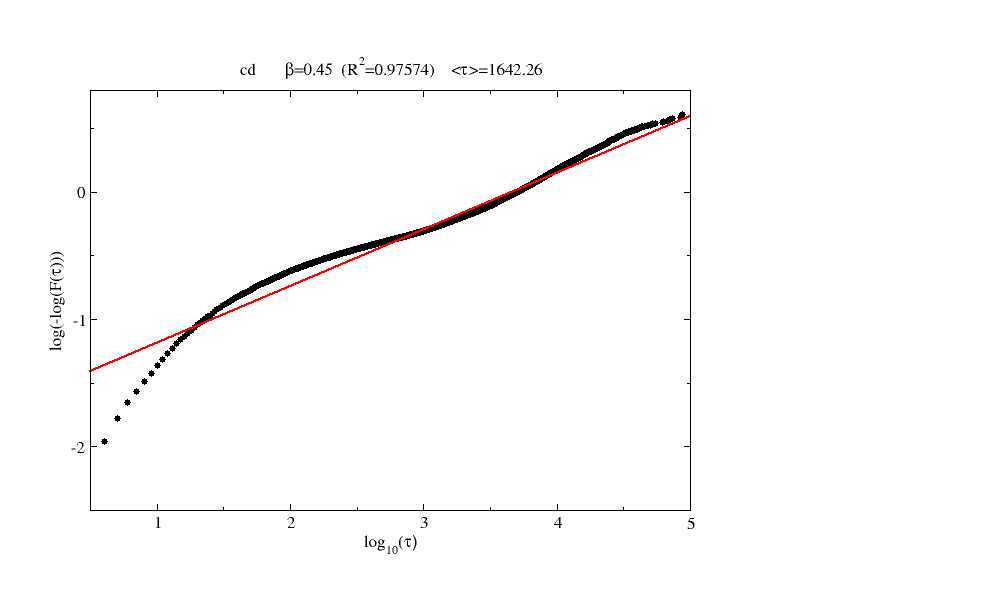

Supplement: Table S1 — Detailed information on the statistical analysis of all words that were studied (six databases). (31.88 MB TAR) [file pone.0007678.s002.tar › recurrence/comp/cd.png]

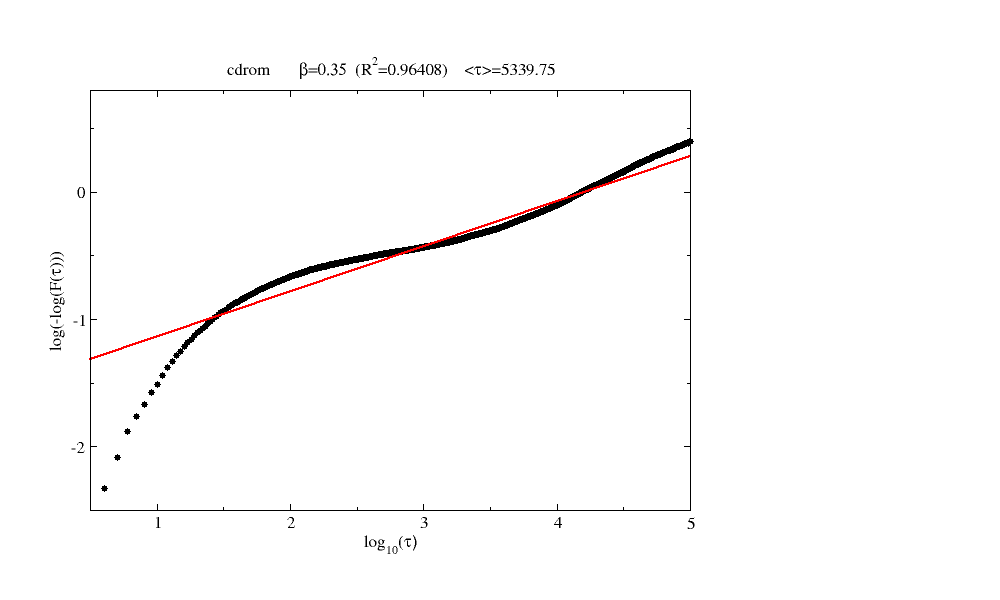

Supplement: Table S1 — Detailed information on the statistical analysis of all words that were studied (six databases). (31.88 MB TAR) [file pone.0007678.s002.tar › recurrence/comp/cdrom.png]

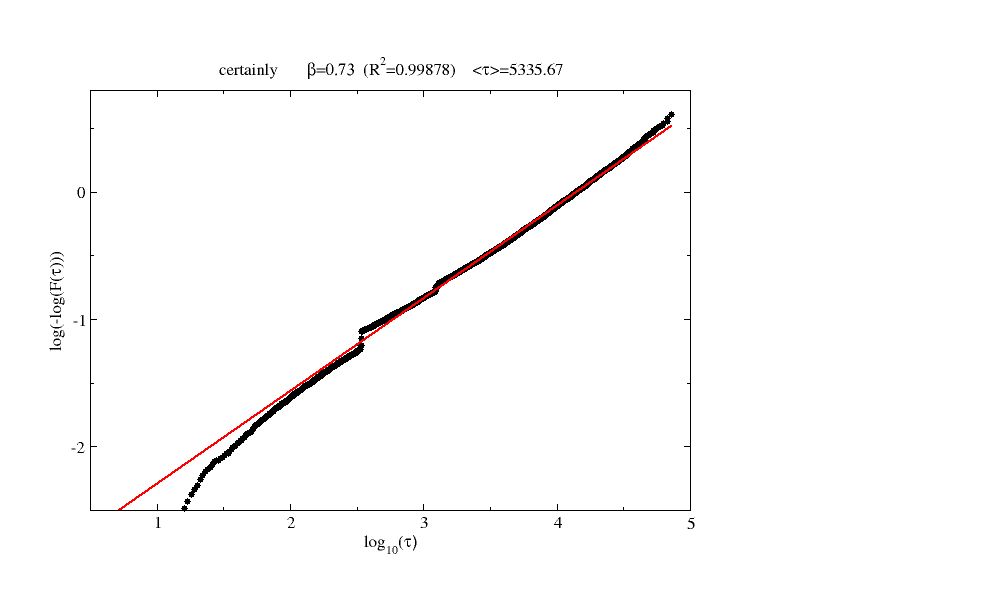

Supplement: Table S1 — Detailed information on the statistical analysis of all words that were studied (six databases). (31.88 MB TAR) [file pone.0007678.s002.tar › recurrence/comp/certainly.png]

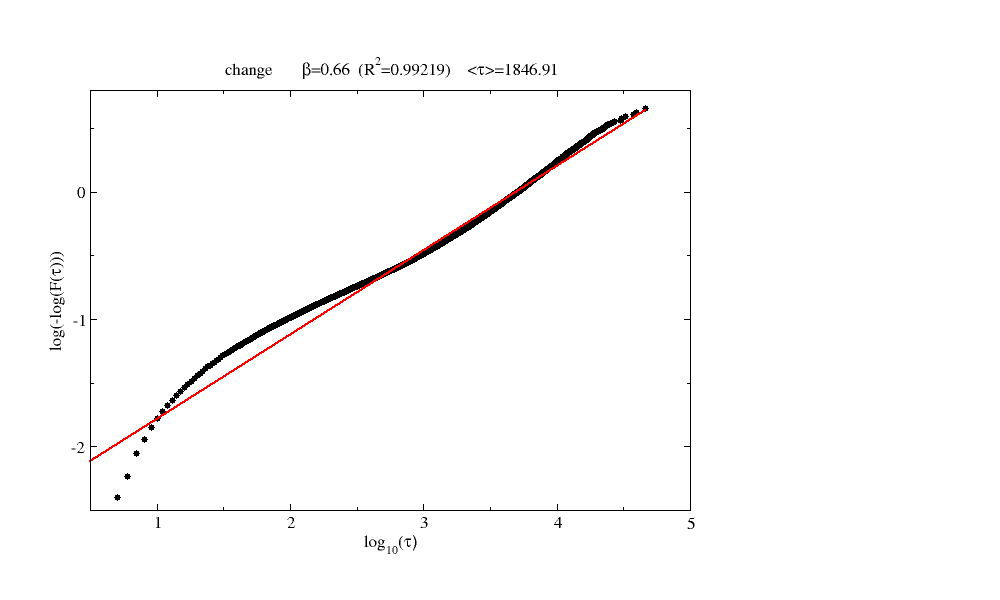

Supplement: Table S1 — Detailed information on the statistical analysis of all words that were studied (six databases). (31.88 MB TAR) [file pone.0007678.s002.tar › recurrence/comp/change.png]

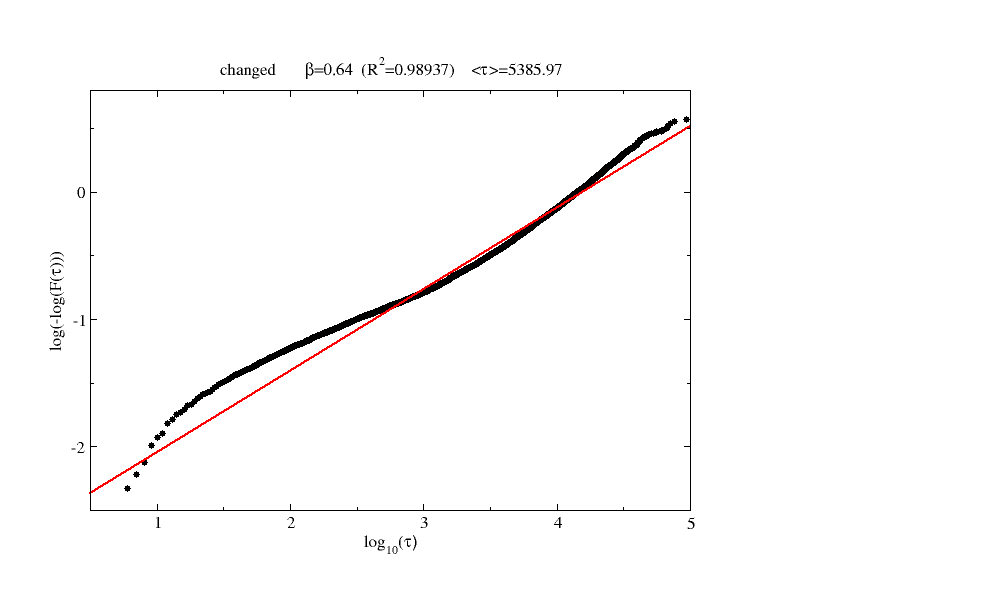

Supplement: Table S1 — Detailed information on the statistical analysis of all words that were studied (six databases). (31.88 MB TAR) [file pone.0007678.s002.tar › recurrence/comp/changed.png]

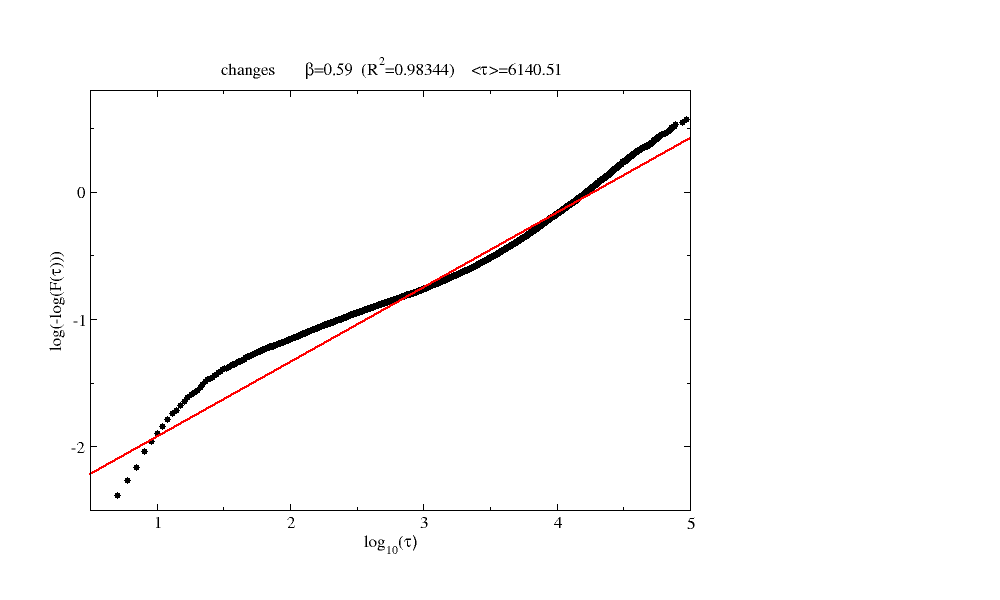

Supplement: Table S1 — Detailed information on the statistical analysis of all words that were studied (six databases). (31.88 MB TAR) [file pone.0007678.s002.tar › recurrence/comp/changes.png]

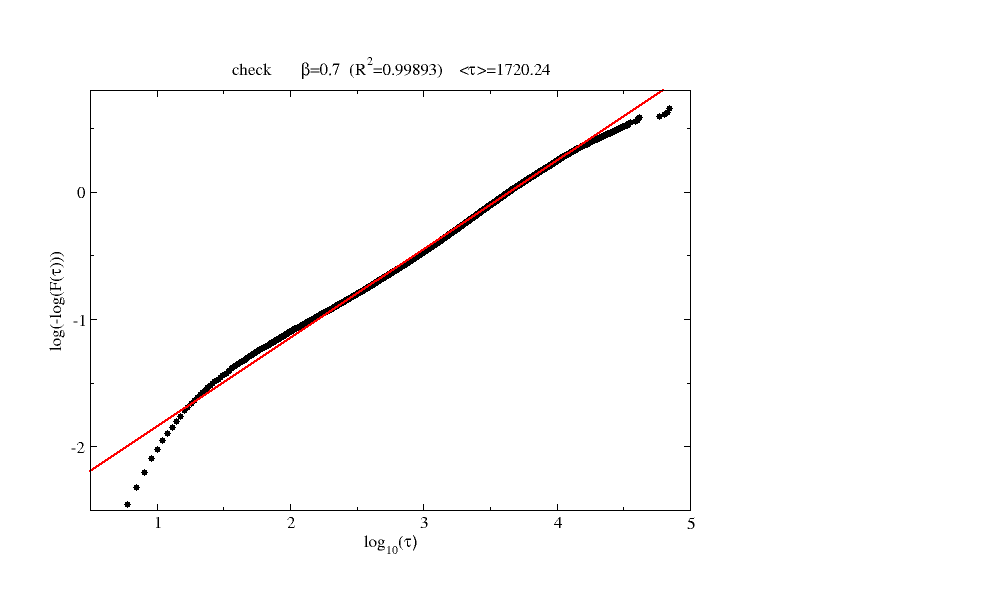

Supplement: Table S1 — Detailed information on the statistical analysis of all words that were studied (six databases). (31.88 MB TAR) [file pone.0007678.s002.tar › recurrence/comp/check.png]

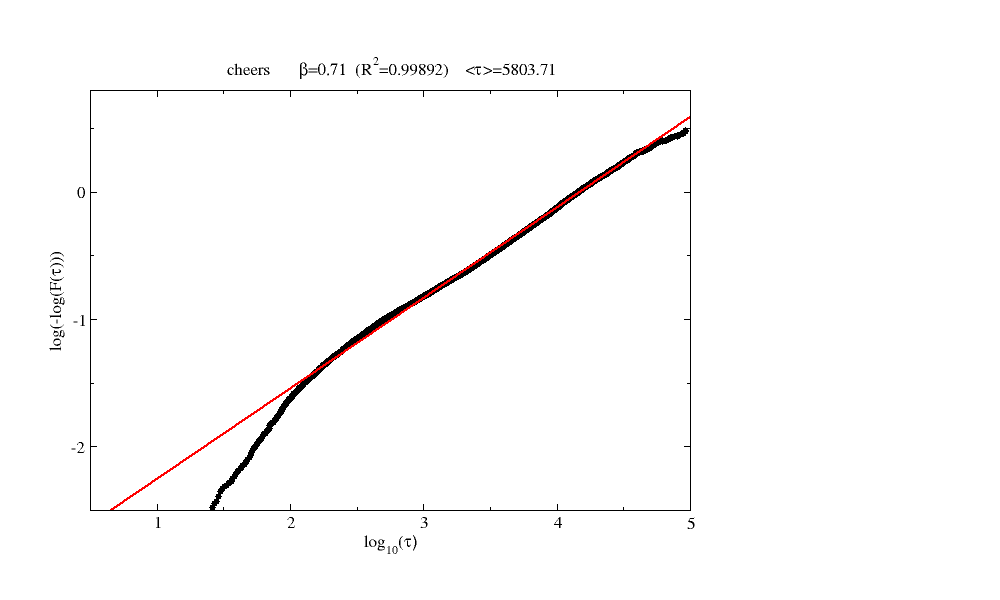

Supplement: Table S1 — Detailed information on the statistical analysis of all words that were studied (six databases). (31.88 MB TAR) [file pone.0007678.s002.tar › recurrence/comp/cheers.png]

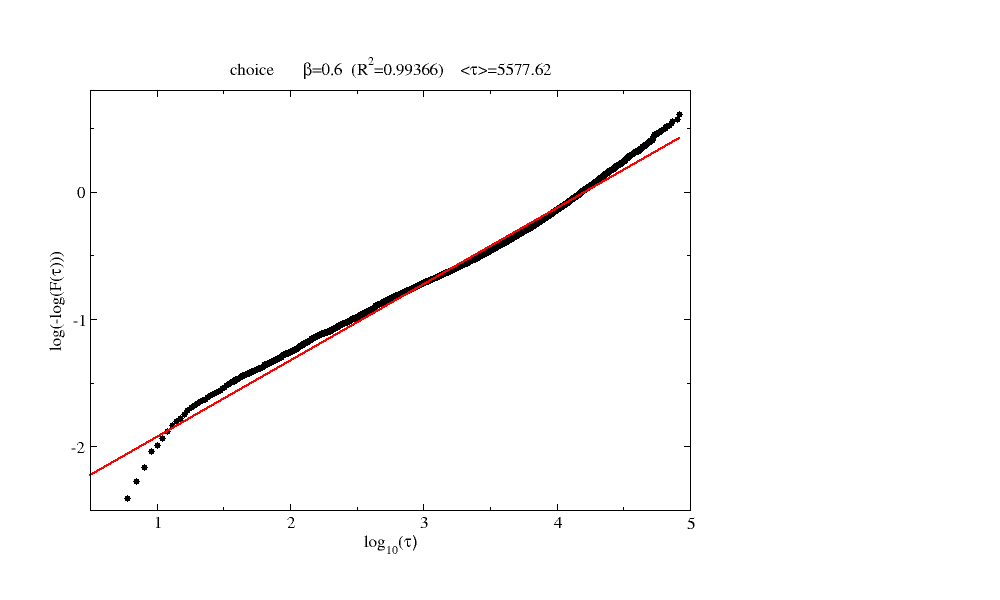

Supplement: Table S1 — Detailed information on the statistical analysis of all words that were studied (six databases). (31.88 MB TAR) [file pone.0007678.s002.tar › recurrence/comp/choice.png]

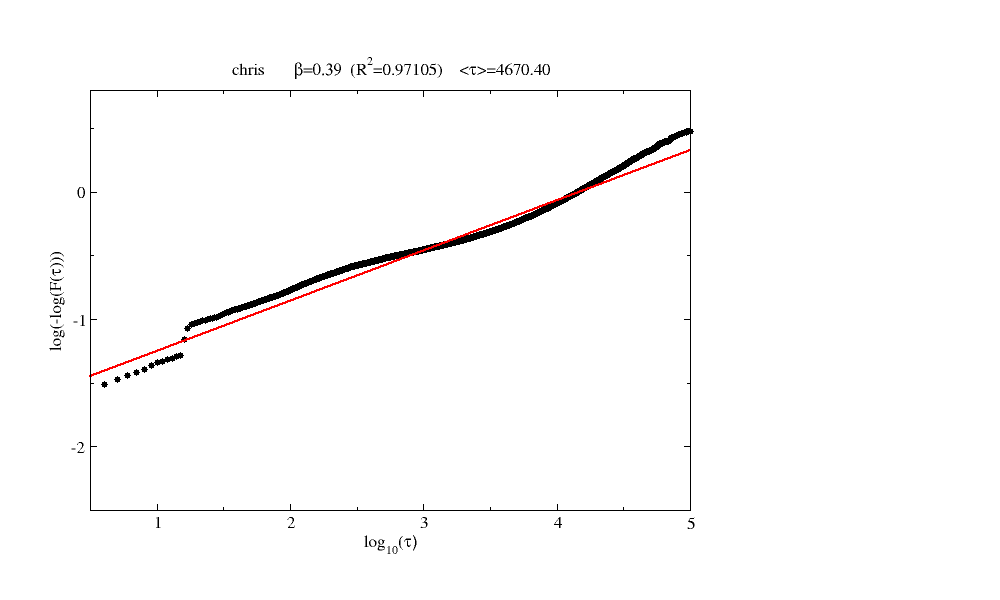

Supplement: Table S1 — Detailed information on the statistical analysis of all words that were studied (six databases). (31.88 MB TAR) [file pone.0007678.s002.tar › recurrence/comp/chris.png]

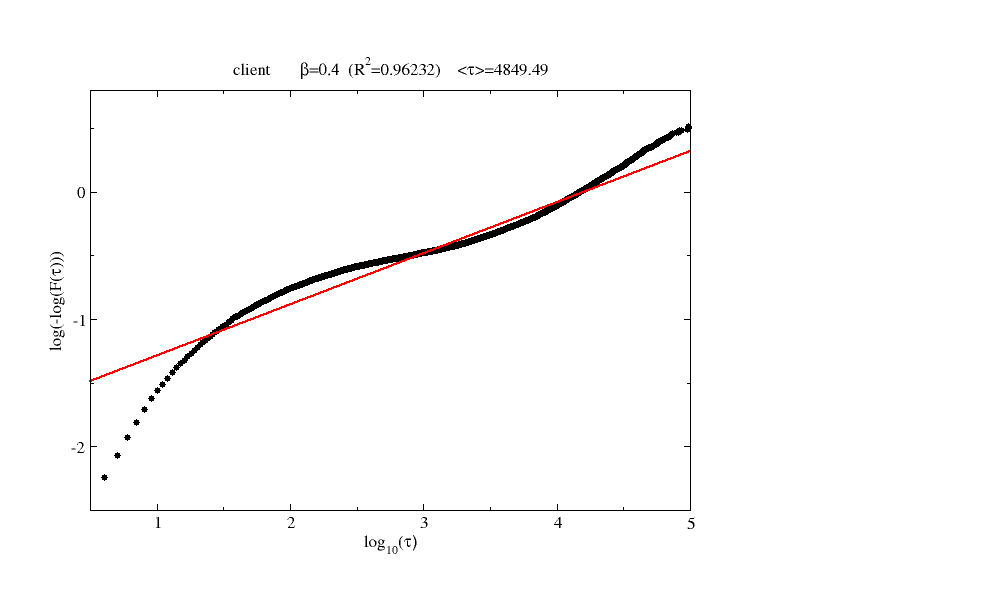

Supplement: Table S1 — Detailed information on the statistical analysis of all words that were studied (six databases). (31.88 MB TAR) [file pone.0007678.s002.tar › recurrence/comp/client.png]

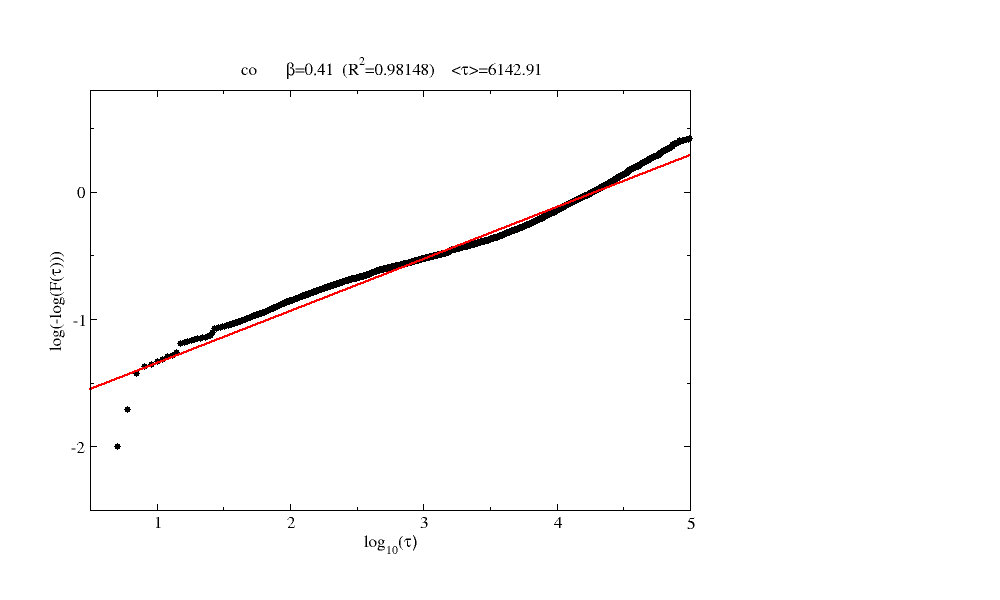

Supplement: Table S1 — Detailed information on the statistical analysis of all words that were studied (six databases). (31.88 MB TAR) [file pone.0007678.s002.tar › recurrence/comp/co.png]

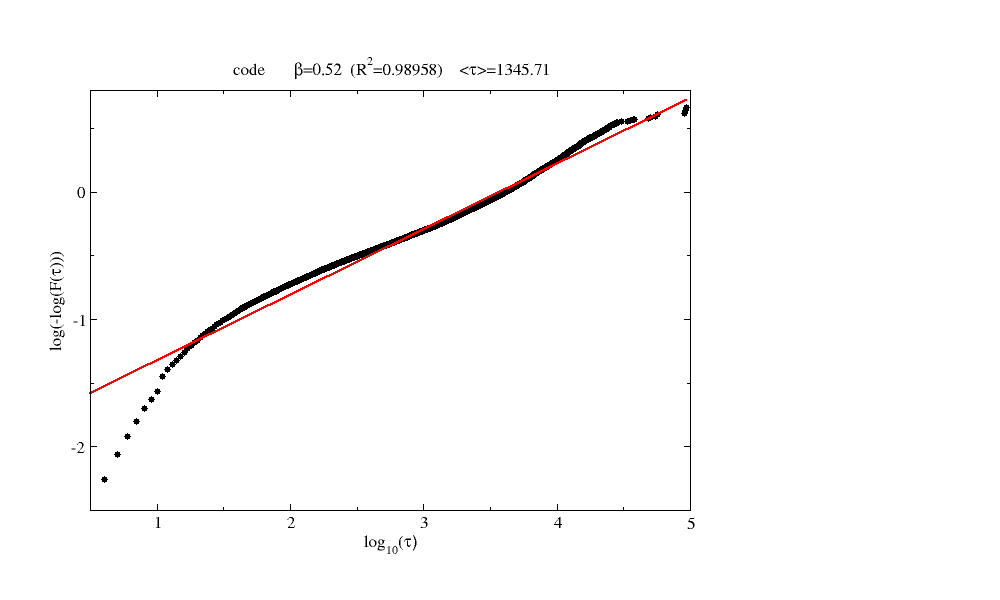

Supplement: Table S1 — Detailed information on the statistical analysis of all words that were studied (six databases). (31.88 MB TAR) [file pone.0007678.s002.tar › recurrence/comp/code.png]

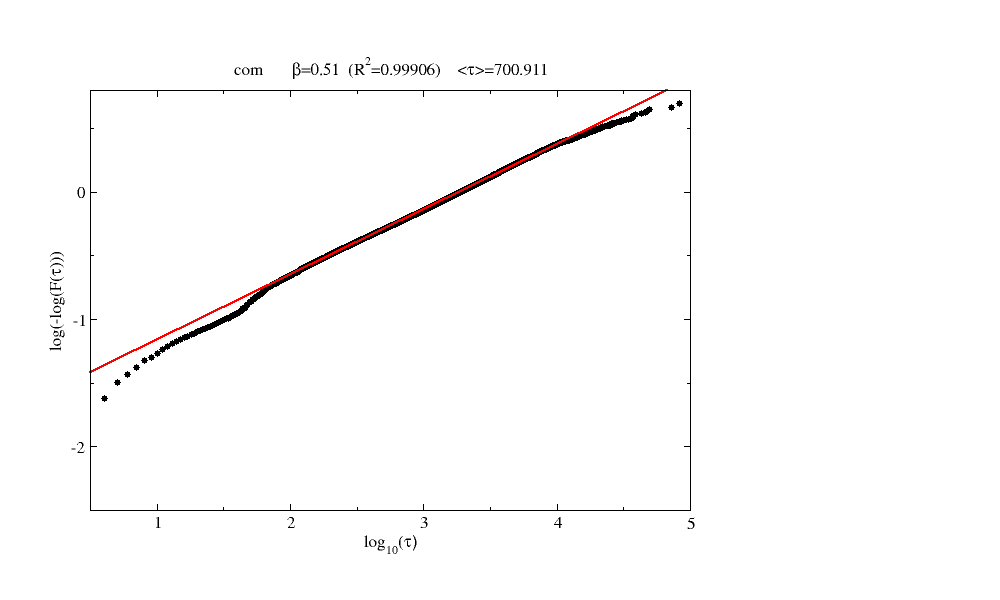

Supplement: Table S1 — Detailed information on the statistical analysis of all words that were studied (six databases). (31.88 MB TAR) [file pone.0007678.s002.tar › recurrence/comp/com.png]
