# Supplementary material for: Beyond Word Frequency: Bursts, Lulls, and Scaling in the Temporal Distributions of Words
Source: PLoS One. 2009 Nov 11;4(11):e7678. doi: 10.1371/journal.pone.0007678 (PMC2770836; doi:10.1371/journal.pone.0007678)
Supplement: Table S1 — Detailed information on the statistical analysis of all words that were studied (six databases). (31.88 MB TAR) [file pone.0007678.s002.tar › recurrence/comp/index.html]

Word < tau > | ------ | beta | ----- | R^2 | ----- || cache | 6283.54 |  | 0.289 |  | 0.9565 |  | | sounds | 6279.78 |  | 0.722 |  | 0.9944 |  | | sense | 6271.00 |  | 0.547 |  | 0.9824 |  | | usb | 6257.27 |  | 0.225 |  | 0.9466 |  | | care | 6252.91 |  | 0.663 |  | 0.9958 |  | | cs | 6243.60 |  | 0.471 |  | 0.9890 |  | | couldn't | 6241.74 |  | 0.709 |  | 0.9925 |  | | complete | 6239.26 |  | 0.657 |  | 0.9904 |  | | light | 6235.54 |  | 0.649 |  | 0.9969 |  | | average | 6222.58 |  | 0.501 |  | 0.9980 |  | | otherwise | 6204.15 |  | 0.782 |  | 0.9969 |  | | takes | 6202.32 |  | 0.648 |  | 0.9904 |  | | per | 6198.65 |  | 0.513 |  | 0.9789 |  | | save | 6193.15 |  | 0.585 |  | 0.9857 |  | | computers | 6185.23 |  | 0.523 |  | 0.9932 |  | | cause | 6182.79 |  | 0.709 |  | 0.9939 |  | | happens | 6170.05 |  | 0.715 |  | 0.9938 |  | | left | 6168.84 |  | 0.637 |  | 0.9881 |  | | editor | 6165.81 |  | 0.365 |  | 0.9792 |  | | google | 6155.55 |  | 0.459 |  | 0.9945 |  | | ac | 6148.92 |  | 0.444 |  | 0.9947 |  | | him | 6146.52 |  | 0.439 |  | 0.9845 |  | | controller | 6144.71 |  | 0.334 |  | 0.9587 |  | | co | 6142.91 |  | 0.410 |  | 0.9815 |  | | changes | 6140.51 |  | 0.587 |  | 0.9834 |  | | created | 6138.11 |  | 0.597 |  | 0.9859 |  | | during | 6116.61 |  | 0.657 |  | 0.9912 |  | | below | 6110.66 |  | 0.627 |  | 0.9925 |  | | mounted | 6103.54 |  | 0.431 |  | 0.9744 |  | | account | 6095.26 |  | 0.455 |  | 0.9737 |  | | hand | 6087.58 |  | 0.726 |  | 0.9972 |  | | move | 6082.28 |  | 0.579 |  | 0.9820 |  | | uk | 6071.12 |  | 0.413 |  | 0.9818 |  | | devices | 6069.36 |  | 0.426 |  | 0.9672 |  | | went | 6065.26 |  | 0.694 |  | 0.9941 |  | | services | 6055.91 |  | 0.486 |  | 0.9910 |  | | anybody | 6052.41 |  | 0.684 |  | 0.9961 |  | | within | 6037.29 |  | 0.674 |  | 0.9949 |  | | figure | 6022.26 |  | 0.739 |  | 0.9944 |  | | snip | 6010.74 |  | 0.561 |  | 0.9853 |  | | directly | 6006.72 |  | 0.710 |  | 0.9955 |  | | opinions | 5980.43 |  | 0.514 |  | 0.9915 |  | | kill | 5962.84 |  | 0.436 |  | 0.9782 |  | | helps | 5961.71 |  | 0.778 |  | 0.9992 |  | | specific | 5947.60 |  | 0.666 |  | 0.9908 |  | | intel | 5945.92 |  | 0.360 |  | 0.9698 |  | | somewhere | 5935.81 |  | 0.785 |  | 0.9978 |  | | stop | 5934.13 |  | 0.607 |  | 0.9874 |  | | trouble | 5931.89 |  | 0.713 |  | 0.9953 |  | | recently | 5907.91 |  | 0.780 |  | 0.9983 |  | | ls | 5907.36 |  | 0.341 |  | 0.9417 |  | | oh | 5891.30 |  | 0.723 |  | 0.9982 |  | | pci | 5891.30 |  | 0.272 |  | 0.9351 |  | | telnet | 5887.44 |  | 0.329 |  | 0.9495 |  | | vi | 5886.33 |  | 0.268 |  | 0.9526 |  | | build | 5885.78 |  | 0.518 |  | 0.9753 |  | | binary | 5885.23 |  | 0.454 |  | 0.9745 |  | | cards | 5876.98 |  | 0.395 |  | 0.9625 |  | | perl | 5876.98 |  | 0.323 |  | 0.9493 |  | | they're | 5828.48 |  | 0.640 |  | 0.9927 |  | | matt | 5822.00 |  | 0.536 |  | 0.9978 |  | | gives | 5809.62 |  | 0.736 |  | 0.9946 |  | | cheers | 5803.72 |  | 0.711 |  | 0.9989 |  | | performance | 5802.65 |  | 0.420 |  | 0.9846 |  | | saying | 5801.04 |  | 0.676 |  | 0.9978 |  | | scripts | 5799.97 |  | 0.457 |  | 0.9772 |  | | particular | 5799.44 |  | 0.710 |  | 0.9963 |  | | mine | 5789.82 |  | 0.630 |  | 0.9910 |  | | office | 5788.75 |  | 0.426 |  | 0.9768 |  | | heard | 5788.75 |  | 0.695 |  | 0.9958 |  | | year | 5787.15 |  | 0.543 |  | 0.9896 |  | | btw | 5778.11 |  | 0.826 |  | 0.9993 |  | | writing | 5761.69 |  | 0.580 |  | 0.9908 |  | | easier | 5736.97 |  | 0.660 |  | 0.9959 |  | | details | 5735.93 |  | 0.539 |  | 0.9838 |  | | prompt | 5715.58 |  | 0.484 |  | 0.9815 |  | | sometimes | 5710.39 |  | 0.634 |  | 0.9849 |  | | isp | 5709.87 |  | 0.351 |  | 0.9682 |  | | mind | 5703.65 |  | 0.727 |  | 0.9978 |  | | function | 5701.07 |  | 0.393 |  | 0.9378 |  | | product | 5681.49 |  | 0.435 |  | 0.9901 |  | | bug | 5672.77 |  | 0.452 |  | 0.9691 |  | | normal | 5671.74 |  | 0.627 |  | 0.9881 |  | | mandrake | 5667.65 |  | 0.423 |  | 0.9894 |  | | gz | 5666.63 |  | 0.327 |  | 0.9549 |  | | ones | 5666.63 |  | 0.676 |  | 0.9895 |  | | terminal | 5666.12 |  | 0.384 |  | 0.9561 |  | | faster | 5656.94 |  | 0.526 |  | 0.9930 |  | | level | 5654.40 |  | 0.520 |  | 0.9723 |  | | ideas | 5645.76 |  | 0.704 |  | 0.9986 |  | | supported | 5598.99 |  | 0.525 |  | 0.9787 |  | | yourself | 5592.52 |  | 0.692 |  | 0.9973 |  | | choice | 5577.63 |  | 0.599 |  | 0.9937 |  | | main | 5540.26 |  | 0.572 |  | 0.9827 |  | | steve | 5535.87 |  | 0.419 |  | 0.9866 |  | | expect | 5535.38 |  | 0.587 |  | 0.9913 |  | | gnome | 5533.92 |  | 0.324 |  | 0.9689 |  | | ibm | 5531.00 |  | 0.368 |  | 0.9779 |  | | given | 5523.22 |  | 0.726 |  | 0.9956 |  | | came | 5522.25 |  | 0.713 |  | 0.9948 |  | | java | 5516.44 |  | 0.248 |  | 0.9385 |  | | important | 5513.53 |  | 0.694 |  | 0.9958 |  | | difference | 5508.22 |  | 0.641 |  | 0.9934 |  | | currently | 5490.42 |  | 0.721 |  | 0.9970 |  | | solaris | 5479.88 |  | 0.311 |  | 0.9603 |  | | ps | 5474.16 |  | 0.459 |  | 0.9698 |  | | libraries | 5471.77 |  | 0.400 |  | 0.9677 |  | | display | 5464.16 |  | 0.426 |  | 0.9628 |  | | provide | 5433.46 |  | 0.654 |  | 0.9955 |  | | you've | 5431.11 |  | 0.703 |  | 0.9940 |  | | domain | 5428.30 |  | 0.388 |  | 0.9682 |  | | changed | 5385.97 |  | 0.642 |  | 0.9894 |  | | newsgroup | 5357.51 |  | 0.549 |  | 0.9912 |  | | cdrom | 5339.76 |  | 0.355 |  | 0.9641 |  | | certainly | 5335.68 |  | 0.730 |  | 0.9988 |  | | bill | 5325.73 |  | 0.465 |  | 0.9875 |  | | commands | 5315.82 |  | 0.518 |  | 0.9866 |  | | myself | 5297.01 |  | 0.765 |  | 0.9978 |  | | switch | 5285.43 |  | 0.564 |  | 0.9847 |  | | dave | 5268.15 |  | 0.483 |  | 0.9876 |  | | console | 5247.48 |  | 0.418 |  | 0.9652 |  | | host | 5230.45 |  | 0.384 |  | 0.9488 |  | | environment | 5221.76 |  | 0.521 |  | 0.9820 |  | | apps | 5188.14 |  | 0.448 |  | 0.9865 |  | | couple | 5165.96 |  | 0.769 |  | 0.9967 |  | | edit | 5160.45 |  | 0.575 |  | 0.9906 |  | | phone | 5159.60 |  | 0.507 |  | 0.9965 |  | | appreciated | 5158.76 |  | 0.747 |  | 0.9979 |  | | serial | 5151.99 |  | 0.325 |  | 0.9537 |  | | everyone | 5151.15 |  | 0.651 |  | 0.9962 |  | | goes | 5150.72 |  | 0.736 |  | 0.9947 |  | | newsgroups | 5136.83 |  | 0.420 |  | 0.9813 |  | | book | 5127.19 |  | 0.420 |  | 0.9643 |  | | three | 5123.43 |  | 0.641 |  | 0.9903 |  | | various | 5108.86 |  | 0.673 |  | 0.9910 |  | | emacs | 5093.54 |  | 0.293 |  | 0.9682 |  | | disks | 5087.36 |  | 0.449 |  | 0.9798 |  | | money | 5082.42 |  | 0.467 |  | 0.9951 |  | | sort | 5071.35 |  | 0.678 |  | 0.9925 |  | | including | 5034.38 |  | 0.766 |  | 0.9980 |  | | speed | 4995.97 |  | 0.442 |  | 0.9748 |  | | later | 4992.79 |  | 0.729 |  | 0.9967 |  | | fast | 4974.22 |  | 0.555 |  | 0.9918 |  | | errors | 4973.44 |  | 0.484 |  | 0.9749 |  | | tape | 4936.72 |  | 0.237 |  | 0.9119 |  | | website | 4891.76 |  | 0.244 |  | 0.8855 |  | | job | 4876.20 |  | 0.569 |  | 0.9852 |  | | gets | 4876.20 |  | 0.750 |  | 0.9959 |  | | useful | 4854.74 |  | 0.689 |  | 0.9958 |  | | client | 4849.49 |  | 0.401 |  | 0.9623 |  | | gui | 4841.28 |  | 0.388 |  | 0.9888 |  | | commercial | 4834.20 |  | 0.447 |  | 0.9922 |  | | de | 4827.89 |  | 0.392 |  | 0.9637 |  | | date | 4825.29 |  | 0.344 |  | 0.9377 |  | | include | 4819.74 |  | 0.599 |  | 0.9900 |  | | setting | 4819.37 |  | 0.635 |  | 0.9924 |  | | servers | 4818.64 |  | 0.514 |  | 0.9900 |  | | pgp | 4807.58 |  | 0.475 |  | 0.9990 |  | | experience | 4805.74 |  | 0.665 |  | 0.9930 |  | | price | 4803.54 |  | 0.285 |  | 0.9657 |  | | reboot | 4796.57 |  | 0.516 |  | 0.9850 |  | | backup | 4789.26 |  | 0.342 |  | 0.9497 |  | | fdisk | 4771.45 |  | 0.279 |  | 0.9469 |  | | thank | 4770.36 |  | 0.668 |  | 0.9950 |  | | service | 4765.30 |  | 0.452 |  | 0.9808 |  | | configure | 4753.41 |  | 0.527 |  | 0.9821 |  | | wouldn't | 4753.41 |  | 0.755 |  | 0.9987 |  | | although | 4735.50 |  | 0.786 |  | 0.9978 |  | | tools | 4733.72 |  | 0.510 |  | 0.9849 |  | | paul | 4720.21 |  | 0.384 |  | 0.9831 |  | | issue | 4709.95 |  | 0.626 |  | 0.9926 |  | | installing | 4708.54 |  | 0.626 |  | 0.9957 |  | | often | 4700.44 |  | 0.691 |  | 0.9976 |  | | general | 4694.12 |  | 0.654 |  | 0.9957 |  | | latest | 4692.72 |  | 0.594 |  | 0.9908 |  | | library | 4692.02 |  | 0.384 |  | 0.9617 |  | | development | 4688.87 |  | 0.513 |  | 0.9927 |  | | play | 4685.02 |  | 0.524 |  | 0.9835 |  | | mark | 4680.84 |  | 0.427 |  | 0.9788 |  | | itself | 4674.92 |  | 0.774 |  | 0.9973 |  | | chris | 4670.40 |  | 0.394 |  | 0.9711 |  | | what's | 4670.40 |  | 0.745 |  | 0.9973 |  | | mb | 4616.21 |  | 0.361 |  | 0.9515 |  | | show | 4602.01 |  | 0.623 |  | 0.9943 |  | | usenet | 4600.33 |  | 0.471 |  | 0.9917 |  | | jordan | 4592.60 |  | 0.104 |  | 0.4107 |  | | bash | 4577.55 |  | 0.330 |  | 0.9439 |  | | written | 4572.55 |  | 0.647 |  | 0.9934 |  | | learn | 4569.89 |  | 0.506 |  | 0.9943 |  | | large | 4569.56 |  | 0.620 |  | 0.9923 |  | | compiled | 4566.57 |  | 0.540 |  | 0.9911 |  | | manager | 4555.32 |  | 0.444 |  | 0.9772 |  | | groups | 4550.70 |  | 0.353 |  | 0.9728 |  | | luck | 4542.81 |  | 0.779 |  | 0.9995 |  | | image | 4518.64 |  | 0.380 |  | 0.9503 |  | | making | 4517.34 |  | 0.740 |  | 0.9981 |  | | likely | 4511.83 |  | 0.679 |  | 0.9963 |  | | security | 4500.84 |  | 0.414 |  | 0.9791 |  | | robert | 4498.59 |  | 0.464 |  | 0.9859 |  | | cpu | 4494.73 |  | 0.349 |  | 0.9618 |  | | ask | 4492.16 |  | 0.744 |  | 0.9974 |  | | advance | 4484.47 |  | 0.763 |  | 0.9975 |  | | connect | 4483.51 |  | 0.448 |  | 0.9759 |  | | questions | 4478.40 |  | 0.557 |  | 0.9907 |  | | spam | 4459.65 |  | 0.417 |  | 0.9961 |  | | almost | 4455.22 |  | 0.780 |  | 0.9990 |  | | away | 4452.70 |  | 0.726 |  | 0.9978 |  | | desktop | 4443.25 |  | 0.435 |  | 0.9855 |  | | password | 4428.54 |  | 0.299 |  | 0.9447 |  | | top | 4424.80 |  | 0.477 |  | 0.9744 |  | | documentation | 4369.13 |  | 0.508 |  | 0.9866 |  | | modules | 4362.46 |  | 0.322 |  | 0.9459 |  | | matter | 4338.07 |  | 0.770 |  | 0.9989 |  | | ago | 4286.30 |  | 0.782 |  | 0.9979 |  | | video | 4283.96 |  | 0.392 |  | 0.9617 |  | | except | 4277.84 |  | 0.809 |  | 0.9993 |  | | application | 4274.35 |  | 0.512 |  | 0.9852 |  | | company | 4266.51 |  | 0.427 |  | 0.9885 |  | | control | 4259.57 |  | 0.537 |  | 0.9836 |  | | power | 4245.48 |  | 0.434 |  | 0.9731 |  | | solution | 4236.32 |  | 0.645 |  | 0.9918 |  | | whether | 4229.76 |  | 0.651 |  | 0.9911 |  | | id | 4225.50 |  | 0.398 |  | 0.9864 |  | | public | 4224.08 |  | 0.560 |  | 0.9955 |  | | needed | 4216.71 |  | 0.672 |  | 0.9898 |  | | posting | 4212.19 |  | 0.472 |  | 0.9838 |  | | path | 4206.55 |  | 0.327 |  | 0.9186 |  | | allow | 4201.21 |  | 0.642 |  | 0.9882 |  | | edu | 4172.48 |  | 0.438 |  | 0.9973 |  | | started | 4171.93 |  | 0.703 |  | 0.9941 |  | | release | 4161.71 |  | 0.528 |  | 0.9889 |  | | registered | 4146.06 |  | 0.381 |  | 0.9967 |  | | echo | 4138.96 |  | 0.313 |  | 0.9743 |  | | drivers | 4111.35 |  | 0.455 |  | 0.9754 |  | | subject | 4105.98 |  | 0.398 |  | 0.9612 |  | | sorry | 4084.63 |  | 0.774 |  | 0.9982 |  | | sun | 4070.87 |  | 0.466 |  | 0.9828 |  | | upgrade | 4062.19 |  | 0.490 |  | 0.9843 |  | | single | 4031.70 |  | 0.610 |  | 0.9904 |  | | versions | 4031.96 |  | 0.623 |  | 0.9919 |  | | printer | 4022.67 |  | 0.253 |  | 0.9090 |  | | pages | 4018.56 |  | 0.484 |  | 0.9803 |  | | sent | 4009.84 |  | 0.459 |  | 0.9947 |  | | ide | 4001.42 |  | 0.335 |  | 0.9516 |  | | rh | 3999.13 |  | 0.445 |  | 0.9948 |  | | small | 3997.60 |  | 0.685 |  | 0.9940 |  | | whole | 3997.09 |  | 0.760 |  | 0.9975 |  | | operating | 3976.11 |  | 0.523 |  | 0.9953 |  | | packages | 3966.57 |  | 0.426 |  | 0.9715 |  | | order | 3964.32 |  | 0.648 |  | 0.9906 |  | | applications | 3941.20 |  | 0.494 |  | 0.9910 |  | | worked | 3936.26 |  | 0.731 |  | 0.9977 |  | | original | 3935.77 |  | 0.632 |  | 0.9931 |  | | full | 3934.54 |  | 0.678 |  | 0.9933 |  | | options | 3921.77 |  | 0.563 |  | 0.9858 |  | | based | 3911.52 |  | 0.652 |  | 0.9916 |  | | mouse | 3903.02 |  | 0.307 |  | 0.9442 |  | | interface | 3890.22 |  | 0.502 |  | 0.9829 |  | | call | 3889.02 |  | 0.582 |  | 0.9845 |  | | thought | 3874.39 |  | 0.786 |  | 0.9976 |  | | runs | 3859.64 |  | 0.664 |  | 0.9940 |  | | hello | 3852.78 |  | 0.688 |  | 0.9992 |  | | bios | 3848.54 |  | 0.327 |  | 0.9533 |  | | reply | 3830.71 |  | 0.618 |  | 0.9961 |  | | output | 3829.54 |  | 0.501 |  | 0.9824 |  | | ppp | 3822.56 |  | 0.302 |  | 0.9631 |  | | link | 3822.32 |  | 0.495 |  | 0.9742 |  | | module | 3814.20 |  | 0.351 |  | 0.9515 |  | | looks | 3801.75 |  | 0.754 |  | 0.9976 |  | | fax | 3790.51 |  | 0.544 |  | 0.9971 |  | | university | 3790.28 |  | 0.587 |  | 0.9968 |  | | current | 3789.60 |  | 0.643 |  | 0.9894 |  | | kind | 3755.18 |  | 0.769 |  | 0.9978 |  | | times | 3749.36 |  | 0.698 |  | 0.9933 |  | | print | 3741.99 |  | 0.352 |  | 0.9390 |  | | posted | 3735.54 |  | 0.635 |  | 0.9980 |  | | similar | 3715.23 |  | 0.793 |  | 0.9977 |  | | true | 3685.17 |  | 0.639 |  | 0.9969 |  | | reading | 3681.29 |  | 0.627 |  | 0.9924 |  | | nike | 3675.26 |  | 0.148 |  | 0.2940 |  | | simply | 3672.90 |  | 0.767 |  | 0.9991 |  | | gcc | 3665.19 |  | 0.309 |  | 0.9653 |  | | drives | 3652.20 |  | 0.398 |  | 0.9668 |  | | next | 3645.63 |  | 0.727 |  | 0.9944 |  | | exactly | 3642.67 |  | 0.802 |  | 0.9990 |  | | login | 3636.14 |  | 0.380 |  | 0.9625 |  | | usually | 3635.51 |  | 0.739 |  | 0.9979 |  | | understand | 3632.99 |  | 0.720 |  | 0.9975 |  | | fix | 3632.36 |  | 0.636 |  | 0.9931 |  | | ever | 3622.52 |  | 0.751 |  | 0.9992 |  | | format | 3618.55 |  | 0.482 |  | 0.9820 |  | | seen | 3613.98 |  | 0.743 |  | 0.9974 |  | | needs | 3609.00 |  | 0.694 |  | 0.9946 |  | | ms | 3602.17 |  | 0.367 |  | 0.9854 |  | | guess | 3600.52 |  | 0.791 |  | 0.9982 |  | | lines | 3595.58 |  | 0.512 |  | 0.9807 |  | | day | 3588.60 |  | 0.653 |  | 0.9956 |  | | remember | 3587.98 |  | 0.781 |  | 0.9974 |  | | word | 3581.85 |  | 0.424 |  | 0.9808 |  | | big | 3556.51 |  | 0.696 |  | 0.9954 |  | | unless | 3548.28 |  | 0.768 |  | 0.9981 |  | | download | 3520.06 |  | 0.542 |  | 0.9898 |  | | test | 3519.08 |  | 0.453 |  | 0.9555 |  | | messages | 3500.46 |  | 0.535 |  | 0.9920 |  | | screen | 3491.71 |  | 0.466 |  | 0.9704 |  | | connection | 3485.13 |  | 0.477 |  | 0.9804 |  | | size | 3483.39 |  | 0.443 |  | 0.9634 |  | | log | 3482.23 |  | 0.453 |  | 0.9767 |  | | correct | 3477.80 |  | 0.716 |  | 0.9959 |  | | perhaps | 3476.26 |  | 0.768 |  | 0.9979 |  | | local | 3473.38 |  | 0.523 |  | 0.9815 |  | | gnu | 3463.24 |  | 0.426 |  | 0.9819 |  | | window | 3441.62 |  | 0.428 |  | 0.9659 |  | | configuration | 3440.11 |  | 0.600 |  | 0.9949 |  | | second | 3430.72 |  | 0.640 |  | 0.9871 |  | | shoes | 3404.15 |  | 0.082 |  | 0.2141 |  | | makes | 3403.23 |  | 0.791 |  | 0.9994 |  | | others | 3402.31 |  | 0.761 |  | 0.9988 |  | | whatever | 3397.71 |  | 0.793 |  | 0.9984 |  | | simple | 3397.16 |  | 0.707 |  | 0.9960 |  | | suse | 3385.45 |  | 0.394 |  | 0.9848 |  | | comes | 3379.99 |  | 0.798 |  | 0.9985 |  | | kde | 3378.90 |  | 0.349 |  | 0.9702 |  | | until | 3364.97 |  | 0.772 |  | 0.9980 |  | | haven't | 3355.81 |  | 0.813 |  | 0.9992 |  | | filesystem | 3329.85 |  | 0.408 |  | 0.9713 |  | | machines | 3325.98 |  | 0.544 |  | 0.9875 |  | | note | 3324.04 |  | 0.743 |  | 0.9975 |  | | deja | 3295.46 |  | 0.269 |  | 0.9697 |  | | compile | 3277.76 |  | 0.514 |  | 0.9923 |  | | days | 3260.75 |  | 0.670 |  | 0.9979 |  | | says | 3237.41 |  | 0.674 |  | 0.9936 |  | | standard | 3225.94 |  | 0.622 |  | 0.9911 |  | | conf | 3220.49 |  | 0.410 |  | 0.9794 |  | | anyway | 3213.41 |  | 0.844 |  | 0.9992 |  | | you'll | 3192.35 |  | 0.719 |  | 0.9965 |  | | means | 3190.73 |  | 0.726 |  | 0.9973 |  | | easy | 3190.09 |  | 0.688 |  | 0.9963 |  | | place | 3186.21 |  | 0.738 |  | 0.9975 |  | | fact | 3178.95 |  | 0.726 |  | 0.9998 |  | | uses | 3170.78 |  | 0.726 |  | 0.9950 |  | | peter | 3162.16 |  | 0.426 |  | 0.9940 |  | | keep | 3156.13 |  | 0.761 |  | 0.9978 |  | | believe | 3155.34 |  | 0.747 |  | 0.9980 |  | | hope | 3132.85 |  | 0.797 |  | 0.9999 |  | | i'll | 3116.23 |  | 0.740 |  | 0.9973 |  | | ip | 3105.91 |  | 0.315 |  | 0.9502 |  | | several | 3088.97 |  | 0.776 |  | 0.9987 |  | | regards | 3078.98 |  | 0.721 |  | 0.9995 |  | | search | 3053.11 |  | 0.522 |  | 0.9957 |  | | partitions | 3049.55 |  | 0.347 |  | 0.9684 |  | | world | 3037.03 |  | 0.609 |  | 0.9976 |  | | years | 3034.83 |  | 0.613 |  | 0.9993 |  | | less | 3033.21 |  | 0.691 |  | 0.9970 |  | | rather | 3020.68 |  | 0.804 |  | 0.9989 |  | | let | 3013.15 |  | 0.796 |  | 0.9976 |  | | swap | 3012.57 |  | 0.308 |  | 0.9480 |  | | misc | 2996.63 |  | 0.520 |  | 0.9969 |  | | sound | 2990.36 |  | 0.374 |  | 0.9388 |  | | david | 2981.98 |  | 0.489 |  | 0.9938 |  | | buy | 2980.43 |  | 0.526 |  | 0.9981 |  | | reason | 2955.62 |  | 0.763 |  | 0.9984 |  | | great | 2952.57 |  | 0.742 |  | 0.9987 |  | | pretty | 2930.27 |  | 0.763 |  | 0.9982 |  | | key | 2916.27 |  | 0.470 |  | 0.9816 |  | | pc | 2897.85 |  | 0.523 |  | 0.9878 |  | | site | 2896.24 |  | 0.557 |  | 0.9888 |  | | mean | 2888.52 |  | 0.728 |  | 0.9966 |  | | debian | 2888.13 |  | 0.368 |  | 0.9737 |  | | remove | 2880.98 |  | 0.595 |  | 0.9987 |  | | load | 2872.81 |  | 0.566 |  | 0.9934 |  | | installation | 2868.09 |  | 0.547 |  | 0.9919 |  | | nice | 2866.52 |  | 0.567 |  | 0.9793 |  | | send | 2862.22 |  | 0.560 |  | 0.9890 |  | | seem | 2861.04 |  | 0.797 |  | 0.9977 |  | | news | 2850.53 |  | 0.416 |  | 0.9697 |  | | cannot | 2804.12 |  | 0.660 |  | 0.9921 |  | | default | 2787.58 |  | 0.573 |  | 0.9903 |  | | config | 2775.76 |  | 0.381 |  | 0.9356 |  | | floppy | 2773.55 |  | 0.366 |  | 0.9704 |  | | mode | 2770.38 |  | 0.474 |  | 0.9734 |  | | between | 2768.42 |  | 0.743 |  | 0.9977 |  | | yet | 2728.29 |  | 0.786 |  | 0.9986 |  | | answer | 2725.21 |  | 0.610 |  | 0.9921 |  | | made | 2721.55 |  | 0.774 |  | 0.9984 |  | | hat | 2713.92 |  | 0.463 |  | 0.9923 |  | | create | 2708.42 |  | 0.586 |  | 0.9911 |  | | process | 2707.14 |  | 0.509 |  | 0.9755 |  | | bad | 2683.56 |  | 0.635 |  | 0.9890 |  | | idea | 2682.64 |  | 0.787 |  | 0.9989 |  | | looking | 2666.03 |  | 0.743 |  | 0.9976 |  | | end | 2655.78 |  | 0.671 |  | 0.9913 |  | | won't | 2655.10 |  | 0.743 |  | 0.9962 |  | | space | 2653.42 |  | 0.514 |  | 0.9828 |  | | called | 2635.62 |  | 0.755 |  | 0.9966 |  | | distribution | 2612.94 |  | 0.545 |  | 0.9925 |  | | info | 2608.71 |  | 0.620 |  | 0.9951 |  | | shell | 2608.39 |  | 0.438 |  | 0.9700 |  | | ram | 2599.76 |  | 0.401 |  | 0.9737 |  | | come | 2599.43 |  | 0.721 |  | 0.9967 |  | | port | 2590.54 |  | 0.421 |  | 0.9691 |  | | michael | 2574.94 |  | 0.457 |  | 0.9951 |  | | once | 2543.88 |  | 0.791 |  | 0.9981 |  | | above | 2538.54 |  | 0.741 |  | 0.9977 |  | | modem | 2533.73 |  | 0.304 |  | 0.9418 |  | | group | 2532.10 |  | 0.544 |  | 0.9863 |  | | john | 2529.96 |  | 0.481 |  | 0.9840 |  | | list | 2525.49 |  | 0.582 |  | 0.9859 |  | | far | 2520.12 |  | 0.813 |  | 0.9986 |  | | netscape | 2520.02 |  | 0.315 |  | 0.9622 |  | | there's | 2517.90 |  | 0.765 |  | 0.9980 |  | | getting | 2513.37 |  | 0.796 |  | 0.9988 |  | | quite | 2513.07 |  | 0.780 |  | 0.9989 |  | | open | 2511.86 |  | 0.553 |  | 0.9893 |  | | instead | 2500.87 |  | 0.811 |  | 0.9983 |  | | internet | 2489.28 |  | 0.581 |  | 0.9950 |  | | copy | 2486.91 |  | 0.592 |  | 0.9880 |  | | his | 2479.46 |  | 0.487 |  | 0.9881 |  | | add | 2479.36 |  | 0.672 |  | 0.9942 |  | | red | 2466.42 |  | 0.477 |  | 0.9914 |  | | text | 2463.23 |  | 0.475 |  | 0.9828 |  | | part | 2455.63 |  | 0.730 |  | 0.9972 |  | | last | 2423.25 |  | 0.758 |  | 0.9971 |  | | ok | 2407.75 |  | 0.682 |  | 0.9931 |  | | example | 2400.67 |  | 0.727 |  | 0.9977 |  | | option | 2388.81 |  | 0.578 |  | 0.9889 |  | | around | 2376.52 |  | 0.801 |  | 0.9992 |  | | real | 2344.35 |  | 0.694 |  | 0.9966 |  | | nt | 2338.24 |  | 0.316 |  | 0.9781 |  | | possible | 2325.52 |  | 0.745 |  | 0.9967 |  | | package | 2314.65 |  | 0.489 |  | 0.9832 |  | | course | 2309.46 |  | 0.814 |  | 0.9996 |  | | must | 2308.53 |  | 0.735 |  | 0.9956 |  | | already | 2302.44 |  | 0.796 |  | 0.9990 |  | | setup | 2302.02 |  | 0.574 |  | 0.9911 |  | | programs | 2300.00 |  | 0.573 |  | 0.9893 |  | | down | 2295.12 |  | 0.719 |  | 0.9970 |  | | enough | 2294.45 |  | 0.780 |  | 0.9994 |  | | always | 2284.28 |  | 0.794 |  | 0.9978 |  | | slackware | 2280.72 |  | 0.430 |  | 0.9973 |  | | wrong | 2277.25 |  | 0.757 |  | 0.9973 |  | | microsoft | 2276.75 |  | 0.420 |  | 0.9920 |  | | nothing | 2272.88 |  | 0.794 |  | 0.9983 |  | | driver | 2267.63 |  | 0.422 |  | 0.9680 |  | | each | 2266.16 |  | 0.662 |  | 0.9947 |  | | little | 2253.08 |  | 0.789 |  | 0.9988 |  | | number | 2229.03 |  | 0.634 |  | 0.9882 |  | | through | 2210.69 |  | 0.752 |  | 0.9973 |  | | said | 2196.63 |  | 0.720 |  | 0.9994 |  | | tar | 2190.27 |  | 0.278 |  | 0.9281 |  | | following | 2173.53 |  | 0.717 |  | 0.9989 |  | | else | 2166.72 |  | 0.814 |  | 0.9985 |  | | having | 2146.44 |  | 0.811 |  | 0.9994 |  | | give | 2125.11 |  | 0.797 |  | 0.9985 |  | | every | 2107.01 |  | 0.747 |  | 0.9975 |  | | long | 2105.39 |  | 0.732 |  | 0.9981 |  | | write | 2095.28 |  | 0.612 |  | 0.9913 |  | | maybe | 2091.31 |  | 0.775 |  | 0.9969 |  | | scsi | 2081.75 |  | 0.293 |  | 0.9404 |  | | everything | 2080.65 |  | 0.761 |  | 0.9987 |  | | org | 2080.58 |  | 0.529 |  | 0.9977 |  | | least | 2078.65 |  | 0.829 |  | 0.9995 |  | | tell | 2075.56 |  | 0.795 |  | 0.9985 |  | | rpm | 2072.69 |  | 0.289 |  | 0.9370 |  | | web | 2060.80 |  | 0.584 |  | 0.9957 |  | | script | 2055.81 |  | 0.435 |  | 0.9773 |  | | home | 2042.85 |  | 0.629 |  | 0.9956 |  | | information | 2041.72 |  | 0.636 |  | 0.9951 |  | | i'd | 2038.21 |  | 0.753 |  | 0.9980 |  | | didn't | 2037.09 |  | 0.762 |  | 0.9978 |  | | stuff | 2031.82 |  | 0.710 |  | 0.9961 |  | | via | 2023.97 |  | 0.603 |  | 0.9990 |  | | few | 2017.47 |  | 0.794 |  | 0.9992 |  | | working | 2011.92 |  | 0.729 |  | 0.9971 |  | | either | 2002.69 |  | 0.846 |  | 0.9992 |  | | bit | 2001.28 |  | 0.661 |  | 0.9901 |  | | page | 1987.99 |  | 0.572 |  | 0.9908 |  | | device | 1985.10 |  | 0.431 |  | 0.9706 |  | | doing | 1983.10 |  | 0.805 |  | 0.9990 |  | | e-mail | 1981.85 |  | 0.492 |  | 0.9915 |  | | someone | 1973.45 |  | 0.779 |  | 0.9990 |  | | best | 1968.81 |  | 0.699 |  | 0.9968 |  | | isn't | 1963.40 |  | 0.799 |  | 0.9996 |  | | access | 1962.30 |  | 0.573 |  | 0.9891 |  | | our | 1960.46 |  | 0.414 |  | 0.9700 |  | | network | 1960.21 |  | 0.522 |  | 0.9850 |  | | hardware | 1959.24 |  | 0.542 |  | 0.9933 |  | | question | 1947.34 |  | 0.701 |  | 0.9957 |  | | mount | 1943.79 |  | 0.350 |  | 0.9592 |  | | case | 1934.99 |  | 0.745 |  | 0.9968 |  | | net | 1921.74 |  | 0.544 |  | 0.9856 |  | | take | 1901.51 |  | 0.795 |  | 0.9997 |  | | lilo | 1884.06 |  | 0.284 |  | 0.9480 |  | | us | 1873.17 |  | 0.615 |  | 0.9973 |  | | again | 1867.22 |  | 0.792 |  | 0.9976 |  | | change | 1846.92 |  | 0.663 |  | 0.9922 |  | | memory | 1843.29 |  | 0.395 |  | 0.9672 |  | | done | 1836.77 |  | 0.767 |  | 0.9965 |  | | old | 1832.49 |  | 0.660 |  | 0.9931 |  | | actually | 1819.07 |  | 0.807 |  | 0.9996 |  | | however | 1811.78 |  | 0.845 |  | 0.9998 |  | | though | 1807.82 |  | 0.818 |  | 0.9993 |  | | seems | 1801.03 |  | 0.791 |  | 0.9991 |  | | going | 1800.88 |  | 0.796 |  | 0.9990 |  | | hard | 1797.74 |  | 0.630 |  | 0.9909 |  | | data | 1794.45 |  | 0.491 |  | 0.9794 |  | | lot | 1787.26 |  | 0.753 |  | 0.9982 |  | | while | 1775.24 |  | 0.804 |  | 0.9989 |  | | post | 1755.51 |  | 0.562 |  | 0.9912 |  | | point | 1752.57 |  | 0.755 |  | 0.9990 |  | | man | 1726.48 |  | 0.535 |  | 0.9839 |  | | trying | 1723.78 |  | 0.774 |  | 0.9991 |  | | own | 1722.65 |  | 0.723 |  | 0.9991 |  | | found | 1722.51 |  | 0.727 |  | 0.9981 |  | | check | 1720.24 |  | 0.697 |  | 0.9989 |  | | off | 1709.30 |  | 0.721 |  | 0.9938 |  | | being | 1705.59 |  | 0.788 |  | 0.9991 |  | | name | 1689.63 |  | 0.582 |  | 0.9851 |  | | fine | 1688.72 |  | 0.712 |  | 0.9992 |  | | back | 1662.33 |  | 0.770 |  | 0.9978 |  | | source | 1661.49 |  | 0.550 |  | 0.9891 |  | | over | 1660.57 |  | 0.750 |  | 0.9982 |  | | systems | 1658.25 |  | 0.631 |  | 0.9982 |  | | address | 1653.10 |  | 0.508 |  | 0.9907 |  | | type | 1651.89 |  | 0.597 |  | 0.9872 |  | | cd | 1642.26 |  | 0.446 |  | 0.9757 |  | | hi | 1640.50 |  | 0.697 |  | 0.9926 |  | | ftp | 1638.67 |  | 0.420 |  | 0.9901 |  | | put | 1633.89 |  | 0.760 |  | 0.9973 |  | | dos | 1616.79 |  | 0.392 |  | 0.9925 |  | | anything | 1603.63 |  | 0.831 |  | 0.9995 |  | | able | 1589.96 |  | 0.791 |  | 0.9989 |  | | card | 1585.07 |  | 0.413 |  | 0.9754 |  | | yes | 1583.47 |  | 0.713 |  | 0.9939 |  | | box | 1576.72 |  | 0.588 |  | 0.9909 |  | | start | 1570.61 |  | 0.663 |  | 0.9955 |  | | never | 1568.61 |  | 0.768 |  | 0.9995 |  | | comp | 1550.27 |  | 0.487 |  | 0.9887 |  | | different | 1545.32 |  | 0.718 |  | 0.9932 |  | | users | 1543.50 |  | 0.593 |  | 0.9955 |  | | both | 1537.83 |  | 0.742 |  | 0.9948 |  | | available | 1517.49 |  | 0.706 |  | 0.9978 |  | | you're | 1497.64 |  | 0.723 |  | 0.9969 |  | | say | 1491.95 |  | 0.760 |  | 0.9996 |  | | problems | 1488.77 |  | 0.687 |  | 0.9984 |  | | were | 1487.92 |  | 0.690 |  | 0.9978 |  | | mail | 1466.91 |  | 0.481 |  | 0.9890 |  | | etc | 1466.67 |  | 0.733 |  | 0.9953 |  | | www | 1456.78 |  | 0.463 |  | 0.9810 |  | | computer | 1442.54 |  | 0.630 |  | 0.9970 |  | | two | 1439.37 |  | 0.757 |  | 0.9969 |  | | probably | 1438.71 |  | 0.813 |  | 0.9990 |  | | better | 1422.43 |  | 0.757 |  | 0.9994 |  | | thing | 1420.98 |  | 0.830 |  | 0.9997 |  | | another | 1415.96 |  | 0.820 |  | 0.9984 |  | | message | 1400.50 |  | 0.636 |  | 0.9983 |  | | without | 1384.91 |  | 0.824 |  | 0.9993 |  | | its | 1376.39 |  | 0.734 |  | 0.9971 |  | | tried | 1369.52 |  | 0.672 |  | 0.9991 |  | | he | 1353.10 |  | 0.493 |  | 0.9820 |  | | free | 1352.99 |  | 0.529 |  | 0.9924 |  | | those | 1349.85 |  | 0.768 |  | 0.9998 |  | | code | 1345.71 |  | 0.516 |  | 0.9896 |  | | redhat | 1330.08 |  | 0.477 |  | 0.9978 |  | | right | 1319.80 |  | 0.794 |  | 0.9982 |  | | such | 1318.69 |  | 0.756 |  | 0.9972 |  | | error | 1307.75 |  | 0.488 |  | 0.9882 |  | | that's | 1304.92 |  | 0.780 |  | 0.9999 |  | | please | 1304.30 |  | 0.702 |  | 0.9992 |  | | directory | 1283.11 |  | 0.465 |  | 0.9875 |  | | since | 1277.66 |  | 0.813 |  | 0.9996 |  | | works | 1270.30 |  | 0.743 |  | 0.9994 |  | | anyone | 1270.14 |  | 0.774 |  | 0.9997 |  | | things | 1256.33 |  | 0.773 |  | 0.9988 |  | | look | 1253.70 |  | 0.816 |  | 0.9995 |  | | got | 1249.36 |  | 0.756 |  | 0.9983 |  | | unix | 1242.77 |  | 0.478 |  | 0.9972 |  | | installed | 1231.35 |  | 0.617 |  | 0.9992 |  | | email | 1229.38 |  | 0.627 |  | 0.9992 |  | | command | 1226.78 |  | 0.534 |  | 0.9950 |  | | might | 1218.93 |  | 0.808 |  | 0.9981 |  | | before | 1218.67 |  | 0.792 |  | 0.9997 |  | | line | 1205.17 |  | 0.581 |  | 0.9910 |  | | many | 1199.62 |  | 0.727 |  | 0.9991 |  | | go | 1185.89 |  | 0.820 |  | 0.9996 |  | | sure | 1171.34 |  | 0.831 |  | 0.9996 |  | | html | 1158.97 |  | 0.551 |  | 0.9942 |  | | support | 1156.22 |  | 0.583 |  | 0.9938 |  | | program | 1146.24 |  | 0.583 |  | 0.9946 |  | | here | 1140.44 |  | 0.820 |  | 0.9992 |  | | under | 1112.96 |  | 0.667 |  | 0.9966 |  | | most | 1112.54 |  | 0.782 |  | 0.9998 |  | | still | 1104.80 |  | 0.833 |  | 0.9991 |  | | after | 1099.41 |  | 0.779 |  | 0.9987 |  | | did | 1090.90 |  | 0.761 |  | 0.9972 |  | | why | 1076.16 |  | 0.759 |  | 0.9996 |  | | these | 1075.44 |  | 0.790 |  | 0.9986 |  | | too | 1074.74 |  | 0.828 |  | 0.9998 |  | | really | 1036.63 |  | 0.802 |  | 0.9997 |  | | can't | 1034.67 |  | 0.801 |  | 0.9986 |  | | doesn't | 1034.59 |  | 0.825 |  | 0.9993 |  | | who | 1026.26 |  | 0.645 |  | 0.9997 |  | | drive | 994.61 |  | 0.421 |  | 0.9776 |  | | first | 988.17 |  | 0.755 |  | 0.9987 |  | | disk | 984.14 |  | 0.450 |  | 0.9874 |  | | where | 976.04 |  | 0.819 |  | 0.9980 |  | | their | 973.61 |  | 0.627 |  | 0.9983 |  | | server | 972.98 |  | 0.482 |  | 0.9872 |  | | user | 972.72 |  | 0.578 |  | 0.9952 |  | | partition | 966.38 |  | 0.324 |  | 0.9557 |  | | machine | 964.66 |  | 0.587 |  | 0.9945 |  | | used | 947.03 |  | 0.785 |  | 0.9985 |  | | much | 938.40 |  | 0.797 |  | 0.9998 |  | | software | 935.16 |  | 0.556 |  | 0.9941 |  | | people | 918.98 |  | 0.573 |  | 0.9983 |  | | set | 912.51 |  | 0.664 |  | 0.9963 |  | | read | 908.72 |  | 0.681 |  | 0.9975 |  | | os | 900.21 |  | 0.543 |  | 0.9979 |  | | into | 899.37 |  | 0.818 |  | 0.9988 |  | | because | 898.11 |  | 0.779 |  | 0.9999 |  | | very | 897.96 |  | 0.796 |  | 0.9988 |  | | been | 892.83 |  | 0.806 |  | 0.9993 |  | | boot | 884.93 |  | 0.397 |  | 0.9800 |  | | find | 881.53 |  | 0.730 |  | 0.9990 |  | | may | 873.92 |  | 0.763 |  | 0.9973 |  | | even | 872.03 |  | 0.819 |  | 0.9999 |  | | root | 870.45 |  | 0.386 |  | 0.9689 |  | | version | 870.45 |  | 0.599 |  | 0.9955 |  | | install | 869.99 |  | 0.520 |  | 0.9968 |  | | help | 856.01 |  | 0.661 |  | 0.9996 |  | | something | 851.16 |  | 0.828 |  | 0.9995 |  | | good | 833.08 |  | 0.782 |  | 0.9992 |  | | well | 825.81 |  | 0.849 |  | 0.9999 |  | | i've | 825.02 |  | 0.694 |  | 0.9988 |  | | same | 823.51 |  | 0.793 |  | 0.9985 |  | | new | 810.96 |  | 0.710 |  | 0.9963 |  | | files | 784.61 |  | 0.530 |  | 0.9966 |  | | had | 783.46 |  | 0.710 |  | 0.9986 |  | | running | 780.62 |  | 0.678 |  | 0.9992 |  | | we | 779.87 |  | 0.513 |  | 0.9827 |  | | way | 772.45 |  | 0.826 |  | 0.9994 |  | | could | 769.72 |  | 0.782 |  | 0.9994 |  | | try | 761.27 |  | 0.751 |  | 0.9997 |  | | thanks | 757.96 |  | 0.639 |  | 0.9771 |  | | am | 747.39 |  | 0.689 |  | 0.9989 |  | | windows | 736.29 |  | 0.494 |  | 0.9941 |  | | think | 735.53 |  | 0.813 |  | 0.9998 |  | | now | 713.95 |  | 0.825 |  | 0.9999 |  | | com | 700.91 |  | 0.513 |  | 0.9991 |  | | them | 686.80 |  | 0.749 |  | 0.9971 |  | | than | 674.97 |  | 0.770 |  | 0.9999 |  | | run | 661.19 |  | 0.653 |  | 0.9992 |  | | see | 659.48 |  | 0.823 |  | 0.9999 |  | | article | 637.05 |  | 0.596 |  | 0.9890 |  | | does | 622.37 |  | 0.796 |  | 0.9995 |  | | time | 621.42 |  | 0.765 |  | 0.9985 |  | | make | 618.14 |  | 0.746 |  | 0.9956 |  | | also | 614.12 |  | 0.860 |  | 0.9999 |  | | need | 607.70 |  | 0.761 |  | 0.9996 |  | | problem | 602.40 |  | 0.660 |  | 0.9991 |  | | work | 591.04 |  | 0.758 |  | 0.9995 |  | | want | 584.59 |  | 0.756 |  | 0.9996 |  | | should | 583.33 |  | 0.784 |  | 0.9988 |  | | other | 560.47 |  | 0.867 |  | 0.9998 |  | | i'm | 556.64 |  | 0.709 |  | 0.9990 |  | | know | 530.78 |  | 0.818 |  | 0.9997 |  | | using | 509.62 |  | 0.752 |  | 0.9994 |  | | only | 507.39 |  | 0.878 |  | 0.9999 |  | | out | 505.80 |  | 0.832 |  | 0.9998 |  | | how | 501.64 |  | 0.805 |  | 0.9994 |  | | kernel | 497.93 |  | 0.426 |  | 0.9909 |  | | more | 475.53 |  | 0.783 |  | 0.9997 |  | | it's | 461.54 |  | 0.743 |  | 0.9998 |  | | has | 452.68 |  | 0.844 |  | 0.9997 |  | | about | 443.60 |  | 0.809 |  | 0.9999 |  | | then | 442.10 |  | 0.786 |  | 0.9996 |  | | wrote | 436.80 |  | 0.587 |  | 0.9677 |  | | system | 435.89 |  | 0.657 |  | 0.9991 |  | | up | 429.15 |  | 0.801 |  | 0.9998 |  | | me | 420.32 |  | 0.786 |  | 0.9997 |  | | some | 417.65 |  | 0.843 |  | 0.9999 |  | | they | 412.26 |  | 0.638 |  | 0.9984 |  | | file | 409.01 |  | 0.501 |  | 0.9975 |  | | when | 400.59 |  | 0.815 |  | 0.9999 |  | | which | 389.00 |  | 0.822 |  | 0.9998 |  | | by | 388.51 |  | 0.818 |  | 0.9998 |  | | don't | 385.83 |  | 0.823 |  | 0.9999 |  | | would | 380.79 |  | 0.743 |  | 0.9988 |  | | like | 369.78 |  | 0.831 |  | 0.9999 |  | | any | 369.37 |  | 0.850 |  | 0.9998 |  | | just | 359.23 |  | 0.810 |  | 0.9981 |  | | no | 359.21 |  | 0.813 |  | 0.9997 |  | | one | 357.69 |  | 0.831 |  | 0.9996 |  | | was | 357.22 |  | 0.665 |  | 0.9975 |  | | get | 346.66 |  | 0.787 |  | 0.9996 |  | | will | 326.67 |  | 0.760 |  | 0.9989 |  | | an | 312.26 |  | 0.825 |  | 0.9993 |  | | there | 311.08 |  | 0.827 |  | 0.9999 |  | | use | 301.65 |  | 0.727 |  | 0.9985 |  | | what | 294.88 |  | 0.801 |  | 0.9995 |  | | all | 292.53 |  | 0.871 |  | 0.9999 |  | | so | 271.53 |  | 0.832 |  | 0.9998 |  | | from | 251.19 |  | 0.813 |  | 0.9998 |  | | at | 250.13 |  | 0.838 |  | 0.9999 |  | | your | 237.87 |  | 0.690 |  | 0.9987 |  | | do | 235.10 |  | 0.816 |  | 0.9999 |  | | http | 215.44 |  | 0.525 |  | 0.9868 |  | | can | 211.15 |  | 0.810 |  | 0.9994 |  | | my | 197.43 |  | 0.692 |  | 0.9985 |  | | as | 189.40 |  | 0.784 |  | 0.9976 |  | | are | 186.88 |  | 0.792 |  | 0.9998 |  | | or | 185.73 |  | 0.831 |  | 0.9997 |  | | but | 171.25 |  | 0.785 |  | 0.9826 |  | | if | 162.80 |  | 0.819 |  | 0.9978 |  | | be | 159.48 |  | 0.783 |  | 0.9935 |  | | not | 154.92 |  | 0.789 |  | 0.9951 |  | | linux | 143.26 |  | 0.515 |  | 0.9586 |  | | this | 143.07 |  | 0.723 |  | 0.9825 |  | | have | 136.80 |  | 0.812 |  | 0.9963 |  | | with | 134.11 |  | 0.847 |  | 0.9986 |  | | on | 123.51 |  | 0.821 |  | 0.9983 |  | | for | 97.04 |  | 0.814 |  | 0.9960 |  | | that | 83.90 |  | 0.700 |  | 0.9712 |  | | in | 74.35 |  | 0.778 |  | 0.9789 |  | | you | 73.04 |  | 0.625 |  | 0.9868 |  | | it | 70.74 |  | 0.650 |  | 0.9540 |  | | of | 66.90 |  | 0.754 |  | 0.9819 |  | | is | 63.95 |  | 0.706 |  | 0.9619 |  | | and | 52.49 |  | 0.701 |  | 0.9262 |  | | i | 47.17 |  | 0.511 |  | 0.8933 |  | | a | 45.00 |  | 0.602 |  | 0.8649 |  | | to | 35.37 |  | 0.596 |  | 0.8324 |  | | the | 23.38 |  | 0.420 |  | 0.5450 |  | |
