# Supplementary material for: Beyond Word Frequency: Bursts, Lulls, and Scaling in the Temporal Distributions of Words
Source: PLoS One. 2009 Nov 11;4(11):e7678. doi: 10.1371/journal.pone.0007678 (PMC2770836; doi:10.1371/journal.pone.0007678)
Supplement: Table S1 — Detailed information on the statistical analysis of all words that were studied (six databases). (31.88 MB TAR) [file pone.0007678.s002.tar › recurrence/debates/index.html]

Word < tau > | ------ | beta | ----- | R^2 | ----- || i'm | 470.99 |  | 0.879 |  | 0.9927 |  | | by | 466.33 |  | 0.972 |  | 0.9883 |  | | don't | 466.33 |  | 0.886 |  | 0.9895 |  | | an | 436.10 |  | 0.919 |  | 0.9908 |  | | from | 436.10 |  | 0.711 |  | 0.9749 |  | | health | 432.10 |  | 0.361 |  | 0.8414 |  | | tax | 432.10 |  | 0.316 |  | 0.7489 |  | | well | 432.10 |  | 1.199 |  | 0.9909 |  | | go | 420.53 |  | 0.845 |  | 0.9947 |  | | when | 420.53 |  | 0.782 |  | 0.9963 |  | | one | 416.81 |  | 0.836 |  | 0.9978 |  | | up | 402.56 |  | 0.806 |  | 0.9943 |  | | get | 389.25 |  | 0.778 |  | 0.9964 |  | | right | 379.83 |  | 0.966 |  | 0.9940 |  | | their | 379.83 |  | 0.763 |  | 0.9965 |  | | some | 367.96 |  | 0.781 |  | 0.9990 |  | | want | 367.96 |  | 0.730 |  | 0.9966 |  | | out | 365.11 |  | 0.857 |  | 0.9939 |  | | who | 362.30 |  | 0.784 |  | 0.9926 |  | | said | 359.53 |  | 0.725 |  | 0.9921 |  | | would | 359.53 |  | 0.611 |  | 0.9954 |  | | been | 354.13 |  | 0.865 |  | 0.9954 |  | | your | 348.88 |  | 0.505 |  | 0.9820 |  | | just | 343.79 |  | 0.791 |  | 0.9855 |  | | at | 331.68 |  | 0.816 |  | 0.9948 |  | | there | 324.82 |  | 0.753 |  | 0.9888 |  | | my | 320.40 |  | 0.588 |  | 0.9922 |  | | we're | 320.40 |  | 0.657 |  | 0.9959 |  | | people | 318.24 |  | 0.804 |  | 0.9969 |  | | them | 318.24 |  | 0.726 |  | 0.9818 |  | | we've | 313.99 |  | 0.626 |  | 0.9900 |  | | because | 309.86 |  | 0.950 |  | 0.9953 |  | | make | 298.09 |  | 0.700 |  | 0.9898 |  | | has | 294.37 |  | 0.820 |  | 0.9935 |  | | obama | 288.95 |  | 0.939 |  | 0.9837 |  | | mccain | 280.35 |  | 0.874 |  | 0.9956 |  | | all | 272.25 |  | 0.952 |  | 0.9939 |  | | know | 272.25 |  | 0.650 |  | 0.9937 |  | | can | 267.61 |  | 0.682 |  | 0.9897 |  | | he | 266.10 |  | 0.492 |  | 0.9785 |  | | that's | 266.10 |  | 0.802 |  | 0.9980 |  | | if | 263.12 |  | 0.864 |  | 0.9934 |  | | it's | 261.66 |  | 0.802 |  | 0.9945 |  | | got | 260.22 |  | 0.633 |  | 0.9932 |  | | was | 257.37 |  | 0.636 |  | 0.9948 |  | | about | 254.59 |  | 0.815 |  | 0.9956 |  | | think | 249.20 |  | 0.783 |  | 0.9961 |  | | now | 247.89 |  | 0.997 |  | 0.9942 |  | | will | 247.89 |  | 0.703 |  | 0.9791 |  | | they | 244.04 |  | 0.694 |  | 0.9778 |  | | so | 234.32 |  | 1.067 |  | 0.9957 |  | | as | 233.16 |  | 0.791 |  | 0.9833 |  | | what | 205.67 |  | 0.857 |  | 0.9963 |  | | do | 187.65 |  | 0.756 |  | 0.9971 |  | | be | 177.06 |  | 0.876 |  | 0.9985 |  | | but | 165.84 |  | 1.001 |  | 0.9944 |  | | our | 160.20 |  | 0.727 |  | 0.9966 |  | | going | 157.52 |  | 0.816 |  | 0.9933 |  | | not | 157.00 |  | 0.925 |  | 0.9984 |  | | with | 152.92 |  | 0.802 |  | 0.9975 |  | | this | 150.48 |  | 0.865 |  | 0.9978 |  | | on | 146.27 |  | 0.891 |  | 0.9988 |  | | are | 136.92 |  | 0.801 |  | 0.9989 |  | | senator | 122.02 |  | 0.968 |  | 0.9980 |  | | for | 120.15 |  | 0.825 |  | 0.9918 |  | | it | 114.60 |  | 0.828 |  | 0.9919 |  | | is | 77.34 |  | 0.958 |  | 0.9994 |  | | you | 75.84 |  | 0.666 |  | 0.9831 |  | | have | 72.46 |  | 0.889 |  | 0.9989 |  | | in | 61.33 |  | 0.899 |  | 0.9905 |  | | i | 57.51 |  | 0.833 |  | 0.9958 |  | | we | 53.89 |  | 0.722 |  | 0.9854 |  | | a | 49.73 |  | 0.945 |  | 0.9970 |  | | of | 42.20 |  | 0.854 |  | 0.9821 |  | | that | 37.09 |  | 0.796 |  | 0.9557 |  | | and | 27.89 |  | 1.063 |  | 0.9985 |  | | to | 25.81 |  | 0.891 |  | 0.9877 |  | | the | 23.42 |  | 0.955 |  | 0.9921 |  | |
