# Supplementary material for: Beyond Word Frequency: Bursts, Lulls, and Scaling in the Temporal Distributions of Words
Source: PLoS One. 2009 Nov 11;4(11):e7678. doi: 10.1371/journal.pone.0007678 (PMC2770836; doi:10.1371/journal.pone.0007678)
Supplement: Table S1 — Detailed information on the statistical analysis of all words that were studied (six databases). (31.88 MB TAR) [file pone.0007678.s002.tar › recurrence/index.html]

## Databases:

 Usenet group talk.origins  
 Usenet group comp.os.linux.misc   
 War and Peace, by L. Tolstoy   
 Os Sertoes, by E. Da Cunha   
 Debates of the 2008 US presidential election   
 Newton's Principia
