# Supplementary material for: Beyond Word Frequency: Bursts, Lulls, and Scaling in the Temporal Distributions of Words
Source: PLoS One. 2009 Nov 11;4(11):e7678. doi: 10.1371/journal.pone.0007678 (PMC2770836; doi:10.1371/journal.pone.0007678)
Supplement: Table S1 — Detailed information on the statistical analysis of all words that were studied (six databases). (31.88 MB TAR) [file pone.0007678.s002.tar › recurrence/principia/index.html]

Word < tau > | ------ | beta | ----- | R^2 | ----- || comets | 2133.38 |  | 0.308 |  | 0.6925 |  | | descent | 2133.38 |  | 0.442 |  | 0.9761 |  | | focus | 2133.38 |  | 0.341 |  | 0.8915 |  | | squares | 2133.38 |  | 0.406 |  | 0.9826 |  | | beginning | 2112.26 |  | 0.416 |  | 0.9853 |  | | describes | 2112.26 |  | 0.610 |  | 0.9787 |  | | pt | 2112.26 |  | 0.253 |  | 0.7738 |  | | direction | 2091.55 |  | 0.459 |  | 0.9885 |  | | our | 2091.55 |  | 0.456 |  | 0.9865 |  | | contrary | 2071.24 |  | 0.535 |  | 0.9903 |  | | great | 2071.24 |  | 0.500 |  | 0.9907 |  | | say | 2071.24 |  | 0.786 |  | 0.9732 |  | | do | 2051.33 |  | 0.602 |  | 0.9972 |  | | height | 2051.33 |  | 0.331 |  | 0.9130 |  | | placed | 2051.33 |  | 0.517 |  | 0.9902 |  | | uniformly | 2051.33 |  | 0.539 |  | 0.9808 |  | | diameters | 2031.79 |  | 0.387 |  | 0.9626 |  | | last | 2031.79 |  | 0.610 |  | 0.9906 |  | | take | 2031.79 |  | 0.679 |  | 0.9970 |  | | position | 1993.81 |  | 0.567 |  | 0.9895 |  | | syzygies | 1993.81 |  | 0.311 |  | 0.7226 |  | | vessel | 1993.81 |  | 0.256 |  | 0.5364 |  | | according | 1975.35 |  | 0.589 |  | 0.9877 |  | | angles | 1975.35 |  | 0.381 |  | 0.9760 |  | | cut | 1975.35 |  | 0.438 |  | 0.9712 |  | | taken | 1975.35 |  | 0.905 |  | 0.9970 |  | | tangent | 1975.35 |  | 0.400 |  | 0.9549 |  | | trajectory | 1975.35 |  | 0.320 |  | 0.7258 |  | | go | 1957.23 |  | 0.609 |  | 0.9903 |  | | ef | 1939.44 |  | 0.248 |  | 0.7394 |  | | spaces | 1939.44 |  | 0.423 |  | 0.9782 |  | | number | 1921.96 |  | 0.466 |  | 0.9857 |  | | pendulum | 1921.96 |  | 0.316 |  | 0.7385 |  | | cylinder | 1904.80 |  | 0.188 |  | 0.2462 |  | | together | 1904.80 |  | 0.574 |  | 0.9818 |  | | without | 1904.80 |  | 0.817 |  | 0.9904 |  | | conic | 1871.39 |  | 0.399 |  | 0.8761 |  | | manner | 1871.39 |  | 0.713 |  | 0.9881 |  | | sides | 1871.39 |  | 0.396 |  | 0.9765 |  | | jupiter | 1839.12 |  | 0.241 |  | 0.5992 |  | | must | 1839.12 |  | 0.576 |  | 0.9966 |  | | section | 1839.12 |  | 0.370 |  | 0.9427 |  | | problem | 1823.40 |  | 0.715 |  | 0.9849 |  | | shall | 1807.95 |  | 0.592 |  | 0.9946 |  | | planets | 1792.76 |  | 0.400 |  | 0.8796 |  | | inches | 1763.12 |  | 0.181 |  | 0.2286 |  | | only | 1763.12 |  | 0.680 |  | 0.9938 |  | | sine | 1763.12 |  | 0.291 |  | 0.8803 |  | | superficies | 1763.12 |  | 0.275 |  | 0.7748 |  | | find | 1748.67 |  | 0.821 |  | 0.9937 |  | | some | 1748.67 |  | 0.558 |  | 0.9914 |  | | attracted | 1734.46 |  | 0.373 |  | 0.9067 |  | | side | 1734.46 |  | 0.465 |  | 0.9898 |  | | themselves | 1734.46 |  | 0.556 |  | 0.9922 |  | | theorem | 1734.46 |  | 0.924 |  | 0.9571 |  | | ps | 1706.70 |  | 0.242 |  | 0.6479 |  | | directly | 1693.16 |  | 0.497 |  | 0.9932 |  | | hence | 1679.83 |  | 0.942 |  | 0.9814 |  | | draw | 1666.70 |  | 0.512 |  | 0.9800 |  | | k | 1666.70 |  | 0.449 |  | 0.9966 |  | | where | 1666.70 |  | 0.633 |  | 0.9924 |  | | bd | 1653.78 |  | 0.235 |  | 0.6359 |  | | cd | 1641.06 |  | 0.324 |  | 0.9217 |  | | feet | 1641.06 |  | 0.291 |  | 0.8061 |  | | ad | 1628.53 |  | 0.251 |  | 0.8624 |  | | arcs | 1628.53 |  | 0.249 |  | 0.7439 |  | | either | 1628.53 |  | 0.667 |  | 0.9957 |  | | rest | 1628.53 |  | 0.404 |  | 0.9609 |  | | areas | 1616.20 |  | 0.392 |  | 0.9588 |  | | common | 1616.20 |  | 0.323 |  | 0.8563 |  | | several | 1616.20 |  | 0.618 |  | 0.9933 |  | | quadratures | 1580.28 |  | 0.375 |  | 0.8095 |  | | book | 1557.21 |  | 0.500 |  | 0.9937 |  | | falling | 1545.93 |  | 0.395 |  | 0.8842 |  | | out | 1545.93 |  | 0.522 |  | 0.9873 |  | | air | 1534.81 |  | 0.312 |  | 0.8324 |  | | sphere | 1534.81 |  | 0.216 |  | 0.5309 |  | | curve | 1513.04 |  | 0.405 |  | 0.9596 |  | | every | 1502.38 |  | 0.517 |  | 0.9954 |  | | no | 1502.38 |  | 0.558 |  | 0.9938 |  | | similar | 1502.38 |  | 0.418 |  | 0.9817 |  | | square | 1502.38 |  | 0.417 |  | 0.9815 |  | | third | 1502.38 |  | 0.512 |  | 0.9940 |  | | second | 1491.87 |  | 0.482 |  | 0.9913 |  | | above | 1461.22 |  | 0.703 |  | 0.9961 |  | | made | 1451.28 |  | 0.655 |  | 0.9953 |  | | ellipsis | 1422.25 |  | 0.318 |  | 0.8315 |  | | after | 1412.83 |  | 0.529 |  | 0.9931 |  | | sum | 1412.83 |  | 0.402 |  | 0.9778 |  | | before | 1394.37 |  | 0.619 |  | 0.9943 |  | | little | 1394.37 |  | 0.387 |  | 0.9702 |  | | like | 1367.55 |  | 0.627 |  | 0.9958 |  | | parallel | 1350.24 |  | 0.533 |  | 0.9915 |  | | both | 1333.36 |  | 0.749 |  | 0.9957 |  | | nodes | 1325.08 |  | 0.291 |  | 0.5633 |  | | rectangle | 1325.08 |  | 0.297 |  | 0.9100 |  | | o | 1316.90 |  | 0.405 |  | 0.9913 |  | | diminished | 1308.82 |  | 0.510 |  | 0.9857 |  | | suppose | 1300.84 |  | 0.831 |  | 0.9958 |  | | pq | 1277.47 |  | 0.312 |  | 0.8305 |  | | such | 1262.36 |  | 0.720 |  | 0.9926 |  | | sp | 1254.93 |  | 0.244 |  | 0.6758 |  | | quantity | 1247.59 |  | 0.481 |  | 0.9889 |  | | move | 1240.34 |  | 0.656 |  | 0.9879 |  | | matter | 1233.17 |  | 0.310 |  | 0.8264 |  | | radius | 1226.08 |  | 0.453 |  | 0.9839 |  | | velocities | 1226.08 |  | 0.364 |  | 0.9142 |  | | another | 1219.07 |  | 0.742 |  | 0.9974 |  | | very | 1219.07 |  | 0.653 |  | 0.9947 |  | | nearly | 1185.21 |  | 0.578 |  | 0.9871 |  | | m | 1178.66 |  | 0.391 |  | 0.9757 |  | | perpendicular | 1172.19 |  | 0.605 |  | 0.9908 |  | | l | 1153.18 |  | 0.405 |  | 0.9773 |  | | were | 1153.18 |  | 0.500 |  | 0.9931 |  | | g | 1140.84 |  | 0.405 |  | 0.9811 |  | | places | 1134.78 |  | 0.445 |  | 0.9893 |  | | diameter | 1128.77 |  | 0.404 |  | 0.9754 |  | | whose | 1128.77 |  | 0.609 |  | 0.9950 |  | | difference | 1122.83 |  | 0.408 |  | 0.9476 |  | | duplicate | 1122.83 |  | 0.499 |  | 0.9875 |  | | greatest | 1122.83 |  | 0.479 |  | 0.9631 |  | | always | 1111.14 |  | 0.660 |  | 0.9964 |  | | now | 1082.93 |  | 0.869 |  | 0.9978 |  | | centripetal | 1077.46 |  | 0.492 |  | 0.9071 |  | | de | 1077.46 |  | 0.470 |  | 0.9911 |  | | fall | 1072.05 |  | 0.566 |  | 0.9885 |  | | figure | 1066.69 |  | 0.373 |  | 0.9625 |  | | density | 1050.93 |  | 0.328 |  | 0.8796 |  | | r | 1040.67 |  | 0.533 |  | 0.9950 |  | | them | 1040.67 |  | 0.715 |  | 0.9983 |  | | case | 1015.90 |  | 0.563 |  | 0.9876 |  | | since | 1011.08 |  | 0.755 |  | 0.9955 |  | | ac | 1006.31 |  | 0.332 |  | 0.9096 |  | | reciprocally | 1006.31 |  | 0.421 |  | 0.9584 |  | | axis | 1001.59 |  | 0.383 |  | 0.9124 |  | | fluid | 965.33 |  | 0.289 |  | 0.6248 |  | | found | 960.98 |  | 0.597 |  | 0.9951 |  | | weight | 960.98 |  | 0.261 |  | 0.7320 |  | | h | 935.69 |  | 0.428 |  | 0.9840 |  | | drawn | 907.82 |  | 0.600 |  | 0.9933 |  | | particles | 907.82 |  | 0.298 |  | 0.7918 |  | | orbit | 900.16 |  | 0.432 |  | 0.9353 |  | | medium | 892.63 |  | 0.362 |  | 0.7964 |  | | proportion | 892.63 |  | 0.428 |  | 0.9742 |  | | being | 877.93 |  | 0.766 |  | 0.9985 |  | | less | 874.34 |  | 0.557 |  | 0.9949 |  | | between | 860.23 |  | 0.541 |  | 0.9941 |  | | v | 856.78 |  | 0.439 |  | 0.9945 |  | | mean | 836.62 |  | 0.404 |  | 0.9603 |  | | prop | 833.35 |  | 0.610 |  | 0.9907 |  | | plane | 830.11 |  | 0.300 |  | 0.8659 |  | | space | 830.11 |  | 0.369 |  | 0.9543 |  | | comet | 817.39 |  | 0.358 |  | 0.5193 |  | | n | 811.17 |  | 0.363 |  | 0.9759 |  | | part | 808.10 |  | 0.456 |  | 0.9823 |  | | length | 799.02 |  | 0.390 |  | 0.9408 |  | | proposition | 796.04 |  | 0.793 |  | 0.9941 |  | | first | 793.08 |  | 0.577 |  | 0.9948 |  | | f | 784.33 |  | 0.500 |  | 0.9951 |  | | motions | 781.46 |  | 0.434 |  | 0.9637 |  | | arc | 767.40 |  | 0.318 |  | 0.8803 |  | | points | 748.55 |  | 0.351 |  | 0.8970 |  | | also | 745.94 |  | 0.833 |  | 0.9968 |  | | distances | 740.76 |  | 0.461 |  | 0.9649 |  | | globe | 733.12 |  | 0.223 |  | 0.4038 |  | | would | 730.61 |  | 0.464 |  | 0.9812 |  | | more | 728.12 |  | 0.514 |  | 0.9963 |  | | have | 720.74 |  | 0.735 |  | 0.9972 |  | | these | 718.31 |  | 0.816 |  | 0.9986 |  | | then | 715.90 |  | 0.959 |  | 0.9946 |  | | greater | 713.51 |  | 0.521 |  | 0.9899 |  | | + | 706.42 |  | 0.264 |  | 0.8889 |  | | each | 699.47 |  | 0.520 |  | 0.9787 |  | | into | 694.91 |  | 0.551 |  | 0.9952 |  | | because | 692.66 |  | 0.778 |  | 0.9967 |  | | ab | 690.41 |  | 0.334 |  | 0.8992 |  | | when | 685.97 |  | 0.512 |  | 0.9902 |  | | describe | 683.78 |  | 0.546 |  | 0.9785 |  | | lines | 672.99 |  | 0.442 |  | 0.9603 |  | | water | 668.77 |  | 0.219 |  | 0.3987 |  | | circle | 666.68 |  | 0.349 |  | 0.8869 |  | | upon | 666.68 |  | 0.653 |  | 0.9965 |  | | there | 648.44 |  | 0.723 |  | 0.9948 |  | | through | 640.65 |  | 0.533 |  | 0.9834 |  | | they | 621.98 |  | 0.501 |  | 0.9925 |  | | place | 620.17 |  | 0.463 |  | 0.9890 |  | | two | 606.07 |  | 0.524 |  | 0.9936 |  | | area | 602.65 |  | 0.291 |  | 0.8028 |  | | angle | 594.26 |  | 0.293 |  | 0.9035 |  | | c | 594.26 |  | 0.478 |  | 0.9709 |  | | gravity | 592.61 |  | 0.394 |  | 0.8784 |  | | whole | 592.61 |  | 0.556 |  | 0.9940 |  | | proportional | 578.15 |  | 0.541 |  | 0.9883 |  | | towards | 562.90 |  | 0.425 |  | 0.9653 |  | | resistance | 548.43 |  | 0.368 |  | 0.8210 |  | | described | 547.02 |  | 0.555 |  | 0.9849 |  | | not | 547.02 |  | 0.622 |  | 0.9912 |  | | t | 538.73 |  | 0.439 |  | 0.9811 |  | | times | 532.01 |  | 0.428 |  | 0.9578 |  | | q | 525.46 |  | 0.645 |  | 0.9864 |  | | b | 521.61 |  | 0.375 |  | 0.9311 |  | | earth | 515.31 |  | 0.349 |  | 0.7757 |  | | than | 514.07 |  | 0.522 |  | 0.9798 |  | | forces | 506.74 |  | 0.424 |  | 0.9094 |  | | we | 506.74 |  | 0.589 |  | 0.9944 |  | | on | 498.45 |  | 0.687 |  | 0.9980 |  | | moon | 492.70 |  | 0.276 |  | 0.4552 |  | | point | 478.34 |  | 0.439 |  | 0.9660 |  | | e | 471.99 |  | 0.674 |  | 0.9869 |  | | about | 466.82 |  | 0.590 |  | 0.9868 |  | | was | 465.80 |  | 0.402 |  | 0.9385 |  | | d | 459.78 |  | 0.567 |  | 0.9854 |  | | an | 454.88 |  | 0.750 |  | 0.9976 |  | | distance | 444.45 |  | 0.480 |  | 0.9658 |  | | so | 430.99 |  | 0.790 |  | 0.9985 |  | | sun | 426.68 |  | 0.368 |  | 0.7920 |  | | parts | 404.82 |  | 0.425 |  | 0.9643 |  | | p | 400.26 |  | 0.408 |  | 0.9121 |  | | one | 395.80 |  | 0.686 |  | 0.9983 |  | | all | 378.93 |  | 0.632 |  | 0.9962 |  | | velocity | 371.02 |  | 0.395 |  | 0.9170 |  | | right | 370.38 |  | 0.448 |  | 0.9461 |  | | those | 367.82 |  | 0.702 |  | 0.9969 |  | | cor | 367.19 |  | 0.593 |  | 0.9630 |  | | s | 345.77 |  | 0.523 |  | 0.9804 |  | | may | 342.99 |  | 0.660 |  | 0.9940 |  | | their | 341.34 |  | 0.515 |  | 0.9907 |  | | let | 340.80 |  | 0.573 |  | 0.9870 |  | | bodies | 338.63 |  | 0.357 |  | 0.8267 |  | | centre | 330.76 |  | 0.466 |  | 0.9478 |  | | other | 323.24 |  | 0.668 |  | 0.9915 |  | | for | 306.96 |  | 0.917 |  | 0.9936 |  | | any | 300.90 |  | 0.665 |  | 0.9948 |  | | x | 300.90 |  | 0.297 |  | 0.9352 |  | | line | 296.30 |  | 0.435 |  | 0.9302 |  | | ratio | 285.98 |  | 0.421 |  | 0.9491 |  | | i | 280.71 |  | 0.704 |  | 0.9968 |  | | given | 276.34 |  | 0.418 |  | 0.9694 |  | | time | 268.01 |  | 0.514 |  | 0.9622 |  | | but | 257.03 |  | 0.960 |  | 0.9952 |  | | this | 249.81 |  | 0.834 |  | 0.9971 |  | | at | 241.06 |  | 0.639 |  | 0.9973 |  | | motion | 231.39 |  | 0.440 |  | 0.9453 |  | | equal | 221.30 |  | 0.598 |  | 0.9928 |  | | body | 205.92 |  | 0.397 |  | 0.8242 |  | | its | 205.13 |  | 0.627 |  | 0.9959 |  | | if | 196.99 |  | 0.819 |  | 0.9874 |  | | force | 196.44 |  | 0.411 |  | 0.8833 |  | | therefore | 195.01 |  | 0.782 |  | 0.9875 |  | | same | 193.24 |  | 0.788 |  | 0.9974 |  | | it | 179.43 |  | 0.680 |  | 0.9962 |  | | with | 175.59 |  | 0.736 |  | 0.9959 |  | | or | 167.59 |  | 0.709 |  | 0.9987 |  | | are | 166.67 |  | 0.616 |  | 0.9866 |  | | which | 125.05 |  | 0.770 |  | 0.9851 |  | | from | 122.96 |  | 0.686 |  | 0.9795 |  | | will | 109.24 |  | 0.642 |  | 0.9570 |  | | by | 90.02 |  | 0.820 |  | 0.9920 |  | | that | 81.12 |  | 0.853 |  | 0.9891 |  | | as | 71.98 |  | 0.687 |  | 0.9839 |  | | a | 67.05 |  | 0.723 |  | 0.9943 |  | | be | 66.13 |  | 0.692 |  | 0.9533 |  | | is | 63.46 |  | 0.686 |  | 0.9644 |  | | in | 38.06 |  | 0.719 |  | 0.9472 |  | | to | 35.17 |  | 0.652 |  | 0.9292 |  | | and | 29.68 |  | 0.732 |  | 0.8743 |  | | of | 21.95 |  | 0.586 |  | 0.7777 |  | | the | 8.62 |  | 0.265 |  | 0.0377 |  | |
