# Supplementary material for: Beyond Word Frequency: Bursts, Lulls, and Scaling in the Temporal Distributions of Words
Source: PLoS One. 2009 Nov 11;4(11):e7678. doi: 10.1371/journal.pone.0007678 (PMC2770836; doi:10.1371/journal.pone.0007678)
Supplement: Table S1 — Detailed information on the statistical analysis of all words that were studied (six databases). (31.88 MB TAR) [file pone.0007678.s002.tar › recurrence/sertoes/index.html]

Word < tau > | ------ | beta | ----- | R^2 | ----- || havia | 1550.30 |  | 0.758 |  | 0.9868 |  | | lo | 1550.30 |  | 0.635 |  | 0.9896 |  | | marcha | 1550.30 |  | 0.750 |  | 0.9937 |  | | luta | 1535.10 |  | 1.005 |  | 0.9961 |  | | la' | 1535.10 |  | 0.844 |  | 0.9952 |  | | sul | 1535.10 |  | 0.721 |  | 0.9735 |  | | unico | 1535.10 |  | 0.955 |  | 0.9950 |  | | alto | 1505.58 |  | 0.720 |  | 0.9838 |  | | aquele | 1505.58 |  | 1.076 |  | 0.9923 |  | | monte | 1491.24 |  | 0.712 |  | 0.9859 |  | | combate | 1477.17 |  | 0.783 |  | 0.9733 |  | | ja' | 1477.17 |  | 0.803 |  | 0.9976 |  | | noite | 1477.17 |  | 0.640 |  | 0.9923 |  | | batalhoes | 1463.36 |  | 0.863 |  | 0.9663 |  | | oficiais | 1463.36 |  | 0.385 |  | 0.9437 |  | | duas | 1449.81 |  | 0.750 |  | 0.9951 |  | | santo | 1423.45 |  | 0.617 |  | 0.9934 |  | | frente | 1398.04 |  | 0.828 |  | 0.9594 |  | | mesma | 1385.66 |  | 0.688 |  | 0.9859 |  | | fim | 1373.51 |  | 1.047 |  | 0.9975 |  | | meio | 1373.51 |  | 0.705 |  | 0.9913 |  | | inimigo | 1326.95 |  | 0.719 |  | 0.9803 |  | | apenas | 1315.80 |  | 1.026 |  | 0.9931 |  | | coronel | 1315.80 |  | 0.546 |  | 0.9885 |  | | dentro | 1294.05 |  | 0.863 |  | 0.9952 |  | | linha | 1283.44 |  | 0.727 |  | 0.9942 |  | | soldados | 1283.44 |  | 0.917 |  | 0.9835 |  | | seu | 1262.74 |  | 0.737 |  | 0.9933 |  | | tinham | 1252.64 |  | 0.800 |  | 0.9918 |  | | alguns | 1232.91 |  | 0.839 |  | 0.9950 |  | | parte | 1232.91 |  | 1.055 |  | 0.9919 |  | | vida | 1213.80 |  | 0.731 |  | 0.9728 |  | | jaguncos | 1204.46 |  | 1.027 |  | 0.9482 |  | | pracas | 1204.46 |  | 0.450 |  | 0.9249 |  | | quase | 1204.46 |  | 0.880 |  | 0.9959 |  | | entao | 1168.51 |  | 1.003 |  | 0.9918 |  | | rio | 1168.51 |  | 0.713 |  | 0.9830 |  | | grande | 1142.92 |  | 0.919 |  | 0.9958 |  | | modo | 1142.92 |  | 0.956 |  | 0.9917 |  | | tres | 1134.64 |  | 0.869 |  | 0.9983 |  | | eram | 1126.47 |  | 0.761 |  | 0.9955 |  | | outros | 1110.50 |  | 0.861 |  | 0.9957 |  | | arraial | 1102.68 |  | 0.930 |  | 0.9862 |  | | esta | 1102.68 |  | 0.982 |  | 0.9944 |  | | vez | 1079.86 |  | 1.046 |  | 0.9982 |  | | porque | 1036.95 |  | 0.847 |  | 0.9944 |  | | fora | 1016.75 |  | 0.898 |  | 0.9925 |  | | mal | 1010.19 |  | 0.969 |  | 0.9973 |  | | quando | 1010.19 |  | 0.871 |  | 0.9970 |  | | sob | 991.01 |  | 0.812 |  | 0.9957 |  | | este | 984.78 |  | 1.037 |  | 0.9960 |  | | dias | 966.54 |  | 0.818 |  | 0.9895 |  | | pouco | 960.61 |  | 0.561 |  | 0.9574 |  | | logo | 937.60 |  | 0.906 |  | 0.9960 |  | | tempo | 921.06 |  | 1.043 |  | 0.9962 |  | | norte | 910.35 |  | 0.805 |  | 0.9793 |  | | dois | 899.89 |  | 0.910 |  | 0.9961 |  | | porem | 879.66 |  | 1.087 |  | 0.9950 |  | | todo | 850.98 |  | 1.083 |  | 0.9965 |  | | terra | 828.47 |  | 0.783 |  | 0.9879 |  | | foi | 802.97 |  | 0.786 |  | 0.9960 |  | | canudos | 790.81 |  | 0.821 |  | 0.9940 |  | | todas | 790.81 |  | 0.748 |  | 0.9927 |  | | numa | 763.80 |  | 0.937 |  | 0.9992 |  | | ainda | 718.26 |  | 0.966 |  | 0.9978 |  | | toda | 714.98 |  | 0.939 |  | 0.9960 |  | | mesmo | 677.84 |  | 0.869 |  | 0.9959 |  | | dia | 639.10 |  | 0.561 |  | 0.9963 |  | | lhes | 631.37 |  | 0.711 |  | 0.9968 |  | | sua | 602.23 |  | 0.857 |  | 0.9946 |  | | depois | 595.36 |  | 0.958 |  | 0.9986 |  | | onde | 593.11 |  | 0.948 |  | 0.9983 |  | | pelas | 593.11 |  | 0.765 |  | 0.9943 |  | | aos | 582.08 |  | 1.018 |  | 0.9971 |  | | ali | 579.93 |  | 1.066 |  | 0.9936 |  | | todos | 579.93 |  | 0.851 |  | 0.9961 |  | | num | 575.66 |  | 0.812 |  | 0.9983 |  | | e' | 565.27 |  | 0.568 |  | 0.9946 |  | | mas | 539.93 |  | 0.995 |  | 0.9973 |  | | ate | 528.99 |  | 0.932 |  | 0.9986 |  | | pelos | 528.99 |  | 0.870 |  | 0.9986 |  | | `as | 527.21 |  | 0.857 |  | 0.9991 |  | | pela | 475.93 |  | 0.946 |  | 0.9990 |  | | entre | 455.17 |  | 0.926 |  | 0.9981 |  | | pelo | 442.32 |  | 0.861 |  | 0.9978 |  | | nas | 438.60 |  | 0.879 |  | 0.9956 |  | | lhe | 374.59 |  | 0.708 |  | 0.9984 |  | | era | 355.06 |  | 0.746 |  | 0.9937 |  | | nos | 354.25 |  | 0.913 |  | 0.9994 |  | | sobre | 346.42 |  | 0.814 |  | 0.9994 |  | | sem | 322.18 |  | 0.786 |  | 0.9953 |  | | ou | 298.82 |  | 0.738 |  | 0.9964 |  | | como | 284.17 |  | 1.012 |  | 0.9986 |  | | com | 197.45 |  | 0.876 |  | 0.9983 |  | | mais | 183.56 |  | 0.899 |  | 0.9993 |  | | por | 182.49 |  | 0.933 |  | 0.9989 |  | | nao | 171.50 |  | 0.840 |  | 0.9994 |  | | `a | 169.83 |  | 0.942 |  | 0.9992 |  | | na | 168.37 |  | 0.944 |  | 0.9995 |  | | ao | 164.82 |  | 0.961 |  | 0.9987 |  | | no | 146.20 |  | 0.953 |  | 0.9995 |  | | para | 145.79 |  | 0.870 |  | 0.9993 |  | | das | 143.13 |  | 0.897 |  | 0.9992 |  | | uma | 139.43 |  | 0.941 |  | 0.9996 |  | | dos | 123.49 |  | 0.923 |  | 0.9992 |  | | um | 93.76 |  | 0.920 |  | 0.9993 |  | | as | 84.00 |  | 0.835 |  | 0.9973 |  | | da | 80.59 |  | 0.897 |  | 0.9976 |  | | do | 68.56 |  | 0.860 |  | 0.9976 |  | | em | 67.43 |  | 0.909 |  | 0.9956 |  | | os | 60.43 |  | 0.853 |  | 0.9965 |  | | que | 57.00 |  | 0.957 |  | 0.9908 |  | | se | 46.07 |  | 0.915 |  | 0.9928 |  | | e | 33.26 |  | 0.945 |  | 0.9817 |  | | o | 32.81 |  | 0.902 |  | 0.9954 |  | | a | 28.05 |  | 0.926 |  | 0.9915 |  | | de | 23.97 |  | 0.870 |  | 0.9744 |  | |
