# Supplementary material for: Beyond Word Frequency: Bursts, Lulls, and Scaling in the Temporal Distributions of Words
Source: PLoS One. 2009 Nov 11;4(11):e7678. doi: 10.1371/journal.pone.0007678 (PMC2770836; doi:10.1371/journal.pone.0007678)
Supplement: Table S1 — Detailed information on the statistical analysis of all words that were studied (six databases). (31.88 MB TAR) [file pone.0007678.s002.tar › recurrence/talk.origins/index.html]

Word Class | < tau > | ------ | beta | ----- | R^2 | ----- || strata | 2 | 23014.43 |  | 0.265 |  | 0.9866 |  | | ii | U | 23058.23 |  | 0.440 |  | 0.9856 |  | | gary | 1 | 22975.39 |  | 0.239 |  | 0.9614 |  | | incredible | 3 | 23012.13 |  | 0.469 |  | 0.9968 |  | | nazi | 1 | 23440.93 |  | 0.307 |  | 0.9875 |  | | absurd | 3 | 23002.94 |  | 0.513 |  | 0.9858 |  | | fixed | 2 | 22929.62 |  | 0.427 |  | 0.9773 |  | | fraud | 2 | 22957.06 |  | 0.325 |  | 0.9816 |  | | implications | 2 | 23007.53 |  | 0.525 |  | 0.9875 |  | | sc | U | 22890.86 |  | 0.286 |  | 0.9413 |  | | supposedly | 4 | 22957.06 |  | 0.628 |  | 0.9913 |  | | validity | 2 | 22929.62 |  | 0.555 |  | 0.9895 |  | | environmental | 3 | 22952.48 |  | 0.392 |  | 0.9882 |  | | attacks | 3 | 22843.16 |  | 0.463 |  | 0.9924 |  | | samples | 2 | 22815.99 |  | 0.238 |  | 0.9668 |  | | largely | 4 | 22845.43 |  | 0.663 |  | 0.9940 |  | | enter | 2 | 22927.34 |  | 0.414 |  | 0.9854 |  | | testament | 2 | 22911.36 |  | 0.346 |  | 0.9926 |  | | joke | 2 | 22825.04 |  | 0.506 |  | 0.9790 |  | | insects | 2 | 22786.64 |  | 0.291 |  | 0.9772 |  | | internal | 3 | 22791.15 |  | 0.430 |  | 0.9760 |  | | activities | 2 | 22811.47 |  | 0.448 |  | 0.9907 |  | | acid | 2 | 22797.92 |  | 0.284 |  | 0.9831 |  | | fundamentalists | 2 | 22759.60 |  | 0.424 |  | 0.9857 |  | | holden | 1 | 22734.88 |  | 0.254 |  | 0.9234 |  | | oil | 2 | 22714.69 |  | 0.249 |  | 0.9634 |  | | guardian | 2 | 22809.21 |  | 0.116 |  | 0.6344 |  | | earle | 1 | 22712.45 |  | 0.284 |  | 0.9872 |  | | threads | 2 | 22645.42 |  | 0.467 |  | 0.9862 |  | | door | 2 | 22669.95 |  | 0.414 |  | 0.9711 |  | | strawman | 2 | 22640.97 |  | 0.422 |  | 0.9881 |  | | pieret | 1 | 22737.12 |  | 0.454 |  | 0.9943 |  | | brains | 2 | 22600.96 |  | 0.401 |  | 0.9792 |  | | evolving | 3 | 22563.30 |  | 0.477 |  | 0.9939 |  | | flow | 2 | 22574.37 |  | 0.336 |  | 0.9774 |  | | monkeys | 2 | 22550.04 |  | 0.269 |  | 0.9708 |  | | condition | 2 | 22800.18 |  | 0.488 |  | 0.9779 |  | | accounts | 2 | 22541.21 |  | 0.454 |  | 0.9776 |  | | pretend | 4 | 22530.18 |  | 0.624 |  | 0.9931 |  | | expert | 2 | 22578.79 |  | 0.493 |  | 0.9838 |  | | trial | 2 | 22616.50 |  | 0.346 |  | 0.9832 |  | | confusion | 2 | 22536.80 |  | 0.568 |  | 0.9898 |  | | category | 2 | 22545.63 |  | 0.434 |  | 0.9670 |  | | additional | 3 | 22505.95 |  | 0.587 |  | 0.9888 |  | | politics | 2 | 22552.25 |  | 0.413 |  | 0.9923 |  | | horse | 2 | 22503.75 |  | 0.342 |  | 0.9615 |  | | imagination | 2 | 22466.42 |  | 0.470 |  | 0.9789 |  | | lets | U | 22462.03 |  | 0.595 |  | 0.9904 |  | | morning | 2 | 22514.76 |  | 0.491 |  | 0.9908 |  | | depending | 3 | 22468.61 |  | 0.704 |  | 0.9954 |  | | davey | 1 | 22416.10 |  | 0.153 |  | 0.6371 |  | | crime | 2 | 22427.02 |  | 0.320 |  | 0.9877 |  | | statistical | 2 | 22435.76 |  | 0.351 |  | 0.9874 |  | | man's | U | 22407.38 |  | 0.403 |  | 0.9812 |  | | huh | U | 22400.83 |  | 0.601 |  | 0.9864 |  | | myths | 2 | 22385.59 |  | 0.342 |  | 0.9816 |  | | satan | 1 | 22379.06 |  | 0.301 |  | 0.9851 |  | | glad | 3 | 22374.71 |  | 0.669 |  | 0.9938 |  | | arbitrary | 3 | 22344.31 |  | 0.417 |  | 0.9867 |  | | shit | U | 22379.06 |  | 0.471 |  | 0.9868 |  | | editor | 2 | 22355.15 |  | 0.380 |  | 0.9904 |  | | layer | 2 | 22324.80 |  | 0.275 |  | 0.9857 |  | | puns | 2 | 22322.64 |  | 0.351 |  | 0.9739 |  | | everywhere | 3 | 22329.13 |  | 0.454 |  | 0.9817 |  | | iron | 2 | 22305.34 |  | 0.247 |  | 0.9672 |  | | believer | 2 | 22281.59 |  | 0.440 |  | 0.9984 |  | | humanity | 2 | 22322.64 |  | 0.451 |  | 0.9895 |  | | falsified | 2 | 22352.98 |  | 0.362 |  | 0.9906 |  | | uh | U | 22275.12 |  | 0.508 |  | 0.9828 |  | | extra | 3 | 22232.10 |  | 0.472 |  | 0.9847 |  | | satire | 2 | 22187.10 |  | 0.351 |  | 0.9722 |  | | grasp | 2 | 22214.94 |  | 0.554 |  | 0.9952 |  | | miracle | 2 | 22202.08 |  | 0.307 |  | 0.9752 |  | | variations | 2 | 22316.15 |  | 0.415 |  | 0.9809 |  | | yep | U | 22193.52 |  | 0.631 |  | 0.9945 |  | | river | 2 | 22152.94 |  | 0.300 |  | 0.9801 |  | | worship | 2 | 22195.66 |  | 0.394 |  | 0.9899 |  | | spaceman | 2 | 22104.02 |  | 0.237 |  | 0.9497 |  | | slow | 3 | 22116.76 |  | 0.524 |  | 0.9882 |  | | minimum | 2 | 22095.53 |  | 0.281 |  | 0.9600 |  | | producing | 3 | 22099.78 |  | 0.565 |  | 0.9942 |  | | region | 2 | 22095.53 |  | 0.381 |  | 0.9852 |  | | centuries | 2 | 22093.41 |  | 0.577 |  | 0.9951 |  | | fred | 1 | 22068.00 |  | 0.201 |  | 0.9317 |  | | vicious | 3 | 22038.42 |  | 0.357 |  | 0.9750 |  | | djt | U | 22027.88 |  | 0.414 |  | 0.9894 |  | | pool | 2 | 22023.67 |  | 0.366 |  | 0.9887 |  | | impact | 2 | 22015.24 |  | 0.394 |  | 0.9681 |  | | received | 2 | 22040.53 |  | 0.518 |  | 0.9796 |  | | notes | 2 | 22055.31 |  | 0.471 |  | 0.9883 |  | | usage | 2 | 21975.31 |  | 0.361 |  | 0.9791 |  | | alleles | 2 | 21948.08 |  | 0.223 |  | 0.9762 |  | | passage | 2 | 22032.10 |  | 0.401 |  | 0.9828 |  | | listen | 3 | 21971.12 |  | 0.562 |  | 0.9885 |  | | listed | 2 | 21931.35 |  | 0.518 |  | 0.9882 |  | | countries | 2 | 21912.56 |  | 0.365 |  | 0.9945 |  | | wood | 2 | 21945.98 |  | 0.282 |  | 0.9856 |  | | letters | 2 | 21902.14 |  | 0.395 |  | 0.9721 |  | | tv | 2 | 21885.48 |  | 0.459 |  | 0.9826 |  | | careful | 3 | 21835.66 |  | 0.686 |  | 0.9944 |  | | carefully | 4 | 21829.45 |  | 0.628 |  | 0.9892 |  | | intermediate | 3 | 21800.51 |  | 0.338 |  | 0.9892 |  | | rejected | 3 | 21825.31 |  | 0.507 |  | 0.9873 |  | | color | 2 | 21806.70 |  | 0.347 |  | 0.9641 |  | | greek | 1 | 21806.70 |  | 0.343 |  | 0.9810 |  | | miles | 2 | 21821.17 |  | 0.349 |  | 0.9726 |  | | repeatedly | 3 | 21802.57 |  | 0.606 |  | 0.9947 |  | | ahead | 3 | 21796.38 |  | 0.639 |  | 0.9910 |  | | iirc | 4 | 21740.80 |  | 0.643 |  | 0.9967 |  | | german | 2 | 22034.21 |  | 0.322 |  | 0.9878 |  | | round | 2 | 21728.49 |  | 0.452 |  | 0.9725 |  | | discussions | 2 | 21759.30 |  | 0.522 |  | 0.9889 |  | | caught | 3 | 21720.29 |  | 0.585 |  | 0.9912 |  | | hall | 2 | 21644.73 |  | 0.450 |  | 0.9988 |  | | murder | 2 | 21742.86 |  | 0.331 |  | 0.9920 |  | | active | 3 | 21630.50 |  | 0.483 |  | 0.9859 |  | | responding | 3 | 21656.95 |  | 0.595 |  | 0.9905 |  | | organic | 2 | 21604.11 |  | 0.340 |  | 0.9796 |  | | carbon | 2 | 21545.47 |  | 0.257 |  | 0.9708 |  | | ye | 1 | 21547.49 |  | 0.297 |  | 0.9935 |  | | entity | 2 | 21589.93 |  | 0.388 |  | 0.9796 |  | | mars | 1 | 21529.35 |  | 0.241 |  | 0.9658 |  | | nested | 3 | 21529.35 |  | 0.243 |  | 0.9815 |  | | sapiens | U | 21533.38 |  | 0.282 |  | 0.9694 |  | | wall | 2 | 21523.31 |  | 0.347 |  | 0.9679 |  | | plausible | 3 | 21489.16 |  | 0.493 |  | 0.9949 |  | | faster | 3 | 21491.17 |  | 0.453 |  | 0.9862 |  | | charge | 2 | 21487.15 |  | 0.427 |  | 0.9792 |  | | hole | 2 | 21415.20 |  | 0.362 |  | 0.9768 |  | | aids | 2 | 21349.66 |  | 0.170 |  | 0.8924 |  | | pdf | U | 21387.34 |  | 0.274 |  | 0.9726 |  | | distinguish | 3 | 21318.03 |  | 0.565 |  | 0.9920 |  | | welcome | U | 21286.49 |  | 0.599 |  | 0.9888 |  | | quality | 2 | 21361.55 |  | 0.516 |  | 0.9807 |  | | status | 2 | 21172.94 |  | 0.483 |  | 0.9827 |  | | release | 2 | 21174.89 |  | 0.421 |  | 0.9980 |  | | mistaken | 3 | 21190.48 |  | 0.634 |  | 0.9923 |  | | western | 2 | 21139.89 |  | 0.462 |  | 0.9896 |  | | decisions | 2 | 21425.16 |  | 0.338 |  | 0.9929 |  | | yec | 2 | 21112.75 |  | 0.345 |  | 0.9843 |  | | plain | 3 | 21151.54 |  | 0.613 |  | 0.9901 |  | | jabriol | 1 | 21298.30 |  | 0.220 |  | 0.9008 |  | | teachers | 2 | 21045.19 |  | 0.330 |  | 0.9861 |  | | orbit | 2 | 21037.50 |  | 0.278 |  | 0.9856 |  | | matthew | 1 | 21010.62 |  | 0.304 |  | 0.9933 |  | | increased | 3 | 21024.05 |  | 0.460 |  | 0.9922 |  | | dates | 2 | 20999.12 |  | 0.313 |  | 0.9784 |  | | alan | 1 | 21008.70 |  | 0.365 |  | 0.9931 |  | | foundation | 2 | 21116.62 |  | 0.451 |  | 0.9867 |  | | astronomy | 2 | 20974.25 |  | 0.443 |  | 0.9962 |  | | detailed | 3 | 20964.70 |  | 0.603 |  | 0.9923 |  | | frequently | 4 | 21033.66 |  | 0.589 |  | 0.9905 |  | | premise | 2 | 20978.07 |  | 0.380 |  | 0.9741 |  | | wife | 2 | 21035.58 |  | 0.430 |  | 0.9833 |  | | properly | 4 | 20899.99 |  | 0.659 |  | 0.9918 |  | | modification | 2 | 20896.19 |  | 0.427 |  | 0.9950 |  | | carl | 1 | 20862.11 |  | 0.375 |  | 0.9923 |  | | floor | 2 | 20862.11 |  | 0.319 |  | 0.9802 |  | | stage | 2 | 20862.11 |  | 0.419 |  | 0.9674 |  | | tim | 1 | 20784.88 |  | 0.309 |  | 0.9903 |  | | ridiculous | 3 | 20824.37 |  | 0.596 |  | 0.9908 |  | | covered | 2 | 20783.01 |  | 0.532 |  | 0.9884 |  | | ee | U | 20745.55 |  | 0.201 |  | 0.8031 |  | | month | 2 | 20777.38 |  | 0.471 |  | 0.9840 |  | | witness | 2 | 20813.07 |  | 0.399 |  | 0.9822 |  | | raised | 3 | 20736.21 |  | 0.600 |  | 0.9866 |  | | proves | 3 | 20691.48 |  | 0.590 |  | 0.9939 |  | | radiation | 2 | 20659.91 |  | 0.324 |  | 0.9788 |  | | presumably | 4 | 20671.04 |  | 0.693 |  | 0.9958 |  | | teacher | 2 | 20665.47 |  | 0.350 |  | 0.9823 |  | | provides | 3 | 20671.04 |  | 0.638 |  | 0.9897 |  | | purposes | 2 | 20645.09 |  | 0.595 |  | 0.9871 |  | | sides | 2 | 20641.39 |  | 0.518 |  | 0.9861 |  | | fight | 2 | 20639.54 |  | 0.467 |  | 0.9882 |  | | fits | 2 | 20582.33 |  | 0.575 |  | 0.9917 |  | | eternal | 2 | 20567.62 |  | 0.362 |  | 0.9858 |  | | refers | 3 | 20569.46 |  | 0.558 |  | 0.9854 |  | | measurements | 2 | 20525.44 |  | 0.290 |  | 0.9881 |  | | we'll | U | 20624.74 |  | 0.635 |  | 0.9868 |  | | eac | 2 | 20536.43 |  | 0.291 |  | 0.9851 |  | | match | 2 | 20530.94 |  | 0.504 |  | 0.9809 |  | | fails | 3 | 20525.44 |  | 0.594 |  | 0.9911 |  | | miller | 1 | 20501.68 |  | 0.336 |  | 0.9876 |  | | thats | U | 20505.33 |  | 0.411 |  | 0.9928 |  | | identify | 3 | 20521.78 |  | 0.517 |  | 0.9863 |  | | bias | 2 | 20514.47 |  | 0.391 |  | 0.9808 |  | | arise | 3 | 20641.39 |  | 0.516 |  | 0.9937 |  | | blue | 2 | 20476.15 |  | 0.375 |  | 0.9714 |  | | aa | U | 20437.98 |  | 0.261 |  | 0.9896 |  | | cultural | 3 | 20459.77 |  | 0.385 |  | 0.9861 |  | | throw | 2 | 20448.87 |  | 0.636 |  | 0.9885 |  | | criteria | 2 | 20428.91 |  | 0.418 |  | 0.9824 |  | | comparison | 2 | 20448.87 |  | 0.528 |  | 0.9838 |  | | defense | 2 | 20425.28 |  | 0.466 |  | 0.9880 |  | | british | 2 | 20418.04 |  | 0.340 |  | 0.9814 |  | | refute | 3 | 20419.85 |  | 0.510 |  | 0.9907 |  | | progress | 2 | 20447.05 |  | 0.462 |  | 0.9738 |  | | assert | 3 | 20430.72 |  | 0.530 |  | 0.9920 |  | | meet | 2 | 20419.85 |  | 0.572 |  | 0.9859 |  | | ha | U | 20794.27 |  | 0.177 |  | 0.7511 |  | | justify | 3 | 20407.18 |  | 0.546 |  | 0.9915 |  | | volume | 2 | 20335.07 |  | 0.369 |  | 0.9806 |  | | supreme | 3 | 20363.85 |  | 0.319 |  | 0.9877 |  | | amazing | 3 | 20347.66 |  | 0.641 |  | 0.9909 |  | | stay | 2 | 20354.85 |  | 0.661 |  | 0.9919 |  | | production | 2 | 20488.91 |  | 0.437 |  | 0.9946 |  | | killing | 2 | 20387.30 |  | 0.404 |  | 0.9955 |  | | stew | 2 | 20299.21 |  | 0.288 |  | 0.9731 |  | | africa | 1 | 20265.26 |  | 0.338 |  | 0.9846 |  | | dinosaur | 2 | 20217.21 |  | 0.301 |  | 0.9830 |  | | kept | 3 | 20258.13 |  | 0.638 |  | 0.9894 |  | | frequency | 2 | 20215.44 |  | 0.324 |  | 0.9831 |  | | hebrew | 1 | 20192.38 |  | 0.266 |  | 0.9832 |  | | whatsoever | 3 | 20211.89 |  | 0.669 |  | 0.9948 |  | | decades | 2 | 20144.68 |  | 0.507 |  | 0.9880 |  | | originally | 4 | 20218.99 |  | 0.671 |  | 0.9938 |  | | dealing | 3 | 20134.11 |  | 0.670 |  | 0.9926 |  | | minutes | 2 | 20105.97 |  | 0.426 |  | 0.9744 |  | | vague | 3 | 20076.16 |  | 0.528 |  | 0.9893 |  | | hi | U | 20022.04 |  | 0.462 |  | 0.9940 |  | | curious | 3 | 20022.04 |  | 0.672 |  | 0.9941 |  | | successful | 3 | 20011.59 |  | 0.531 |  | 0.9856 |  | | don | 1 | 19992.48 |  | 0.300 |  | 0.9858 |  | | green | 2 | 20037.72 |  | 0.378 |  | 0.9766 |  | | bodies | 2 | 20002.90 |  | 0.454 |  | 0.9812 |  | | scsi | 2 | 19949.17 |  | 0.242 |  | 0.9309 |  | | leaves | 2 | 19983.80 |  | 0.553 |  | 0.9815 |  | | connection | 2 | 19985.53 |  | 0.546 |  | 0.9865 |  | | gaps | 2 | 19947.44 |  | 0.325 |  | 0.9853 |  | | ratio | 2 | 19954.35 |  | 0.254 |  | 0.9724 |  | | criticism | 2 | 19942.25 |  | 0.470 |  | 0.9866 |  | | st | U | 19892.28 |  | 0.362 |  | 0.9829 |  | | aside | 3 | 19849.40 |  | 0.747 |  | 0.9956 |  | | wild | 3 | 19803.30 |  | 0.451 |  | 0.9779 |  | | feeling | 2 | 19801.60 |  | 0.494 |  | 0.9772 |  | | conspiracy | 2 | 19796.49 |  | 0.408 |  | 0.9847 |  | | aspects | 2 | 19777.78 |  | 0.595 |  | 0.9908 |  | | speculation | 2 | 19738.78 |  | 0.444 |  | 0.9892 |  | | relativity | 2 | 19725.25 |  | 0.321 |  | 0.9849 |  | | subjective | 3 | 19723.56 |  | 0.336 |  | 0.9862 |  | | distinct | 3 | 19703.30 |  | 0.536 |  | 0.9888 |  | | minor | 3 | 19686.45 |  | 0.585 |  | 0.9849 |  | | naturalistic | 3 | 19679.72 |  | 0.311 |  | 0.9885 |  | | path | 2 | 19693.19 |  | 0.446 |  | 0.9731 |  | | evolvethought | 1 | 19683.09 |  | 0.449 |  | 0.9927 |  | | prevent | 3 | 19688.14 |  | 0.616 |  | 0.9927 |  | | benefit | 2 | 19693.19 |  | 0.536 |  | 0.9854 |  | | everybody | 3 | 19686.45 |  | 0.567 |  | 0.9917 |  | | venus | 1 | 19636.08 |  | 0.159 |  | 0.8498 |  | | central | 3 | 19654.52 |  | 0.514 |  | 0.9833 |  | | waiting | 3 | 19607.65 |  | 0.608 |  | 0.9904 |  | | incapable | 3 | 19619.34 |  | 0.512 |  | 0.9969 |  | | selected | 3 | 19580.96 |  | 0.453 |  | 0.9898 |  | | url | U | 19592.63 |  | 0.432 |  | 0.9979 |  | | extreme | 3 | 19577.63 |  | 0.590 |  | 0.9893 |  | | seemed | 4 | 19564.32 |  | 0.668 |  | 0.9905 |  | | art | 2 | 19519.53 |  | 0.375 |  | 0.9741 |  | | growth | 2 | 19501.34 |  | 0.349 |  | 0.9759 |  | | synthesis | 2 | 19501.34 |  | 0.349 |  | 0.9937 |  | | library | 2 | 19488.14 |  | 0.426 |  | 0.9758 |  | | creating | 3 | 19468.36 |  | 0.608 |  | 0.9938 |  | | west | 2 | 19486.49 |  | 0.366 |  | 0.9867 |  | | harter | 1 | 19443.70 |  | 0.295 |  | 0.9839 |  | | policy | 2 | 19460.13 |  | 0.389 |  | 0.9962 |  | | creature | 2 | 19446.98 |  | 0.407 |  | 0.9855 |  | | smart | 3 | 19414.18 |  | 0.460 |  | 0.9885 |  | | toe | 2 | 19460.13 |  | 0.301 |  | 0.9903 |  | | sean | 1 | 19414.18 |  | 0.228 |  | 0.8476 |  | | weeks | 2 | 19397.82 |  | 0.506 |  | 0.9855 |  | | sorts | 2 | 19404.36 |  | 0.693 |  | 0.9936 |  | | acts | 2 | 19407.64 |  | 0.467 |  | 0.9865 |  | | parody | 2 | 19350.54 |  | 0.347 |  | 0.9826 |  | | consequences | 2 | 19427.29 |  | 0.436 |  | 0.9775 |  | | spent | 2 | 19383.13 |  | 0.604 |  | 0.9908 |  | | studied | 3 | 19357.05 |  | 0.590 |  | 0.9895 |  | | scenario | 2 | 19365.19 |  | 0.433 |  | 0.9887 |  | | translation | 2 | 19360.30 |  | 0.389 |  | 0.9827 |  | | mightymartia | 1 | 19501.34 |  | 0.412 |  | 0.9982 |  | | useless | 3 | 19316.44 |  | 0.562 |  | 0.9900 |  | | legs | 2 | 19303.49 |  | 0.312 |  | 0.9691 |  | | degrees | 2 | 19253.44 |  | 0.383 |  | 0.9721 |  | | cited | 3 | 19321.31 |  | 0.542 |  | 0.9925 |  | | spend | 2 | 19250.22 |  | 0.602 |  | 0.9882 |  | | constitution | 2 | 19248.61 |  | 0.265 |  | 0.9786 |  | | fallacy | 2 | 19240.57 |  | 0.396 |  | 0.9828 |  | | attacked | 3 | 19214.87 |  | 0.491 |  | 0.9991 |  | | mitchell | 1 | 19376.60 |  | 0.263 |  | 0.9822 |  | | turns | 2 | 19214.87 |  | 0.679 |  | 0.9908 |  | | stick | 2 | 19210.06 |  | 0.643 |  | 0.9904 |  | | walk | 2 | 19240.57 |  | 0.435 |  | 0.9830 |  | | subduction | 2 | 19179.65 |  | 0.190 |  | 0.7574 |  | | la | U | 25531.04 |  | 0.241 |  | 0.9238 |  | | putting | 2 | 19187.65 |  | 0.742 |  | 0.9949 |  | | boy | 2 | 19160.50 |  | 0.472 |  | 0.9787 |  | | affect | 3 | 19152.53 |  | 0.545 |  | 0.9880 |  | | powerful | 3 | 19136.61 |  | 0.549 |  | 0.9883 |  | | applies | 3 | 19112.78 |  | 0.595 |  | 0.9892 |  | | assumed | 3 | 19065.29 |  | 0.595 |  | 0.9887 |  | | william | 1 | 19154.12 |  | 0.446 |  | 0.9923 |  | | clue | 2 | 19033.76 |  | 0.619 |  | 0.9916 |  | | primary | 3 | 18983.54 |  | 0.509 |  | 0.9807 |  | | private | 3 | 19033.76 |  | 0.382 |  | 0.9819 |  | | concerning | 3 | 18992.94 |  | 0.558 |  | 0.9933 |  | | naturally | 4 | 18997.64 |  | 0.568 |  | 0.9898 |  | | forced | 3 | 18980.41 |  | 0.568 |  | 0.9896 |  | | nuclear | 3 | 18944.48 |  | 0.341 |  | 0.9796 |  | | conscious | 3 | 18972.59 |  | 0.214 |  | 0.9490 |  | | allele | 2 | 18938.25 |  | 0.227 |  | 0.9746 |  | | immediately | 3 | 18919.57 |  | 0.693 |  | 0.9937 |  | | macroevolution | 2 | 18888.53 |  | 0.237 |  | 0.9738 |  | | headers | 2 | 18869.95 |  | 0.122 |  | 0.7358 |  | | conflict | 2 | 18868.40 |  | 0.448 |  | 0.9860 |  | | biohumanities | 2 | 18871.50 |  | 0.476 |  | 0.9951 |  | | suggesting | 3 | 18851.41 |  | 0.640 |  | 0.9933 |  | | east | 2 | 18851.41 |  | 0.386 |  | 0.9906 |  | | return | 2 | 18873.04 |  | 0.549 |  | 0.9836 |  | | particles | 2 | 18788.33 |  | 0.278 |  | 0.9816 |  | | authors | 2 | 18837.52 |  | 0.448 |  | 0.9842 |  | | party | 2 | 18856.04 |  | 0.360 |  | 0.9778 |  | | met | 2 | 18803.67 |  | 0.536 |  | 0.9882 |  | | andy | 1 | 18750.07 |  | 0.264 |  | 0.9615 |  | | propose | 3 | 18762.30 |  | 0.565 |  | 0.9944 |  | | offered | 2 | 18760.77 |  | 0.563 |  | 0.9895 |  | | lenny | 1 | 18722.62 |  | 0.254 |  | 0.9634 |  | | tiny | 3 | 18737.86 |  | 0.509 |  | 0.9828 |  | | dic | 1 | 18701.33 |  | 0.395 |  | 0.9932 |  | | stanley | 1 | 18743.96 |  | 0.295 |  | 0.9876 |  | | cold | 3 | 18702.85 |  | 0.427 |  | 0.9855 |  | | noticed | 3 | 18701.33 |  | 0.699 |  | 0.9920 |  | | plant | 2 | 18690.70 |  | 0.353 |  | 0.9721 |  | | rna | 2 | 18612.13 |  | 0.207 |  | 0.9713 |  | | poster | 2 | 18601.60 |  | 0.481 |  | 0.9866 |  | | primitive | 3 | 18664.94 |  | 0.431 |  | 0.9874 |  | | doctrine | 2 | 18640.76 |  | 0.398 |  | 0.9896 |  | | carry | 2 | 18600.10 |  | 0.573 |  | 0.9910 |  | | distribution | 2 | 18580.58 |  | 0.402 |  | 0.9829 |  | | fitness | 2 | 18687.67 |  | 0.227 |  | 0.9757 |  | | behaviour | 2 | 18571.59 |  | 0.290 |  | 0.9642 |  | | speech | 2 | 18592.58 |  | 0.331 |  | 0.9834 |  | | spread | 2 | 18546.15 |  | 0.521 |  | 0.9840 |  | | noah | 1 | 18523.77 |  | 0.258 |  | 0.9740 |  | | indicates | 3 | 18550.63 |  | 0.639 |  | 0.9932 |  | | limits | 2 | 18526.75 |  | 0.455 |  | 0.9815 |  | | checked | 3 | 18485.10 |  | 0.509 |  | 0.9963 |  | | closed | 3 | 18456.94 |  | 0.341 |  | 0.9744 |  | | usa | 1 | 18519.30 |  | 0.391 |  | 0.9887 |  | | forward | 3 | 18436.24 |  | 0.606 |  | 0.9903 |  | | win | 2 | 18443.63 |  | 0.489 |  | 0.9834 |  | | insist | 3 | 18442.15 |  | 0.615 |  | 0.9929 |  | | intent | 2 | 18425.91 |  | 0.370 |  | 0.9697 |  | | europe | 1 | 18452.50 |  | 0.403 |  | 0.9931 |  | | hit | 2 | 18431.82 |  | 0.561 |  | 0.9832 |  | | dictionary | 2 | 18442.15 |  | 0.361 |  | 0.9845 |  | | box | 2 | 18348.08 |  | 0.393 |  | 0.9775 |  | | surprised | 3 | 18368.58 |  | 0.685 |  | 0.9921 |  | | female | 2 | 18374.44 |  | 0.312 |  | 0.9736 |  | | cross | 2 | 18349.54 |  | 0.440 |  | 0.9776 |  | | thoughts | 2 | 18275.25 |  | 0.472 |  | 0.9840 |  | | leading | 3 | 18308.68 |  | 0.635 |  | 0.9910 |  | | ignoring | 3 | 18269.45 |  | 0.643 |  | 0.9931 |  | | ape | 2 | 18241.95 |  | 0.285 |  | 0.9845 |  | | advanced | 3 | 18218.85 |  | 0.522 |  | 0.9897 |  | | dan | 1 | 18190.06 |  | 0.321 |  | 0.9913 |  | | bear | 2 | 18192.93 |  | 0.440 |  | 0.9773 |  | | howard | 1 | 18097.11 |  | 0.294 |  | 0.9824 |  | | draw | 2 | 18077.22 |  | 0.588 |  | 0.9877 |  | | hours | 2 | 18077.22 |  | 0.431 |  | 0.9765 |  | | removed | 2 | 18091.42 |  | 0.541 |  | 0.9870 |  | | discussed | 3 | 18063.03 |  | 0.650 |  | 0.9940 |  | | americans | 2 | 18051.70 |  | 0.378 |  | 0.9946 |  | | elsewhere | 3 | 18033.32 |  | 0.672 |  | 0.9955 |  | | contain | 2 | 18024.85 |  | 0.560 |  | 0.9908 |  | | york | 1 | 18101.38 |  | 0.442 |  | 0.9839 |  | | loss | 2 | 18038.97 |  | 0.422 |  | 0.9783 |  | | surprise | 2 | 18029.08 |  | 0.544 |  | 0.9905 |  | | users | 2 | 17986.83 |  | 0.360 |  | 0.9890 |  | | refuse | 3 | 18029.08 |  | 0.547 |  | 0.9902 |  | | proved | 3 | 17989.64 |  | 0.538 |  | 0.9943 |  | | hypotheses | 2 | 17975.59 |  | 0.393 |  | 0.9873 |  | | scripture | 2 | 17988.23 |  | 0.350 |  | 0.9929 |  | | expanding | 3 | 17937.77 |  | 0.248 |  | 0.9453 |  | | tectonics | 2 | 17926.60 |  | 0.334 |  | 0.9920 |  | | lose | 3 | 17953.16 |  | 0.586 |  | 0.9867 |  | | presence | 2 | 17936.38 |  | 0.546 |  | 0.9881 |  | | convinced | 3 | 17951.76 |  | 0.618 |  | 0.9875 |  | | solid | 2 | 18070.12 |  | 0.499 |  | 0.9902 |  | | cat | 2 | 17905.68 |  | 0.337 |  | 0.9774 |  | | factors | 2 | 17922.41 |  | 0.454 |  | 0.9875 |  | | core | 2 | 17858.46 |  | 0.348 |  | 0.9790 |  | | smith | 1 | 17908.47 |  | 0.362 |  | 0.9912 |  | | followed | 2 | 17864.00 |  | 0.647 |  | 0.9896 |  | | associated | 3 | 17862.61 |  | 0.547 |  | 0.9869 |  | | final | 3 | 17895.94 |  | 0.566 |  | 0.9859 |  | | disease | 2 | 17833.56 |  | 0.363 |  | 0.9784 |  | | universal | 3 | 17841.85 |  | 0.456 |  | 0.9832 |  | | fields | 2 | 17803.22 |  | 0.489 |  | 0.9915 |  | | compare | 3 | 17919.62 |  | 0.607 |  | 0.9900 |  | | distinction | 2 | 17789.46 |  | 0.511 |  | 0.9910 |  | | playing | 3 | 17786.71 |  | 0.610 |  | 0.9905 |  | | walter | 1 | 17751.05 |  | 0.383 |  | 0.9924 |  | | iraq | 1 | 17771.61 |  | 0.213 |  | 0.8943 |  | | rare | 3 | 17755.16 |  | 0.524 |  | 0.9884 |  | | mere | 3 | 17772.98 |  | 0.653 |  | 0.9943 |  | | odd | 3 | 17746.95 |  | 0.656 |  | 0.9903 |  | | represent | 3 | 17749.68 |  | 0.565 |  | 0.9905 |  | | fool | 2 | 17738.74 |  | 0.512 |  | 0.9865 |  | | naturalism | 2 | 17699.19 |  | 0.231 |  | 0.9686 |  | | passed | 2 | 17753.79 |  | 0.574 |  | 0.9883 |  | | mother | 2 | 17794.96 |  | 0.424 |  | 0.9829 |  | | measured | 3 | 17669.31 |  | 0.429 |  | 0.9944 |  | | equivalent | 3 | 17681.52 |  | 0.594 |  | 0.9909 |  | | bbc | 1 | 17636.82 |  | 0.181 |  | 0.9334 |  | | allows | 4 | 17639.52 |  | 0.662 |  | 0.9947 |  | | teeth | 2 | 17626.01 |  | 0.298 |  | 0.9632 |  | | bottom | 2 | 17605.80 |  | 0.501 |  | 0.9850 |  | | easier | 3 | 17591.00 |  | 0.682 |  | 0.9919 |  | | unable | 3 | 17624.67 |  | 0.665 |  | 0.9928 |  | | previously | 3 | 17582.94 |  | 0.590 |  | 0.9920 |  | | grow | 3 | 17595.03 |  | 0.532 |  | 0.9822 |  | | forth | 3 | 17562.81 |  | 0.587 |  | 0.9884 |  | | australia | 1 | 17552.10 |  | 0.397 |  | 0.9942 |  | | fire | 2 | 17513.38 |  | 0.420 |  | 0.9741 |  | | led | 2 | 17534.72 |  | 0.636 |  | 0.9918 |  | | closer | 3 | 17494.75 |  | 0.574 |  | 0.9861 |  | | purely | 4 | 17505.39 |  | 0.558 |  | 0.9911 |  | | ages | 2 | 17473.50 |  | 0.432 |  | 0.9836 |  | | sky | 2 | 17468.20 |  | 0.411 |  | 0.9833 |  | | danger | 2 | 17474.83 |  | 0.500 |  | 0.9995 |  | | testing | 3 | 17469.52 |  | 0.441 |  | 0.9892 |  | | reach | 2 | 17466.87 |  | 0.600 |  | 0.9884 |  | | failure | 2 | 17494.75 |  | 0.535 |  | 0.9844 |  | | cover | 2 | 17460.25 |  | 0.566 |  | 0.9855 |  | | explaining | 3 | 17452.31 |  | 0.670 |  | 0.9950 |  | | impression | 2 | 17437.77 |  | 0.636 |  | 0.9923 |  | | named | 2 | 17406.12 |  | 0.544 |  | 0.9893 |  | | convince | 3 | 17404.81 |  | 0.578 |  | 0.9887 |  | | dogs | 2 | 17371.97 |  | 0.302 |  | 0.9782 |  | | harshman | 1 | 17327.52 |  | 0.352 |  | 0.9653 |  | | dean | 2 | 17343.18 |  | 0.306 |  | 0.9879 |  | | slightly | 4 | 17344.49 |  | 0.618 |  | 0.9880 |  | | prediction | 2 | 17334.04 |  | 0.338 |  | 0.9836 |  | | tradition | 2 | 17353.64 |  | 0.393 |  | 0.9927 |  | | variety | 2 | 17351.02 |  | 0.592 |  | 0.9910 |  | | pz | U | 17293.67 |  | 0.293 |  | 0.9773 |  | | secular | 2 | 17374.59 |  | 0.323 |  | 0.9879 |  | | critical | 3 | 17275.50 |  | 0.500 |  | 0.9807 |  | | car | 2 | 17250.91 |  | 0.360 |  | 0.9698 |  | | responses | 2 | 17235.41 |  | 0.532 |  | 0.9891 |  | | ron | 1 | 17199.36 |  | 0.349 |  | 0.9935 |  | | definitely | 4 | 17200.64 |  | 0.716 |  | 0.9956 |  | | re | 2 | 17296.27 |  | 0.446 |  | 0.9894 |  | | bunch | 2 | 17181.38 |  | 0.683 |  | 0.9932 |  | | chimps | 2 | 17166.01 |  | 0.277 |  | 0.9830 |  | | unlike | 3 | 17187.80 |  | 0.710 |  | 0.9965 |  | | wide | 3 | 17212.22 |  | 0.581 |  | 0.9925 |  | | strongly | 4 | 17123.88 |  | 0.654 |  | 0.9942 |  | | uk | U | 17100.98 |  | 0.338 |  | 0.9874 |  | | deleted | 3 | 17093.36 |  | 0.470 |  | 0.9974 |  | | grand | 3 | 17076.88 |  | 0.333 |  | 0.9728 |  | | apparent | 3 | 17085.75 |  | 0.618 |  | 0.9897 |  | | develop | 3 | 17081.95 |  | 0.543 |  | 0.9901 |  | | limit | 2 | 17051.58 |  | 0.480 |  | 0.9806 |  | | property | 2 | 17069.28 |  | 0.387 |  | 0.9870 |  | | save | 3 | 17074.34 |  | 0.573 |  | 0.9900 |  | | disclaimer | 2 | 17018.81 |  | 0.332 |  | 0.9572 |  | | numerous | 3 | 17033.92 |  | 0.624 |  | 0.9948 |  | | classes | 2 | 17010.00 |  | 0.425 |  | 0.9851 |  | | phenomenon | 2 | 17018.81 |  | 0.465 |  | 0.9852 |  | | mechanics | 2 | 16998.70 |  | 0.390 |  | 0.9895 |  | | acids | 2 | 16976.14 |  | 0.240 |  | 0.9791 |  | | glenn | 1 | 17071.81 |  | 0.227 |  | 0.9181 |  | | shape | 2 | 17042.74 |  | 0.448 |  | 0.9817 |  | | break | 2 | 17141.73 |  | 0.604 |  | 0.9910 |  | | memory | 2 | 16979.89 |  | 0.477 |  | 0.9802 |  | | ought | 4 | 16986.16 |  | 0.557 |  | 0.9895 |  | | stephen | 1 | 16941.16 |  | 0.425 |  | 0.9937 |  | | goal | 2 | 17054.11 |  | 0.457 |  | 0.9795 |  | | background | 2 | 16953.63 |  | 0.514 |  | 0.9874 |  | | billions | 3 | 16923.72 |  | 0.478 |  | 0.9902 |  | | influence | 2 | 16939.91 |  | 0.506 |  | 0.9865 |  | | troll | 2 | 16923.72 |  | 0.360 |  | 0.9886 |  | | friends | 2 | 16923.72 |  | 0.526 |  | 0.9899 |  | | anywhere | 3 | 16892.68 |  | 0.727 |  | 0.9961 |  | | diversity | 2 | 16923.72 |  | 0.415 |  | 0.9870 |  | | machine | 2 | 16900.12 |  | 0.348 |  | 0.9787 |  | | selective | 3 | 16867.92 |  | 0.403 |  | 0.9882 |  | | weak | 3 | 16871.63 |  | 0.513 |  | 0.9859 |  | | weight | 2 | 16822.31 |  | 0.391 |  | 0.9692 |  | | oxygen | 2 | 16758.64 |  | 0.249 |  | 0.9750 |  | | throughout | 2 | 16773.29 |  | 0.596 |  | 0.9921 |  | | florian | 1 | 16707.55 |  | 0.221 |  | 0.7153 |  | | conrad | 1 | 16709.98 |  | 0.220 |  | 0.9435 |  | | identical | 3 | 16680.92 |  | 0.454 |  | 0.9852 |  | | martin | 1 | 16709.98 |  | 0.360 |  | 0.9928 |  | | attempts | 3 | 16673.67 |  | 0.668 |  | 0.9940 |  | | steps | 2 | 16645.94 |  | 0.385 |  | 0.9815 |  | | plus | 2 | 16649.55 |  | 0.541 |  | 0.9792 |  | | pointing | 3 | 16648.35 |  | 0.698 |  | 0.9943 |  | | happening | 3 | 16637.52 |  | 0.556 |  | 0.9925 |  | | ignored | 3 | 16666.43 |  | 0.631 |  | 0.9942 |  | | toward | 2 | 16679.71 |  | 0.573 |  | 0.9874 |  | | crap | 2 | 16624.31 |  | 0.555 |  | 0.9901 |  | | hands | 2 | 16599.13 |  | 0.567 |  | 0.9847 |  | | unique | 3 | 16552.59 |  | 0.474 |  | 0.9850 |  | | einstein | 1 | 16607.52 |  | 0.325 |  | 0.9826 |  | | contains | 2 | 16547.83 |  | 0.567 |  | 0.9891 |  | | extinct | 3 | 16619.50 |  | 0.411 |  | 0.9933 |  | | roger | 1 | 16514.59 |  | 0.223 |  | 0.9300 |  | | discussing | 3 | 16522.89 |  | 0.635 |  | 0.9919 |  | | johnson | 1 | 16527.63 |  | 0.281 |  | 0.9732 |  | | former | 3 | 16519.33 |  | 0.632 |  | 0.9935 |  | | reaction | 2 | 16446.16 |  | 0.419 |  | 0.9720 |  | | considering | 3 | 16425.04 |  | 0.743 |  | 0.9956 |  | | legal | 3 | 16435.59 |  | 0.376 |  | 0.9935 |  | | lagenta | 1 | 16439.12 |  | 0.110 |  | 0.7654 |  | | somebody | 3 | 16425.04 |  | 0.606 |  | 0.9935 |  | | israel | 1 | 16461.45 |  | 0.211 |  | 0.9467 |  | | dembski | 1 | 16384.13 |  | 0.235 |  | 0.9468 |  | | yahoo | 1 | 16349.22 |  | 0.246 |  | 0.9772 |  | | paragraph | 2 | 16415.67 |  | 0.547 |  | 0.9859 |  | | character | 2 | 16364.33 |  | 0.449 |  | 0.9818 |  | | afraid | 3 | 16346.90 |  | 0.631 |  | 0.9910 |  | | correctly | 4 | 16298.29 |  | 0.706 |  | 0.9949 |  | | layers | 2 | 16277.55 |  | 0.257 |  | 0.9832 |  | | conclude | 3 | 16286.76 |  | 0.587 |  | 0.9929 |  | | derived | 3 | 16290.22 |  | 0.519 |  | 0.9884 |  | | suggested | 3 | 16217.92 |  | 0.672 |  | 0.9946 |  | | snipped | 2 | 16214.49 |  | 0.570 |  | 0.9946 |  | | image | 2 | 16256.86 |  | 0.387 |  | 0.9735 |  | | excuse | 2 | 16201.94 |  | 0.614 |  | 0.9934 |  | | tests | 2 | 16142.86 |  | 0.389 |  | 0.9824 |  | | business | 2 | 16111.23 |  | 0.511 |  | 0.9833 |  | | title | 2 | 16080.84 |  | 0.490 |  | 0.9855 |  | | cite | 3 | 16102.22 |  | 0.546 |  | 0.9914 |  | | sites | 2 | 16044.98 |  | 0.400 |  | 0.9792 |  | | fly | 2 | 16077.48 |  | 0.409 |  | 0.9891 |  | | moron | 2 | 16079.72 |  | 0.410 |  | 0.9970 |  | | opposite | 3 | 16048.34 |  | 0.634 |  | 0.9910 |  | | perspective | 2 | 16041.63 |  | 0.501 |  | 0.9823 |  | | tony | 1 | 16021.53 |  | 0.234 |  | 0.9508 |  | | waste | 2 | 15994.82 |  | 0.550 |  | 0.9901 |  | | pip | U | 16031.57 |  | 0.113 |  | 0.7424 |  | | essentially | 4 | 15983.71 |  | 0.688 |  | 0.9952 |  | | gas | 2 | 15965.97 |  | 0.290 |  | 0.9729 |  | | began | 3 | 16007.05 |  | 0.555 |  | 0.9869 |  | | testable | 3 | 15908.60 |  | 0.388 |  | 0.9918 |  | | soul | 2 | 15928.41 |  | 0.325 |  | 0.9799 |  | | principles | 2 | 16022.65 |  | 0.479 |  | 0.9861 |  | | sample | 2 | 15880.07 |  | 0.260 |  | 0.9631 |  | | report | 2 | 16029.34 |  | 0.430 |  | 0.9808 |  | | united | 3 | 15891.03 |  | 0.383 |  | 0.9899 |  | | crust | 2 | 15846.19 |  | 0.198 |  | 0.9085 |  | | appearance | 2 | 15919.60 |  | 0.474 |  | 0.9868 |  | | mathematical | 2 | 15842.91 |  | 0.413 |  | 0.9926 |  | | hundreds | 3 | 15853.82 |  | 0.609 |  | 0.9935 |  | | woman | 2 | 15872.41 |  | 0.362 |  | 0.9854 |  | | fundamentalist | 2 | 15825.49 |  | 0.448 |  | 0.9888 |  | | philosophical | 3 | 15804.85 |  | 0.443 |  | 0.9924 |  | | php | U | 15770.20 |  | 0.232 |  | 0.9520 |  | | website | 2 | 15787.50 |  | 0.423 |  | 0.9903 |  | | odds | 2 | 15763.72 |  | 0.335 |  | 0.9749 |  | | flat | 2 | 15745.39 |  | 0.392 |  | 0.9741 |  | | thermodynamics | 2 | 15728.18 |  | 0.264 |  | 0.9687 |  | | typical | 3 | 15757.25 |  | 0.643 |  | 0.9940 |  | | spirit | 2 | 15781.01 |  | 0.357 |  | 0.9847 |  | | repeat | 3 | 15758.33 |  | 0.655 |  | 0.9933 |  | | extent | 2 | 15729.26 |  | 0.665 |  | 0.9937 |  | | one's | U | 15720.66 |  | 0.558 |  | 0.9887 |  | | leaders | 2 | 15724.96 |  | 0.478 |  | 0.9992 |  | | closely | 4 | 15701.36 |  | 0.548 |  | 0.9898 |  | | quickly | 4 | 15694.94 |  | 0.648 |  | 0.9943 |  | | dogma | 2 | 15720.66 |  | 0.432 |  | 0.9957 |  | | minds | 2 | 15714.22 |  | 0.568 |  | 0.9909 |  | | absence | 2 | 15705.65 |  | 0.482 |  | 0.9871 |  | | agreed | 3 | 15702.43 |  | 0.631 |  | 0.9923 |  | | equally | 4 | 15676.77 |  | 0.645 |  | 0.9908 |  | | factor | 2 | 15657.58 |  | 0.447 |  | 0.9787 |  | | drift | 2 | 15665.04 |  | 0.324 |  | 0.9826 |  | | trust | 2 | 15646.94 |  | 0.489 |  | 0.9852 |  | | reproduce | 3 | 15707.79 |  | 0.406 |  | 0.9911 |  | | popular | 3 | 15602.40 |  | 0.576 |  | 0.9895 |  | | indicate | 3 | 15598.17 |  | 0.664 |  | 0.9942 |  | | meaningless | 3 | 15560.21 |  | 0.528 |  | 0.9920 |  | | fast | 3 | 15482.77 |  | 0.525 |  | 0.9897 |  | | favor | 2 | 15511.99 |  | 0.642 |  | 0.9930 |  | | rise | 2 | 15583.39 |  | 0.525 |  | 0.9869 |  | | darwinian | 3 | 15610.86 |  | 0.365 |  | 0.9935 |  | | familiar | 3 | 15492.15 |  | 0.673 |  | 0.9935 |  | | engineering | 2 | 15461.98 |  | 0.393 |  | 0.9905 |  | | postdoctoral | 2 | 15454.71 |  | 0.441 |  | 0.9938 |  | | prefer | 3 | 15457.82 |  | 0.702 |  | 0.9947 |  | | initial | 3 | 15433.99 |  | 0.472 |  | 0.9846 |  | | negative | 3 | 15431.92 |  | 0.433 |  | 0.9771 |  | | prior | 3 | 15461.98 |  | 0.572 |  | 0.9902 |  | | birth | 2 | 15461.98 |  | 0.416 |  | 0.9811 |  | | friend | 2 | 15442.27 |  | 0.516 |  | 0.9891 |  | | organization | 2 | 15435.02 |  | 0.455 |  | 0.9885 |  | | male | 2 | 15399.92 |  | 0.305 |  | 0.9739 |  | | leads | 2 | 15414.35 |  | 0.658 |  | 0.9940 |  | | patterns | 2 | 15370.10 |  | 0.389 |  | 0.9824 |  | | properties | 2 | 15374.21 |  | 0.379 |  | 0.9837 |  | | apart | 3 | 15389.62 |  | 0.654 |  | 0.9934 |  | | defend | 3 | 15364.97 |  | 0.557 |  | 0.9926 |  | | regardless | 4 | 15366.00 |  | 0.671 |  | 0.9967 |  | | myers | 1 | 15324.06 |  | 0.272 |  | 0.9601 |  | | latter | 3 | 15288.44 |  | 0.741 |  | 0.9974 |  | | front | 2 | 15261.08 |  | 0.507 |  | 0.9824 |  | | damn | U | 15255.01 |  | 0.606 |  | 0.9914 |  | | king | 2 | 15251.98 |  | 0.349 |  | 0.9828 |  | | ediacara | 1 | 15216.69 |  | 0.396 |  | 0.9961 |  | | proper | 3 | 15227.77 |  | 0.612 |  | 0.9906 |  | | showed | 3 | 15211.67 |  | 0.649 |  | 0.9934 |  | | ten | 3 | 15165.57 |  | 0.509 |  | 0.9821 |  | | determined | 3 | 15192.59 |  | 0.560 |  | 0.9898 |  | | icr | 1 | 15144.62 |  | 0.289 |  | 0.9864 |  | | implies | 3 | 15186.58 |  | 0.592 |  | 0.9919 |  | | months | 2 | 15169.57 |  | 0.561 |  | 0.9917 |  | | offspring | 2 | 15124.72 |  | 0.333 |  | 0.9863 |  | | addition | 2 | 15110.82 |  | 0.653 |  | 0.9957 |  | | killed | 2 | 15140.64 |  | 0.460 |  | 0.9964 |  | | access | 2 | 15080.14 |  | 0.452 |  | 0.9850 |  | | learning | 2 | 15087.06 |  | 0.535 |  | 0.9901 |  | | functional | 3 | 15075.20 |  | 0.300 |  | 0.9851 |  | | hundred | 3 | 15075.20 |  | 0.581 |  | 0.9872 |  | | larry | 1 | 15048.59 |  | 0.314 |  | 0.9846 |  | | pure | 3 | 15046.63 |  | 0.543 |  | 0.9862 |  | | chain | 2 | 15008.37 |  | 0.382 |  | 0.9957 |  | | service | 2 | 15063.36 |  | 0.368 |  | 0.9877 |  | | mail | 2 | 15010.33 |  | 0.396 |  | 0.9960 |  | | believing | 3 | 14970.31 |  | 0.552 |  | 0.9951 |  | | functions | 2 | 14967.39 |  | 0.337 |  | 0.9889 |  | | room | 2 | 14969.33 |  | 0.516 |  | 0.9821 |  | | plan | 2 | 14962.52 |  | 0.460 |  | 0.9820 |  | | temperature | 2 | 14863.98 |  | 0.274 |  | 0.9828 |  | | mental | 3 | 14909.23 |  | 0.394 |  | 0.9758 |  | | characteristics | 2 | 14839.07 |  | 0.426 |  | 0.9852 |  | | pages | 2 | 14848.64 |  | 0.451 |  | 0.9865 |  | | jack | 1 | 14815.20 |  | 0.317 |  | 0.9871 |  | | concepts | 2 | 14827.60 |  | 0.522 |  | 0.9897 |  | | decay | 2 | 14801.86 |  | 0.248 |  | 0.9750 |  | | chapter | 2 | 14815.20 |  | 0.414 |  | 0.9823 |  | | reproduction | 2 | 14962.52 |  | 0.340 |  | 0.9883 |  | | beneficial | 3 | 14879.35 |  | 0.256 |  | 0.9809 |  | | atoms | 2 | 14766.73 |  | 0.285 |  | 0.9801 |  | | solution | 2 | 14764.84 |  | 0.448 |  | 0.9851 |  | | supports | 2 | 14754.43 |  | 0.624 |  | 0.9947 |  | | regard | 2 | 14749.70 |  | 0.625 |  | 0.9925 |  | | practice | 2 | 14724.23 |  | 0.567 |  | 0.9908 |  | | positive | 3 | 14701.67 |  | 0.460 |  | 0.9799 |  | | decision | 2 | 14935.34 |  | 0.371 |  | 0.9862 |  | | dont | U | 14678.23 |  | 0.373 |  | 0.9971 |  | | father | 1 | 14727.06 |  | 0.383 |  | 0.9806 |  | | levels | 2 | 14651.14 |  | 0.445 |  | 0.9849 |  | | unlikely | 4 | 14656.74 |  | 0.578 |  | 0.9927 |  | | applied | 3 | 14642.75 |  | 0.584 |  | 0.9921 |  | | building | 2 | 14652.07 |  | 0.502 |  | 0.9874 |  | | bone | 2 | 14719.53 |  | 0.249 |  | 0.9658 |  | | tested | 3 | 14626.94 |  | 0.478 |  | 0.9926 |  | | motion | 2 | 14641.82 |  | 0.326 |  | 0.9866 |  | | smaller | 3 | 14660.47 |  | 0.504 |  | 0.9892 |  | | consciousness | 2 | 14675.42 |  | 0.194 |  | 0.9309 |  | | bird | 2 | 14582.47 |  | 0.303 |  | 0.9822 |  | | includes | 3 | 14581.54 |  | 0.671 |  | 0.9937 |  | | queensland | 1 | 14581.54 |  | 0.397 |  | 0.9871 |  | | suggests | 3 | 14576.00 |  | 0.662 |  | 0.9935 |  | | intended | 3 | 14564.01 |  | 0.623 |  | 0.9921 |  | | six | 3 | 14572.31 |  | 0.466 |  | 0.9804 |  | | decided | 3 | 14583.39 |  | 0.648 |  | 0.9936 |  | | holy | 3 | 14576.93 |  | 0.420 |  | 0.9902 |  | | values | 2 | 14587.09 |  | 0.363 |  | 0.9788 |  | | son | 1 | 14759.16 |  | 0.387 |  | 0.9868 |  | | atmosphere | 2 | 14497.06 |  | 0.258 |  | 0.9753 |  | | turned | 2 | 14535.51 |  | 0.655 |  | 0.9927 |  | | heaven | 1 | 14489.76 |  | 0.375 |  | 0.9901 |  | | okay | U | 14492.50 |  | 0.604 |  | 0.9942 |  | | effort | 2 | 14492.50 |  | 0.662 |  | 0.9913 |  | | star | 2 | 14454.28 |  | 0.332 |  | 0.9747 |  | | scientifically | 4 | 14443.40 |  | 0.543 |  | 0.9958 |  | | strange | 3 | 14433.44 |  | 0.618 |  | 0.9923 |  | | length | 2 | 14404.54 |  | 0.450 |  | 0.9841 |  | | worked | 2 | 14438.87 |  | 0.676 |  | 0.9942 |  | | pick | 2 | 14400.04 |  | 0.649 |  | 0.9894 |  | | traits | 2 | 14382.94 |  | 0.333 |  | 0.9833 |  | | voice | 2 | 14346.19 |  | 0.355 |  | 0.9728 |  | | anybody | 3 | 14328.34 |  | 0.624 |  | 0.9949 |  | | success | 2 | 14367.68 |  | 0.407 |  | 0.9818 |  | | self | 2 | 14295.42 |  | 0.480 |  | 0.9866 |  | | bother | 2 | 14259.11 |  | 0.707 |  | 0.9953 |  | | hate | 2 | 14276.80 |  | 0.463 |  | 0.9882 |  | | shouldn't | 4 | 14264.41 |  | 0.723 |  | 0.9958 |  | | noted | 2 | 14268.83 |  | 0.645 |  | 0.9938 |  | | ac | U | 14267.95 |  | 0.362 |  | 0.9952 |  | | we've | U | 14239.71 |  | 0.661 |  | 0.9927 |  | | idiot | 2 | 14285.66 |  | 0.483 |  | 0.9908 |  | | phrase | 2 | 14250.29 |  | 0.543 |  | 0.9839 |  | | precisely | 4 | 14246.76 |  | 0.678 |  | 0.9979 |  | | clausen | 1 | 14347.98 |  | 0.436 |  | 0.9975 |  | | city | 2 | 14237.95 |  | 0.423 |  | 0.9923 |  | | myth | 2 | 14196.68 |  | 0.381 |  | 0.9873 |  | | weren't | U | 14219.48 |  | 0.719 |  | 0.9968 |  | | journal | 2 | 14207.19 |  | 0.416 |  | 0.9832 |  | | reproductive | 2 | 14275.91 |  | 0.304 |  | 0.9845 |  | | intellectual | 2 | 14222.12 |  | 0.515 |  | 0.9896 |  | | massive | 3 | 14200.18 |  | 0.511 |  | 0.9976 |  | | older | 3 | 14180.94 |  | 0.516 |  | 0.9905 |  | | built | 2 | 14167.85 |  | 0.513 |  | 0.9862 |  | | relatively | 4 | 14164.36 |  | 0.627 |  | 0.9940 |  | | compared | 3 | 14157.39 |  | 0.669 |  | 0.9946 |  | | neutral | 3 | 14204.56 |  | 0.307 |  | 0.9850 |  | | opposed | 3 | 14161.75 |  | 0.705 |  | 0.9960 |  | | lab | 2 | 14116.59 |  | 0.453 |  | 0.9924 |  | | role | 2 | 14156.52 |  | 0.545 |  | 0.9906 |  | | assertions | 2 | 14118.32 |  | 0.505 |  | 0.9913 |  | | charles | 1 | 14125.25 |  | 0.398 |  | 0.9907 |  | | sign | 2 | 14099.30 |  | 0.544 |  | 0.9895 |  | | medical | 2 | 14261.76 |  | 0.419 |  | 0.9951 |  | | pass | 2 | 14088.08 |  | 0.624 |  | 0.9893 |  | | whom | U | 14065.70 |  | 0.648 |  | 0.9954 |  | | sin | 2 | 14190.56 |  | 0.305 |  | 0.9854 |  | | aaron | 1 | 14085.50 |  | 0.372 |  | 0.9890 |  | | predict | 3 | 14038.26 |  | 0.426 |  | 0.9882 |  | | jewish | 2 | 14120.06 |  | 0.310 |  | 0.9881 |  | | running | 3 | 14029.70 |  | 0.578 |  | 0.9927 |  | | infinite | 2 | 14010.06 |  | 0.307 |  | 0.9767 |  | | plate | 2 | 13978.59 |  | 0.237 |  | 0.9507 |  | | changing | 3 | 13981.98 |  | 0.603 |  | 0.9928 |  | | huge | 3 | 13946.41 |  | 0.647 |  | 0.9943 |  | | replace | 3 | 13938.81 |  | 0.481 |  | 0.9965 |  | | elements | 2 | 13927.00 |  | 0.404 |  | 0.9750 |  | | ic | U | 13902.61 |  | 0.175 |  | 0.8864 |  | | occurs | 3 | 13923.63 |  | 0.588 |  | 0.9956 |  | | advantage | 2 | 13914.38 |  | 0.437 |  | 0.9866 |  | | names | 2 | 13918.58 |  | 0.481 |  | 0.9849 |  | | blind | 2 | 13920.27 |  | 0.455 |  | 0.9806 |  | | sexual | 2 | 13919.43 |  | 0.309 |  | 0.9794 |  | | spiritual | 3 | 13927.00 |  | 0.325 |  | 0.9889 |  | | held | 2 | 13912.70 |  | 0.634 |  | 0.9932 |  | | national | 3 | 13963.33 |  | 0.447 |  | 0.9922 |  | | rates | 2 | 13869.11 |  | 0.353 |  | 0.9847 |  | | exact | 3 | 13888.36 |  | 0.631 |  | 0.9912 |  | | recognize | 3 | 13911.86 |  | 0.620 |  | 0.9928 |  | | student | 2 | 13859.09 |  | 0.421 |  | 0.9883 |  | | college | 2 | 13850.75 |  | 0.454 |  | 0.9878 |  | | build | 2 | 13855.76 |  | 0.494 |  | 0.9858 |  | | potential | 3 | 13927.85 |  | 0.496 |  | 0.9865 |  | | expansion | 2 | 13827.46 |  | 0.233 |  | 0.9223 |  | | feet | 2 | 13824.97 |  | 0.374 |  | 0.9765 |  | | nando | 1 | 14125.25 |  | 0.166 |  | 0.6835 |  | | section | 2 | 13758.87 |  | 0.402 |  | 0.9713 |  | | jason | 1 | 13729.33 |  | 0.244 |  | 0.9460 |  | | inside | 2 | 13717.06 |  | 0.498 |  | 0.9895 |  | | included | 3 | 13719.51 |  | 0.655 |  | 0.9932 |  | | missed | 3 | 13679.56 |  | 0.713 |  | 0.9954 |  | | divine | 2 | 13690.14 |  | 0.434 |  | 0.9896 |  | | internet | 2 | 13695.84 |  | 0.479 |  | 0.9954 |  | | technical | 2 | 13652.79 |  | 0.421 |  | 0.9936 |  | | vote | 2 | 13666.57 |  | 0.279 |  | 0.9793 |  | | models | 2 | 13623.71 |  | 0.407 |  | 0.9840 |  | | forum | 2 | 13632.58 |  | 0.405 |  | 0.9895 |  | | concerned | 3 | 13643.89 |  | 0.672 |  | 0.9951 |  | | non | U | 13641.47 |  | 0.535 |  | 0.9905 |  | | unknown | 3 | 13590.73 |  | 0.469 |  | 0.9834 |  | | piece | 2 | 13601.17 |  | 0.602 |  | 0.9881 |  | | range | 2 | 13600.36 |  | 0.514 |  | 0.9846 |  | | remain | 3 | 13593.14 |  | 0.658 |  | 0.9959 |  | | supporting | 3 | 13579.51 |  | 0.618 |  | 0.9945 |  | | heart | 2 | 13593.94 |  | 0.449 |  | 0.9815 |  | | morality | 2 | 13593.94 |  | 0.298 |  | 0.9877 |  | | press | 2 | 13679.56 |  | 0.466 |  | 0.9795 |  | | solar | 2 | 13545.95 |  | 0.332 |  | 0.9881 |  | | appeared | 4 | 13554.72 |  | 0.528 |  | 0.9903 |  | | detail | 2 | 13534.80 |  | 0.646 |  | 0.9941 |  | | generations | 2 | 13531.62 |  | 0.435 |  | 0.9892 |  | | scale | 2 | 13496.71 |  | 0.475 |  | 0.9843 |  | | lived | 2 | 13504.63 |  | 0.544 |  | 0.9931 |  | | gone | U | 13528.43 |  | 0.716 |  | 0.9952 |  | | north | 2 | 13477.75 |  | 0.395 |  | 0.9916 |  | | nope | 4 | 13484.06 |  | 0.570 |  | 0.9932 |  | | starting | 3 | 13488.80 |  | 0.603 |  | 0.9895 |  | | empirical | 3 | 13443.12 |  | 0.361 |  | 0.9904 |  | | fail | 3 | 13475.38 |  | 0.681 |  | 0.9943 |  | | fear | 2 | 13454.12 |  | 0.459 |  | 0.9870 |  | | decide | 3 | 13455.69 |  | 0.602 |  | 0.9946 |  | | actions | 2 | 13495.92 |  | 0.471 |  | 0.9968 |  | | replies | 2 | 13386.06 |  | 0.329 |  | 0.9735 |  | | copy | 3 | 13390.73 |  | 0.494 |  | 0.9820 |  | | knowing | 3 | 13404.76 |  | 0.617 |  | 0.9906 |  | | eat | 2 | 13385.28 |  | 0.414 |  | 0.9834 |  | | standards | 2 | 13384.50 |  | 0.430 |  | 0.9778 |  | | excellent | 3 | 13374.39 |  | 0.643 |  | 0.9954 |  | | existing | 3 | 13348.02 |  | 0.527 |  | 0.9911 |  | | added | 2 | 13340.29 |  | 0.605 |  | 0.9934 |  | | multiple | 3 | 13340.29 |  | 0.538 |  | 0.9892 |  | | night | 2 | 13352.67 |  | 0.499 |  | 0.9898 |  | | bet | 2 | 13338.74 |  | 0.519 |  | 0.9736 |  | | letter | 2 | 13345.70 |  | 0.361 |  | 0.9741 |  | | watch | 2 | 13342.61 |  | 0.524 |  | 0.9865 |  | | expressed | 3 | 13329.48 |  | 0.533 |  | 0.9975 |  | | vast | 3 | 13296.37 |  | 0.607 |  | 0.9912 |  | | rights | 2 | 13304.06 |  | 0.309 |  | 0.9907 |  | | earth's | U | 13257.32 |  | 0.360 |  | 0.9933 |  | | died | 2 | 13287.93 |  | 0.501 |  | 0.9929 |  | | peace | 2 | 13280.26 |  | 0.381 |  | 0.9960 |  | | demonstrated | 3 | 13282.56 |  | 0.630 |  | 0.9962 |  | | sufficient | 3 | 13239.02 |  | 0.622 |  | 0.9945 |  | | mathematics | 2 | 13231.41 |  | 0.396 |  | 0.9947 |  | | planets | 2 | 13229.13 |  | 0.327 |  | 0.9860 |  | | usual | 3 | 13226.85 |  | 0.712 |  | 0.9959 |  | | five | 3 | 13217.74 |  | 0.522 |  | 0.9810 |  | | phenomena | 2 | 13218.50 |  | 0.426 |  | 0.9893 |  | | au | U | 13195.77 |  | 0.296 |  | 0.9714 |  | | objects | 2 | 13185.94 |  | 0.387 |  | 0.9827 |  | | papers | 2 | 13169.35 |  | 0.430 |  | 0.9891 |  | | austin | 1 | 13162.57 |  | 0.389 |  | 0.9942 |  | | wait | 2 | 13167.84 |  | 0.680 |  | 0.9935 |  | | hot | 3 | 13158.06 |  | 0.453 |  | 0.9915 |  | | theology | 2 | 13136.28 |  | 0.419 |  | 0.9940 |  | | geological | 2 | 13122.81 |  | 0.404 |  | 0.9969 |  | | mistake | 2 | 13140.78 |  | 0.605 |  | 0.9896 |  | | somewhat | 3 | 13127.30 |  | 0.761 |  | 0.9972 |  | | center | 2 | 13128.04 |  | 0.455 |  | 0.9904 |  | | ocean | 2 | 13090.72 |  | 0.305 |  | 0.9850 |  | | game | 2 | 13110.10 |  | 0.496 |  | 0.9801 |  | | literally | 4 | 13074.36 |  | 0.491 |  | 0.9887 |  | | product | 2 | 13128.04 |  | 0.444 |  | 0.9824 |  | | president | 2 | 13084.02 |  | 0.365 |  | 0.9935 |  | | tells | 3 | 13073.62 |  | 0.626 |  | 0.9908 |  | | happy | 3 | 13061.75 |  | 0.646 |  | 0.9933 |  | | appropriate | 3 | 13072.14 |  | 0.643 |  | 0.9932 |  | | activity | 2 | 13054.35 |  | 0.365 |  | 0.9797 |  | | distance | 2 | 13003.47 |  | 0.348 |  | 0.9786 |  | | cut | 2 | 13029.23 |  | 0.598 |  | 0.9888 |  | | technology | 2 | 13010.08 |  | 0.424 |  | 0.9907 |  | | imply | 3 | 12971.23 |  | 0.654 |  | 0.9962 |  | | moving | 3 | 12974.15 |  | 0.450 |  | 0.9887 |  | | seeing | 3 | 12970.50 |  | 0.706 |  | 0.9945 |  | | established | 3 | 12961.01 |  | 0.577 |  | 0.9891 |  | | board | 2 | 12910.13 |  | 0.348 |  | 0.9768 |  | | newsgroups | 2 | 12917.37 |  | 0.378 |  | 0.9906 |  | | ice | 2 | 12903.61 |  | 0.251 |  | 0.9655 |  | | forget | 3 | 12900.00 |  | 0.717 |  | 0.9960 |  | | contrary | 3 | 12876.91 |  | 0.703 |  | 0.9969 |  | | learned | 3 | 12844.58 |  | 0.611 |  | 0.9874 |  | | answered | 2 | 12820.26 |  | 0.594 |  | 0.9940 |  | | straight | 3 | 12787.50 |  | 0.554 |  | 0.9889 |  | | alive | 3 | 12839.56 |  | 0.447 |  | 0.9846 |  | | week | 2 | 12788.21 |  | 0.517 |  | 0.9835 |  | | pressure | 2 | 12772.60 |  | 0.364 |  | 0.9748 |  | | incorrect | 3 | 12774.02 |  | 0.590 |  | 0.9921 |  | | currently | 3 | 12732.34 |  | 0.662 |  | 0.9957 |  | | darwinism | 2 | 12781.11 |  | 0.315 |  | 0.9905 |  | | trouble | 2 | 12711.95 |  | 0.720 |  | 0.9952 |  | | movement | 2 | 12745.73 |  | 0.444 |  | 0.9909 |  | | bones | 2 | 12681.14 |  | 0.324 |  | 0.9835 |  | | besides | 4 | 12680.44 |  | 0.768 |  | 0.9981 |  | | base | 2 | 12670.67 |  | 0.505 |  | 0.9775 |  | | guys | 2 | 12640.75 |  | 0.546 |  | 0.9906 |  | | extremely | 4 | 12651.87 |  | 0.685 |  | 0.9953 |  | | hasn't | 4 | 12604.76 |  | 0.611 |  | 0.9907 |  | | stone | 2 | 12568.29 |  | 0.313 |  | 0.9749 |  | | surely | 4 | 12575.84 |  | 0.715 |  | 0.9964 |  | | responsible | 3 | 12616.51 |  | 0.524 |  | 0.9877 |  | | catholic | 2 | 12588.91 |  | 0.319 |  | 0.9853 |  | | beings | 2 | 12595.11 |  | 0.484 |  | 0.9916 |  | | amino | 2 | 12515.00 |  | 0.225 |  | 0.9747 |  | | relationship | 2 | 12491.91 |  | 0.503 |  | 0.9823 |  | | approach | 2 | 12468.23 |  | 0.579 |  | 0.9898 |  | | calling | 2 | 12479.72 |  | 0.622 |  | 0.9935 |  | | regarding | 3 | 12501.41 |  | 0.636 |  | 0.9959 |  | | molecules | 2 | 12429.86 |  | 0.309 |  | 0.9885 |  | | race | 2 | 12458.78 |  | 0.359 |  | 0.9904 |  | | kids | 2 | 12391.72 |  | 0.382 |  | 0.9873 |  | | review | 2 | 12414.44 |  | 0.432 |  | 0.9790 |  | | confused | 3 | 12377.06 |  | 0.641 |  | 0.9934 |  | | deep | 3 | 12355.14 |  | 0.554 |  | 0.9913 |  | | community | 2 | 12347.85 |  | 0.508 |  | 0.9904 |  | | ian | 1 | 12328.02 |  | 0.234 |  | 0.9912 |  | | blood | 2 | 12358.46 |  | 0.355 |  | 0.9781 |  | | links | 2 | 12313.51 |  | 0.456 |  | 0.9914 |  | | basically | 4 | 12316.80 |  | 0.722 |  | 0.9970 |  | | towards | 2 | 12310.88 |  | 0.559 |  | 0.9904 |  | | freedom | 2 | 12255.17 |  | 0.319 |  | 0.9860 |  | | attention | 2 | 12248.65 |  | 0.678 |  | 0.9936 |  | | constant | 2 | 12233.68 |  | 0.411 |  | 0.9860 |  | | dog | 2 | 12223.94 |  | 0.375 |  | 0.9847 |  | | middle | 3 | 12223.94 |  | 0.564 |  | 0.9907 |  | | fully | 4 | 12212.91 |  | 0.591 |  | 0.9900 |  | | follows | 2 | 12220.69 |  | 0.590 |  | 0.9963 |  | | late | 3 | 12206.44 |  | 0.633 |  | 0.9930 |  | | capable | 3 | 12207.73 |  | 0.619 |  | 0.9943 |  | | house | 2 | 12231.73 |  | 0.462 |  | 0.9842 |  | | culture | 2 | 12212.91 |  | 0.415 |  | 0.9898 |  | | key | 2 | 12189.63 |  | 0.586 |  | 0.9930 |  | | referring | 3 | 12157.44 |  | 0.666 |  | 0.9945 |  | | speed | 2 | 12130.54 |  | 0.334 |  | 0.9768 |  | | finding | 2 | 12130.54 |  | 0.642 |  | 0.9934 |  | | born | 3 | 12154.24 |  | 0.483 |  | 0.9885 |  | | quoted | 3 | 12126.07 |  | 0.558 |  | 0.9951 |  | | content | 2 | 12080.89 |  | 0.488 |  | 0.9859 |  | | biologists | 2 | 12106.30 |  | 0.472 |  | 0.9955 |  | | hence | 4 | 12041.71 |  | 0.612 |  | 0.9938 |  | | hitler | 1 | 12567.60 |  | 0.212 |  | 0.9348 |  | | errors | 2 | 11990.28 |  | 0.469 |  | 0.9871 |  | | count | 2 | 11996.53 |  | 0.566 |  | 0.9835 |  | | zoe | 1 | 11974.69 |  | 0.186 |  | 0.6677 |  | | dark | 2 | 11980.92 |  | 0.374 |  | 0.9856 |  | | reject | 3 | 11986.53 |  | 0.515 |  | 0.9931 |  | | global | 3 | 11987.16 |  | 0.364 |  | 0.9900 |  | | picture | 2 | 11965.97 |  | 0.527 |  | 0.9853 |  | | religions | 2 | 11965.97 |  | 0.413 |  | 0.9957 |  | | expected | 3 | 11948.58 |  | 0.596 |  | 0.9911 |  | | stuart | 1 | 11916.42 |  | 0.273 |  | 0.9899 |  | | lives | 2 | 11930.63 |  | 0.584 |  | 0.9974 |  | | spam | 2 | 11885.05 |  | 0.395 |  | 0.9985 |  | | missing | 3 | 11902.87 |  | 0.541 |  | 0.9832 |  | | normal | 3 | 11877.08 |  | 0.528 |  | 0.9841 |  | | description | 2 | 11859.94 |  | 0.579 |  | 0.9903 |  | | soon | 3 | 11866.67 |  | 0.707 |  | 0.9969 |  | | moment | 2 | 11836.16 |  | 0.664 |  | 0.9941 |  | | definitions | 2 | 11823.40 |  | 0.447 |  | 0.9955 |  | | studies | 2 | 11803.39 |  | 0.504 |  | 0.9903 |  | | formation | 2 | 11838.60 |  | 0.407 |  | 0.9925 |  | | here's | U | 11791.91 |  | 0.713 |  | 0.9957 |  | | perfectly | 4 | 11789.49 |  | 0.688 |  | 0.9950 |  | | timberwoof | 1 | 11749.18 |  | 0.169 |  | 0.8881 |  | | xp | 1 | 11749.18 |  | 0.255 |  | 0.9838 |  | | believes | 3 | 11772.01 |  | 0.584 |  | 0.9944 |  | | literal | 3 | 11755.18 |  | 0.395 |  | 0.9937 |  | | dishonest | 3 | 11719.27 |  | 0.438 |  | 0.9943 |  | | despite | 2 | 11725.24 |  | 0.731 |  | 0.9971 |  | | action | 2 | 11705.57 |  | 0.543 |  | 0.9894 |  | | blogspot | 2 | 11672.92 |  | 0.351 |  | 0.9861 |  | | stories | 2 | 11675.88 |  | 0.423 |  | 0.9863 |  | | areas | 2 | 11676.47 |  | 0.529 |  | 0.9879 |  | | offer | 2 | 11648.71 |  | 0.575 |  | 0.9908 |  | | judge | 2 | 11704.38 |  | 0.404 |  | 0.9868 |  | | proposed | 3 | 11640.46 |  | 0.567 |  | 0.9945 |  | | relative | 3 | 11653.42 |  | 0.498 |  | 0.9886 |  | | analogy | 2 | 11652.24 |  | 0.427 |  | 0.9880 |  | | thousand | 3 | 11624.59 |  | 0.533 |  | 0.9893 |  | | quotes | 2 | 11647.53 |  | 0.437 |  | 0.9877 |  | | refer | 3 | 11629.29 |  | 0.626 |  | 0.9941 |  | | plenty | 2 | 11598.24 |  | 0.621 |  | 0.9957 |  | | survival | 2 | 11622.83 |  | 0.379 |  | 0.9902 |  | | worse | 3 | 11599.99 |  | 0.657 |  | 0.9944 |  | | magic | 2 | 11557.48 |  | 0.383 |  | 0.9955 |  | | developed | 3 | 11565.02 |  | 0.574 |  | 0.9921 |  | | rational | 3 | 11572.58 |  | 0.475 |  | 0.9899 |  | | mostly | 4 | 11536.63 |  | 0.720 |  | 0.9966 |  | | pay | 2 | 11529.69 |  | 0.491 |  | 0.9878 |  | | observe | 3 | 11521.61 |  | 0.496 |  | 0.9942 |  | | trees | 2 | 11523.34 |  | 0.357 |  | 0.9918 |  | | eventually | 4 | 11511.24 |  | 0.671 |  | 0.9956 |  | | department | 2 | 11488.27 |  | 0.420 |  | 0.9973 |  | | parents | 2 | 11551.10 |  | 0.395 |  | 0.9843 |  | | forces | 2 | 11483.11 |  | 0.437 |  | 0.9889 |  | | giving | 3 | 11435.20 |  | 0.750 |  | 0.9966 |  | | creatures | 2 | 11422.15 |  | 0.442 |  | 0.9906 |  | | articles | 2 | 11405.17 |  | 0.456 |  | 0.9841 |  | | explains | 3 | 11400.09 |  | 0.606 |  | 0.9940 |  | | types | 2 | 11386.56 |  | 0.560 |  | 0.9905 |  | | absolute | 3 | 11383.18 |  | 0.447 |  | 0.9813 |  | | sex | 2 | 11375.86 |  | 0.306 |  | 0.9735 |  | | third | 3 | 11365.19 |  | 0.633 |  | 0.9919 |  | | quantum | 2 | 11347.27 |  | 0.336 |  | 0.9863 |  | | literature | 2 | 11341.12 |  | 0.532 |  | 0.9925 |  | | understood | 3 | 11359.59 |  | 0.664 |  | 0.9944 |  | | et | U | 11322.71 |  | 0.380 |  | 0.9652 |  | | personally | 4 | 11327.73 |  | 0.728 |  | 0.9982 |  | | supported | 2 | 11325.50 |  | 0.604 |  | 0.9947 |  | | edu | U | 11293.27 |  | 0.323 |  | 0.9963 |  | | homo | U | 11304.36 |  | 0.323 |  | 0.9880 |  | | failed | 3 | 11321.60 |  | 0.636 |  | 0.9949 |  | | experiments | 2 | 11276.13 |  | 0.435 |  | 0.9922 |  | | sources | 2 | 11273.36 |  | 0.529 |  | 0.9912 |  | | dick | U | 11297.71 |  | 0.361 |  | 0.9932 |  | | molecular | 2 | 11255.73 |  | 0.380 |  | 0.9915 |  | | lower | 3 | 11254.08 |  | 0.515 |  | 0.9939 |  | | matters | 2 | 11238.69 |  | 0.637 |  | 0.9966 |  | | hey | U | 11321.04 |  | 0.698 |  | 0.9972 |  | | ground | 2 | 11209.69 |  | 0.533 |  | 0.9880 |  | | nearly | 4 | 11193.34 |  | 0.683 |  | 0.9962 |  | | series | 2 | 11242.53 |  | 0.501 |  | 0.9844 |  | | survive | 3 | 11206.96 |  | 0.471 |  | 0.9931 |  | | recall | 3 | 11176.49 |  | 0.664 |  | 0.9952 |  | | genetics | 2 | 11175.41 |  | 0.426 |  | 0.9950 |  | | member | 2 | 11154.29 |  | 0.433 |  | 0.9897 |  | | mammals | 2 | 11127.33 |  | 0.343 |  | 0.9906 |  | | unfortunately | 4 | 11120.88 |  | 0.795 |  | 0.9991 |  | | independent | 3 | 11124.64 |  | 0.499 |  | 0.9878 |  | | situation | 2 | 11121.42 |  | 0.631 |  | 0.9951 |  | | somewhere | 3 | 11098.36 |  | 0.750 |  | 0.9966 |  | | bush | U | 11081.80 |  | 0.270 |  | 0.9762 |  | | predictions | 2 | 11065.29 |  | 0.358 |  | 0.9893 |  | | believed | 3 | 11062.10 |  | 0.588 |  | 0.9950 |  | | joe | 1 | 11011.83 |  | 0.258 |  | 0.9635 |  | | dinosaurs | 2 | 11000.26 |  | 0.297 |  | 0.9805 |  | | geocities | 1 | 10998.68 |  | 0.289 |  | 0.9893 |  | | virus | 2 | 10985.03 |  | 0.248 |  | 0.9853 |  | | red | 2 | 10991.85 |  | 0.435 |  | 0.9787 |  | | discovered | 3 | 10984.51 |  | 0.601 |  | 0.9928 |  | | manner | 2 | 10977.70 |  | 0.673 |  | 0.9968 |  | | looked | 2 | 10970.90 |  | 0.677 |  | 0.9936 |  | | begin | 3 | 10967.24 |  | 0.686 |  | 0.9947 |  | | allowed | 4 | 10976.65 |  | 0.637 |  | 0.9945 |  | | proteins | 2 | 10950.55 |  | 0.263 |  | 0.9866 |  | | becomes | 3 | 10985.56 |  | 0.670 |  | 0.9919 |  | | scott | 1 | 10932.87 |  | 0.298 |  | 0.9811 |  | | funny | 3 | 10933.91 |  | 0.639 |  | 0.9946 |  | | thomas | 1 | 10914.21 |  | 0.444 |  | 0.9967 |  | | south | 2 | 10913.17 |  | 0.426 |  | 0.9958 |  | | larger | 3 | 10871.43 |  | 0.524 |  | 0.9893 |  | | heat | 2 | 10854.51 |  | 0.275 |  | 0.9764 |  | | ancestors | 2 | 10847.35 |  | 0.422 |  | 0.9943 |  | | arguing | 3 | 10845.82 |  | 0.620 |  | 0.9935 |  | | challenge | 2 | 10852.98 |  | 0.451 |  | 0.9844 |  | | blog | 2 | 10811.19 |  | 0.458 |  | 0.9958 |  | | sentence | 2 | 10817.79 |  | 0.544 |  | 0.9851 |  | | air | 2 | 11304.92 |  | 0.427 |  | 0.9716 |  | | low | 3 | 10824.40 |  | 0.541 |  | 0.9927 |  | | occurred | 3 | 10804.59 |  | 0.582 |  | 0.9954 |  | | showing | 2 | 10793.95 |  | 0.680 |  | 0.9967 |  | | ah | U | 10797.49 |  | 0.722 |  | 0.9972 |  | | fundamental | 3 | 10775.26 |  | 0.485 |  | 0.9955 |  | | attack | 3 | 10775.76 |  | 0.485 |  | 0.9919 |  | | tom | 1 | 10769.21 |  | 0.480 |  | 0.9991 |  | | became | 3 | 10777.78 |  | 0.600 |  | 0.9922 |  | | remains | 3 | 10727.56 |  | 0.611 |  | 0.9914 |  | | cheers | U | 10706.60 |  | 0.523 |  | 0.9972 |  | | conclusions | 2 | 10703.62 |  | 0.547 |  | 0.9923 |  | | lord | 1 | 10664.45 |  | 0.346 |  | 0.9813 |  | | objective | 2 | 10649.15 |  | 0.406 |  | 0.9894 |  | | equal | 3 | 10641.77 |  | 0.519 |  | 0.9876 |  | | fun | 2 | 10611.36 |  | 0.589 |  | 0.9948 |  | | hardly | 4 | 10625.07 |  | 0.722 |  | 0.9981 |  | | america | 1 | 10629.48 |  | 0.439 |  | 0.9953 |  | | uses | 2 | 10585.98 |  | 0.654 |  | 0.9957 |  | | project | 2 | 10548.63 |  | 0.419 |  | 0.9973 |  | | perfect | 3 | 10567.52 |  | 0.506 |  | 0.9818 |  | | proven | 3 | 10528.37 |  | 0.521 |  | 0.9951 |  | | direction | 2 | 10524.04 |  | 0.443 |  | 0.9846 |  | | authority | 2 | 10523.56 |  | 0.446 |  | 0.9887 |  | | child | 2 | 10528.85 |  | 0.394 |  | 0.9829 |  | | main | 3 | 10508.19 |  | 0.657 |  | 0.9937 |  | | writing | 3 | 10489.04 |  | 0.569 |  | 0.9910 |  | | court | 2 | 10504.83 |  | 0.319 |  | 0.9877 |  | | asp | U | 10462.34 |  | 0.246 |  | 0.9495 |  | | reasoning | 2 | 10450.47 |  | 0.537 |  | 0.9918 |  | | telling | 3 | 10450.47 |  | 0.690 |  | 0.9953 |  | | texas | 1 | 10415.48 |  | 0.394 |  | 0.9975 |  | | btw | 4 | 10407.48 |  | 0.773 |  | 0.9998 |  | | zero | 3 | 10393.38 |  | 0.376 |  | 0.9800 |  | | genome | 2 | 10352.25 |  | 0.283 |  | 0.9872 |  | | recently | 3 | 10360.64 |  | 0.753 |  | 0.9985 |  | | finally | 4 | 10366.70 |  | 0.725 |  | 0.9979 |  | | faq | 2 | 10343.41 |  | 0.341 |  | 0.9958 |  | | bryant | 1 | 10347.60 |  | 0.361 |  | 0.9947 |  | | places | 2 | 10349.46 |  | 0.614 |  | 0.9948 |  | | interest | 2 | 10348.53 |  | 0.658 |  | 0.9944 |  | | vs | 4 | 10345.27 |  | 0.398 |  | 0.9735 |  | | object | 2 | 10325.32 |  | 0.461 |  | 0.9822 |  | | respond | 3 | 10335.06 |  | 0.579 |  | 0.9930 |  | | across | 2 | 10304.06 |  | 0.675 |  | 0.9946 |  | | chemical | 2 | 10303.14 |  | 0.401 |  | 0.9925 |  | | sciences | 2 | 10295.77 |  | 0.519 |  | 0.9970 |  | | folks | 2 | 10284.73 |  | 0.566 |  | 0.9941 |  | | depends | 3 | 10278.30 |  | 0.713 |  | 0.9972 |  | | explanations | 2 | 10264.09 |  | 0.492 |  | 0.9908 |  | | methods | 2 | 10250.84 |  | 0.465 |  | 0.9863 |  | | discovery | 2 | 10225.34 |  | 0.418 |  | 0.9817 |  | | isaak | 1 | 10176.53 |  | 0.405 |  | 0.9976 |  | | darwin's | U | 10231.25 |  | 0.391 |  | 0.9933 |  | | ark | 1 | 10162.60 |  | 0.224 |  | 0.9405 |  | | moon | 2 | 10163.05 |  | 0.324 |  | 0.9837 |  | | usenet | 2 | 10173.83 |  | 0.443 |  | 0.9920 |  | | brought | 2 | 10172.93 |  | 0.668 |  | 0.9978 |  | | structures | 2 | 10185.98 |  | 0.362 |  | 0.9881 |  | | speaking | 3 | 10163.05 |  | 0.719 |  | 0.9972 |  | | fairly | 4 | 10141.56 |  | 0.685 |  | 0.9959 |  | | effects | 2 | 10152.29 |  | 0.494 |  | 0.9914 |  | | ted | 1 | 10121.05 |  | 0.205 |  | 0.8956 |  | | thank | 2 | 10124.16 |  | 0.654 |  | 0.9936 |  | | rule | 2 | 10115.27 |  | 0.519 |  | 0.9854 |  | | describe | 3 | 10117.05 |  | 0.626 |  | 0.9950 |  | | specifically | 4 | 10116.16 |  | 0.701 |  | 0.9974 |  | | chemistry | 2 | 10085.14 |  | 0.420 |  | 0.9920 |  | | stand | 2 | 10107.72 |  | 0.662 |  | 0.9929 |  | | notice | 2 | 10083.37 |  | 0.706 |  | 0.9945 |  | | cox | 1 | 10050.79 |  | 0.317 |  | 0.9837 |  | | wikipedia | 1 | 10033.27 |  | 0.290 |  | 0.9868 |  | | determine | 3 | 10043.78 |  | 0.600 |  | 0.9958 |  | | evolutionist | 2 | 10045.97 |  | 0.448 |  | 0.9987 |  | | provided | 3 | 10021.91 |  | 0.632 |  | 0.9945 |  | | women | 2 | 10034.58 |  | 0.328 |  | 0.9815 |  | | honest | 3 | 10018.42 |  | 0.577 |  | 0.9959 |  | | assumptions | 2 | 9998.41 |  | 0.427 |  | 0.9871 |  | | features | 2 | 9998.84 |  | 0.440 |  | 0.9914 |  | | willing | 4 | 9993.64 |  | 0.656 |  | 0.9954 |  | | bring | 2 | 9993.64 |  | 0.691 |  | 0.9954 |  | | separate | 3 | 9990.60 |  | 0.586 |  | 0.9922 |  | | white | 2 | 9991.47 |  | 0.400 |  | 0.9807 |  | | kinds | 2 | 9971.13 |  | 0.533 |  | 0.9860 |  | | send | 3 | 9972.85 |  | 0.516 |  | 0.9953 |  | | previous | 3 | 9951.30 |  | 0.699 |  | 0.9965 |  | | bobby | 1 | 9946.14 |  | 0.353 |  | 0.9942 |  | | wants | 4 | 9940.99 |  | 0.644 |  | 0.9953 |  | | code | 2 | 9920.85 |  | 0.335 |  | 0.9837 |  | | professor | 2 | 9918.29 |  | 0.441 |  | 0.9978 |  | | top | 2 | 9906.34 |  | 0.563 |  | 0.9885 |  | | instance | 2 | 9904.63 |  | 0.702 |  | 0.9971 |  | | writes | 2 | 9867.27 |  | 0.464 |  | 0.9959 |  | | you'd | U | 9873.62 |  | 0.683 |  | 0.9934 |  | | yours | U | 9867.27 |  | 0.705 |  | 0.9980 |  | | institute | 2 | 9853.34 |  | 0.473 |  | 0.9963 |  | | accurate | 3 | 9841.96 |  | 0.595 |  | 0.9934 |  | | demonstrate | 3 | 9839.44 |  | 0.606 |  | 0.9962 |  | | limited | 3 | 9833.98 |  | 0.648 |  | 0.9948 |  | | details | 2 | 9831.04 |  | 0.622 |  | 0.9931 |  | | de | U | 11811.87 |  | 0.301 |  | 0.9412 |  | | program | 2 | 9803.41 |  | 0.378 |  | 0.9776 |  | | share | 2 | 9808.00 |  | 0.548 |  | 0.9939 |  | | described | 3 | 9792.56 |  | 0.657 |  | 0.9945 |  | | entropy | 2 | 9742.83 |  | 0.155 |  | 0.8327 |  | | food | 2 | 9755.22 |  | 0.416 |  | 0.9880 |  | | dawkins | 1 | 9804.24 |  | 0.280 |  | 0.9782 |  | | jim | 1 | 9726.78 |  | 0.343 |  | 0.9950 |  | | silly | 3 | 9728.42 |  | 0.656 |  | 0.9938 |  | | sci | U | 9714.05 |  | 0.240 |  | 0.9510 |  | | protein | 2 | 9712.82 |  | 0.264 |  | 0.9841 |  | | members | 2 | 9715.69 |  | 0.516 |  | 0.9914 |  | | caused | 4 | 9728.01 |  | 0.576 |  | 0.9929 |  | | realize | 3 | 9700.96 |  | 0.740 |  | 0.9969 |  | | defined | 3 | 9665.93 |  | 0.540 |  | 0.9969 |  | | lines | 2 | 9644.06 |  | 0.581 |  | 0.9890 |  | | notion | 2 | 9646.08 |  | 0.589 |  | 0.9959 |  | | truly | 4 | 9623.90 |  | 0.531 |  | 0.9871 |  | | choose | 3 | 9608.64 |  | 0.563 |  | 0.9914 |  | | eye | 2 | 9592.22 |  | 0.353 |  | 0.9609 |  | | transitional | 3 | 9598.22 |  | 0.265 |  | 0.9798 |  | | experiment | 2 | 9574.27 |  | 0.405 |  | 0.9883 |  | | sequences | 3 | 9562.74 |  | 0.271 |  | 0.9866 |  | | avoid | 3 | 9532.25 |  | 0.683 |  | 0.9963 |  | | near | 2 | 9499.99 |  | 0.635 |  | 0.9954 |  | | worth | 3 | 9502.74 |  | 0.672 |  | 0.9941 |  | | analysis | 2 | 9500.78 |  | 0.512 |  | 0.9902 |  | | thinks | 3 | 9508.63 |  | 0.666 |  | 0.9946 |  | | link | 2 | 9487.47 |  | 0.521 |  | 0.9917 |  | | thousands | 3 | 9461.34 |  | 0.582 |  | 0.9924 |  | | boikat | 1 | 9484.73 |  | 0.364 |  | 0.9952 |  | | fair | 3 | 9453.18 |  | 0.596 |  | 0.9925 |  | | james | 1 | 9445.81 |  | 0.434 |  | 0.9959 |  | | generation | 2 | 9469.90 |  | 0.406 |  | 0.9824 |  | | karl | 1 | 9421.46 |  | 0.212 |  | 0.9267 |  | | mechanisms | 2 | 9423.39 |  | 0.439 |  | 0.9955 |  | | geology | 2 | 9390.33 |  | 0.401 |  | 0.9971 |  | | ad | 2 | 9404.53 |  | 0.447 |  | 0.9852 |  | | populations | 2 | 9398.38 |  | 0.377 |  | 0.9949 |  | | existed | 2 | 9389.57 |  | 0.575 |  | 0.9947 |  | | sound | 2 | 9364.36 |  | 0.641 |  | 0.9906 |  | | play | 2 | 9351.05 |  | 0.648 |  | 0.9940 |  | | step | 2 | 9331.34 |  | 0.485 |  | 0.9777 |  | | behe | 1 | 9329.44 |  | 0.248 |  | 0.9548 |  | | assuming | 3 | 9333.23 |  | 0.689 |  | 0.9968 |  | | totally | 4 | 9323.40 |  | 0.679 |  | 0.9966 |  | | rocks | 2 | 9296.29 |  | 0.328 |  | 0.9866 |  | | bacteria | 2 | 9298.54 |  | 0.315 |  | 0.9874 |  | | sea | 2 | 9316.98 |  | 0.362 |  | 0.9905 |  | | dejanews | 1 | 9213.69 |  | 0.316 |  | 0.9423 |  | | plants | 2 | 9222.92 |  | 0.355 |  | 0.9806 |  | | nyikos | 1 | 9189.42 |  | 0.231 |  | 0.7884 |  | | wanted | 4 | 9227.73 |  | 0.679 |  | 0.9959 |  | | total | 2 | 9170.39 |  | 0.512 |  | 0.9841 |  | | apes | 2 | 9163.09 |  | 0.285 |  | 0.9854 |  | | principle | 2 | 9387.65 |  | 0.488 |  | 0.9895 |  | | tend | 4 | 9174.77 |  | 0.662 |  | 0.9944 |  | | couple | 2 | 9166.73 |  | 0.748 |  | 0.9978 |  | | gould | 1 | 9181.72 |  | 0.271 |  | 0.9755 |  | | gans | 1 | 9147.07 |  | 0.248 |  | 0.9434 |  | | dating | 3 | 9133.28 |  | 0.267 |  | 0.9767 |  | | consistent | 3 | 9135.81 |  | 0.537 |  | 0.9914 |  | | nobody | 3 | 9125.67 |  | 0.654 |  | 0.9947 |  | | control | 3 | 9107.62 |  | 0.486 |  | 0.9913 |  | | al | U | 9245.89 |  | 0.381 |  | 0.9749 |  | | significant | 3 | 9091.44 |  | 0.609 |  | 0.9958 |  | | choice | 2 | 9060.31 |  | 0.485 |  | 0.9861 |  | | bang | 2 | 9039.67 |  | 0.299 |  | 0.9827 |  | | measure | 2 | 9020.19 |  | 0.454 |  | 0.9939 |  | | whose | U | 9027.62 |  | 0.717 |  | 0.9967 |  | | formed | 3 | 9008.90 |  | 0.474 |  | 0.9939 |  | | edconrad | 1 | 8994.47 |  | 0.099 |  | 0.5812 |  | | greater | 3 | 9037.19 |  | 0.591 |  | 0.9928 |  | | local | 3 | 8999.04 |  | 0.528 |  | 0.9868 |  | | historical | 3 | 8986.39 |  | 0.459 |  | 0.9915 |  | | apply | 3 | 8965.04 |  | 0.635 |  | 0.9948 |  | | individuals | 2 | 8989.20 |  | 0.461 |  | 0.9883 |  | | serious | 3 | 8933.04 |  | 0.663 |  | 0.9934 |  | | alternative | 3 | 8916.09 |  | 0.522 |  | 0.9902 |  | | variation | 2 | 8913.67 |  | 0.382 |  | 0.9906 |  | | eyes | 2 | 8924.73 |  | 0.470 |  | 0.9826 |  | | somehow | 3 | 8891.98 |  | 0.740 |  | 0.9963 |  | | development | 2 | 8892.67 |  | 0.496 |  | 0.9917 |  | | steve | 1 | 8851.64 |  | 0.374 |  | 0.9954 |  | | liar | 2 | 8871.76 |  | 0.402 |  | 0.9954 |  | | yeah | U | 8854.70 |  | 0.697 |  | 0.9979 |  | | fellow | 2 | 8856.75 |  | 0.468 |  | 0.9984 |  | | poor | 2 | 8844.50 |  | 0.628 |  | 0.9934 |  | | students | 2 | 8831.94 |  | 0.348 |  | 0.9853 |  | | majority | 2 | 8801.89 |  | 0.543 |  | 0.9917 |  | | highly | 4 | 8785.43 |  | 0.633 |  | 0.9959 |  | | kill | 2 | 8784.42 |  | 0.405 |  | 0.9906 |  | | pattern | 2 | 8765.36 |  | 0.370 |  | 0.9872 |  | | move | 2 | 8765.02 |  | 0.576 |  | 0.9944 |  | | seriously | 4 | 8748.70 |  | 0.713 |  | 0.9963 |  | | george | 1 | 8746.71 |  | 0.356 |  | 0.9910 |  | | comments | 2 | 8744.72 |  | 0.612 |  | 0.9967 |  | | gods | 2 | 8728.14 |  | 0.379 |  | 0.9906 |  | | increase | 3 | 8715.91 |  | 0.423 |  | 0.9934 |  | | e-mail | 2 | 8711.29 |  | 0.452 |  | 0.9992 |  | | accepted | 3 | 8717.56 |  | 0.619 |  | 0.9944 |  | | math | 2 | 8664.74 |  | 0.421 |  | 0.9949 |  | | direct | 3 | 8648.14 |  | 0.606 |  | 0.9919 |  | | stars | 2 | 8621.26 |  | 0.338 |  | 0.9900 |  | | particularly | 4 | 8619.96 |  | 0.730 |  | 0.9973 |  | | continue | 3 | 8609.00 |  | 0.710 |  | 0.9960 |  | | causes | 2 | 8606.75 |  | 0.482 |  | 0.9872 |  | | deny | 3 | 8609.33 |  | 0.573 |  | 0.9946 |  | | die | 2 | 8596.47 |  | 0.486 |  | 0.9937 |  | | mine | U | 8567.99 |  | 0.635 |  | 0.9952 |  | | sounds | 2 | 8570.86 |  | 0.751 |  | 0.9962 |  | | guy | 2 | 8546.68 |  | 0.579 |  | 0.9950 |  | | coming | 2 | 8538.12 |  | 0.699 |  | 0.9960 |  | | beginning | 2 | 8536.54 |  | 0.582 |  | 0.9903 |  | | lead | 2 | 8530.53 |  | 0.669 |  | 0.9950 |  | | ancient | 3 | 8506.89 |  | 0.486 |  | 0.9931 |  | | tried | 3 | 8514.75 |  | 0.724 |  | 0.9959 |  | | christ | 1 | 8507.52 |  | 0.353 |  | 0.9947 |  | | stated | 2 | 8507.20 |  | 0.691 |  | 0.9972 |  | | adam | 1 | 8471.20 |  | 0.348 |  | 0.9940 |  | | jews | 2 | 8583.65 |  | 0.272 |  | 0.9816 |  | | earlier | 3 | 8462.79 |  | 0.700 |  | 0.9967 |  | | issues | 2 | 8473.70 |  | 0.600 |  | 0.9944 |  | | face | 2 | 8480.25 |  | 0.649 |  | 0.9933 |  | | absolutely | 4 | 8457.20 |  | 0.722 |  | 0.9972 |  | | aware | 3 | 8475.57 |  | 0.682 |  | 0.9939 |  | | gives | 2 | 8464.04 |  | 0.711 |  | 0.9958 |  | | family | 2 | 8451.92 |  | 0.464 |  | 0.9895 |  | | write | 2 | 8430.87 |  | 0.648 |  | 0.9931 |  | | lying | 3 | 8432.42 |  | 0.491 |  | 0.9948 |  | | wish | 2 | 8432.11 |  | 0.721 |  | 0.9965 |  | | author | 2 | 8425.32 |  | 0.499 |  | 0.9930 |  | | possibility | 2 | 8397.04 |  | 0.624 |  | 0.9956 |  | | teaching | 2 | 8379.01 |  | 0.392 |  | 0.9938 |  | | shall | 4 | 8401.03 |  | 0.384 |  | 0.9653 |  | | lots | 3 | 8376.27 |  | 0.636 |  | 0.9917 |  | | respect | 2 | 8376.88 |  | 0.581 |  | 0.9913 |  | | behind | 2 | 8373.53 |  | 0.676 |  | 0.9959 |  | | ancestor | 2 | 8370.18 |  | 0.341 |  | 0.9927 |  | | rules | 2 | 8358.33 |  | 0.465 |  | 0.9841 |  | | millions | 3 | 8357.42 |  | 0.540 |  | 0.9948 |  | | per | 2 | 8367.14 |  | 0.459 |  | 0.9798 |  | | four | 3 | 8332.01 |  | 0.523 |  | 0.9856 |  | | differences | 2 | 8322.67 |  | 0.438 |  | 0.9858 |  | | nonsense | 2 | 8326.58 |  | 0.627 |  | 0.9948 |  | | logical | 3 | 8313.35 |  | 0.533 |  | 0.9941 |  | | published | 2 | 8272.13 |  | 0.520 |  | 0.9929 |  | | saw | 3 | 8262.33 |  | 0.646 |  | 0.9937 |  | | fall | 2 | 8246.06 |  | 0.598 |  | 0.9930 |  | | organism | 2 | 8260.26 |  | 0.400 |  | 0.9974 |  | | produced | 3 | 8229.25 |  | 0.540 |  | 0.9936 |  | | job | U | 8228.08 |  | 0.588 |  | 0.9901 |  | | strong | 3 | 8223.67 |  | 0.642 |  | 0.9945 |  | | irrelevant | 3 | 8208.71 |  | 0.617 |  | 0.9962 |  | | taking | 3 | 8215.45 |  | 0.770 |  | 0.9979 |  | | surface | 2 | 8183.91 |  | 0.350 |  | 0.9915 |  | | alt | U | 8197.02 |  | 0.346 |  | 0.9932 |  | | allow | 4 | 8192.65 |  | 0.720 |  | 0.9963 |  | | require | 3 | 8182.46 |  | 0.657 |  | 0.9960 |  | | leave | 2 | 8188.86 |  | 0.726 |  | 0.9959 |  | | define | 3 | 8187.69 |  | 0.545 |  | 0.9957 |  | | pointed | 2 | 8179.55 |  | 0.730 |  | 0.9986 |  | | entirely | 4 | 8174.03 |  | 0.740 |  | 0.9972 |  | | imagine | 3 | 8155.50 |  | 0.675 |  | 0.9929 |  | | topic | 2 | 8146.84 |  | 0.609 |  | 0.9939 |  | | fish | 2 | 8156.36 |  | 0.318 |  | 0.9728 |  | | add | 2 | 8135.04 |  | 0.698 |  | 0.9942 |  | | difficult | 3 | 8136.76 |  | 0.725 |  | 0.9971 |  | | average | 3 | 8105.53 |  | 0.410 |  | 0.9823 |  | | land | 2 | 8098.98 |  | 0.409 |  | 0.9865 |  | | half | 3 | 8096.13 |  | 0.562 |  | 0.9859 |  | | mike | 1 | 8087.31 |  | 0.333 |  | 0.9873 |  | | speak | 2 | 8090.72 |  | 0.656 |  | 0.9955 |  | | special | 3 | 8072.28 |  | 0.581 |  | 0.9884 |  | | asking | 3 | 8051.39 |  | 0.641 |  | 0.9940 |  | | rich | 2 | 8045.76 |  | 0.311 |  | 0.9824 |  | | changed | 2 | 8034.25 |  | 0.621 |  | 0.9934 |  | | biblical | 2 | 8023.89 |  | 0.471 |  | 0.9987 |  | | creator | 1 | 8022.77 |  | 0.383 |  | 0.9935 |  | | andrew | 1 | 8013.00 |  | 0.345 |  | 0.9929 |  | | alone | 3 | 8032.85 |  | 0.711 |  | 0.9965 |  | | hear | 3 | 8014.11 |  | 0.669 |  | 0.9947 |  | | figure | 2 | 8001.58 |  | 0.635 |  | 0.9927 |  | | include | 3 | 8002.13 |  | 0.704 |  | 0.9965 |  | | relevant | 3 | 7996.30 |  | 0.638 |  | 0.9951 |  | | evolve | 3 | 7962.83 |  | 0.454 |  | 0.9972 |  | | directly | 3 | 7939.76 |  | 0.659 |  | 0.9946 |  | | mentioned | 3 | 7926.64 |  | 0.729 |  | 0.9974 |  | | generally | 4 | 7944.14 |  | 0.717 |  | 0.9973 |  | | billion | 3 | 7890.23 |  | 0.405 |  | 0.9842 |  | | working | 3 | 7898.08 |  | 0.687 |  | 0.9950 |  | | needs | 2 | 7884.83 |  | 0.708 |  | 0.9962 |  | | comment | 2 | 7881.32 |  | 0.658 |  | 0.9957 |  | | period | 2 | 7874.04 |  | 0.575 |  | 0.9928 |  | | act | 2 | 7883.21 |  | 0.577 |  | 0.9923 |  | | couldn't | 4 | 7875.39 |  | 0.729 |  | 0.9973 |  | | degree | 2 | 7861.14 |  | 0.551 |  | 0.9906 |  | | assumption | 2 | 7843.99 |  | 0.500 |  | 0.9915 |  | | class | 2 | 7846.40 |  | 0.449 |  | 0.9845 |  | | assertion | 2 | 7847.20 |  | 0.527 |  | 0.9933 |  | | lies | 2 | 7839.19 |  | 0.532 |  | 0.9922 |  | | probability | 2 | 7816.30 |  | 0.284 |  | 0.9785 |  | | future | 2 | 7818.15 |  | 0.567 |  | 0.9895 |  | | political | 2 | 7827.99 |  | 0.445 |  | 0.9985 |  | | tree | 2 | 7826.66 |  | 0.360 |  | 0.9806 |  | | possibly | 4 | 7789.32 |  | 0.782 |  | 0.9983 |  | | cells | 2 | 7774.59 |  | 0.305 |  | 0.9839 |  | | lost | 3 | 7785.63 |  | 0.633 |  | 0.9940 |  | | ignore | 3 | 7779.84 |  | 0.663 |  | 0.9976 |  | | easily | 4 | 7769.08 |  | 0.758 |  | 0.9986 |  | | needed | 3 | 7776.43 |  | 0.652 |  | 0.9945 |  | | atheists | 2 | 7779.32 |  | 0.343 |  | 0.9933 |  | | references | 2 | 7755.21 |  | 0.548 |  | 0.9961 |  | | ignorant | 3 | 7766.20 |  | 0.588 |  | 0.9958 |  | | gave | 2 | 7762.27 |  | 0.714 |  | 0.9964 |  | | newsgroup | 2 | 7739.31 |  | 0.469 |  | 0.9943 |  | | ray | 1 | 7736.96 |  | 0.170 |  | 0.8087 |  | | computer | 2 | 7734.88 |  | 0.450 |  | 0.9880 |  | | speciation | 2 | 7710.02 |  | 0.269 |  | 0.9857 |  | | abiogenesis | 2 | 7699.44 |  | 0.273 |  | 0.9792 |  | | bill | 1 | 7688.90 |  | 0.386 |  | 0.9924 |  | | knew | 3 | 7701.76 |  | 0.663 |  | 0.9949 |  | | takes | 2 | 7681.46 |  | 0.733 |  | 0.9968 |  | | robert | 1 | 7683.51 |  | 0.454 |  | 0.9958 |  | | michael | 2 | 7805.43 |  | 0.451 |  | 0.9974 |  | | disagree | 3 | 7677.36 |  | 0.662 |  | 0.9980 |  | | social | 3 | 7674.80 |  | 0.395 |  | 0.9933 |  | | schools | 2 | 7632.56 |  | 0.349 |  | 0.9899 |  | | century | 2 | 7636.61 |  | 0.528 |  | 0.9939 |  | | god's | U | 7650.57 |  | 0.448 |  | 0.9985 |  | | claiming | 3 | 7620.44 |  | 0.691 |  | 0.9981 |  | | valid | 3 | 7592.54 |  | 0.584 |  | 0.9932 |  | | discuss | 3 | 7589.29 |  | 0.493 |  | 0.9934 |  | | answers | 2 | 7563.60 |  | 0.557 |  | 0.9916 |  | | necessarily | 4 | 7535.13 |  | 0.697 |  | 0.9992 |  | | you'll | U | 7531.68 |  | 0.616 |  | 0.9930 |  | | country | 2 | 7528.48 |  | 0.482 |  | 0.9993 |  | | teach | 3 | 7496.85 |  | 0.396 |  | 0.9904 |  | | english | 2 | 7508.84 |  | 0.448 |  | 0.9860 |  | | hell | U | 7541.06 |  | 0.572 |  | 0.9932 |  | | observations | 2 | 7458.96 |  | 0.436 |  | 0.9940 |  | | behavior | 2 | 7423.36 |  | 0.446 |  | 0.9866 |  | | complete | 3 | 7423.60 |  | 0.694 |  | 0.9947 |  | | black | 2 | 7425.04 |  | 0.420 |  | 0.9791 |  | | conditions | 2 | 7403.55 |  | 0.482 |  | 0.9902 |  | | moral | 2 | 7417.86 |  | 0.356 |  | 0.9903 |  | | views | 2 | 7386.21 |  | 0.545 |  | 0.9961 |  | | area | 2 | 7377.22 |  | 0.551 |  | 0.9911 |  | | statements | 2 | 7352.48 |  | 0.575 |  | 0.9928 |  | | check | 2 | 7338.41 |  | 0.624 |  | 0.9938 |  | | turn | 2 | 7347.08 |  | 0.729 |  | 0.9954 |  | | error | 2 | 7333.97 |  | 0.511 |  | 0.9870 |  | | suspect | 2 | 7329.07 |  | 0.728 |  | 0.9973 |  | | we're | U | 7301.41 |  | 0.658 |  | 0.9938 |  | | animal | 2 | 7276.25 |  | 0.458 |  | 0.9944 |  | | required | 3 | 7260.42 |  | 0.636 |  | 0.9971 |  | | hold | 2 | 7249.67 |  | 0.682 |  | 0.9949 |  | | higher | 3 | 7257.22 |  | 0.549 |  | 0.9921 |  | | explained | 3 | 7242.83 |  | 0.633 |  | 0.9974 |  | | fine | 3 | 7244.20 |  | 0.684 |  | 0.9960 |  | | run | 2 | 7188.55 |  | 0.641 |  | 0.9939 |  | | useful | 3 | 7182.27 |  | 0.576 |  | 0.9941 |  | | presented | 3 | 7166.84 |  | 0.625 |  | 0.9975 |  | | gravity | 2 | 7158.38 |  | 0.346 |  | 0.9906 |  | | cell | 2 | 7141.06 |  | 0.331 |  | 0.9890 |  | | dead | 2 | 7135.97 |  | 0.552 |  | 0.9902 |  | | looks | 2 | 7126.47 |  | 0.700 |  | 0.9962 |  | | planet | 2 | 7103.38 |  | 0.470 |  | 0.9934 |  | | education | 2 | 7057.21 |  | 0.444 |  | 0.9909 |  | | appears | 4 | 7062.84 |  | 0.736 |  | 0.9966 |  | | requires | 3 | 7041.67 |  | 0.635 |  | 0.9976 |  | | money | 2 | 7029.85 |  | 0.433 |  | 0.9872 |  | | occur | 3 | 7017.84 |  | 0.561 |  | 0.9977 |  | | cases | 2 | 7005.88 |  | 0.598 |  | 0.9938 |  | | atheism | 2 | 7053.97 |  | 0.344 |  | 0.9944 |  | | value | 2 | 7007.16 |  | 0.505 |  | 0.9851 |  | | went | U | 6989.72 |  | 0.686 |  | 0.9954 |  | | society | 2 | 6994.60 |  | 0.451 |  | 0.9947 |  | | claimed | 3 | 6977.22 |  | 0.636 |  | 0.9972 |  | | gets | 2 | 6971.73 |  | 0.733 |  | 0.9963 |  | | follow | 2 | 6974.68 |  | 0.669 |  | 0.9943 |  | | recent | 3 | 6967.08 |  | 0.657 |  | 0.9978 |  | | rock | 2 | 6952.15 |  | 0.332 |  | 0.9833 |  | | birds | 2 | 6935.81 |  | 0.303 |  | 0.9825 |  | | below | 2 | 6942.08 |  | 0.620 |  | 0.9969 |  | | admit | 3 | 6895.30 |  | 0.685 |  | 0.9962 |  | | mr | U | 6894.06 |  | 0.389 |  | 0.9805 |  | | supposed | 3 | 6858.12 |  | 0.707 |  | 0.9959 |  | | ability | 2 | 6854.04 |  | 0.595 |  | 0.9924 |  | | started | 2 | 6848.53 |  | 0.699 |  | 0.9952 |  | | open | 2 | 6824.57 |  | 0.613 |  | 0.9928 |  | | descent | 2 | 6802.79 |  | 0.350 |  | 0.9924 |  | | government | 2 | 6790.35 |  | 0.350 |  | 0.9940 |  | | jpg | U | 6750.73 |  | 0.142 |  | 0.9150 |  | | opinions | 2 | 6745.98 |  | 0.473 |  | 0.9981 |  | | search | 2 | 6732.76 |  | 0.455 |  | 0.9930 |  | | text | 2 | 6708.83 |  | 0.501 |  | 0.9897 |  | | nice | 3 | 6706.68 |  | 0.736 |  | 0.9962 |  | | impossible | 3 | 6695.75 |  | 0.622 |  | 0.9963 |  | | longer | 3 | 6695.36 |  | 0.704 |  | 0.9952 |  | | christianity | 2 | 6678.47 |  | 0.391 |  | 0.9972 |  | | dave | 1 | 6653.57 |  | 0.317 |  | 0.9870 |  | | structure | 2 | 6649.34 |  | 0.450 |  | 0.9895 |  | | complexity | 2 | 6640.52 |  | 0.312 |  | 0.9896 |  | | mention | 2 | 6628.66 |  | 0.722 |  | 0.9962 |  | | date | 2 | 6610.96 |  | 0.502 |  | 0.9923 |  | | buy | 2 | 6606.97 |  | 0.388 |  | 0.9789 |  | | pagano | 1 | 6558.04 |  | 0.159 |  | 0.6916 |  | | purpose | 2 | 6566.46 |  | 0.493 |  | 0.9857 |  | | available | 3 | 6558.97 |  | 0.652 |  | 0.9957 |  | | interested | 3 | 6564.96 |  | 0.692 |  | 0.9959 |  | | version | 2 | 6536.62 |  | 0.572 |  | 0.9941 |  | | attempt | 3 | 6507.06 |  | 0.729 |  | 0.9975 |  | | evil | 2 | 6517.01 |  | 0.376 |  | 0.9889 |  | | supernatural | 3 | 6493.11 |  | 0.318 |  | 0.9884 |  | | posting | 2 | 6498.43 |  | 0.564 |  | 0.9960 |  | | dot | 2 | 6486.53 |  | 0.285 |  | 0.9977 |  | | taught | 3 | 6468.30 |  | 0.417 |  | 0.9931 |  | | experience | 2 | 6466.30 |  | 0.508 |  | 0.9898 |  | | philosophy | 2 | 6468.48 |  | 0.446 |  | 0.9969 |  | | outside | 2 | 6446.75 |  | 0.602 |  | 0.9949 |  | | young | 3 | 6437.91 |  | 0.507 |  | 0.9966 |  | | amount | 2 | 6431.08 |  | 0.598 |  | 0.9938 |  | | reasonable | 3 | 6421.39 |  | 0.654 |  | 0.9961 |  | | meant | 3 | 6403.35 |  | 0.692 |  | 0.9960 |  | | type | 2 | 6379.22 |  | 0.609 |  | 0.9913 |  | | size | 2 | 6364.76 |  | 0.416 |  | 0.9899 |  | | involved | 3 | 6377.28 |  | 0.704 |  | 0.9973 |  | | wonder | 3 | 6362.83 |  | 0.742 |  | 0.9975 |  | | ed | 1 | 6361.07 |  | 0.241 |  | 0.9435 |  | | took | 2 | 6357.03 |  | 0.723 |  | 0.9977 |  | | basis | 2 | 6349.84 |  | 0.678 |  | 0.9967 |  | | myself | U | 6348.61 |  | 0.707 |  | 0.9989 |  | | individual | 2 | 6349.14 |  | 0.550 |  | 0.9911 |  | | happens | 3 | 6343.54 |  | 0.709 |  | 0.9975 |  | | numbers | 2 | 6340.39 |  | 0.463 |  | 0.9879 |  | | ways | 2 | 6333.77 |  | 0.725 |  | 0.9968 |  | | home | 2 | 6341.44 |  | 0.544 |  | 0.9960 |  | | full | 3 | 6321.76 |  | 0.701 |  | 0.9957 |  | | sometimes | 3 | 6303.93 |  | 0.674 |  | 0.9915 |  | | suppose | 3 | 6294.63 |  | 0.723 |  | 0.9952 |  | | short | 3 | 6283.63 |  | 0.740 |  | 0.9980 |  | | entire | 3 | 6282.60 |  | 0.724 |  | 0.9968 |  | | force | 2 | 6365.64 |  | 0.484 |  | 0.9861 |  | | create | 3 | 6272.33 |  | 0.541 |  | 0.9944 |  | | knows | 3 | 6267.89 |  | 0.655 |  | 0.9913 |  | | head | 2 | 6264.65 |  | 0.645 |  | 0.9953 |  | | basic | 3 | 6253.25 |  | 0.634 |  | 0.9941 |  | | usually | 4 | 6237.15 |  | 0.733 |  | 0.9973 |  | | close | 2 | 6232.93 |  | 0.716 |  | 0.9961 |  | | remove | 2 | 6220.47 |  | 0.403 |  | 0.9856 |  | | event | 2 | 6222.99 |  | 0.487 |  | 0.9889 |  | | argue | 3 | 6221.65 |  | 0.670 |  | 0.9969 |  | | scientist | 2 | 6165.82 |  | 0.532 |  | 0.9969 |  | | paper | 2 | 6169.96 |  | 0.445 |  | 0.9874 |  | | lie | 2 | 6113.92 |  | 0.503 |  | 0.9944 |  | | what's | U | 6103.06 |  | 0.715 |  | 0.9971 |  | | otherwise | 4 | 6091.91 |  | 0.812 |  | 0.9993 |  | | interpretation | 2 | 6084.99 |  | 0.499 |  | 0.9941 |  | | designed | 3 | 6079.20 |  | 0.382 |  | 0.9895 |  | | shown | 3 | 6070.71 |  | 0.672 |  | 0.9981 |  | | ken | 1 | 6035.38 |  | 0.353 |  | 0.9967 |  | | states | 2 | 6031.42 |  | 0.507 |  | 0.9912 |  | | account | 2 | 6032.37 |  | 0.583 |  | 0.9937 |  | | war | 2 | 6033.79 |  | 0.349 |  | 0.9897 |  | | rate | 2 | 6008.44 |  | 0.417 |  | 0.9843 |  | | together | 3 | 6019.91 |  | 0.663 |  | 0.9946 |  | | parts | 2 | 6005.78 |  | 0.541 |  | 0.9864 |  | | stupid | 3 | 5975.69 |  | 0.562 |  | 0.9958 |  | | himself | U | 5962.22 |  | 0.641 |  | 0.9988 |  | | news | 2 | 5960.22 |  | 0.456 |  | 0.9856 |  | | deal | 2 | 5933.03 |  | 0.732 |  | 0.9974 |  | | fit | 2 | 5935.63 |  | 0.572 |  | 0.9888 |  | | conclusion | 2 | 5927.07 |  | 0.598 |  | 0.9944 |  | | men | 2 | 5922.34 |  | 0.448 |  | 0.9921 |  | | considered | 3 | 5916.26 |  | 0.720 |  | 0.9964 |  | | web | 2 | 5904.12 |  | 0.480 |  | 0.9962 |  | | related | 3 | 5880.59 |  | 0.581 |  | 0.9937 |  | | stop | 2 | 5885.55 |  | 0.685 |  | 0.9959 |  | | suggest | 3 | 5873.69 |  | 0.752 |  | 0.9980 |  | | environment | 2 | 5883.00 |  | 0.415 |  | 0.9906 |  | | logic | 2 | 5868.45 |  | 0.512 |  | 0.9915 |  | | designer | 2 | 5857.55 |  | 0.294 |  | 0.9903 |  | | language | 2 | 5861.72 |  | 0.423 |  | 0.9822 |  | | evolutionists | 2 | 5835.13 |  | 0.434 |  | 0.9983 |  | | apparently | 4 | 5820.52 |  | 0.773 |  | 0.9988 |  | | method | 2 | 5809.07 |  | 0.457 |  | 0.9883 |  | | appear | 4 | 5785.56 |  | 0.706 |  | 0.9968 |  | | love | 2 | 5786.29 |  | 0.522 |  | 0.9918 |  | | ok | U | 5782.08 |  | 0.714 |  | 0.9976 |  | | mass | 2 | 5750.88 |  | 0.337 |  | 0.9818 |  | | merely | 4 | 5741.27 |  | 0.730 |  | 0.9993 |  | | anyway | 3 | 5708.38 |  | 0.816 |  | 0.9992 |  | | easy | 3 | 5702.16 |  | 0.708 |  | 0.9982 |  | | sequence | 2 | 5694.26 |  | 0.296 |  | 0.9839 |  | | heard | 3 | 5693.28 |  | 0.702 |  | 0.9978 |  | | thanks | 2 | 5675.03 |  | 0.715 |  | 0.9982 |  | | books | 2 | 5647.19 |  | 0.514 |  | 0.9908 |  | | sun | 2 | 5640.83 |  | 0.388 |  | 0.9852 |  | | told | 3 | 5619.76 |  | 0.664 |  | 0.9962 |  | | goes | U | 5600.07 |  | 0.794 |  | 0.9979 |  | | regards | 2 | 5631.30 |  | 0.514 |  | 0.9970 |  | | atheist | 2 | 5594.76 |  | 0.388 |  | 0.9968 |  | | necessary | 3 | 5577.01 |  | 0.636 |  | 0.9960 |  | | let's | U | 5575.39 |  | 0.688 |  | 0.9962 |  | | especially | 4 | 5576.20 |  | 0.832 |  | 0.9995 |  | | examples | 2 | 5560.58 |  | 0.605 |  | 0.9966 |  | | forms | 2 | 5543.57 |  | 0.510 |  | 0.9914 |  | | results | 2 | 5531.58 |  | 0.588 |  | 0.9954 |  | | peter | 1 | 5514.36 |  | 0.244 |  | 0.9175 |  | | processes | 2 | 5510.40 |  | 0.456 |  | 0.9959 |  | | exists | 2 | 5524.28 |  | 0.592 |  | 0.9974 |  | | reasons | 2 | 5507.37 |  | 0.644 |  | 0.9959 |  | | silberstein | 1 | 5495.53 |  | 0.372 |  | 0.9837 |  | | body | 2 | 5495.14 |  | 0.489 |  | 0.9856 |  | | actual | 3 | 5478.27 |  | 0.701 |  | 0.9971 |  | | hope | 2 | 5470.98 |  | 0.760 |  | 0.9979 |  | | various | 3 | 5466.56 |  | 0.737 |  | 0.9972 |  | | ignorance | 2 | 5471.37 |  | 0.592 |  | 0.9969 |  | | observation | 2 | 5441.50 |  | 0.488 |  | 0.9957 |  | | none | 3 | 5431.48 |  | 0.754 |  | 0.9964 |  | | beyond | 2 | 5428.28 |  | 0.621 |  | 0.9940 |  | | early | 3 | 5416.40 |  | 0.584 |  | 0.9942 |  | | asked | 3 | 5426.62 |  | 0.667 |  | 0.9963 |  | | standard | 2 | 5393.18 |  | 0.534 |  | 0.9929 |  | | field | 2 | 5387.75 |  | 0.515 |  | 0.9911 |  | | taken | 2 | 5375.05 |  | 0.758 |  | 0.9979 |  | | concept | 2 | 5365.65 |  | 0.578 |  | 0.9948 |  | | neither | 4 | 5356.66 |  | 0.738 |  | 0.9978 |  | | children | 2 | 5330.12 |  | 0.423 |  | 0.9938 |  | | obviously | 4 | 5322.61 |  | 0.786 |  | 0.9985 |  | | reference | 2 | 5300.79 |  | 0.579 |  | 0.9923 |  | | personal | 3 | 5305.68 |  | 0.595 |  | 0.9956 |  | | study | 2 | 5285.70 |  | 0.563 |  | 0.9920 |  | | context | 2 | 5278.31 |  | 0.548 |  | 0.9933 |  | | power | 2 | 5268.64 |  | 0.541 |  | 0.9935 |  | | care | 2 | 5251.70 |  | 0.662 |  | 0.9952 |  | | debate | 2 | 5234.14 |  | 0.475 |  | 0.9858 |  | | dr | U | 5228.68 |  | 0.427 |  | 0.9941 |  | | evolved | 3 | 5200.21 |  | 0.498 |  | 0.9986 |  | | site | 2 | 5190.01 |  | 0.440 |  | 0.9925 |  | | including | 3 | 5181.37 |  | 0.806 |  | 0.9988 |  | | during | 2 | 5189.54 |  | 0.626 |  | 0.9969 |  | | due | 2 | 5172.52 |  | 0.661 |  | 0.9960 |  | | following | 2 | 5173.80 |  | 0.727 |  | 0.9986 |  | | events | 2 | 5162.66 |  | 0.493 |  | 0.9921 |  | | chris | 1 | 5153.65 |  | 0.399 |  | 0.9988 |  | | further | 3 | 5144.32 |  | 0.769 |  | 0.9978 |  | | major | 3 | 5139.49 |  | 0.632 |  | 0.9964 |  | | biological | 2 | 5153.18 |  | 0.492 |  | 0.9967 |  | | they're | U | 5138.35 |  | 0.635 |  | 0.9971 |  | | shows | 2 | 5140.41 |  | 0.705 |  | 0.9978 |  | | death | 2 | 5145.93 |  | 0.445 |  | 0.9922 |  | | wasn't | U | 5135.37 |  | 0.745 |  | 0.9987 |  | | stuff | 2 | 5114.26 |  | 0.691 |  | 0.9953 |  | | along | 2 | 5102.25 |  | 0.772 |  | 0.9979 |  | | assume | 3 | 5087.81 |  | 0.676 |  | 0.9968 |  | | genesis | 2 | 5072.79 |  | 0.360 |  | 0.9939 |  | | everyone | 3 | 5072.57 |  | 0.705 |  | 0.9986 |  | | side | 2 | 5068.77 |  | 0.608 |  | 0.9896 |  | | test | 2 | 5030.12 |  | 0.488 |  | 0.9902 |  | | left | 2 | 5038.26 |  | 0.708 |  | 0.9950 |  | | days | 2 | 5008.78 |  | 0.565 |  | 0.9909 |  | | among | 2 | 5002.79 |  | 0.685 |  | 0.9966 |  | | getting | 3 | 4994.11 |  | 0.770 |  | 0.9983 |  | | american | 2 | 4999.64 |  | 0.499 |  | 0.9968 |  | | response | 2 | 4994.97 |  | 0.635 |  | 0.9945 |  | | intelligence | 2 | 4991.08 |  | 0.370 |  | 0.9892 |  | | chance | 2 | 5015.10 |  | 0.514 |  | 0.9870 |  | | written | 2 | 4976.51 |  | 0.612 |  | 0.9964 |  | | points | 2 | 4971.78 |  | 0.649 |  | 0.9931 |  | | source | 2 | 4964.17 |  | 0.575 |  | 0.9928 |  | | million | 3 | 4959.04 |  | 0.432 |  | 0.9883 |  | | current | 3 | 4946.46 |  | 0.650 |  | 0.9957 |  | | mechanism | 2 | 4940.52 |  | 0.424 |  | 0.9975 |  | | gene | 2 | 4938.08 |  | 0.330 |  | 0.9921 |  | | org | U | 4935.22 |  | 0.321 |  | 0.9940 |  | | according | 3 | 4925.72 |  | 0.715 |  | 0.9968 |  | | problems | 2 | 4924.35 |  | 0.647 |  | 0.9948 |  | | page | 2 | 4916.67 |  | 0.507 |  | 0.9957 |  | | list | 2 | 4904.94 |  | 0.507 |  | 0.9874 |  | | expect | 3 | 4892.12 |  | 0.694 |  | 0.9960 |  | | material | 2 | 4880.09 |  | 0.531 |  | 0.9896 |  | | richard | 1 | 4850.68 |  | 0.444 |  | 0.9967 |  | | sorry | U | 4846.09 |  | 0.788 |  | 0.9987 |  | | live | 2 | 4850.07 |  | 0.615 |  | 0.9979 |  | | brain | 2 | 4838.25 |  | 0.378 |  | 0.9787 |  | | rest | 2 | 4827.19 |  | 0.778 |  | 0.9973 |  | | provide | 3 | 4802.01 |  | 0.669 |  | 0.9962 |  | | won't | 4 | 4800.01 |  | 0.695 |  | 0.9948 |  | | produce | 3 | 4805.02 |  | 0.558 |  | 0.9972 |  | | effect | 2 | 4794.41 |  | 0.588 |  | 0.9932 |  | | origin | 2 | 4789.43 |  | 0.508 |  | 0.9955 |  | | later | 3 | 4765.43 |  | 0.709 |  | 0.9983 |  | | looking | 3 | 4761.89 |  | 0.726 |  | 0.9970 |  | | net | 2 | 4801.71 |  | 0.436 |  | 0.9947 |  | | learn | 3 | 4742.07 |  | 0.605 |  | 0.9962 |  | | completely | 4 | 4732.91 |  | 0.758 |  | 0.9985 |  | | function | 2 | 4727.66 |  | 0.334 |  | 0.9839 |  | | beliefs | 2 | 4723.39 |  | 0.481 |  | 0.9973 |  | | terms | 2 | 4708.71 |  | 0.619 |  | 0.9956 |  | | feel | 2 | 4706.01 |  | 0.693 |  | 0.9973 |  | | comes | 2 | 4694.69 |  | 0.762 |  | 0.9963 |  | | obvious | 3 | 4691.72 |  | 0.720 |  | 0.9980 |  | | next | 3 | 4658.02 |  | 0.741 |  | 0.9974 |  | | sent | 3 | 4643.74 |  | 0.390 |  | 0.9702 |  | | remember | 3 | 4634.58 |  | 0.739 |  | 0.9974 |  | | proof | 2 | 4620.72 |  | 0.495 |  | 0.9918 |  | | observed | 3 | 4619.05 |  | 0.486 |  | 0.9980 |  | | meaning | 2 | 4612.48 |  | 0.555 |  | 0.9916 |  | | although | 4 | 4576.81 |  | 0.794 |  | 0.9992 |  | | whatever | 3 | 4579.81 |  | 0.787 |  | 0.9985 |  | | term | 2 | 4578.90 |  | 0.572 |  | 0.9914 |  | | become | 3 | 4554.26 |  | 0.724 |  | 0.9972 |  | | except | 2 | 4546.35 |  | 0.823 |  | 0.9992 |  | | haven't | 4 | 4540.07 |  | 0.764 |  | 0.9986 |  | | address | 2 | 4536.67 |  | 0.571 |  | 0.9981 |  | | happened | 3 | 4508.42 |  | 0.671 |  | 0.9972 |  | | help | 2 | 4501.37 |  | 0.731 |  | 0.9971 |  | | unless | 4 | 4492.06 |  | 0.785 |  | 0.9988 |  | | talkorigins | 1 | 4478.86 |  | 0.270 |  | 0.9912 |  | | guess | 3 | 4471.47 |  | 0.784 |  | 0.9984 |  | | groups | 2 | 4460.72 |  | 0.459 |  | 0.9899 |  | | christians | 2 | 4463.14 |  | 0.410 |  | 0.9980 |  | | public | 2 | 4458.48 |  | 0.480 |  | 0.9966 |  | | genes | 2 | 4444.10 |  | 0.323 |  | 0.9916 |  | | mutations | 2 | 4445.39 |  | 0.284 |  | 0.9854 |  | | mutation | 2 | 4436.74 |  | 0.296 |  | 0.9862 |  | | space | 2 | 4420.21 |  | 0.431 |  | 0.9899 |  | | church | 2 | 4430.76 |  | 0.383 |  | 0.9954 |  | | story | 2 | 4418.34 |  | 0.509 |  | 0.9900 |  | | ones | 3 | 4421.48 |  | 0.728 |  | 0.9950 |  | | upon | 2 | 4422.08 |  | 0.647 |  | 0.9954 |  | | themselves | U | 4418.17 |  | 0.730 |  | 0.9985 |  | | opinion | 2 | 4408.61 |  | 0.577 |  | 0.9942 |  | | paul | 1 | 4387.86 |  | 0.352 |  | 0.9917 |  | | works | 2 | 4382.18 |  | 0.681 |  | 0.9964 |  | | systems | 2 | 4378.51 |  | 0.421 |  | 0.9926 |  | | past | 2 | 4379.26 |  | 0.681 |  | 0.9956 |  | | co | U | 4483.13 |  | 0.291 |  | 0.9754 |  | | almost | 4 | 4372.27 |  | 0.770 |  | 0.9989 |  | | via | 2 | 4355.48 |  | 0.441 |  | 0.9871 |  | | three | 3 | 4351.53 |  | 0.650 |  | 0.9933 |  | | matt | 1 | 4343.16 |  | 0.347 |  | 0.9816 |  | | aren't | U | 4346.44 |  | 0.770 |  | 0.9985 |  | | happen | 3 | 4315.31 |  | 0.696 |  | 0.9972 |  | | www | U | 4412.42 |  | 0.319 |  | 0.9833 |  | | reply | 2 | 4306.84 |  | 0.568 |  | 0.9980 |  | | discussion | 2 | 4302.73 |  | 0.646 |  | 0.9972 |  | | instead | 4 | 4304.50 |  | 0.807 |  | 0.9993 |  | | similar | 3 | 4303.21 |  | 0.664 |  | 0.9954 |  | | model | 2 | 4274.61 |  | 0.407 |  | 0.9895 |  | | you've | U | 4275.88 |  | 0.670 |  | 0.9968 |  | | quote | 2 | 4277.23 |  | 0.500 |  | 0.9879 |  | | year | 2 | 4268.43 |  | 0.567 |  | 0.9930 |  | | specific | 3 | 4263.29 |  | 0.605 |  | 0.9956 |  | | prove | 3 | 4260.45 |  | 0.574 |  | 0.9961 |  | | age | 2 | 4257.38 |  | 0.454 |  | 0.9897 |  | | hand | 2 | 4257.38 |  | 0.718 |  | 0.9953 |  | | organisms | 2 | 4253.05 |  | 0.415 |  | 0.9989 |  | | line | 2 | 4210.73 |  | 0.605 |  | 0.9866 |  | | thinking | 2 | 4211.50 |  | 0.706 |  | 0.9969 |  | | random | 3 | 4195.92 |  | 0.374 |  | 0.9930 |  | | high | 3 | 4191.95 |  | 0.618 |  | 0.9963 |  | | lack | 2 | 4191.64 |  | 0.681 |  | 0.9973 |  | | i'd | U | 4159.61 |  | 0.726 |  | 0.9981 |  | | david | 1 | 4193.63 |  | 0.419 |  | 0.9959 |  | | understanding | 3 | 4151.88 |  | 0.662 |  | 0.9974 |  | | hard | 2 | 4134.28 |  | 0.760 |  | 0.9980 |  | | complex | 2 | 4133.99 |  | 0.460 |  | 0.9956 |  | | clearly | 4 | 4115.14 |  | 0.730 |  | 0.9976 |  | | general | 3 | 4107.73 |  | 0.698 |  | 0.9975 |  | | reading | 2 | 4080.21 |  | 0.725 |  | 0.9979 |  | | energy | 2 | 4051.84 |  | 0.298 |  | 0.9798 |  | | record | 2 | 4048.06 |  | 0.460 |  | 0.9927 |  | | reality | 2 | 4049.27 |  | 0.547 |  | 0.9918 |  | | facts | 2 | 4045.29 |  | 0.539 |  | 0.9923 |  | | hypothesis | 2 | 4024.85 |  | 0.411 |  | 0.9895 |  | | posted | 2 | 4016.77 |  | 0.562 |  | 0.9981 |  | | fossils | 2 | 3999.88 |  | 0.337 |  | 0.9932 |  | | bob | 1 | 3997.52 |  | 0.353 |  | 0.9950 |  | | thus | 4 | 3979.56 |  | 0.695 |  | 0.9981 |  | | yourself | U | 3973.72 |  | 0.687 |  | 0.9991 |  | | wouldn't | 4 | 3974.68 |  | 0.769 |  | 0.9982 |  | | important | 3 | 3972.48 |  | 0.693 |  | 0.9965 |  | | thread | 2 | 3942.55 |  | 0.586 |  | 0.9960 |  | | arguments | 2 | 3921.20 |  | 0.583 |  | 0.9955 |  | | level | 2 | 3924.54 |  | 0.587 |  | 0.9916 |  | | indeed | 4 | 3912.07 |  | 0.776 |  | 0.9994 |  | | came | 2 | 3914.79 |  | 0.692 |  | 0.9972 |  | | cause | 2 | 3914.86 |  | 0.563 |  | 0.9888 |  | | he's | U | 3883.95 |  | 0.580 |  | 0.9933 |  | | result | 2 | 3876.23 |  | 0.666 |  | 0.9973 |  | | doubt | 2 | 3872.06 |  | 0.697 |  | 0.9958 |  | | physical | 2 | 3863.94 |  | 0.468 |  | 0.9897 |  | | interesting | 3 | 3841.63 |  | 0.746 |  | 0.9984 |  | | theories | 2 | 3820.33 |  | 0.475 |  | 0.9958 |  | | ideas | 2 | 3810.34 |  | 0.580 |  | 0.9946 |  | | sort | 2 | 3780.25 |  | 0.775 |  | 0.9985 |  | | existence | 2 | 3773.62 |  | 0.550 |  | 0.9973 |  | | flood | 2 | 3762.52 |  | 0.276 |  | 0.9728 |  | | changes | 2 | 3735.53 |  | 0.469 |  | 0.9939 |  | | school | 2 | 3722.67 |  | 0.438 |  | 0.9905 |  | | physics | 2 | 3714.50 |  | 0.450 |  | 0.9983 |  | | consider | 3 | 3716.54 |  | 0.745 |  | 0.9981 |  | | light | 2 | 3707.68 |  | 0.434 |  | 0.9753 |  | | oh | U | 3703.74 |  | 0.726 |  | 0.9977 |  | | position | 2 | 3686.95 |  | 0.598 |  | 0.9944 |  | | jesus | 1 | 3680.00 |  | 0.320 |  | 0.9894 |  | | note | 2 | 3645.21 |  | 0.725 |  | 0.9978 |  | | laws | 2 | 3630.90 |  | 0.418 |  | 0.9923 |  | | issue | 2 | 3630.27 |  | 0.659 |  | 0.9972 |  | | likely | 4 | 3628.21 |  | 0.691 |  | 0.9975 |  | | nor | 4 | 3638.18 |  | 0.718 |  | 0.9964 |  | | today | 3 | 3619.99 |  | 0.664 |  | 0.9985 |  | | itself | U | 3617.60 |  | 0.719 |  | 0.9984 |  | | clear | 3 | 3605.37 |  | 0.752 |  | 0.9989 |  | | original | 3 | 3599.16 |  | 0.641 |  | 0.9953 |  | | posts | 2 | 3594.28 |  | 0.489 |  | 0.9868 |  | | bad | 3 | 3598.32 |  | 0.663 |  | 0.9969 |  | | population | 2 | 3574.69 |  | 0.381 |  | 0.9906 |  | | pretty | 3 | 3564.83 |  | 0.786 |  | 0.9991 |  | | second | 3 | 3563.95 |  | 0.659 |  | 0.9937 |  | | false | 3 | 3565.55 |  | 0.570 |  | 0.9951 |  | | set | 2 | 3566.32 |  | 0.657 |  | 0.9934 |  | | animals | 2 | 3559.60 |  | 0.421 |  | 0.9952 |  | | ask | 3 | 3554.05 |  | 0.726 |  | 0.9973 |  | | often | 3 | 3551.42 |  | 0.746 |  | 0.9982 |  | | i'll | U | 3543.54 |  | 0.737 |  | 0.9980 |  | | large | 3 | 3541.53 |  | 0.645 |  | 0.9972 |  | | therefore | 4 | 3541.31 |  | 0.693 |  | 0.9982 |  | | questions | 2 | 3537.77 |  | 0.577 |  | 0.9927 |  | | her | U | 3598.15 |  | 0.383 |  | 0.9775 |  | | single | 3 | 3519.38 |  | 0.637 |  | 0.9961 |  | | everything | 3 | 3499.91 |  | 0.705 |  | 0.9977 |  | | small | 3 | 3494.75 |  | 0.638 |  | 0.9966 |  | | subject | 2 | 3497.14 |  | 0.707 |  | 0.9970 |  | | certain | 3 | 3483.38 |  | 0.718 |  | 0.9972 |  | | start | 2 | 3479.28 |  | 0.690 |  | 0.9975 |  | | mark | 1 | 3477.54 |  | 0.478 |  | 0.9967 |  | | she | U | 3495.81 |  | 0.366 |  | 0.9693 |  | | genetic | 3 | 3457.28 |  | 0.402 |  | 0.9971 |  | | dna | 2 | 3440.95 |  | 0.321 |  | 0.9911 |  | | difference | 2 | 3435.56 |  | 0.667 |  | 0.9958 |  | | call | 2 | 3437.25 |  | 0.727 |  | 0.9969 |  | | belief | 2 | 3405.27 |  | 0.485 |  | 0.9971 |  | | several | 3 | 3399.69 |  | 0.779 |  | 0.9992 |  | | darwin | 1 | 3413.70 |  | 0.354 |  | 0.9906 |  | | living | 2 | 3408.55 |  | 0.563 |  | 0.9958 |  | | until | 2 | 3396.83 |  | 0.768 |  | 0.9981 |  | | present | 2 | 3388.23 |  | 0.684 |  | 0.9980 |  | | end | 2 | 3368.99 |  | 0.717 |  | 0.9972 |  | | accept | 3 | 3363.38 |  | 0.630 |  | 0.9977 |  | | keep | 3 | 3343.16 |  | 0.769 |  | 0.9982 |  | | away | 3 | 3327.79 |  | 0.725 |  | 0.9982 |  | | email | 2 | 3313.04 |  | 0.502 |  | 0.9996 |  | | bit | 2 | 3296.72 |  | 0.792 |  | 0.9985 |  | | within | 2 | 3298.00 |  | 0.660 |  | 0.9971 |  | | modern | 3 | 3294.93 |  | 0.537 |  | 0.9945 |  | | water | 2 | 3287.45 |  | 0.378 |  | 0.9820 |  | | whole | 3 | 3281.92 |  | 0.787 |  | 0.9986 |  | | particular | 3 | 3277.53 |  | 0.713 |  | 0.9976 |  | | called | 2 | 3267.57 |  | 0.737 |  | 0.9972 |  | | view | 2 | 3264.42 |  | 0.632 |  | 0.9967 |  | | explanation | 2 | 3244.27 |  | 0.551 |  | 0.9959 |  | | biology | 2 | 3247.84 |  | 0.508 |  | 0.9990 |  | | simple | 3 | 3234.02 |  | 0.691 |  | 0.9975 |  | | fossil | 2 | 3215.85 |  | 0.354 |  | 0.9913 |  | | there's | U | 3205.69 |  | 0.717 |  | 0.9985 |  | | able | 3 | 3195.37 |  | 0.769 |  | 0.9981 |  | | exactly | 4 | 3174.17 |  | 0.792 |  | 0.9990 |  | | talking | 3 | 3152.36 |  | 0.709 |  | 0.9972 |  | | order | 2 | 3148.48 |  | 0.668 |  | 0.9951 |  | | faith | 2 | 3147.18 |  | 0.412 |  | 0.9939 |  | | doing | 4 | 3137.28 |  | 0.754 |  | 0.9986 |  | | created | 3 | 3137.58 |  | 0.518 |  | 0.9971 |  | | google | 1 | 3206.00 |  | 0.289 |  | 0.9696 |  | | statement | 2 | 3124.38 |  | 0.612 |  | 0.9952 |  | | correct | 3 | 3100.27 |  | 0.730 |  | 0.9982 |  | | let | 4 | 3067.43 |  | 0.771 |  | 0.9978 |  | | having | 4 | 3037.96 |  | 0.802 |  | 0.9987 |  | | making | 3 | 3010.55 |  | 0.795 |  | 0.9991 |  | | once | 3 | 2980.49 |  | 0.812 |  | 0.9990 |  | | day | 2 | 2969.65 |  | 0.598 |  | 0.9922 |  | | research | 2 | 2965.67 |  | 0.551 |  | 0.9982 |  | | law | 2 | 2938.68 |  | 0.408 |  | 0.9900 |  | | known | 3 | 2932.13 |  | 0.674 |  | 0.9965 |  | | knowledge | 2 | 2932.88 |  | 0.584 |  | 0.9948 |  | | name | 2 | 2927.66 |  | 0.587 |  | 0.9926 |  | | wilkins | 1 | 2917.68 |  | 0.352 |  | 0.9976 |  | | times | 2 | 2923.34 |  | 0.733 |  | 0.9966 |  | | exist | 2 | 2916.46 |  | 0.600 |  | 0.9973 |  | | ago | 3 | 2914.13 |  | 0.684 |  | 0.9976 |  | | certainly | 4 | 2899.41 |  | 0.796 |  | 0.9993 |  | | under | 2 | 2897.33 |  | 0.723 |  | 0.9980 |  | | message | 2 | 2892.13 |  | 0.519 |  | 0.9993 |  | | maybe | 4 | 2885.17 |  | 0.727 |  | 0.9968 |  | | truth | 2 | 2886.04 |  | 0.533 |  | 0.9964 |  | | done | 4 | 2870.03 |  | 0.762 |  | 0.9982 |  | | place | 2 | 2868.60 |  | 0.766 |  | 0.9979 |  | | free | 3 | 2845.90 |  | 0.510 |  | 0.9924 |  | | person | 2 | 2827.76 |  | 0.630 |  | 0.9978 |  | | process | 2 | 2808.47 |  | 0.568 |  | 0.9972 |  | | claims | 2 | 2799.53 |  | 0.590 |  | 0.9970 |  | | form | 2 | 2796.36 |  | 0.670 |  | 0.9972 |  | | state | 2 | 2785.43 |  | 0.572 |  | 0.9930 |  | | else | 4 | 2764.40 |  | 0.818 |  | 0.9996 |  | | deja | 1 | 2753.12 |  | 0.232 |  | 0.8732 |  | | intelligent | 3 | 2750.85 |  | 0.397 |  | 0.9952 |  | | already | 3 | 2748.85 |  | 0.772 |  | 0.9988 |  | | put | 2 | 2747.96 |  | 0.799 |  | 0.9991 |  | | agree | 3 | 2741.45 |  | 0.719 |  | 0.9991 |  | | using | 3 | 2736.01 |  | 0.749 |  | 0.9987 |  | | history | 2 | 2731.56 |  | 0.607 |  | 0.9975 |  | | etc | U | 2726.19 |  | 0.670 |  | 0.9948 |  | | university | 2 | 2726.61 |  | 0.530 |  | 0.9997 |  | | seen | 3 | 2722.39 |  | 0.751 |  | 0.9982 |  | | definition | 2 | 2711.87 |  | 0.470 |  | 0.9957 |  | | christian | 2 | 2704.80 |  | 0.437 |  | 0.9988 |  | | lot | 2 | 2692.46 |  | 0.761 |  | 0.9988 |  | | big | 3 | 2682.45 |  | 0.617 |  | 0.9934 |  | | mind | 2 | 2677.90 |  | 0.703 |  | 0.9968 |  | | data | 2 | 2650.21 |  | 0.433 |  | 0.9907 |  | | trying | 3 | 2637.88 |  | 0.778 |  | 0.9986 |  | | words | 2 | 2636.89 |  | 0.671 |  | 0.9972 |  | | great | 3 | 2627.41 |  | 0.720 |  | 0.9983 |  | | id | U | 2616.42 |  | 0.292 |  | 0.9785 |  | | group | 2 | 2603.87 |  | 0.547 |  | 0.9932 |  | | kind | 2 | 2603.69 |  | 0.710 |  | 0.9965 |  | | best | 3 | 2555.71 |  | 0.708 |  | 0.9970 |  | | last | 3 | 2547.79 |  | 0.755 |  | 0.9988 |  | | support | 2 | 2533.91 |  | 0.653 |  | 0.9980 |  | | got | 2 | 2485.14 |  | 0.762 |  | 0.9990 |  | | htm | U | 2441.93 |  | 0.355 |  | 0.9936 |  | | off | 2 | 2440.98 |  | 0.766 |  | 0.9994 |  | | information | 2 | 2414.30 |  | 0.480 |  | 0.9765 |  | | creationism | 2 | 2410.18 |  | 0.424 |  | 0.9983 |  | | based | 2 | 2402.34 |  | 0.705 |  | 0.9983 |  | | probably | 4 | 2375.66 |  | 0.789 |  | 0.9992 |  | | given | 3 | 2368.77 |  | 0.775 |  | 0.9988 |  | | humans | 2 | 2348.49 |  | 0.458 |  | 0.9970 |  | | sense | 2 | 2327.86 |  | 0.700 |  | 0.9979 |  | | others | 3 | 2322.27 |  | 0.780 |  | 0.9991 |  | | design | 2 | 2313.47 |  | 0.329 |  | 0.9889 |  | | less | 3 | 2311.80 |  | 0.750 |  | 0.9980 |  | | anyone | 3 | 2311.31 |  | 0.772 |  | 0.9997 |  | | always | 3 | 2305.38 |  | 0.764 |  | 0.9982 |  | | please | U | 2297.63 |  | 0.676 |  | 0.9971 |  | | down | 3 | 2281.37 |  | 0.742 |  | 0.9983 |  | | each | 3 | 2259.12 |  | 0.643 |  | 0.9940 |  | | word | 2 | 2253.72 |  | 0.612 |  | 0.9950 |  | | possible | 3 | 2247.39 |  | 0.717 |  | 0.9985 |  | | evolutionary | 3 | 2243.01 |  | 0.490 |  | 0.9991 |  | | nature | 2 | 2244.28 |  | 0.577 |  | 0.9960 |  | | try | 2 | 2237.43 |  | 0.799 |  | 0.9992 |  | | makes | 3 | 2230.06 |  | 0.822 |  | 0.9994 |  | | simply | 4 | 2203.69 |  | 0.770 |  | 0.9997 |  | | perhaps | 4 | 2199.08 |  | 0.784 |  | 0.9990 |  | | someone | 3 | 2198.29 |  | 0.741 |  | 0.9989 |  | | says | 3 | 2198.45 |  | 0.685 |  | 0.9976 |  | | against | 2 | 2195.41 |  | 0.682 |  | 0.9988 |  | | saying | 3 | 2193.01 |  | 0.727 |  | 0.9989 |  | | creationist | 2 | 2188.05 |  | 0.506 |  | 0.9996 |  | | number | 2 | 2178.46 |  | 0.606 |  | 0.9924 |  | | seem | 4 | 2163.01 |  | 0.811 |  | 0.9995 |  | | give | 2 | 2155.29 |  | 0.795 |  | 0.9991 |  | | explain | 3 | 2154.69 |  | 0.655 |  | 0.9990 |  | | whether | 4 | 2150.08 |  | 0.703 |  | 0.9975 |  | | while | 2 | 2147.19 |  | 0.825 |  | 0.9997 |  | | though | 4 | 2123.74 |  | 0.824 |  | 0.9997 |  | | old | 3 | 2122.96 |  | 0.646 |  | 0.9973 |  | | i've | U | 2122.00 |  | 0.706 |  | 0.9983 |  | | above | 2 | 2115.56 |  | 0.748 |  | 0.9995 |  | | book | 2 | 2114.26 |  | 0.526 |  | 0.9950 |  | | selection | 2 | 2130.11 |  | 0.359 |  | 0.9941 |  | | around | 2 | 2104.21 |  | 0.767 |  | 0.9991 |  | | thought | 2 | 2088.27 |  | 0.777 |  | 0.9990 |  | | religious | 2 | 2077.33 |  | 0.472 |  | 0.9993 |  | | means | 3 | 2078.77 |  | 0.751 |  | 0.9981 |  | | through | 2 | 2073.70 |  | 0.750 |  | 0.9979 |  | | scientists | 2 | 2072.46 |  | 0.511 |  | 0.9995 |  | | quite | 4 | 2067.57 |  | 0.816 |  | 0.9994 |  | | common | 2 | 2060.94 |  | 0.543 |  | 0.9926 |  | | idea | 2 | 2057.41 |  | 0.742 |  | 0.9977 |  | | show | 2 | 2051.14 |  | 0.698 |  | 0.9977 |  | | answer | 2 | 2050.88 |  | 0.629 |  | 0.9955 |  | | creation | 2 | 2045.49 |  | 0.457 |  | 0.9989 |  | | cannot | 4 | 2040.77 |  | 0.677 |  | 0.9991 |  | | yet | 4 | 2028.99 |  | 0.813 |  | 0.9995 |  | | real | 3 | 2024.30 |  | 0.705 |  | 0.9966 |  | | ever | 3 | 2014.79 |  | 0.788 |  | 0.9991 |  | | system | 2 | 1998.54 |  | 0.475 |  | 0.9907 |  | | few | 3 | 1986.46 |  | 0.791 |  | 0.9996 |  | | little | 3 | 1986.22 |  | 0.786 |  | 0.9988 |  | | creationists | 2 | 1980.96 |  | 0.488 |  | 0.9992 |  | | found | 3 | 1973.45 |  | 0.670 |  | 0.9977 |  | | seems | 4 | 1963.64 |  | 0.796 |  | 0.9996 |  | | reason | 2 | 1941.16 |  | 0.768 |  | 0.9986 |  | | either | 4 | 1935.95 |  | 0.851 |  | 0.9997 |  | | going | U | 1935.80 |  | 0.775 |  | 0.9988 |  | | enough | 3 | 1930.14 |  | 0.787 |  | 0.9986 |  | | sure | 3 | 1903.75 |  | 0.833 |  | 0.9997 |  | | far | 3 | 1904.35 |  | 0.819 |  | 0.9992 |  | | tell | 3 | 1887.97 |  | 0.756 |  | 0.9987 |  | | every | 3 | 1885.39 |  | 0.724 |  | 0.9962 |  | | matter | 2 | 1876.80 |  | 0.684 |  | 0.9952 |  | | religion | 2 | 1873.01 |  | 0.414 |  | 0.9984 |  | | least | 3 | 1863.05 |  | 0.861 |  | 0.9998 |  | | didn't | 4 | 1847.80 |  | 0.750 |  | 0.9990 |  | | however | 3 | 1843.80 |  | 0.822 |  | 0.9999 |  | | origins | 2 | 1820.34 |  | 0.548 |  | 0.9996 |  | | used | 3 | 1819.37 |  | 0.736 |  | 0.9980 |  | | man | 2 | 1819.84 |  | 0.526 |  | 0.9961 |  | | better | 3 | 1817.47 |  | 0.773 |  | 0.9989 |  | | come | 2 | 1816.18 |  | 0.808 |  | 0.9993 |  | | back | 2 | 1805.68 |  | 0.793 |  | 0.9990 |  | | isn't | U | 1795.34 |  | 0.778 |  | 0.9994 |  | | long | 3 | 1788.72 |  | 0.784 |  | 0.9990 |  | | rather | 4 | 1785.15 |  | 0.815 |  | 0.9998 |  | | both | 3 | 1758.51 |  | 0.786 |  | 0.9983 |  | | part | 2 | 1755.38 |  | 0.780 |  | 0.9977 |  | | change | 2 | 1737.96 |  | 0.578 |  | 0.9955 |  | | understand | 3 | 1737.35 |  | 0.709 |  | 0.9985 |  | | case | 2 | 1736.27 |  | 0.773 |  | 0.9986 |  | | universe | 2 | 1718.14 |  | 0.391 |  | 0.9932 |  | | argument | 2 | 1704.27 |  | 0.588 |  | 0.9968 |  | | problem | 2 | 1699.46 |  | 0.714 |  | 0.9981 |  | | post | 2 | 1693.29 |  | 0.622 |  | 0.9968 |  | | bible | 1 | 1673.46 |  | 0.374 |  | 0.9923 |  | | work | 2 | 1656.71 |  | 0.724 |  | 0.9977 |  | | yes | U | 1655.12 |  | 0.770 |  | 0.9991 |  | | example | 2 | 1648.37 |  | 0.745 |  | 0.9999 |  | | look | 2 | 1630.65 |  | 0.771 |  | 0.9990 |  | | need | 2 | 1603.87 |  | 0.769 |  | 0.9987 |  | | thing | 2 | 1602.86 |  | 0.821 |  | 0.9995 |  | | talk | 2 | 1588.68 |  | 0.599 |  | 0.9988 |  | | snip | 2 | 1557.76 |  | 0.576 |  | 0.9968 |  | | com | U | 1558.04 |  | 0.408 |  | 0.9873 |  | | john | 1 | 1553.60 |  | 0.464 |  | 0.9988 |  | | you're | U | 1546.23 |  | 0.650 |  | 0.9985 |  | | natural | 3 | 1554.23 |  | 0.468 |  | 0.9989 |  | | take | 2 | 1521.84 |  | 0.823 |  | 0.9996 |  | | another | 3 | 1519.90 |  | 0.839 |  | 0.9993 |  | | without | 2 | 1507.52 |  | 0.772 |  | 0.9988 |  | | after | 2 | 1501.11 |  | 0.787 |  | 0.9995 |  | | again | 3 | 1482.46 |  | 0.779 |  | 0.9991 |  | | anything | 3 | 1477.59 |  | 0.812 |  | 0.9996 |  | | nothing | 3 | 1467.33 |  | 0.771 |  | 0.9993 |  | | human | 2 | 1460.34 |  | 0.530 |  | 0.9982 |  | | own | 2 | 1452.54 |  | 0.732 |  | 0.9997 |  | | mean | 2 | 1441.37 |  | 0.756 |  | 0.9988 |  | | claim | 2 | 1428.05 |  | 0.623 |  | 0.9985 |  | | him | U | 1416.40 |  | 0.576 |  | 0.9982 |  | | course | 2 | 1412.72 |  | 0.837 |  | 0.9999 |  | | must | 4 | 1408.26 |  | 0.755 |  | 0.9980 |  | | can't | 4 | 1404.31 |  | 0.763 |  | 0.9992 |  | | want | 4 | 1402.96 |  | 0.740 |  | 0.9983 |  | | find | 2 | 1388.12 |  | 0.784 |  | 0.9988 |  | | made | 3 | 1387.92 |  | 0.777 |  | 0.9990 |  | | go | U | 1388.08 |  | 0.794 |  | 0.9994 |  | | use | 2 | 1367.54 |  | 0.741 |  | 0.9988 |  | | too | 4 | 1366.18 |  | 0.801 |  | 0.9995 |  | | true | 3 | 1357.86 |  | 0.719 |  | 0.9978 |  | | question | 2 | 1345.01 |  | 0.689 |  | 0.9976 |  | | between | 2 | 1337.24 |  | 0.725 |  | 0.9988 |  | | might | 4 | 1334.04 |  | 0.771 |  | 0.9988 |  | | world | 2 | 1323.59 |  | 0.637 |  | 0.9989 |  | | species | 2 | 1301.03 |  | 0.369 |  | 0.9947 |  | | actually | 4 | 1295.55 |  | 0.822 |  | 0.9998 |  | | earth | 2 | 1273.92 |  | 0.463 |  | 0.9971 |  | | wrong | 3 | 1270.51 |  | 0.690 |  | 0.9982 |  | | never | 3 | 1264.31 |  | 0.777 |  | 0.9991 |  | | different | 3 | 1242.80 |  | 0.675 |  | 0.9974 |  | | before | 2 | 1223.17 |  | 0.759 |  | 0.9997 |  | | two | 3 | 1206.19 |  | 0.738 |  | 0.9979 |  | | new | 3 | 1203.48 |  | 0.648 |  | 0.9972 |  | | right | 2 | 1200.19 |  | 0.761 |  | 0.9986 |  | | still | 3 | 1199.25 |  | 0.823 |  | 0.9993 |  | | doesn't | 4 | 1191.19 |  | 0.772 |  | 0.9996 |  | | am | U | 1175.49 |  | 0.690 |  | 0.9979 |  | | that's | U | 1173.21 |  | 0.757 |  | 0.9999 |  | | over | 2 | 1166.84 |  | 0.765 |  | 0.9982 |  | | since | 4 | 1124.40 |  | 0.818 |  | 0.9998 |  | | said | 3 | 1124.11 |  | 0.712 |  | 0.9975 |  | | things | 2 | 1121.45 |  | 0.724 |  | 0.9987 |  | | first | 3 | 1111.62 |  | 0.783 |  | 0.9988 |  | | scientific | 3 | 1095.16 |  | 0.515 |  | 0.9992 |  | | believe | 3 | 1078.51 |  | 0.652 |  | 0.9986 |  | | its | U | 1072.25 |  | 0.714 |  | 0.9982 |  | | really | 4 | 1062.60 |  | 0.810 |  | 0.9995 |  | | good | 3 | 1062.89 |  | 0.775 |  | 0.9991 |  | | something | 3 | 1054.39 |  | 0.794 |  | 0.9995 |  | | our | U | 1034.01 |  | 0.606 |  | 0.9963 |  | | html | U | 1011.34 |  | 0.392 |  | 0.9960 |  | | fact | 2 | 1012.15 |  | 0.756 |  | 0.9991 |  | | read | 2 | 979.42 |  | 0.734 |  | 0.9997 |  | | may | 4 | 976.78 |  | 0.761 |  | 0.9980 |  | | years | 2 | 968.95 |  | 0.614 |  | 0.9983 |  | | us | U | 932.15 |  | 0.694 |  | 0.9977 |  | | where | 3 | 930.12 |  | 0.802 |  | 0.9992 |  | | here | 3 | 923.33 |  | 0.814 |  | 0.9998 |  | | most | 3 | 920.06 |  | 0.806 |  | 0.9997 |  | | point | 2 | 919.61 |  | 0.761 |  | 0.9996 |  | | life | 2 | 910.63 |  | 0.519 |  | 0.9971 |  | | should | 4 | 905.49 |  | 0.788 |  | 0.9988 |  | | i'm | U | 883.67 |  | 0.707 |  | 0.9984 |  | | same | 3 | 877.99 |  | 0.784 |  | 0.9988 |  | | much | 3 | 870.11 |  | 0.832 |  | 0.9998 |  | | into | 2 | 863.35 |  | 0.813 |  | 0.9995 |  | | get | 2 | 854.08 |  | 0.791 |  | 0.9998 |  | | being | U | 848.98 |  | 0.814 |  | 0.9995 |  | | many | 3 | 842.89 |  | 0.782 |  | 0.9993 |  | | make | 3 | 842.60 |  | 0.829 |  | 0.9997 |  | | those | U | 824.90 |  | 0.813 |  | 0.9993 |  | | now | 3 | 824.23 |  | 0.824 |  | 0.9998 |  | | well | U | 823.18 |  | 0.866 |  | 1.0000 |  | | theory | 2 | 820.08 |  | 0.459 |  | 0.9984 |  | | also | 4 | 819.89 |  | 0.851 |  | 0.9999 |  | | very | 4 | 812.85 |  | 0.806 |  | 0.9992 |  | | way | 2 | 803.00 |  | 0.838 |  | 0.9999 |  | | had | 4 | 765.50 |  | 0.720 |  | 0.9989 |  | | such | 3 | 755.53 |  | 0.789 |  | 0.9997 |  | | did | 4 | 748.09 |  | 0.741 |  | 0.9990 |  | | say | 3 | 745.14 |  | 0.797 |  | 0.9996 |  | | these | U | 743.02 |  | 0.754 |  | 0.9994 |  | | could | 4 | 688.20 |  | 0.793 |  | 0.9991 |  | | see | 3 | 628.25 |  | 0.819 |  | 0.9997 |  | | up | 2 | 617.32 |  | 0.828 |  | 0.9999 |  | | because | 4 | 615.31 |  | 0.802 |  | 0.9998 |  | | time | 2 | 607.96 |  | 0.773 |  | 0.9989 |  | | even | 3 | 602.23 |  | 0.856 |  | 1.0000 |  | | article | 2 | 599.37 |  | 0.507 |  | 0.9789 |  | | people | 2 | 599.20 |  | 0.633 |  | 0.9995 |  | | it's | U | 587.52 |  | 0.693 |  | 0.9999 |  | | god | 1 | 585.90 |  | 0.394 |  | 0.9892 |  | | does | 4 | 584.65 |  | 0.776 |  | 0.9997 |  | | why | 4 | 580.15 |  | 0.745 |  | 0.9993 |  | | them | U | 576.76 |  | 0.779 |  | 0.9993 |  | | been | U | 553.80 |  | 0.802 |  | 0.9998 |  | | out | 3 | 543.32 |  | 0.851 |  | 0.9999 |  | | evidence | 2 | 538.44 |  | 0.519 |  | 0.9988 |  | | science | 2 | 537.67 |  | 0.463 |  | 0.9956 |  | | then | 4 | 537.23 |  | 0.812 |  | 0.9999 |  | | me | 1 | 531.17 |  | 0.724 |  | 0.9996 |  | | know | 3 | 525.13 |  | 0.768 |  | 0.9994 |  | | when | 3 | 524.54 |  | 0.827 |  | 0.9997 |  | | were | U | 519.16 |  | 0.726 |  | 0.9988 |  | | only | 4 | 518.54 |  | 0.863 |  | 1.0000 |  | | think | 3 | 512.33 |  | 0.772 |  | 0.9999 |  | | their | U | 509.71 |  | 0.693 |  | 0.9991 |  | | his | U | 504.01 |  | 0.576 |  | 0.9990 |  | | other | 3 | 493.86 |  | 0.852 |  | 0.9999 |  | | than | 4 | 489.80 |  | 0.843 |  | 0.9999 |  | | like | 3 | 479.69 |  | 0.824 |  | 0.9999 |  | | evolution | 2 | 476.01 |  | 0.402 |  | 0.9669 |  | | who | 3 | 469.26 |  | 0.679 |  | 0.9987 |  | | will | 4 | 453.93 |  | 0.706 |  | 0.9959 |  | | how | 3 | 448.61 |  | 0.793 |  | 0.9995 |  | | just | 3 | 430.49 |  | 0.824 |  | 0.9997 |  | | don't | 4 | 429.27 |  | 0.754 |  | 0.9998 |  | | any | 3 | 422.09 |  | 0.825 |  | 0.9998 |  | | wrote | 2 | 416.52 |  | 0.546 |  | 0.9808 |  | | some | 3 | 403.22 |  | 0.822 |  | 0.9998 |  | | my | U | 395.02 |  | 0.687 |  | 0.9990 |  | | more | 3 | 385.94 |  | 0.845 |  | 0.9998 |  | | has | 4 | 374.77 |  | 0.830 |  | 0.9998 |  | | which | U | 350.19 |  | 0.794 |  | 0.9999 |  | | he | U | 318.38 |  | 0.535 |  | 0.9952 |  | | about | 2 | 299.48 |  | 0.805 |  | 0.9998 |  | | can | 4 | 295.89 |  | 0.798 |  | 0.9998 |  | | so | 4 | 294.44 |  | 0.867 |  | 0.9998 |  | | one | 3 | 290.03 |  | 0.842 |  | 0.9998 |  | | would | 4 | 283.46 |  | 0.769 |  | 0.9987 |  | | at | 2 | 278.63 |  | 0.849 |  | 0.9999 |  | | do | 4 | 274.93 |  | 0.781 |  | 0.9998 |  | | we | U | 273.95 |  | 0.656 |  | 0.9951 |  | | there | 3 | 271.21 |  | 0.789 |  | 0.9997 |  | | no | 4 | 268.65 |  | 0.805 |  | 1.0000 |  | | an | 4 | 266.84 |  | 0.858 |  | 0.9999 |  | | all | 3 | 259.37 |  | 0.847 |  | 0.9999 |  | | your | U | 258.98 |  | 0.610 |  | 0.9986 |  | | from | 2 | 245.90 |  | 0.831 |  | 0.9999 |  | | by | 2 | 232.80 |  | 0.839 |  | 0.9999 |  | | http | U | 229.82 |  | 0.425 |  | 0.9953 |  | | they | U | 229.30 |  | 0.712 |  | 0.9966 |  | | what | 3 | 225.18 |  | 0.779 |  | 0.9997 |  | | was | U | 218.86 |  | 0.691 |  | 0.9986 |  | | if | 4 | 212.21 |  | 0.804 |  | 0.9983 |  | | or | 4 | 194.92 |  | 0.818 |  | 0.9998 |  | | but | 4 | 192.09 |  | 0.871 |  | 0.9955 |  | | on | 2 | 184.48 |  | 0.848 |  | 0.9995 |  | | with | 2 | 182.70 |  | 0.877 |  | 0.9996 |  | | this | U | 153.55 |  | 0.804 |  | 0.9966 |  | | as | 4 | 149.84 |  | 0.818 |  | 0.9985 |  | | be | U | 144.01 |  | 0.837 |  | 0.9988 |  | | have | 4 | 143.99 |  | 0.821 |  | 0.9983 |  | | for | 2 | 136.28 |  | 0.861 |  | 0.9989 |  | | are | U | 122.30 |  | 0.767 |  | 0.9979 |  | | not | 4 | 115.34 |  | 0.759 |  | 0.9893 |  | | it | U | 83.48 |  | 0.770 |  | 0.9933 |  | | i | U | 82.08 |  | 0.599 |  | 0.9821 |  | | you | U | 71.01 |  | 0.524 |  | 0.9674 |  | | in | 2 | 59.25 |  | 0.760 |  | 0.9618 |  | | is | U | 53.00 |  | 0.659 |  | 0.9434 |  | | that | U | 50.63 |  | 0.635 |  | 0.9029 |  | | and | 4 | 50.16 |  | 0.748 |  | 0.9487 |  | | a | 4 | 46.94 |  | 0.765 |  | 0.9712 |  | | to | 4 | 38.98 |  | 0.669 |  | 0.8840 |  | | of | 2 | 34.77 |  | 0.577 |  | 0.7815 |  | | the | 4 | 20.42 |  | 0.491 |  | 0.6026 |  | |
