# Supplementary material for: Beyond Word Frequency: Bursts, Lulls, and Scaling in the Temporal Distributions of Words
Source: PLoS One. 2009 Nov 11;4(11):e7678. doi: 10.1371/journal.pone.0007678 (PMC2770836; doi:10.1371/journal.pone.0007678)
Supplement: Table S1 — Detailed information on the statistical analysis of all words that were studied (six databases). (31.88 MB TAR) [file pone.0007678.s002.tar › recurrence/wrnpc/index.html]

Word < tau > | ------ | beta | ----- | R^2 | ----- || body | 5642.95 |  | 0.587 |  | 0.9808 |  | | business | 5642.95 |  | 0.600 |  | 0.9896 |  | | captain | 5642.95 |  | 0.319 |  | 0.8898 |  | | galloped | 5642.95 |  | 0.376 |  | 0.9699 |  | | however | 5642.95 |  | 0.605 |  | 0.9848 |  | | run | 5642.95 |  | 0.668 |  | 0.9973 |  | | thin | 5642.95 |  | 0.559 |  | 0.9929 |  | | wait | 5642.95 |  | 0.578 |  | 0.9820 |  | | aim | 5587.08 |  | 0.363 |  | 0.9840 |  | | child | 5587.08 |  | 0.445 |  | 0.9841 |  | | country | 5587.08 |  | 0.594 |  | 0.9916 |  | | decided | 5587.08 |  | 0.652 |  | 0.9696 |  | | guns | 5587.08 |  | 0.307 |  | 0.9088 |  | | legs | 5587.08 |  | 0.569 |  | 0.9884 |  | | ought | 5587.08 |  | 0.640 |  | 0.9817 |  | | dress | 5532.30 |  | 0.460 |  | 0.9946 |  | | remembered | 5532.30 |  | 0.549 |  | 0.9789 |  | | tomorrow | 5532.30 |  | 0.448 |  | 0.9896 |  | | considered | 5478.59 |  | 0.872 |  | 0.9952 |  | | emperor's | 5478.59 |  | 0.445 |  | 0.9685 |  | | four | 5478.59 |  | 0.587 |  | 0.9928 |  | | mamma | 5478.59 |  | 0.371 |  | 0.9664 |  | | rostopchin | 5478.59 |  | 0.304 |  | 0.7989 |  | | shouting | 5478.59 |  | 0.476 |  | 0.9936 |  | | smolensk | 5478.59 |  | 0.406 |  | 0.9803 |  | | colonel | 5425.91 |  | 0.318 |  | 0.9303 |  | | generals | 5425.91 |  | 0.501 |  | 0.9892 |  | | straight | 5425.91 |  | 0.869 |  | 0.9931 |  | | turn | 5425.91 |  | 0.689 |  | 0.9855 |  | | campaign | 5374.24 |  | 0.496 |  | 0.9926 |  | | close | 5374.24 |  | 0.784 |  | 0.9958 |  | | man's | 5374.24 |  | 0.490 |  | 0.9843 |  | | running | 5374.24 |  | 0.639 |  | 0.9984 |  | | silence | 5374.24 |  | 0.674 |  | 0.9886 |  | | asking | 5323.54 |  | 0.855 |  | 0.9885 |  | | attack | 5323.54 |  | 0.451 |  | 0.9876 |  | | beyond | 5323.54 |  | 0.499 |  | 0.9902 |  | | children | 5323.54 |  | 0.540 |  | 0.9923 |  | | hussars | 5323.54 |  | 0.334 |  | 0.9395 |  | | i'm | 5323.54 |  | 0.508 |  | 0.9961 |  | | late | 5323.54 |  | 0.704 |  | 0.9898 |  | | meet | 5323.54 |  | 0.679 |  | 0.9937 |  | | raised | 5323.54 |  | 0.874 |  | 0.9954 |  | | shoulders | 5323.54 |  | 0.720 |  | 0.9949 |  | | able | 5273.79 |  | 0.852 |  | 0.9985 |  | | company | 5273.79 |  | 0.432 |  | 0.9886 |  | | giving | 5273.79 |  | 0.954 |  | 0.9917 |  | | need | 5273.79 |  | 0.700 |  | 0.9938 |  | | borodino | 5224.95 |  | 0.368 |  | 0.9432 |  | | ground | 5224.95 |  | 0.528 |  | 0.9911 |  | | handsome | 5224.95 |  | 0.635 |  | 0.9984 |  | | making | 5224.95 |  | 1.019 |  | 0.9949 |  | | rest | 5224.95 |  | 0.928 |  | 0.9927 |  | | activity | 5177.02 |  | 0.301 |  | 0.9432 |  | | angry | 5177.02 |  | 0.713 |  | 0.9932 |  | | he's | 5177.02 |  | 0.530 |  | 0.9863 |  | | red | 5177.02 |  | 0.685 |  | 0.9958 |  | | peace | 5129.95 |  | 0.482 |  | 0.9878 |  | | showed | 5129.95 |  | 0.929 |  | 0.9924 |  | | led | 5083.74 |  | 0.615 |  | 0.9940 |  | | ordered | 5083.74 |  | 0.780 |  | 0.9906 |  | | show | 5083.74 |  | 0.832 |  | 0.9982 |  | | besides | 5038.35 |  | 0.890 |  | 0.9893 |  | | drew | 5038.35 |  | 0.912 |  | 0.9884 |  | | fear | 5038.35 |  | 0.714 |  | 0.9883 |  | | feet | 5038.35 |  | 0.546 |  | 0.9856 |  | | human | 5038.35 |  | 0.553 |  | 0.9952 |  | | pale | 5038.35 |  | 0.739 |  | 0.9921 |  | | window | 5038.35 |  | 0.338 |  | 0.9637 |  | | pavlovna | 4993.76 |  | 0.330 |  | 0.6674 |  | | study | 4993.76 |  | 0.493 |  | 0.9917 |  | | ball | 4949.96 |  | 0.418 |  | 0.9835 |  | | freedom | 4949.96 |  | 0.275 |  | 0.8970 |  | | lips | 4949.96 |  | 0.658 |  | 0.9808 |  | | marya | 4949.96 |  | 0.256 |  | 0.7322 |  | | whose | 4949.96 |  | 0.770 |  | 0.9687 |  | | everybody | 4906.91 |  | 0.926 |  | 0.9933 |  | | natasha's | 4906.91 |  | 0.483 |  | 0.9774 |  | | second | 4906.91 |  | 0.782 |  | 0.9920 |  | | short | 4906.91 |  | 0.668 |  | 0.9886 |  | | across | 4864.61 |  | 0.654 |  | 0.9970 |  | | bourienne | 4864.61 |  | 0.312 |  | 0.8015 |  | | military | 4864.61 |  | 0.474 |  | 0.9945 |  | | opened | 4864.61 |  | 0.723 |  | 0.9953 |  | | attention | 4823.03 |  | 0.721 |  | 0.9950 |  | | carriage | 4823.03 |  | 0.496 |  | 0.9913 |  | | frightened | 4823.03 |  | 0.747 |  | 0.9948 |  | | prisoners | 4823.03 |  | 0.321 |  | 0.8990 |  | | waiting | 4823.03 |  | 0.717 |  | 0.9929 |  | | yourself | 4823.03 |  | 0.640 |  | 0.9934 |  | | bed | 4782.16 |  | 0.492 |  | 0.9946 |  | | coat | 4782.16 |  | 0.589 |  | 0.9911 |  | | expressed | 4782.16 |  | 0.802 |  | 0.9876 |  | | line | 4782.16 |  | 0.378 |  | 0.9786 |  | | name | 4782.16 |  | 0.563 |  | 0.9949 |  | | perhaps | 4782.16 |  | 0.651 |  | 0.9899 |  | | thoughts | 4782.16 |  | 0.505 |  | 0.9900 |  | | years | 4782.16 |  | 0.613 |  | 0.9960 |  | | alexander | 4741.97 |  | 0.370 |  | 0.9262 |  | | high | 4741.97 |  | 0.743 |  | 0.9965 |  | | kissed | 4741.97 |  | 0.592 |  | 0.9947 |  | | moving | 4741.97 |  | 0.669 |  | 0.9977 |  | | peasants | 4741.97 |  | 0.300 |  | 0.9171 |  | | strength | 4741.97 |  | 0.593 |  | 0.9968 |  | | affairs | 4702.46 |  | 0.661 |  | 0.9976 |  | | black | 4702.46 |  | 0.559 |  | 0.9928 |  | | clear | 4702.46 |  | 0.732 |  | 0.9957 |  | | former | 4702.46 |  | 0.683 |  | 0.9903 |  | | historians | 4702.46 |  | 0.289 |  | 0.7870 |  | | holding | 4702.46 |  | 0.718 |  | 0.9891 |  | | loved | 4702.46 |  | 0.472 |  | 0.9951 |  | | mikhaylovna | 4702.46 |  | 0.311 |  | 0.7736 |  | | says | 4702.46 |  | 0.391 |  | 0.9656 |  | | town | 4702.46 |  | 0.536 |  | 0.9971 |  | | true | 4702.46 |  | 0.550 |  | 0.9936 |  | | usual | 4702.46 |  | 0.756 |  | 0.9958 |  | | events | 4663.60 |  | 0.315 |  | 0.9107 |  | | presence | 4663.60 |  | 0.697 |  | 0.9942 |  | | thinking | 4663.60 |  | 0.680 |  | 0.9906 |  | | work | 4663.60 |  | 0.527 |  | 0.9948 |  | | become | 4625.37 |  | 0.759 |  | 0.9905 |  | | daughter | 4625.37 |  | 0.541 |  | 0.9803 |  | | fine | 4625.37 |  | 0.577 |  | 0.9946 |  | | hundred | 4625.37 |  | 0.500 |  | 0.9897 |  | | appeared | 4587.76 |  | 0.811 |  | 0.9950 |  | | bad | 4587.76 |  | 0.587 |  | 0.9939 |  | | course | 4587.76 |  | 0.669 |  | 0.9859 |  | | grew | 4587.76 |  | 0.837 |  | 0.9929 |  | | believe | 4550.77 |  | 0.551 |  | 0.9955 |  | | doctor | 4550.77 |  | 0.277 |  | 0.8938 |  | | excellency | 4550.77 |  | 0.374 |  | 0.9840 |  | | pleasure | 4550.77 |  | 0.725 |  | 0.9935 |  | | smoke | 4550.77 |  | 0.364 |  | 0.9587 |  | | contrary | 4514.36 |  | 0.786 |  | 0.9953 |  | | fell | 4514.36 |  | 0.721 |  | 0.9949 |  | | money | 4514.36 |  | 0.415 |  | 0.9865 |  | | present | 4514.36 |  | 0.774 |  | 0.9902 |  | | best | 4478.53 |  | 0.704 |  | 0.9823 |  | | reason | 4478.53 |  | 0.604 |  | 0.9938 |  | | someone | 4478.53 |  | 0.642 |  | 0.9913 |  | | either | 4443.27 |  | 0.848 |  | 0.9961 |  | | remember | 4443.27 |  | 0.336 |  | 0.9678 |  | | boy | 4408.55 |  | 0.436 |  | 0.9893 |  | | brother | 4374.38 |  | 0.508 |  | 0.9932 |  | | girl | 4374.38 |  | 0.461 |  | 0.9933 |  | | glad | 4374.38 |  | 0.588 |  | 0.9824 |  | | happiness | 4374.38 |  | 0.465 |  | 0.9885 |  | | important | 4374.38 |  | 0.672 |  | 0.9942 |  | | killed | 4374.38 |  | 0.477 |  | 0.9943 |  | | known | 4374.38 |  | 0.768 |  | 0.9908 |  | | service | 4374.38 |  | 0.542 |  | 0.9850 |  | | third | 4374.38 |  | 0.723 |  | 0.9938 |  | | command | 4340.73 |  | 0.426 |  | 0.9925 |  | | immediately | 4340.73 |  | 0.874 |  | 0.9967 |  | | read | 4340.73 |  | 0.463 |  | 0.9950 |  | | merely | 4307.60 |  | 0.674 |  | 0.9940 |  | | ever | 4274.96 |  | 0.871 |  | 0.9944 |  | | used | 4274.96 |  | 0.847 |  | 0.9972 |  | | bridge | 4242.82 |  | 0.241 |  | 0.8074 |  | | feel | 4242.82 |  | 0.730 |  | 0.9896 |  | | later | 4242.82 |  | 0.910 |  | 0.9963 |  | | sometimes | 4242.82 |  | 0.476 |  | 0.9794 |  | | alpatych | 4211.16 |  | 0.245 |  | 0.4393 |  | | society | 4211.16 |  | 0.499 |  | 0.9928 |  | | air | 4179.96 |  | 0.765 |  | 0.9967 |  | | bagration | 4179.96 |  | 0.264 |  | 0.7236 |  | | followed | 4179.96 |  | 0.803 |  | 0.9977 |  | | please | 4179.96 |  | 0.657 |  | 0.9948 |  | | sides | 4179.96 |  | 0.597 |  | 0.9938 |  | | staff | 4179.96 |  | 0.403 |  | 0.9730 |  | | force | 4149.23 |  | 0.364 |  | 0.9803 |  | | listened | 4149.23 |  | 0.704 |  | 0.9955 |  | | certain | 4118.94 |  | 0.664 |  | 0.9963 |  | | cold | 4118.94 |  | 0.504 |  | 0.9877 |  | | half | 4118.94 |  | 0.641 |  | 0.9938 |  | | husband | 4118.94 |  | 0.417 |  | 0.9719 |  | | necessary | 4118.94 |  | 0.781 |  | 0.9921 |  | | steps | 4118.94 |  | 0.742 |  | 0.9979 |  | | family | 4089.09 |  | 0.563 |  | 0.9963 |  | | helene | 4089.09 |  | 0.349 |  | 0.8381 |  | | quickly | 4089.09 |  | 0.738 |  | 0.9845 |  | | especially | 4059.68 |  | 0.903 |  | 0.9980 |  | | often | 4059.68 |  | 0.803 |  | 0.9973 |  | | state | 4059.68 |  | 0.645 |  | 0.9943 |  | | doing | 4030.68 |  | 0.756 |  | 0.9804 |  | | hear | 4030.68 |  | 0.896 |  | 0.9951 |  | | lost | 4030.68 |  | 0.724 |  | 0.9953 |  | | de | 4002.09 |  | 0.350 |  | 0.9735 |  | | help | 4002.09 |  | 0.692 |  | 0.9960 |  | | held | 3973.91 |  | 0.863 |  | 0.9966 |  | | kind | 3973.91 |  | 0.720 |  | 0.9957 |  | | mademoiselle | 3973.91 |  | 0.327 |  | 0.8412 |  | | together | 3973.91 |  | 0.714 |  | 0.9978 |  | | answer | 3946.12 |  | 0.627 |  | 0.9856 |  | | field | 3946.12 |  | 0.570 |  | 0.9915 |  | | less | 3946.12 |  | 0.526 |  | 0.9886 |  | | voices | 3946.12 |  | 0.580 |  | 0.9939 |  | | won't | 3946.12 |  | 0.660 |  | 0.9938 |  | | light | 3918.72 |  | 0.577 |  | 0.9957 |  | | smiling | 3918.72 |  | 0.741 |  | 0.9828 |  | | women | 3918.72 |  | 0.451 |  | 0.9932 |  | | different | 3891.69 |  | 0.831 |  | 0.9983 |  | | repeated | 3891.69 |  | 0.791 |  | 0.9926 |  | | above | 3865.03 |  | 0.802 |  | 0.9942 |  | | cause | 3865.03 |  | 0.422 |  | 0.9905 |  | | reached | 3865.03 |  | 1.022 |  | 0.9783 |  | | returned | 3865.03 |  | 0.945 |  | 0.9961 |  | | russians | 3865.03 |  | 0.457 |  | 0.9834 |  | | village | 3865.03 |  | 0.425 |  | 0.9884 |  | | full | 3838.74 |  | 0.896 |  | 0.9976 |  | | reply | 3838.74 |  | 0.807 |  | 0.9963 |  | | tone | 3838.74 |  | 0.703 |  | 0.9978 |  | | afraid | 3812.80 |  | 0.508 |  | 0.9842 |  | | myself | 3812.80 |  | 0.523 |  | 0.9812 |  | | kept | 3787.21 |  | 0.802 |  | 0.9946 |  | | free | 3761.97 |  | 0.372 |  | 0.9638 |  | | smiled | 3761.97 |  | 0.759 |  | 0.9883 |  | | soul | 3712.47 |  | 0.729 |  | 0.9974 |  | | answered | 3688.20 |  | 0.922 |  | 0.9934 |  | | fact | 3688.20 |  | 0.667 |  | 0.9974 |  | | glanced | 3688.20 |  | 0.722 |  | 0.9962 |  | | hair | 3688.20 |  | 0.703 |  | 0.9984 |  | | almost | 3664.25 |  | 0.949 |  | 0.9921 |  | | set | 3664.25 |  | 0.912 |  | 0.9968 |  | | times | 3664.25 |  | 0.713 |  | 0.9861 |  | | speaking | 3617.28 |  | 0.884 |  | 0.9855 |  | | history | 3594.24 |  | 0.255 |  | 0.7691 |  | | honor | 3594.24 |  | 0.515 |  | 0.9947 |  | | met | 3594.24 |  | 0.714 |  | 0.9932 |  | | standing | 3594.24 |  | 0.831 |  | 0.9965 |  | | terrible | 3594.24 |  | 0.605 |  | 0.9936 |  | | adjutant | 3571.49 |  | 0.369 |  | 0.9617 |  | | else | 3571.49 |  | 0.906 |  | 0.9967 |  | | faces | 3549.03 |  | 0.614 |  | 0.9973 |  | | strange | 3549.03 |  | 0.688 |  | 0.9941 |  | | itself | 3526.84 |  | 0.700 |  | 0.9968 |  | | rostovs | 3526.84 |  | 0.459 |  | 0.9776 |  | | sound | 3526.84 |  | 0.690 |  | 0.9963 |  | | bolkonski | 3504.94 |  | 0.477 |  | 0.9843 |  | | better | 3483.30 |  | 0.738 |  | 0.9963 |  | | find | 3483.30 |  | 0.771 |  | 0.9944 |  | | gone | 3483.30 |  | 0.817 |  | 0.9956 |  | | world | 3483.30 |  | 0.613 |  | 0.9839 |  | | coming | 3461.93 |  | 0.724 |  | 0.9897 |  | | cannot | 3440.82 |  | 0.561 |  | 0.9970 |  | | silent | 3440.82 |  | 0.855 |  | 0.9962 |  | | trying | 3440.82 |  | 0.861 |  | 0.9958 |  | | ready | 3419.97 |  | 0.713 |  | 0.9965 |  | | russia | 3399.37 |  | 0.496 |  | 0.9937 |  | | tears | 3399.37 |  | 0.545 |  | 0.9896 |  | | conversation | 3379.01 |  | 0.584 |  | 0.9952 |  | | dinner | 3379.01 |  | 0.451 |  | 0.9890 |  | | pierre's | 3379.01 |  | 0.563 |  | 0.9709 |  | | friend | 3358.90 |  | 0.546 |  | 0.9875 |  | | happened | 3358.90 |  | 0.591 |  | 0.9874 |  | | spoke | 3358.90 |  | 0.719 |  | 0.9897 |  | | thousand | 3358.90 |  | 0.422 |  | 0.9755 |  | | became | 3319.38 |  | 0.866 |  | 0.9968 |  | | few | 3319.38 |  | 0.870 |  | 0.9976 |  | | large | 3319.38 |  | 0.904 |  | 0.9977 |  | | seeing | 3319.38 |  | 0.800 |  | 0.9937 |  | | news | 3299.97 |  | 0.492 |  | 0.9861 |  | | far | 3280.78 |  | 0.853 |  | 0.9968 |  | | matter | 3280.78 |  | 0.768 |  | 0.9967 |  | | possible | 3280.78 |  | 0.699 |  | 0.9982 |  | | woman | 3280.78 |  | 0.444 |  | 0.9892 |  | | around | 3261.82 |  | 0.631 |  | 0.9977 |  | | home | 3261.82 |  | 0.556 |  | 0.9975 |  | | forward | 3243.07 |  | 0.579 |  | 0.9922 |  | | past | 3243.07 |  | 0.679 |  | 0.9963 |  | | till | 3243.07 |  | 0.784 |  | 0.9976 |  | | arm | 3224.54 |  | 0.566 |  | 0.9948 |  | | arms | 3206.22 |  | 0.733 |  | 0.9943 |  | | longer | 3206.22 |  | 0.697 |  | 0.9958 |  | | heart | 3188.11 |  | 0.630 |  | 0.9930 |  | | nor | 3188.11 |  | 0.554 |  | 0.9521 |  | | impossible | 3170.20 |  | 0.653 |  | 0.9935 |  | | anyone | 3152.49 |  | 0.764 |  | 0.9961 |  | | given | 3152.49 |  | 0.867 |  | 0.9931 |  | | noticed | 3152.49 |  | 0.694 |  | 0.9968 |  | | small | 3152.49 |  | 0.654 |  | 0.9967 |  | | remained | 3134.97 |  | 0.952 |  | 0.9972 |  | | turning | 3117.65 |  | 0.778 |  | 0.9970 |  | | ah | 3100.52 |  | 0.606 |  | 0.9977 |  | | each | 3100.52 |  | 0.618 |  | 0.9831 |  | | wanted | 3100.52 |  | 0.810 |  | 0.9969 |  | | end | 3066.82 |  | 0.792 |  | 0.9974 |  | | beside | 3050.24 |  | 0.730 |  | 0.9984 |  | | death | 3017.62 |  | 0.513 |  | 0.9970 |  | | movement | 3017.62 |  | 0.364 |  | 0.9731 |  | | soldier | 3017.62 |  | 0.390 |  | 0.9655 |  | | wish | 3017.62 |  | 0.754 |  | 0.9984 |  | | fellow | 3001.57 |  | 0.622 |  | 0.9969 |  | | several | 3001.57 |  | 0.891 |  | 0.9980 |  | | since | 3001.57 |  | 0.834 |  | 0.9938 |  | | rose | 2985.69 |  | 0.763 |  | 0.9875 |  | | talking | 2985.69 |  | 0.861 |  | 0.9983 |  | | tried | 2985.69 |  | 0.780 |  | 0.9968 |  | | wished | 2985.69 |  | 0.781 |  | 0.9906 |  | | i'll | 2969.97 |  | 0.502 |  | 0.9937 |  | | passed | 2954.42 |  | 0.729 |  | 0.9983 |  | | next | 2923.81 |  | 0.808 |  | 0.9930 |  | | stopped | 2923.81 |  | 0.757 |  | 0.9933 |  | | happy | 2908.74 |  | 0.609 |  | 0.9962 |  | | white | 2908.74 |  | 0.564 |  | 0.9977 |  | | action | 2893.82 |  | 0.373 |  | 0.9760 |  | | days | 2893.82 |  | 0.659 |  | 0.9948 |  | | can't | 2879.06 |  | 0.613 |  | 0.9932 |  | | fire | 2849.97 |  | 0.384 |  | 0.9666 |  | | lay | 2849.97 |  | 0.749 |  | 0.9978 |  | | understood | 2849.97 |  | 0.652 |  | 0.9918 |  | | ask | 2821.47 |  | 0.702 |  | 0.9969 |  | | mother | 2821.47 |  | 0.425 |  | 0.9755 |  | | received | 2821.47 |  | 0.797 |  | 0.9968 |  | | orders | 2807.44 |  | 0.533 |  | 0.9949 |  | | drawing | 2793.54 |  | 0.609 |  | 0.9972 |  | | got | 2779.78 |  | 0.867 |  | 0.9956 |  | | really | 2779.78 |  | 0.672 |  | 0.9962 |  | | taking | 2779.78 |  | 0.890 |  | 0.9946 |  | | themselves | 2779.78 |  | 0.670 |  | 0.9895 |  | | road | 2766.15 |  | 0.388 |  | 0.9642 |  | | does | 2739.30 |  | 0.616 |  | 0.9956 |  | | enemy | 2726.06 |  | 0.503 |  | 0.9902 |  | | near | 2726.06 |  | 0.851 |  | 0.9969 |  | | anatole | 2712.96 |  | 0.294 |  | 0.7304 |  | | morning | 2699.98 |  | 0.655 |  | 0.9937 |  | | power | 2699.98 |  | 0.338 |  | 0.9729 |  | | added | 2687.12 |  | 0.780 |  | 0.9962 |  | | cried | 2687.12 |  | 0.663 |  | 0.9980 |  | | leave | 2687.12 |  | 0.708 |  | 0.9954 |  | | wounded | 2687.12 |  | 0.387 |  | 0.9624 |  | | brought | 2674.38 |  | 0.815 |  | 0.9986 |  | | crowd | 2674.38 |  | 0.351 |  | 0.9088 |  | | speak | 2636.89 |  | 0.708 |  | 0.9972 |  | | vasili | 2636.89 |  | 0.358 |  | 0.8484 |  | | continued | 2624.63 |  | 0.810 |  | 0.9968 |  | | among | 2612.48 |  | 0.737 |  | 0.9966 |  | | entered | 2612.48 |  | 0.716 |  | 0.9946 |  | | evening | 2612.48 |  | 0.690 |  | 0.9941 |  | | moved | 2612.48 |  | 0.814 |  | 0.9967 |  | | question | 2576.69 |  | 0.544 |  | 0.9936 |  | | found | 2564.98 |  | 0.805 |  | 0.9978 |  | | sitting | 2564.98 |  | 0.642 |  | 0.9980 |  | | everyone | 2553.37 |  | 0.771 |  | 0.9992 |  | | night | 2553.37 |  | 0.559 |  | 0.9949 |  | | against | 2530.47 |  | 0.695 |  | 0.9909 |  | | rode | 2530.47 |  | 0.484 |  | 0.9759 |  | | during | 2519.17 |  | 0.705 |  | 0.9985 |  | | sent | 2519.17 |  | 0.677 |  | 0.9981 |  | | table | 2507.98 |  | 0.460 |  | 0.9928 |  | | regiment | 2496.88 |  | 0.397 |  | 0.9734 |  | | mind | 2485.88 |  | 0.826 |  | 0.9954 |  | | along | 2474.98 |  | 0.553 |  | 0.9975 |  | | letter | 2464.17 |  | 0.331 |  | 0.9146 |  | | thing | 2464.17 |  | 0.804 |  | 0.9983 |  | | called | 2453.46 |  | 0.805 |  | 0.9974 |  | | officers | 2453.46 |  | 0.498 |  | 0.9873 |  | | son | 2453.46 |  | 0.446 |  | 0.9864 |  | | things | 2442.84 |  | 0.773 |  | 0.9979 |  | | many | 2432.31 |  | 0.675 |  | 0.9906 |  | | part | 2421.87 |  | 0.769 |  | 0.9978 |  | | saying | 2421.87 |  | 0.808 |  | 0.9979 |  | | whether | 2411.52 |  | 0.837 |  | 0.9960 |  | | word | 2411.52 |  | 0.703 |  | 0.9950 |  | | chief | 2380.99 |  | 0.514 |  | 0.9870 |  | | between | 2351.23 |  | 0.742 |  | 0.9985 |  | | alone | 2331.80 |  | 0.630 |  | 0.9884 |  | | taken | 2322.20 |  | 0.665 |  | 0.9914 |  | | petersburg | 2312.68 |  | 0.537 |  | 0.9942 |  | | shouted | 2312.68 |  | 0.566 |  | 0.9935 |  | | shall | 2284.60 |  | 0.579 |  | 0.9869 |  | | horses | 2275.38 |  | 0.479 |  | 0.9956 |  | | petya | 2257.18 |  | 0.254 |  | 0.6176 |  | | wife | 2257.18 |  | 0.510 |  | 0.9844 |  | | want | 2248.19 |  | 0.548 |  | 0.9816 |  | | soon | 2239.27 |  | 0.915 |  | 0.9922 |  | | talk | 2221.63 |  | 0.698 |  | 0.9976 |  | | three | 2212.92 |  | 0.710 |  | 0.9908 |  | | both | 2162.05 |  | 0.772 |  | 0.9958 |  | | hands | 2153.80 |  | 0.671 |  | 0.9960 |  | | troops | 2153.80 |  | 0.536 |  | 0.9854 |  | | great | 2145.61 |  | 0.663 |  | 0.9936 |  | | ran | 2145.61 |  | 0.607 |  | 0.9984 |  | | front | 2129.42 |  | 0.574 |  | 0.9962 |  | | god | 2129.42 |  | 0.557 |  | 0.9971 |  | | also | 2105.58 |  | 0.858 |  | 0.9978 |  | | put | 2082.27 |  | 0.801 |  | 0.9946 |  | | make | 2059.47 |  | 0.848 |  | 0.9921 |  | | may | 2037.17 |  | 0.544 |  | 0.9865 |  | | give | 2029.84 |  | 0.768 |  | 0.9959 |  | | new | 1993.98 |  | 0.687 |  | 0.9974 |  | | commander | 1966.18 |  | 0.436 |  | 0.9754 |  | | dolokhov | 1966.18 |  | 0.267 |  | 0.6620 |  | | horse | 1966.18 |  | 0.418 |  | 0.9669 |  | | seen | 1966.18 |  | 0.696 |  | 0.9890 |  | | that's | 1945.84 |  | 0.540 |  | 0.9930 |  | | anything | 1932.52 |  | 0.813 |  | 0.9941 |  | | toward | 1932.52 |  | 0.824 |  | 0.9984 |  | | anna | 1925.92 |  | 0.332 |  | 0.7448 |  | | boris | 1925.92 |  | 0.309 |  | 0.7596 |  | | order | 1912.86 |  | 0.573 |  | 0.9968 |  | | position | 1906.40 |  | 0.496 |  | 0.9875 |  | | war | 1906.40 |  | 0.453 |  | 0.9928 |  | | side | 1887.27 |  | 0.546 |  | 0.9883 |  | | expression | 1874.73 |  | 0.717 |  | 0.9987 |  | | replied | 1874.73 |  | 0.742 |  | 0.9969 |  | | others | 1868.53 |  | 0.759 |  | 0.9961 |  | | done | 1862.36 |  | 0.743 |  | 0.9954 |  | | gave | 1844.10 |  | 0.853 |  | 0.9981 |  | | stood | 1832.13 |  | 0.723 |  | 0.9966 |  | | place | 1820.31 |  | 0.808 |  | 0.9977 |  | | behind | 1814.45 |  | 0.666 |  | 0.9906 |  | | might | 1791.41 |  | 0.740 |  | 0.9952 |  | | oh | 1791.41 |  | 0.594 |  | 0.9977 |  | | going | 1768.95 |  | 0.769 |  | 0.9930 |  | | feeling | 1763.42 |  | 0.676 |  | 0.9978 |  | | most | 1741.65 |  | 0.766 |  | 0.9973 |  | | already | 1736.29 |  | 0.942 |  | 0.9991 |  | | herself | 1720.41 |  | 0.551 |  | 0.9817 |  | | door | 1704.82 |  | 0.435 |  | 0.9836 |  | | father | 1689.51 |  | 0.505 |  | 0.9874 |  | | every | 1674.47 |  | 0.619 |  | 0.9945 |  | | sat | 1635.64 |  | 0.666 |  | 0.9989 |  | | last | 1630.91 |  | 0.800 |  | 0.9964 |  | | yet | 1626.21 |  | 0.998 |  | 0.9980 |  | | understand | 1616.89 |  | 0.585 |  | 0.9976 |  | | evidently | 1612.27 |  | 0.916 |  | 0.9969 |  | | soldiers | 1598.57 |  | 0.461 |  | 0.9627 |  | | take | 1598.57 |  | 0.770 |  | 0.9970 |  | | battle | 1594.05 |  | 0.429 |  | 0.9632 |  | | get | 1580.66 |  | 0.712 |  | 0.9967 |  | | once | 1580.66 |  | 0.874 |  | 0.9987 |  | | think | 1576.24 |  | 0.733 |  | 0.9937 |  | | denisov | 1546.01 |  | 0.281 |  | 0.7016 |  | | under | 1541.79 |  | 0.720 |  | 0.9946 |  | | chapter | 1537.59 |  | 2.958 |  | 0.9788 |  | | tell | 1504.79 |  | 0.639 |  | 0.9979 |  | | quite | 1500.78 |  | 0.805 |  | 0.9980 |  | | through | 1496.80 |  | 0.726 |  | 0.9962 |  | | it's | 1484.99 |  | 0.569 |  | 0.9984 |  | | let | 1484.99 |  | 0.713 |  | 0.9964 |  | | dear | 1465.70 |  | 0.544 |  | 0.9938 |  | | good | 1465.70 |  | 0.638 |  | 0.9938 |  | | turned | 1465.70 |  | 0.804 |  | 0.9980 |  | | looking | 1443.21 |  | 0.830 |  | 0.9988 |  | | too | 1439.53 |  | 0.779 |  | 0.9981 |  | | much | 1432.22 |  | 0.814 |  | 0.9960 |  | | words | 1428.59 |  | 0.685 |  | 0.9977 |  | | house | 1424.99 |  | 0.571 |  | 0.9940 |  | | took | 1421.40 |  | 0.890 |  | 0.9989 |  | | while | 1414.27 |  | 0.877 |  | 0.9977 |  | | whom | 1389.89 |  | 0.778 |  | 0.9990 |  | | never | 1383.08 |  | 0.692 |  | 0.9989 |  | | told | 1379.69 |  | 0.731 |  | 0.9990 |  | | knew | 1366.33 |  | 0.723 |  | 0.9983 |  | | everything | 1363.03 |  | 0.730 |  | 0.9977 |  | | moment | 1359.75 |  | 0.777 |  | 0.9969 |  | | smile | 1359.75 |  | 0.664 |  | 0.9981 |  | | sonya | 1353.23 |  | 0.311 |  | 0.8218 |  | | officer | 1349.99 |  | 0.380 |  | 0.9407 |  | | round | 1340.37 |  | 0.712 |  | 0.9984 |  | | us | 1334.03 |  | 0.572 |  | 0.9963 |  | | voice | 1330.88 |  | 0.650 |  | 0.9970 |  | | off | 1324.64 |  | 0.775 |  | 0.9990 |  | | suddenly | 1306.24 |  | 0.831 |  | 0.9983 |  | | young | 1276.69 |  | 0.585 |  | 0.9978 |  | | kutuzov | 1253.99 |  | 0.339 |  | 0.7970 |  | | right | 1253.99 |  | 0.659 |  | 0.9965 |  | | russian | 1245.68 |  | 0.509 |  | 0.9923 |  | | love | 1242.94 |  | 0.410 |  | 0.9926 |  | | though | 1237.49 |  | 0.934 |  | 0.9986 |  | | made | 1226.73 |  | 0.862 |  | 0.9969 |  | | being | 1221.42 |  | 0.895 |  | 0.9988 |  | | saw | 1218.78 |  | 0.709 |  | 0.9980 |  | | always | 1216.15 |  | 0.690 |  | 0.9985 |  | | any | 1213.54 |  | 0.750 |  | 0.9986 |  | | nothing | 1213.54 |  | 0.701 |  | 0.9920 |  | | can | 1205.76 |  | 0.678 |  | 0.9958 |  | | must | 1195.54 |  | 0.678 |  | 0.9890 |  | | look | 1187.99 |  | 0.713 |  | 0.9990 |  | | here | 1165.90 |  | 0.695 |  | 0.9990 |  | | general | 1163.49 |  | 0.545 |  | 0.9920 |  | | napoleon | 1163.49 |  | 0.364 |  | 0.8774 |  | | countess | 1158.72 |  | 0.408 |  | 0.9032 |  | | because | 1151.62 |  | 0.562 |  | 0.9769 |  | | way | 1149.28 |  | 0.812 |  | 0.9981 |  | | having | 1142.30 |  | 0.827 |  | 0.9976 |  | | heard | 1133.12 |  | 0.745 |  | 0.9990 |  | | own | 1124.09 |  | 0.888 |  | 0.9986 |  | | even | 1108.63 |  | 0.868 |  | 0.9985 |  | | long | 1102.14 |  | 0.837 |  | 0.9991 |  | | back | 1087.27 |  | 0.683 |  | 0.9983 |  | | should | 1081.02 |  | 0.722 |  | 0.9921 |  | | don't | 1068.74 |  | 0.538 |  | 0.9983 |  | | emperor | 1068.74 |  | 0.383 |  | 0.9169 |  | | people | 1062.70 |  | 0.647 |  | 0.9984 |  | | whole | 1060.70 |  | 0.811 |  | 0.9983 |  | | than | 1058.71 |  | 0.832 |  | 0.9971 |  | | hand | 1046.93 |  | 0.746 |  | 0.9979 |  | | why | 1041.13 |  | 0.583 |  | 0.9964 |  | | am | 1035.40 |  | 0.562 |  | 0.9980 |  | | day | 1029.74 |  | 0.663 |  | 0.9964 |  | | little | 1013.10 |  | 0.626 |  | 0.9954 |  | | yes | 1005.87 |  | 0.529 |  | 0.9954 |  | | head | 995.23 |  | 0.805 |  | 0.9991 |  | | its | 993.48 |  | 0.633 |  | 0.9971 |  | | just | 993.48 |  | 0.910 |  | 0.9990 |  | | other | 989.99 |  | 0.854 |  | 0.9985 |  | | seemed | 967.92 |  | 0.733 |  | 0.9988 |  | | two | 961.32 |  | 0.814 |  | 0.9995 |  | | these | 956.43 |  | 0.736 |  | 0.9986 |  | | such | 954.81 |  | 0.746 |  | 0.9973 |  | | over | 942.06 |  | 0.839 |  | 0.9987 |  | | something | 938.93 |  | 0.701 |  | 0.9993 |  | | another | 929.65 |  | 0.816 |  | 0.9989 |  | | left | 925.07 |  | 0.733 |  | 0.9975 |  | | life | 923.56 |  | 0.494 |  | 0.9924 |  | | away | 917.55 |  | 0.771 |  | 0.9986 |  | | mary | 917.55 |  | 0.364 |  | 0.8036 |  | | where | 913.10 |  | 0.726 |  | 0.9995 |  | | first | 908.69 |  | 0.885 |  | 0.9982 |  | | our | 907.23 |  | 0.487 |  | 0.9961 |  | | felt | 895.71 |  | 0.700 |  | 0.9983 |  | | nicholas | 891.46 |  | 0.369 |  | 0.8336 |  | | say | 881.71 |  | 0.740 |  | 0.9989 |  | | looked | 866.81 |  | 0.838 |  | 0.9966 |  | | count | 858.90 |  | 0.448 |  | 0.9555 |  | | army | 856.29 |  | 0.409 |  | 0.9470 |  | | those | 853.70 |  | 0.752 |  | 0.9990 |  | | same | 849.84 |  | 0.701 |  | 0.9910 |  | | without | 846.02 |  | 0.789 |  | 0.9974 |  | | asked | 844.75 |  | 0.781 |  | 0.9985 |  | | still | 843.49 |  | 0.832 |  | 0.9975 |  | | came | 826.20 |  | 0.883 |  | 0.9982 |  | | come | 826.20 |  | 0.751 |  | 0.9992 |  | | down | 816.64 |  | 0.762 |  | 0.9991 |  | | well | 808.45 |  | 0.723 |  | 0.9986 |  | | has | 799.28 |  | 0.635 |  | 0.9982 |  | | again | 795.90 |  | 0.740 |  | 0.9989 |  | | began | 785.93 |  | 0.786 |  | 0.9992 |  | | moscow | 785.93 |  | 0.459 |  | 0.9707 |  | | rostov | 784.83 |  | 0.311 |  | 0.7222 |  | | see | 771.95 |  | 0.723 |  | 0.9974 |  | | like | 758.46 |  | 0.808 |  | 0.9985 |  | | go | 747.41 |  | 0.623 |  | 0.9944 |  | | room | 744.45 |  | 0.566 |  | 0.9850 |  | | thought | 735.72 |  | 0.709 |  | 0.9971 |  | | men | 731.90 |  | 0.678 |  | 0.9955 |  | | very | 702.73 |  | 0.825 |  | 0.9970 |  | | old | 701.86 |  | 0.583 |  | 0.9942 |  | | your | 695.80 |  | 0.525 |  | 0.9951 |  | | eyes | 682.34 |  | 0.704 |  | 0.9954 |  | | before | 679.87 |  | 0.882 |  | 0.9993 |  | | after | 674.99 |  | 0.834 |  | 0.9991 |  | | some | 667.80 |  | 0.776 |  | 0.9978 |  | | know | 667.02 |  | 0.645 |  | 0.9974 |  | | went | 654.63 |  | 0.806 |  | 0.9978 |  | | french | 641.97 |  | 0.484 |  | 0.9705 |  | | face | 631.91 |  | 0.702 |  | 0.9982 |  | | princess | 616.04 |  | 0.338 |  | 0.7600 |  | | time | 608.73 |  | 0.856 |  | 0.9988 |  | | then | 599.04 |  | 0.797 |  | 0.9966 |  | | we | 584.16 |  | 0.448 |  | 0.9918 |  | | how | 564.86 |  | 0.682 |  | 0.9956 |  | | into | 562.05 |  | 0.821 |  | 0.9983 |  | | about | 558.16 |  | 0.805 |  | 0.9995 |  | | himself | 553.23 |  | 0.770 |  | 0.9984 |  | | do | 543.11 |  | 0.748 |  | 0.9994 |  | | andrew | 538.96 |  | 0.388 |  | 0.8112 |  | | more | 535.38 |  | 0.724 |  | 0.9897 |  | | will | 531.85 |  | 0.567 |  | 0.9943 |  | | man | 520.57 |  | 0.672 |  | 0.9976 |  | | natasha | 513.93 |  | 0.334 |  | 0.7112 |  | | could | 506.09 |  | 0.775 |  | 0.9990 |  | | my | 460.27 |  | 0.543 |  | 0.9965 |  | | out | 457.66 |  | 0.837 |  | 0.9991 |  | | are | 444.33 |  | 0.671 |  | 0.9976 |  | | me | 443.28 |  | 0.537 |  | 0.9969 |  | | if | 436.09 |  | 0.840 |  | 0.9988 |  | | only | 434.74 |  | 0.885 |  | 0.9987 |  | | now | 423.33 |  | 0.724 |  | 0.9991 |  | | would | 413.40 |  | 0.668 |  | 0.9929 |  | | no | 403.07 |  | 0.850 |  | 0.9998 |  | | their | 391.87 |  | 0.658 |  | 0.9983 |  | | been | 382.31 |  | 0.788 |  | 0.9994 |  | | there | 382.31 |  | 0.749 |  | 0.9990 |  | | did | 380.00 |  | 0.801 |  | 0.9995 |  | | when | 377.20 |  | 0.824 |  | 0.9995 |  | | or | 370.27 |  | 0.707 |  | 0.9982 |  | | them | 369.30 |  | 0.723 |  | 0.9989 |  | | up | 363.36 |  | 0.840 |  | 0.9988 |  | | an | 346.83 |  | 0.880 |  | 0.9995 |  | | pierre | 314.20 |  | 0.328 |  | 0.6407 |  | | so | 302.57 |  | 0.840 |  | 0.9992 |  | | prince | 298.41 |  | 0.394 |  | 0.8418 |  | | have | 285.72 |  | 0.689 |  | 0.9986 |  | | which | 274.33 |  | 0.846 |  | 0.9985 |  | | one | 273.80 |  | 0.892 |  | 0.9996 |  | | this | 271.95 |  | 0.872 |  | 0.9988 |  | | who | 262.71 |  | 0.849 |  | 0.9990 |  | | they | 259.09 |  | 0.670 |  | 0.9976 |  | | what | 242.29 |  | 0.778 |  | 0.9983 |  | | by | 236.70 |  | 0.857 |  | 0.9998 |  | | were | 232.70 |  | 0.741 |  | 0.9990 |  | | be | 231.46 |  | 0.821 |  | 0.9996 |  | | from | 209.46 |  | 0.874 |  | 0.9997 |  | | all | 202.62 |  | 0.883 |  | 0.9997 |  | | said | 198.56 |  | 0.640 |  | 0.9769 |  | | is | 169.92 |  | 0.578 |  | 0.9937 |  | | she | 164.95 |  | 0.309 |  | 0.7442 |  | | for | 160.36 |  | 0.844 |  | 0.9997 |  | | you | 156.75 |  | 0.431 |  | 0.9645 |  | | on | 141.00 |  | 0.873 |  | 0.9991 |  | | as | 140.27 |  | 0.831 |  | 0.9977 |  | | but | 139.37 |  | 0.951 |  | 0.9957 |  | | i | 135.84 |  | 0.430 |  | 0.9664 |  | | at | 124.51 |  | 0.878 |  | 0.9980 |  | | him | 121.69 |  | 0.662 |  | 0.9916 |  | | not | 120.68 |  | 0.869 |  | 0.9993 |  | | her | 119.43 |  | 0.288 |  | 0.7105 |  | | it | 108.12 |  | 0.837 |  | 0.9989 |  | | had | 105.18 |  | 0.719 |  | 0.9947 |  | | with | 99.65 |  | 0.885 |  | 0.9961 |  | | was | 76.69 |  | 0.731 |  | 0.9830 |  | | that | 71.51 |  | 0.831 |  | 0.9954 |  | | his | 70.68 |  | 0.603 |  | 0.9737 |  | | in | 63.37 |  | 0.922 |  | 0.9968 |  | | he | 57.18 |  | 0.529 |  | 0.9091 |  | | a | 53.56 |  | 0.899 |  | 0.9987 |  | | of | 37.92 |  | 0.790 |  | 0.9857 |  | | to | 33.85 |  | 0.927 |  | 0.9953 |  | | and | 25.40 |  | 0.912 |  | 0.9464 |  | | the | 16.34 |  | 0.675 |  | 0.8871 |  | |
